# A Formal Rearrangement of Allylic Silanols

**DOI:** 10.3390/molecules26133829

**Published:** 2021-06-23

**Authors:** Ranjeet A. Dhokale, Frederick J. Seidl, Shyam Sathyamoorthi

**Affiliations:** 1 Department of Medicinal Chemistry, University of Kansas, Lawrence, KS 66047, USA; r.dhokale@ku.edu; 2 Independent Researcher, San Bruno, CA 94066, USA; fseidl@alumni.stanford.edu

**Keywords:** rearrangement, organic synthesis, organic methodology

## Abstract

We show that 1M aqueous HCl/THF or NaBH_4_/DMF allows for demercurative ring-opening of cyclic organomercurial synthons into secondary silanol products bearing terminal alkenes. We had previously demonstrated that primary allylic silanols are readily transformed into cyclic organomercurials using Hg(OTf)_2_/NaHCO_3_ in THF. Overall, this amounts to a facile two-step protocol for the rearrangement of primary allylic silanol substrates. Computational investigations suggest that this rearrangement is under thermodynamic control and that the di-tert-butylsilanol protecting group is essential for product selectivity.

## 1. Introduction

**Scheme 1:**
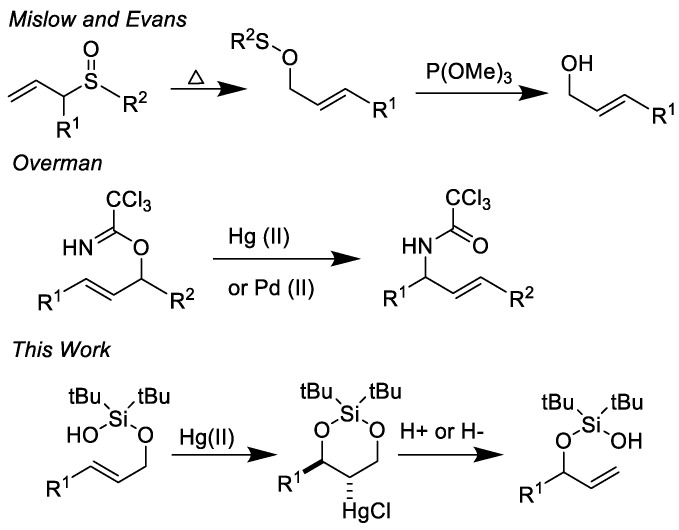
Known allylic rearrangement reactions inspire this work.

Rearrangement reactions can be grouped based on mechanism. One category contains true pericyclic reactions, involving a concerted flow of electrons that results in the breaking of a σ bond, simultaneous rearrangement of a π system, and formation of a new σ bond [1]. These rearrangements are effected thermally or through Lewis acid catalysis, and many landmark reactions (Cope [2], Claisen [3], Ireland-Claisen [4,5,6,7], Mislow-Evans [8], etc.) fall into this category. The second category contains *formal* sigmatropic processes, where the rearrangement proceeds through a discrete organometallic intermediate. A prominent example of this latter process is the Overman transposition of allylic trichloroacetimidates, which is catalyzed by either mercuric or palladium (II) salts (Scheme 1) [9].

**Scheme 2:**
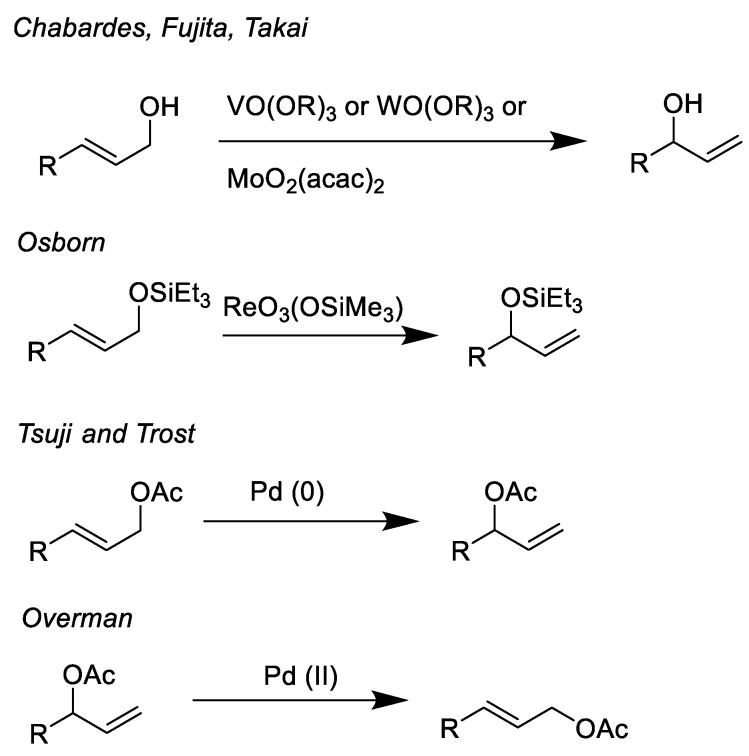
Few protocols exist for the transposition of free and protected allylic alcohols.

In general, there are few rearrangement reactions of free and protected alcohols (Scheme 2). Pioneering work in this area was accomplished using early transition metal oxo species [10,11,12,13,14,15] and with Pd (0)/Pd (II) salts [16,17,18,19]. Elegant mechanistic studies of these reactions have been conducted by Henry [20], Osborn [21,22], and Grubbs [23,24]. Recent advances have been provided by Floreancig [25,26,27], Zakarian [28], Lee [29,30,31], and others [32,33,34,35]. Here, we describe a two-step protocol for a rearrangement of allylic silanols. We recently demonstrated a facile transformation of alkenyl silanols into organomercurial synthons [36]. We now show that these organomercurial species can serve as intermediates for a transposition of the allylic silanol substrate.

## 2. Results and Discussion

### 2.1. Synthetic Investigations

**Scheme 3:**
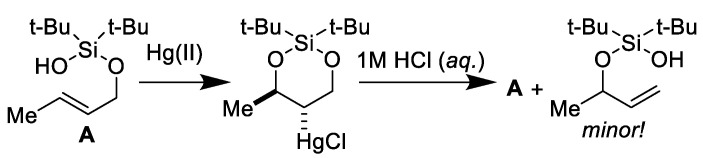
1M aqueous HCl promotes an allylic rearrangement reaction.

This reaction was discovered serendipitously. To remove unreacted mercuric salts after the cyclization reaction, we explored using a 1M aqueous HCl workup. To our consternation, we recovered starting material with trace amounts of the rearranged product (Scheme 3). Repeating this experiment led to the same result. We thus realized that 1M aqueous HCl was promoting a demercuration reaction leading to starting material regeneration. We wondered if we could change the product distribution to favor the rearranged silanol.

All attempts to improve the product distribution with **A** were unsuccessful. However, we observed a dramatic improvement upon switching to an organomercurial substrate with a pendant isopropyl group (Table 1, Entry 1). Increasing the reaction temperature gave identical results, but dropping the temperature to 0 °C led to a decrease in yield and selectivity (Table 1, Entries 2–3). Interestingly, treatment with two equivalents of NaBH_4_ in either DMF or DMSO (Table 1, Entries 4–5) was equally effective in forming rearranged product. Demercuration reactions are known with NaBH_4_, but generally the products consist of mercury simply substituted with H, OH, or I [35]. There was a profound solvent dependence on outcome with markedly worse reactions observed in MeOH, DMA, and THF (Table 1, Entries 6–8). Switching from NaBH_4_ to LiBH_4_ led to marked decomposition of substrate with little discernible product formation (Table 1, Entry 9).

**Scheme 4:**
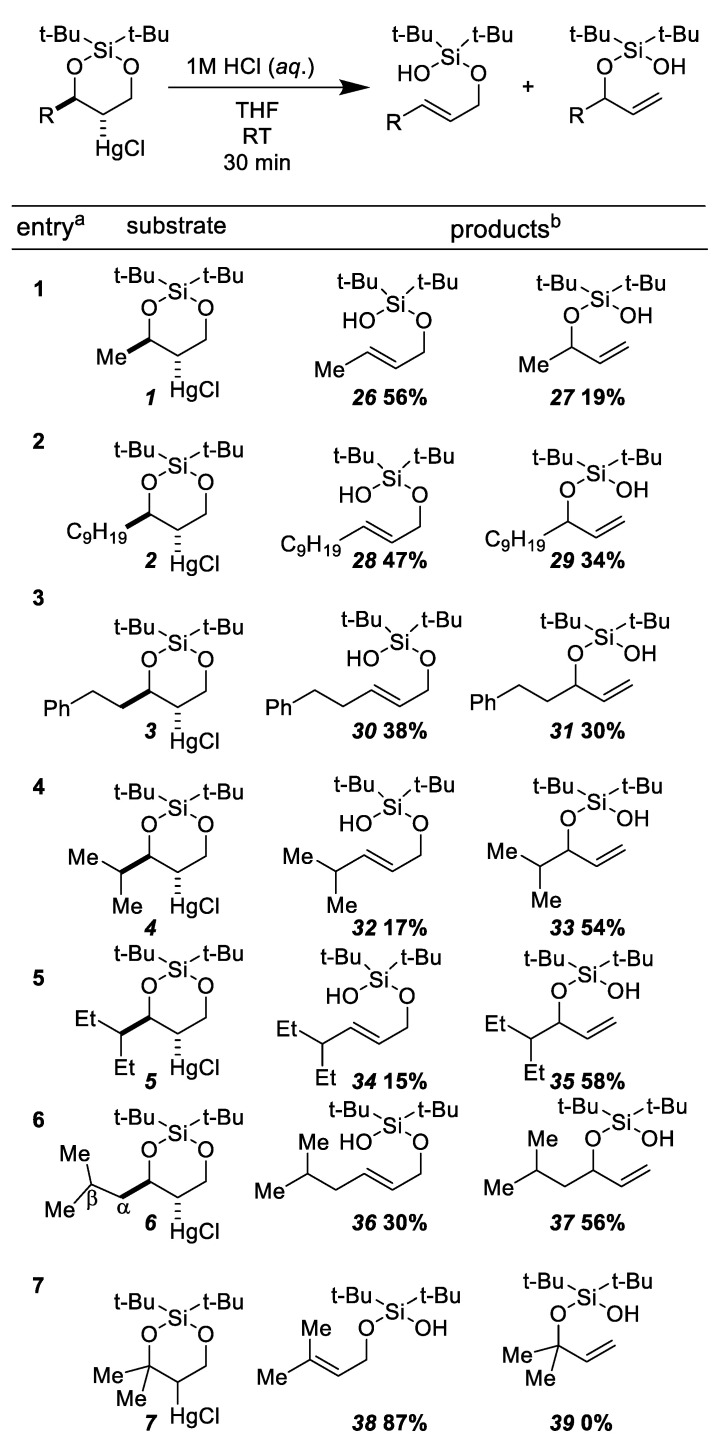
Alkene Substituents Markedly Affect Product Distribution. ^a^ Stereochemistry shown is relative. ^b^ Isolated yields unless otherwise mentioned.

The nature of the substituent *trans* to the HgCl group greatly affected the ratio of the two regioisomeric products (Scheme 4). Generally, as the steric bulk of the linear alkyl chain increased, so too did the yield of the terminal alkene regioisomer (Scheme 4, Entries 1–3). With branching at the α carbon (Scheme 4, Entries 4–5) or at the β carbon (Scheme 4, Entry 6), the terminal alkene regioisomer predominated. When there was competition between formation of a terminal alkene and its tri-substituted isomer, the more substituted olefin formed exclusively (Scheme 4, Entry 7).

**Scheme 5:**
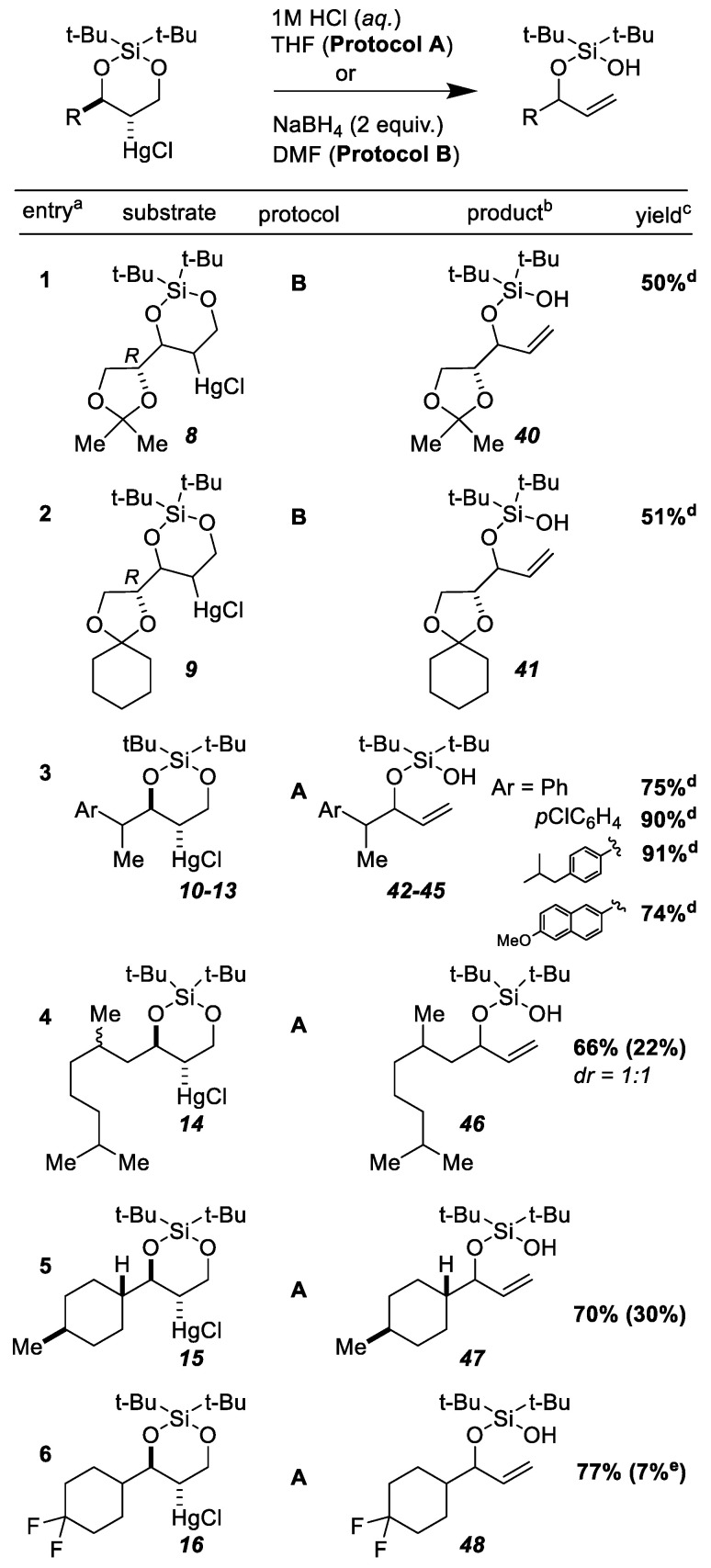

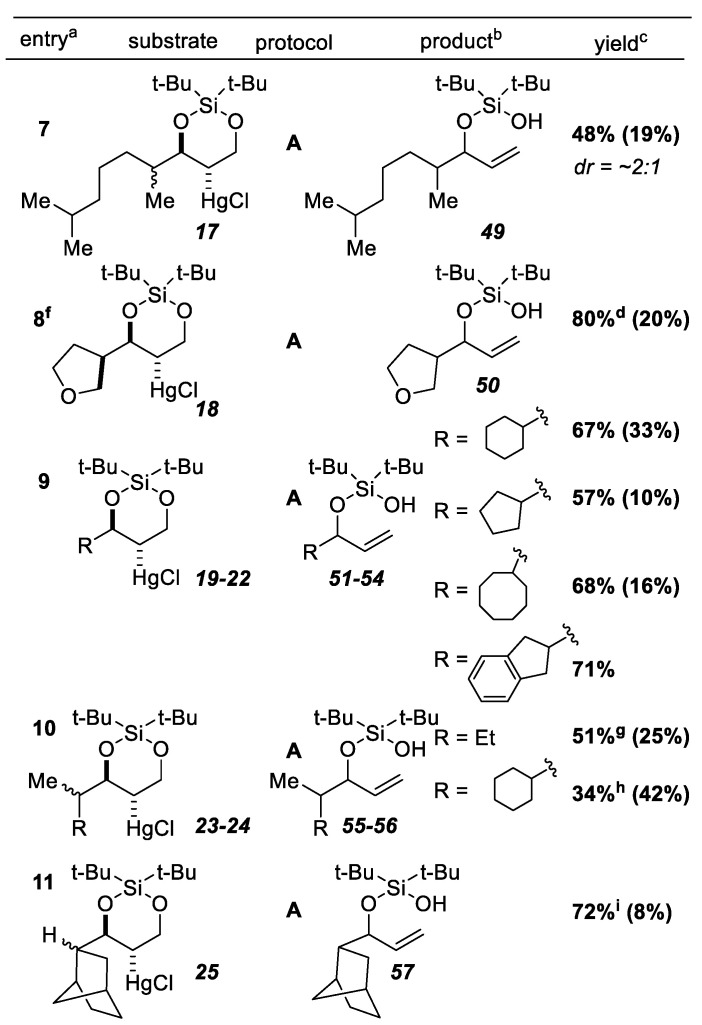
Substrate Scope. ^a^ Relative stereochemistry shown unless explicitly indicated. ^b^ The yield of the internal alkene regio-isomer is shown in parentheses. ^c^ Isolated yield unless otherwise mentioned. ^d^ Single diastereomer but relative stereochemistry unassigned. ^e^ Yield estimated using ^1^H NMR integration against methyl phenyl sulfone as an internal standard. ^f^ CCDC number 2052702. ^g^ dr = ~1.5:1. ^h^ dr = ~2.5:1. ^i^ Mixture of diastereomers.

**Scheme 6:**
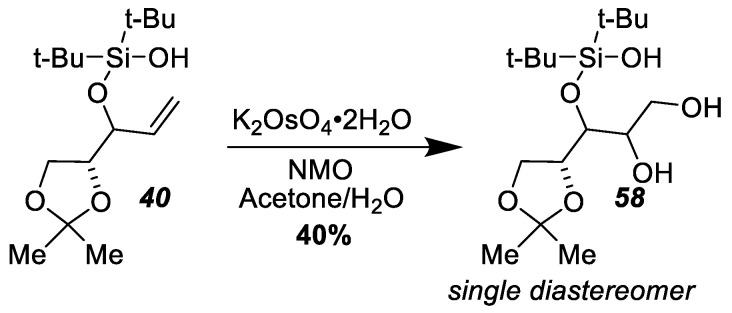
Product **40** serves as a convenient precursor for a protected pentitol.

Our optimized protocols were compatible with a wide array of substrates (Scheme 5). While protocol A (1M aq. HCl/THF) was tested with the majority of substrates, protocol B (NaBH_4_/DMF) was used for those bearing acid sensitive functionality (Scheme 5, Entries 1–2). In all but one instance (Scheme 5, Entry 10), terminal alkene products were greatly favored; in many cases (Scheme 5, Entries 1–3), these were the exclusive product. A variety of functional groups, including ketals (Scheme 5, Entries 1–2), halogens (Scheme 5, Entry 3; Scheme 5, Entry 6), and alkyl ethers (Scheme 5, Entry 3; Scheme 5, Entry 8) were well tolerated. We were pleased to successfully convert product **40** into a single diastereomer of a protected pentitol using a combination of catalytic K_2_OsO_4_•2H_2_O and stoichiometric NMO (Scheme 6).

### 2.2. Mechanistic Studies

In order to better understand the observed selectivity, we turned to DFT calculations using the ORCA software package [37,38]. All calculations were performed using the B3LYP functional [39,40] with D3BJ dispersion correction [41,42] using the RIJCOSX approximation [43]. The def2-TZVP basis set [44] was used, and implicit water solvation was applied using the SMD model [45]. When mercury was present, the def2-ECP [46] was applied automatically. When multiple conformations were possible, a systematic rotor search was performed in Avogadro [47] to identify the lowest energy conformation as a starting point. Further details and atomic coordinates are reported in the Supplementary Materials.

Noting that lower temperature led to lower selectivity for the terminal alkene isomer, we investigated the reaction thermodynamics to determine whether the product distribution was due to equilibrium or kinetics. The simplified reaction mechanism for protocol A is:

organomercury + HCl → alkene + HgCl_2_

**Figure 1:**
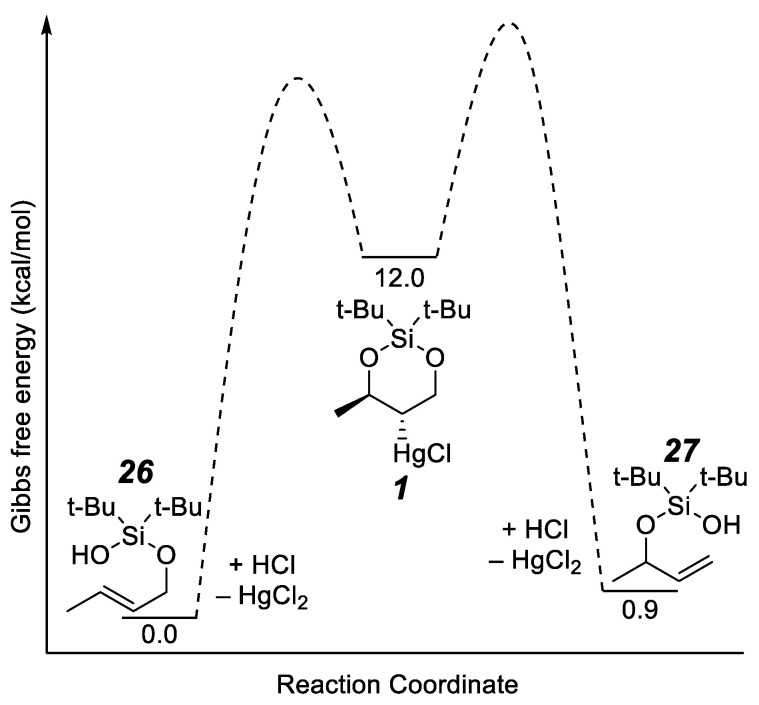
Calculated thermodynamics for methyl substrate **1**.

For methyl substrate **1**, the internal alkene **26** is calculated to be preferred by 0.9 kcal/mol (Figure 1). Experimentally, the observed 2.95:1 ratio favoring **26** over **27** corresponds to an expected ΔG of 0.64 kcal/mol according to a Boltzmann population analysis at room temperature:

$$\frac{A}{B}=\exp\frac{E\left(A\right)-E\left(B\right)}{kT}$$

**Figure 2:**
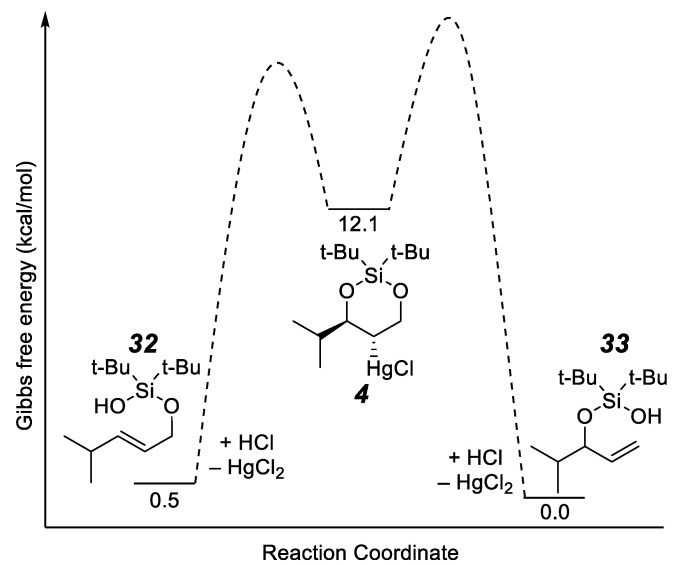
Calculated thermodynamics for isopropyl substrate **4**.

For isopropyl substrate **4**, the terminal alkene **33** is calculated to be preferred by 0.5 kcal/mol (Figure 2). Experimentally, the observed 3.0:1 ratio favoring **33** over **32** corresponds to an expected ΔG of 0.65 kcal/mol by the same equation above. Because these calculated values align reasonably well with experiment, it appears that the observed reaction selectivity is due to equilibrium thermodynamics.

To better understand this thermodynamic preference, we also modeled the deprotected allylic alcohols. For methyl substrate **1**, the internal alkene (*E*)-2-buten-1-ol was 0.02 kcal/mol higher in Gibbs Free Energy than terminal alkene 3-buten-2-ol or nearly isoenergetic. However, for isopropyl substrate **4**, the internal alkene was 0.28 kcal/mol lower in Gibbs Free Energy than the terminal alkene. Importantly, the molecular dipole of the internal alkene is about 1 Debye larger than the dipole of the terminal alkene in both cases, so implicit water solvation significantly stabilizes the internal alkene with its larger molecular dipole. Because the deprotected allylic alcohols fail to account for the observed selectivity, we conclude that the pendant di-tert-butylsilanol group plays a critical role in determining the thermodynamic selectivity of the reaction.

**Figure 3:**
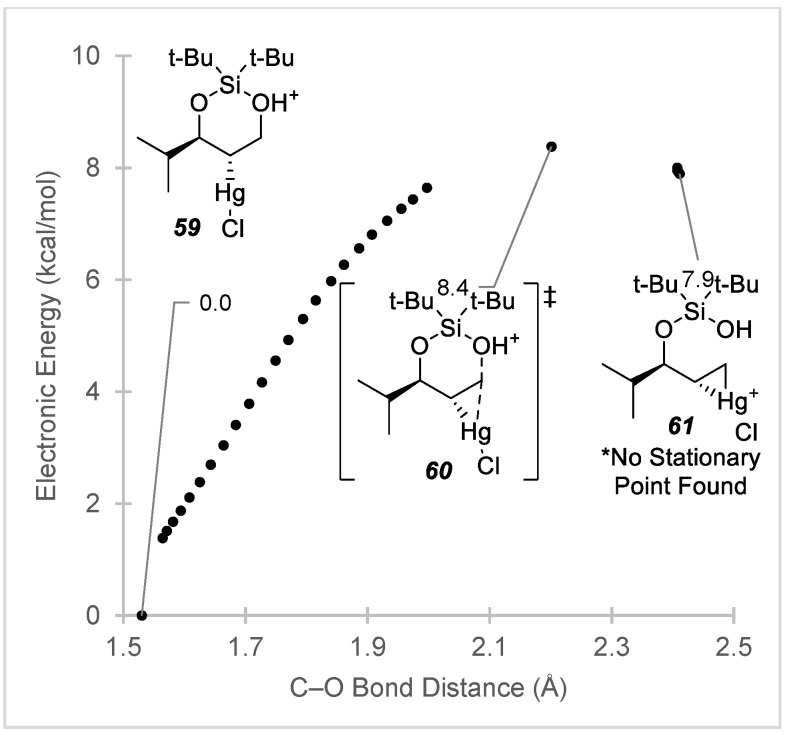
Mercuronium rearrangement of isopropyl substrate 4 (IRC calculation from the transition state).

For the reaction to be under thermodynamic control, it must be reversible. The reaction barriers must be low enough to be overcome rapidly at room temperature (<20 kcal/mol). We began by modeling mercuronium rearrangements based on literature precedent for similar reactions [48]. An intrinsic reaction coordinate (IRC) calculation from the rearrangement transition state leading to the major terminal alkene isomer **33** is included below (Figure 3). Starting from **4**, the silanol oxygen is protonated to form **59**, then the C–O bond is broken with concomitant mercuronium formation. The transition state **60** is very late and product-like, and the potential energy surface in this region is very flat, complicating analysis. A stationary point could not be located for the discrete mercuronium product **61**. Instead, **61** spontaneously engages in a 5-exo ring closure to reversibly re-form an isomeric alkylmercury species. This pathway is ultimately not productive as no side products of this type are isolated experimentally. Most likely, mercuronium **61** is very short-lived and is rapidly abstracted by chloride to form HgCl_2_, which was not modeled. The overall calculated reaction pathway is exothermic by 12 kcal/mol and has an 8 kcal/mol barrier from SM **59** to TS **60**. It follows that the reverse reaction starting from alkene **33** should have a barrier of about 20 kcal/mol, which establishes the reaction as feasibly reversible at room temperature.

The same transition state analysis was performed for the pathway leading to the minor internal alkene product **32**, for which a reaction barrier of only 5.1 kcal/mol was observed. Were this reaction under kinetic control, **32** would be overwhelmingly preferred over **33** with a ΔΔG^‡^ of over 3 kcal/mol. This preference for a more substituted mercuronium ion is expected from Markovnikov selectivity rules due to the partial carbocation character of the mercuronium ion. Overall, for methyl organomercury substrate **1**, the internal alkene **26** is favored by both thermodynamics and kinetics, whereas for isopropyl organomercury substrate **4**, kinetics favors the internal alkene **32**, and thermodynamics favors the terminal alkene **33**.

## 3. Materials and Methods

All reagents were obtained commercially unless otherwise noted. Solvents were purified by passage under 10 psi N_2_ through activated alumina columns. Infrared (IR) spectra were recorded on a Thermo Scientific™ Nicolet™ iS™5 FT-IR Spectrometer (Waltham, MA, USA); data are reported in frequency of absorption (cm^−1^). NMR spectra were recorded on a Bruker Avance (Billerica, MA, USA) 400 operating at 400 and 100 MHz. 1H NMR spectra were recorded at 400 MHz.

Data were recorded as: chemical shift in ppm referenced internally using residue solvent peaks, multiplicity (s = singlet, d = doublet, t = triplet, q = quartet, m = multiplet or overlap of nonequivalent resonances), integration, coupling constant (Hz). ^13^C NMR spectra were recorded at 100 MHz. Exact mass spectra were recorded using an electrospray ion source (ESI) either in positive mode or negative mode and with a time-of-flight (TOF) analyzer on a Waters LCT PremierTM mass spectrometer (Milford, MA, USA) and are given in m/z. TLC was performed on pre-coated glass plates (Merck) and visualized either with a UV lamp (254 nm) or by dipping into a solution of KMnO_4_–K_2_CO_3_ in water followed by heating. Flash chromatography was performed on silica gel (230–400 mesh). Reversed phase HPLC was performed on a Hamilton PRP-1.7 μm, 21.2 × 250 mm, C18 column. Hg(OTf)_2_ was purchased from either Alfa Aesar (Ward Hill, MA, USA) or Strem Chemicals (Newburyport, MA, USA). Di-tert-butylsilyl Bis(trifluoromethanesulfonate) was purchased from either TCI America (Portland, OR, USA) or from Sigma-Aldrich (St. Louis, MO, USA).

## 4. Conclusions

In summary, we presented a two-step protocol for the rearrangement of allylic silanols. We had previously demonstrated that primary allylic silanols are readily transformed into cyclic organomercurials using Hg(OTf)_2_/NaHCO_3_ in THF. Here, we show that using either 1M aqueous HCl/THF or NaBH_4_/DMF allows for demercurative ring-opening to form secondary silanol products bearing terminal alkenes. Computational investigations suggest that this rearrangement is under thermodynamic control and that the di-tert-butylsilanol protecting group is essential for product selectivity.

## Figures and Tables

**Table 1:**
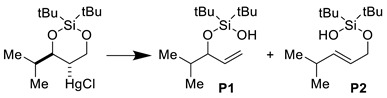
Optimization of this allylic rearrangement.

Entry	Reagent	Solvent	Temp.,Time	P1/P2 ^a^
1	1M HCl (aqueous)	THF	RT, 30 min	60/20
2	1M HCl (aqueous)	THF	35 °C, 30 min	60/20
3	1M HCl (aqueous)	THF	0 °C, 30 min	50/22
4	NaBH_4_ (2 equiv)	DMF	RT, 30 min	64/20
5	NaBH_4_ (2 equiv)	DMSO	RT, 30 min	62/20
6	NaBH_4_ (2 equiv)	MeOH	RT, 30 min	56/11
7	NaBH_4_ (2 equiv)	DMA	RT, 30 min	6/4
8	NaBH_4_ (2 equiv)	THF	RT, 30 min	43/7
9	LiBH_4_ (2 equiv)	THF	RT, 30 min	10/0

^a^ Yield estimated using ^1^H NMR integration against methyl phenyl sulfone as an internal standard.

## Data Availability

Requests for data not shown in the supporting information should be directed to the corresponding author (ssathyam@ku.edu).

## Supplementary Materials

The following are available online: I. General Considerations, II. Characterization of Previously Unreported Substrates, III. General Procedures for Allylic Rearrangement Reactions, IV. Characterization of Allylic Rearrangement Products, V. Dihydroxylation Procedure, VI. Crystal Structure Data for **18** (CCDC: 2052702), VII. Computational Procedures and Atomic Coordinates, VIII. NMR Spectra.

## Sample Availability

Upon request to the corresponding author (ssathyam@ku.edu).
