# Supplementary material for: A Formal Rearrangement of Allylic Silanols
Source: Molecules. 2021 Jun 23;26(13):3829. doi: 10.3390/molecules26133829 (PMC8270268; doi:10.3390/molecules26133829)

Supporting Information

A Formal Rearrangement of Allylic Silanols

Dr. Ranjeet A. Dhokale, Dr. Frederick J. Seidl, and Professor Shyam Sathyamoorthi

Contents

I. General Considerations
II. Characterization of Previously Unreported Substrates
III. General Procedures for Allylic Rearrangement Reactions
IV. Characterization of Allylic Rearrangement Products
V. Dihydroxylation Procedure
VI. Crystal Structure Data for **18** (CCDC: 2052702)
VII. Computational Procedures and Atomic Coordinates
VIII. NMR Spectra

I. General Considerations:

All reagents were obtained commercially unless otherwise noted. Solvents were purified by passage under 10 psi N_2 through activated alumina columns. Infrared (IR) spectra were recorded on a Thermo Scientific™ Nicolet™ iS™5 FT-IR Spectrometer; data are reported in frequency of absorption (cm^{-1}). NMR spectra were recorded on a Bruker Avance 400 operating at 400 and 100 MHz. ^{1}H NMR spectra were recorded at 400 MHz. Data are recorded as: chemical shift in ppm referenced internally using residue solvent peaks, multiplicity (s = singlet, d = doublet, t = triplet, q = quartet, m = multiplet or overlap of nonequivalent resonances), integration, coupling constant (Hz). ^{13}C NMR spectra were recorded at 100 MHz. Exact mass spectra were recorded using an electrospray ion source (ESI) either in positive mode or negative mode and with a time-of-flight (TOF) analyzer on a Waters LCT PremierTM mass spectrometer and are given in m/z. TLC was performed on pre-coated glass plates (Merck) and visualized either with a UV lamp (254 nm) or by dipping into a solution of $KMnO_4$–K_2CO_3 in water followed by heating. Flash chromatography was performed on silica gel (230-400 mesh). Reversed phase HPLC was performed on a Hamilton PRP-1.7 µm, 21.2 x 250 mm, C18 column. $Hg(OTf)_2$ was purchased from either Alfa Aesar or Strem Chemicals. Di-*tert*-butylsilyl Bis(trifluoromethanesulfonate) was purchased from either TCI America or from Sigma-Aldrich.

II. Characterization of Previously Unreported Substrates

Note 1: Substrates were synthesized according to previously reported procedures. See Org. Lett. 2020, 22, 21, 8665–8669.

Note 2: During ESI-MS, in almost all cases, we observed cleavage of the carbon mercury bond into a carbocation fragment. A representative mass spectrum of this phenomenon is found in Org. Lett. 2020, 22, 21, 8665–8669.

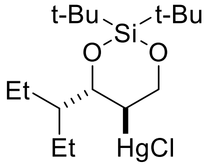

2,2-di-*tert*-butyl-4-(pentan-3-yl)-1,3,2-dioxasilinan-5-yl)mercury(II) chloride

Compound 5: single *trans* diastereomer; purified using a gradient of 0 to 10% EtOAc in hexanes; (white solid, 17% yield); ^{1}H NMR (400 MHz, $CDCl_3$) δ 4.42 – 4.31 (m, 2H), 4.25 (dd, *J* = 10.9, 4.3 Hz, 1H), 3.11 (ddd, *J* = 12.5, 11.3, 4.2 Hz, 1H), 1.56 – 1.38 (m, 4H), 1.28 (ddq, *J* = 14.5, 9.2, 7.3 Hz, 1H), 0.97 (s, 9H), 0.94 – 0.86 (m, 15H). ^{13}C NMR (101 MHz, $CDCl_3$) δ 67.8, 58.1, 51.9, 27.5, 27.1, 23.0, 22.9, 20.8, 20.0, 12.8, 11.9. IR 2956, 1377, 1067, 782 cm^{-1}.

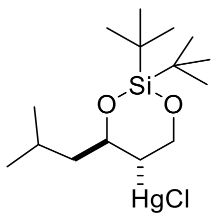

2,2-di-*tert*-butyl-4-isobutyl-1,3,2-dioxasilinan-5-yl)mercury(II) chloride

Compound 6: single diastereomer; purified using a gradient of 0 to 10% EtOAc in hexanes; (White solid, 53%); ^{1}H NMR (400 MHz, Chloroform-*d*) δ 4.48 – 4.32 (m, 2H), 4.25 (dd, *J* = 11.1, 4.0 Hz, 1H), 2.99 (ddd, *J* = 12.8, 11.0, 4.0 Hz, 1H), 2.09 – 1.95 (m, 1H), 1.63 (ddd, *J* = 13.2, 10.6, 4.1 Hz, 1H), 1.29 (ddd, *J* = 12.9, 9.4, 2.5 Hz, 1H), 1.04 (s, 9H), 0.99 (s, 9H), 0.96 (d, *J* = 6.7 Hz, 6H).^{13}C NMR (101 MHz, Chloroform-*d*) δ 74.3, 67.1, 60.6, 50.8, 27.4, 27.0, 24.3, 23.6, 22.8, 21.3, 19.6. IR 2950, 1471, 1075, 650 cm^{-1}.

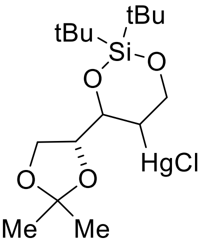

(2,2-di-*tert*-butyl-4-((*R*)-2,2-dimethyl-1,3-dioxolan-4-yl)-1,3,2-dioxasilinan-5-yl)mercury(II) chloride

Compound 8: single diastereomer, relative stereochemistry unassigned; purified using a gradient of 0 to 20% EtOAc in hexanes; (clear oil, 30% yield); ^{1}H NMR (400 MHz, $CDCl_3$) δ 4.38 – 4.19 (m, 2H), 4.10 (dd, *J* = 9.0, 6.4 Hz, 1H), 4.04 – 3.95 (m, 2H), 3.67 (ddd, *J* = 9.2, 6.4, 4.7 Hz, 1H), 2.59 (ddd, *J* = 12.6, 11.4, 4.4 Hz, 1H), 1.43 (s, 3H), 1.35 – 1.24 (m, 3H), 0.97 (s, 9H), 0.88 (s, 9H). ^{13}C NMR (101 MHz, $CDCl_3$) δ 110.9, 78.9, 78.2, 68.0, 67.4, 53.0, 27.4, 27.1, 27.0, 25.0, 22.8, 19.7. IR 2945, 1382, 1078, 833 cm^{-1}.

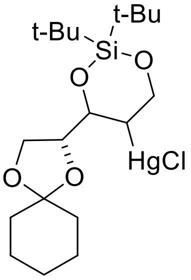

(2,2-di-*tert*-butyl-4-((*R*)-1,4-dioxaspiro[4.5]decan-2-yl)-1,3,2-dioxasilinan-5-yl)mercury(II) chloride

Compound 9: single diastereomer, relative stereochemistry unassigned; purified using a gradient of 0 to 20% EtOAc:Hexanes; (white foam, 28% yield); ^{1}H NMR (400 MHz, $CDCl_3$) δ 4.41 (dd, *J* = 12.6, 11.3 Hz, 1H), 4.33 (dd, *J* = 11.3, 4.3 Hz, 1H), 4.18 (dd, *J* = 8.9, 6.3 Hz, 1H), 4.09 (dd, *J* = 11.3, 9.1 Hz, 1H), 4.03 (dd, *J* = 8.9, 5.5 Hz, 1H), 3.80 – 3.72 (m, 1H), 2.72 (ddd, *J* = 12.5, 11.3, 4.4 Hz, 1H), 1.76 (ddd, *J* = 15.7, 9.7, 3.3 Hz, 2H), 1.61 (ddd, *J* = 23.4, 10.6, 5.0 Hz, 6H), 1.47 –

1.39 (m, 2H), 1.04 (s, 9H), 0.99 (s, 9H). ^{13}C NMR (101 MHz, $CDCl_3$) δ 111.7, 79.8, 78.1, 67.8, 67.4, 53.4, 36.5, 34.6, 27.9, 26.2, 24.9, 24.3, 23.7, 22.8, 19.7. IR 2866, 1477, 1047, 829 cm^{-1}.

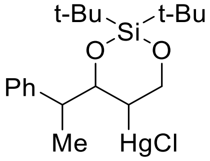

(2,2-di-*tert*-butyl-4-(1-phenylethyl)-1,3,2-dioxasilinan-5-yl)mercury(II) chloride

Compound 10: single diastereomer, relative stereochemistry unassigned; purified using a gradient of 0 to 20% EtOAc:Hexanes; (white solid, 67% yield); ^{1}H NMR (400 MHz, $CDCl_3$) δ 7.41 – 7.21 (m, 5H), 4.58 (dd, J = 11.2, 3.6 Hz, 1H), 4.31 – 4.07 (m, 2H), 3.15 (qd, J = 7.0, 3.6 Hz, 1H), 2.44 (ddd, J = 12.5, 11.2, 4.7 Hz, 1H), 1.36 (d, J = 7.1 Hz, 3H), 1.02 (s, 9H), 0.94 (s, 9H). ^{13}C NMR (101 MHz, $CDCl_3$) δ 141.8, 129.5, 128.0, 127.7, 81.7, 67.7, 52.0, 46.2, 27.7, 27.1, 22.6, 20.0, 13.8. IR 2863, 1477, 1060, 772 cm^{-1}.

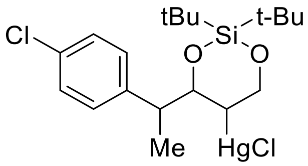

(2,2-di-*tert*-butyl-4-(1-(4-chlorophenyl)ethyl)-1,3,2-dioxasilinan-5-yl)mercury(II) chloride

Compound 11: single diastereomer, relative stereochemistry unassigned; purified using a gradient of 0 to 20% EtOAc:hexanes followed by preparative thin layer chromatography (50% DCM:hexanes); (white foam, 40% yield); ^{1}H NMR (400 MHz, $CDCl_3$) δ 7.29 – 7.24 (m, 2H), 7.24 – 7.17 (m, 2H), 4.53 (dd, J = 11.2, 3.4 Hz, 1H), 4.25 (dd, J = 12.7, 11.1 Hz, 1H), 4.16 (dd, J = 11.1, 4.5 Hz, 1H), 3.01 (qd, J = 7.0, 3.3 Hz, 1H), 2.39 (ddd, J = 12.6, 11.1, 4.5 Hz, 1H), 1.35 (d, J = 7.0 Hz, 3H), 1.01 (s, 9H), 0.91 (s, 9H). ^{13}C NMR (101 MHz, $CDCl_3$) δ 140.2, 133.4, 129.6, 129.3, 80.9, 67.6, 53.8, 46.7, 27.7, 27.0, 23.1, 20.0, 15.4. IR 2973, 1353, 1229, 1062 cm^{-1}.

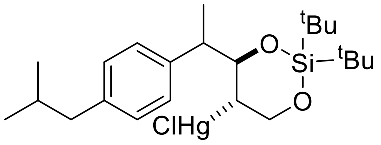

2,2-di-*tert*-butyl-4-((*R*)-1-(4-isobutylphenyl)ethyl)-1,3,2-dioxasilinan-5-yl)mercury(II) chloride

Compound 12: Single diastereomer, relative stereochemistry unassigned; purified using a gradient of 0 to 10% EtOAc in hexanes; (White solid, 73%); ^{1}H NMR (400 MHz, Chloroform-*d*) δ 7.28 – 7.17 (m, 4H), 4.65 (dd, J = 11.3, 3.6 Hz, 1H), 4.38 – 4.21 (m, 2H), 3.24 (dd, J = 7.0, 3.6 Hz, 1H), 2.52 (d, J = 7.2 Hz, 3H), 1.91 (dt, J = 13.4, 6.8 Hz, 1H), 1.43 (d, J = 7.0 Hz, 3H), 1.12 (s, 9H), 1.03 (s, 9H), 0.95 (dd, J = 6.6, 2.3 Hz, 6H). ^{13}C NMR (101 MHz, Chloroform-*d*) δ 141.5, 138.9, 130.4, 127.5, 81.7, 67.7, 51.4, 45.6, 45.0, 30.0, 27.6, 27.0, 23.0, 22.49, 22.42, 20.0, 13.3. IR 3006, 1275, 1057, 750 cm^{-1}.

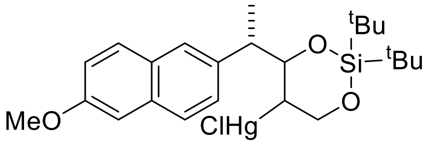

2,2-di-*tert*-butyl-4-((*R*)-1-(6-methoxynaphthalen-2-yl)ethyl)-1,3,2-dioxasilinan-5-yl)mercury(II) chloride

Compound 13: Single diastereomer but absolute stereochemistry unassigned; purified using a gradient of 0 to 10% EtOAc in hexanes; (White solid, 38%); ^{1}H NMR (400 MHz, Chloroform-*d*) δ 7.84 – 7.64 (m, 3H), 7.46 (dd, *J* = 8.4, 1.8 Hz, 1H), 7.25 – 7.13 (m, 2H), 4.75 (dd, *J* = 11.1, 3.6 Hz, 1H), 4.38 – 4.17 (m, 2H), 3.95 (s, 3H), 3.38 – 3.26 (m, 1H), 2.51 (ddd, *J* = 12.6, 11.1, 4.5 Hz, 1H), 1.54 (d, *J* = 7.0 Hz, 3H), 1.14 (s, 9H), 1.06 (s, 9H). ^{13}C NMR (101 MHz, Chloroform-*d*) δ 157.8, 137.0, 134.0, 129.3, 129.1, 128.1, 126.7, 126.3, 119.4, 105.7, 81.4, 67.8, 55.3, 52.5, 46.5, 27.7, 27.1, 23.1, 20.0, 14.3. IR 2933, 1605, 1275, 750 cm^{-1}.

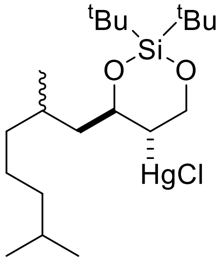

2,2-di-*tert*-butyl-4-(2,6-dimethylheptyl)-1,3,2-dioxasilinan-5-yl)mercury(II) chloride

Compound 14 (*dr* = 1:1): purified using a gradient of 0 to 10% EtOAc in hexanes; (Colorless thick oil, 46%); ^{1}H NMR (400 MHz, Chloroform-*d*) δ 4.46 – 4.34 (m, 2H), 4.25 (dd, *J* = 11.2, 4.0 Hz, 1H), 2.99 (ddt, *J* = 12.8, 11.0, 4.3 Hz, 1H), 1.88 (m, 1H), 1.74 – 1.58 (m, 1H), 1.56 – 1.48 (m, 1H), 1.46 – 1.38 (m, 1H), 1.31 – 1.14 (m, 6H), 1.04 (s, 9H), 0.99 (s, 9H), 0.94 (dd, *J* = 6.7, 2.9 Hz, 3H), 0.88 (d, *J* = 6.6 Hz, 6H); ^{13}C NMR (101 MHz, Chloroform-*d*) δ 74.4, 73.9, 67.19, 67.16, 60.7, 60.5, 49.3, 48.7, 39.2, 39.0, 37.9, 35.8, 29.3, 28.7, 27.9, 27.8, 27.42, 27.40, 27.2, 27.0, 26.9, 24.7, 22.88, 22.84, 22.7, 22.66, 22.63, 22.5, 20.5, 19.6, 19.1. IR 2929, 1473, 1021, 825, 652 cm^{-1}.

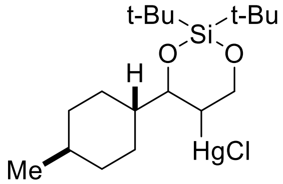

(2,2-di-*tert*-butyl-4-((1*r*,4*r*)-4-methylcyclohexyl)-1,3,2-dioxasilinan-5-yl)mercury(II) chloride

Compound 15: single diastereomer; purified using a gradient of 0 to 20% EtOAc:hexanes; (white foam, 50% yield); ^{1}H NMR (400 MHz, $CDCl_3$) δ 4.43 (dd, *J* = 12.6, 11.0 Hz, 1H), 4.33 (dd, *J* = 11.0, 4.3 Hz, 1H), 4.24 (dd, *J* = 11.4, 1.8 Hz, 1H), 3.14 (ddd, *J* = 12.5, 11.3, 4.3 Hz, 1H), 1.85 – 1.72 (m, 2H), 1.69 – 1.59 (m, 3H), 1.50 – 1.26 (m, 5H), 1.05 (s, 9H), 1.01 (s, 9H), 0.91 (d, *J* = 6.6 Hz, 3H). ^{13}C NMR (101 MHz, $CDCl_3$) δ 80.0, 67.6, 57.7, 47.7, 35.0, 34.8, 32.6, 30.2, 27.5, 27.1, 25.0, 23.0, 22.5, 20.0. IR 2913, 1465, 1082, 988 cm^{-1}.

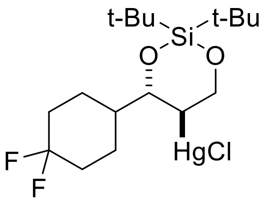

2,2-di-*tert*-butyl-4-(4,4-difluorocyclohexyl)-1,3,2-dioxasilinan-5-yl)mercury(II) chloride

Compound 16: single diastereomer; purified using a gradient of 0 to 20% EtOAc:hexanes; (white foam, 15% yield); ^{1}H NMR (400 MHz, $CDCl_3$) δ 4.44 (dd, *J* = 12.5, 11.0 Hz, 1H), 4.39 – 4.26 (m, 2H), 3.12 (ddd, *J* = 12.5, 11.3, 4.3 Hz, 1H), 2.19 (t, *J* = 15.3 Hz, 2H), 1.97 (dt, *J* = 15.7, 12.8 Hz, 1H), 1.87 – 1.63 (m, 5H), 1.51 (m, 1H), 1.03 (d, *J* = 16.8 Hz, 18H). ^{13}C NMR (101 MHz, $CDCl_3$) δ 79.0, 67.4, 57.7, 45.7, 33.89 – 32.82 (m), 27.5, 27.0, 26.5 (d, *J* = 10 Hz), 23.0, 21.3 (d, *J* = 10 Hz), 19.9. IR 2866, 1365, 1088, 823, 653 cm^{-1}

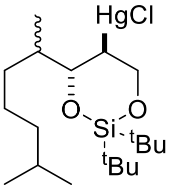

2,2-di-*tert*-butyl-4-(6-methylheptan-2-yl)-1,3,2-dioxasilinan-5-yl)mercury(II) chloride

Compound 17 (Mixture of diastereomers): purified using a gradient of 0 to 10% EtOAc:hexanes; (Colorless thick oil, 45%); ^{1}H NMR (400 MHz, Chloroform-*d*) δ 4.47 – 4.21 (m, 3H), 3.12 (dddd, *J* = 12.5, 11.1, 8.6, 4.3 Hz, 1H), 1.65 – 1.56 (m, 1H), 1.55 – 1.50 (m, 1H), 1.46 – 1.30 (m, 2H), 1.29 – 1.14 (m, 3H), 1.08 (d, *J* = 6.7 Hz, 3H), 1.04 (s, 9H), 1.00 (s, 9H), 0.96 (d, *J* = 6.6 Hz, 1H), 0.92 – 0.90 (m, 3H), 0.90 – 0.88 (m, 3H). ^{13}C NMR (101 MHz, Chloroform-*d*) δ 81.1, 67.6, 57.2, 43.2, 42.6, 39.2, 30.0, 28.0, 27.55, 27.50, 27.0, 25.3, 25.0, 23.0, 22.7, 22.4, 20.0, 16.9, 13.1. IR 2931, 1469, 1064, 824, 651 cm^{1}.

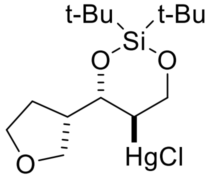

2,2-di-*tert*-butyl-4-(tetrahydrofuran-3-yl)-1,3,2-dioxasilinan-5-yl)mercury(II) chloride

Compound 18: single diastereomer, relative stereochemistry established by single molecule X-ray diffraction (see Section VI); purified using a gradient of 0 to 20% EtOAc:hexanes; (white solid, 52% yield); ^{1}H NMR (400 MHz, $CDCl_3$) δ 4.33 (ddd, *J* = 11.3, 8.8, 3.8 Hz, 2H), 4.22 (dd, *J* = 11.2, 4.2 Hz, 1H), 3.87 – 3.68 (m, 4H), 2.82 (ddd, *J* = 12.5, 11.2, 4.2 Hz, 1H), 2.44 – 2.30 (m, 1H), 2.03 – 1.86 (m, 2H), 1.04 – 0.86 (m, 18H). ^{13}C NMR (101 MHz, $CDCl_3$) δ 78.4, 69.1, 68.6, 67.3, 56.9, 47.9, 29.3, 27.5, 27.1, 22.9, 19.9. IR 2855, 1459, 1071, 829 cm^{-1}.

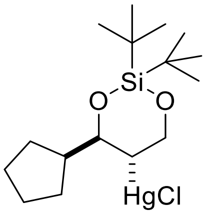

2,2-di-*tert*-butyl-4-cyclopentyl-1,3,2-dioxasilinan-5-yl)mercury(II) chloride

Compound 20: single diastereomer; purified using a gradient of 0 to 10% EtOAc:hexanes; (White solid, 30%); ^{1}H NMR (400 MHz, Chloroform-*d*) δ 4.39 – 4.14 (m, 3H), 2.96 (ddd, *J* = 12.7, 11.1, 4.2 Hz, 1H), 1.94 (dt, *J* = 7.8, 4.0 Hz, 1H), 1.72 – 1.62 (m, 1H), 1.52-1.61 (m, 5H), 1.48-1.42 (m, 2H), 0.97 (s, 9H), 0.92 (s, 9H); ^{13}C NMR (101 MHz, Chloroform-*d*) δ 78.5, 67.4, 59.0, 49.4, 29.7, 27.5, 27.1, 26.1, 25.8, 20.0. IR 2857, 1473, 1056, 825 cm^{-1}.

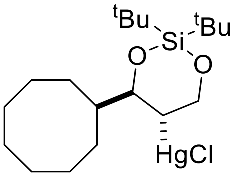

2,2-di-*tert*-butyl-4-cyclooctyl-1,3,2-dioxasilinan-5-yl)mercury(II) chloride

Compound 21: single diastereomer; purified using a gradient of 0 to 10% EtOAc:hexanes; (White solid, 24%); ^{1}H NMR (400 MHz, Chloroform-*d*) δ 4.48 – 4.19 (m, 3H), 3.16 (ddd, *J* = 12.6, 11.2, 4.4 Hz, 1H), 1.85-1.75 (m, 2H), 1.75 – 1.59 (m, 7H), 1.58 – 1.48 (m, 6H), 1.06 (s, 9H), 1.02 (s, 9H).^{13}C NMR (101 MHz, Chloroform-*d*) δ 82.4, 67.7, 57.6, 47.4, 32.0, 27.5, 27.1, 27.0, 26.8, 26.7, 26.5, 26.1, 25.7, 23.0, 20.0. IR 2928, 1473, 1071, 825, 764, 651 cm^{-1}.

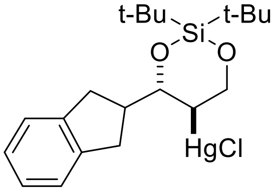

2,2-di-*tert*-butyl-4-(2,3-dihydro-1*H*-inden-2-yl)-1,3,2-dioxasilinan-5-yl)mercury(II) chloride

Compound 22: single diastereomer; Purified using preparative TLC with 20% EtOAc:Hex; (colorless oil, 35% yield); ^{1}H NMR (400 MHz, $CDCl_3$) δ 7.18 – 7.03 (m, 4H), 4.43 (dd, *J* = 11.2, 3.5 Hz, 1H), 4.36 (dd, *J* = 12.6, 11.1 Hz, 1H), 4.27 – 4.21 (m, 1H), 3.14 – 2.79 (m, 5H), 2.65 – 2.57 (m, 1H), 0.97 (s, 9H), 0.89 (s, 9H). ^{13}C NMR (101 MHz, $CDCl_3$) δ 142.9, 142.4, 126.4, 126.3, 124.6, 124.3, 77.9, 67.4, 57.8, 49.7, 35.6, 32.8, 27.6, 27.1, 23.0, 20.0. IR 2940, 2852, 1468, 1048, 744 cm^{-1}.

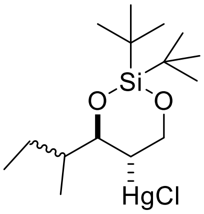

4-(*sec*-butyl)-2,2-di-*tert*-butyl-1,3,2-dioxasilinan-5-yl)mercury(II) chloride

Compound 23 (Mixture of diastereomers): purified using a gradient of 0 to 10% EtOAc:hexanes; (Colorless oil, 49%); ^{1}H NMR (400 MHz, Chloroform-*d*) δ 4.48 – 4.21 (m, 3H), 3.19 – 3.06 (m, 1H), 1.52 (s, 1H), 1.46 – 1.28 (m, 1H), 1.08 (d, *J* = 6.6 Hz, 2H), 1.04 (s, 9H), 1.00 (d, *J* = 1.3 Hz, 9H), 0.98 – 0.94 (m, 5H). ^{13}C NMR (101 MHz, Chloroform-*d*) δ 81.0, 78.2, 67.6, 58.2, 57.2, 44.7, 27.55, 27.52, 27.10, 27.08, 27.01, 23.0, 22.5, 20.05, 20.00, 16.4, 12.6, 12.3, 11.8. IR 2856, 1471, 1063, 824, 651 cm^{-1}.

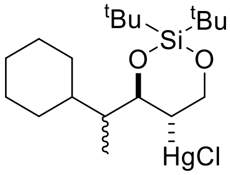

2,2-di-*tert*-butyl-4-(1-cyclohexylethyl)-1,3,2-dioxasilinan-5-yl)mercury(II) chloride

Compound 24 (Mixture of diastereomers): purified using a gradient of 0 to 10% EtOAc:hexanes; (Colorless thick oil, 13% brsm); ^{1}H NMR (400 MHz, Chloroform-*d*) δ 4.47 – 4.29 (m, 3H), 3.02 (ddd, *J* = 12.2, 11.0, 4.6 Hz, 1H), 1.99 – 1.87 (m, 1H), 1.80 – 1.65 (m, 4H), 1.64 – 1.50 (m, 3H), 1.37 – 1.08 (m, 7H), 1.04 (s, 9H), 1.00 (s, 9H). ^{13}C NMR (101 MHz, Chloroform-*d*) δ 80.0, 67.7, 55.3, 47.0, 39.2, 33.0, 30.1, 27.6, 27.4, 27.1, 27.0, 26.7, 26.4, 22.9, 19.9, 13.1. IR 2926, 1473, 1060, 751, 650 cm^{-1}.

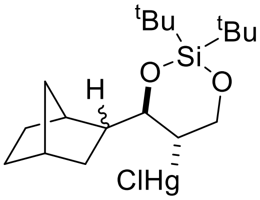

bicyclo[2.2.1]heptan-2-yl)-2,2-di-*tert*-butyl-1,3,2-dioxasilinan-5-yl)mercury(II) chloride

Compound 25 (Mixture of diastereomers): purified using a gradient of 0 to 10% EtOAc:hexanes; (White solid, 17%); ^{1}H NMR (400 MHz, Chloroform-*d*) (Mixture of diastereomers) δ 4.49 – 4.18 (m, 2.4H), 4.08-4.04 (m, 0.4H), 3.95-3.91 (m, 0.2H), 2.99 – 2.81 (m, 1H), 2.61 – 2.41 (m, 1H), 2.33-2.28 (m, 1H), 1.91 – 1.80 (m, 1H), 1.77 – 1.63 (m, 1H), 1.52 – 1.40 (m, 2H), 1.40 – 1.25 (m, 3H), 1.24 – 1.11 (m, 2H), 1.07 (d, *J* = 1.3 Hz, 6H), 1.06 (s, 3H), 1.03 (s, 2H), 1.02 (s, 4H), 1.01 (s, 3H). ^{13}C NMR (101 MHz, Chloroform-*d*) δ 80.1, 79.9, 79.1, 67.7, 67.4, 66.9, 58.3, 57.6, 57.1, 51.4, 49.2, 48.8, 40.8, 40.2, 39.0, 38.9, 38.2, 37.1, 37.0, 36.6, 36.5, 36.4, 35.7, 33.4, 30.4, 30.3, 30.1, 29.6, 28.6, 27.6, 27.55, 27.53, 27.3, 27.12, 27.10, 27.0, 23.5, 22.9, 22.88, 22.85, 19.8, 19.7. IR 2857, 1473, 1050, 825, 651 cm^{-1}

III. General Procedures for Allylic Rearrangement Reactions

Protocol A

A 10 mL round-bottom flask was charged with organomecurial starting material (0.1 to 0.2 mmol) and a stir bar. THF (mL quantity was 10 times the mmol of starting material) and 1M aqueous HCl (mL quantity was 10 times the mmol of starting material) were added sequentially. After thirty minutes of stirring at room temperature, the contents of the reaction flask were transferred to a separatory funnel with EtOAc and H_2O. The organic layer was collected, and the water layer was extracted with two additional portions of EtOAc. The organic layers were pooled, dried with $MgSO_4$, and concentrated under reduced pressure. The resulting residue was purified by chromatography on silica gel (see individual compounds for column conditions).

Protocol B

A 10 mL round-bottom flask was charged with organomercurial starting material (0.1 to 0.2 mmol) and a stir bar. DMF (mL quantity was 10 times the mmol of starting material) was then added followed by 2 equivalents of $NaBH_4$. After stirring for thirty minutes at room temperature, the reaction was quenched by slow addition of 2 mL of H_2O. The contents of the reaction flask were transferred to the separatory funnel with EtOAc and H_2O. The organic layer was collected, and the water layer was extracted with two additional portions of EtOAc. The organic layers were pooled, washed with an additional 20 mL of H_2O, dried with $MgSO_4$, and concentrated under reduced pressure. The resulting residue was purified by chromatography on silica gel (see individual compounds for column conditions).

IV. Characterization of Silanol Products

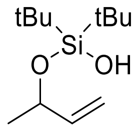

(but-3-en-2-yloxy)di-*tert*-butylsilanol

Compound 27: Synthesized using protocol A; Purified using a gradient of 0 to 5% EtOAc/hexanes followed by preparative thin layer chromatography (0.4% acetone/DCM); (colorless oil, 19% isolated yield); ^{1}H NMR (400 MHz, $CDCl_3$) δ 5.81 (ddd, *J* = 17.2, 10.4, 5.9 Hz, 1H), 5.11 (dt, *J* = 17.2, 1.6 Hz, 1H), 4.93 (ddd, *J* = 10.4, 1.8, 1.2 Hz, 1H), 4.56 – 4.38 (m, 1H), 1.26 – 1.10 (m, 3H), 1.02 – 0.81 (m, 18H). ^{13}C NMR (101 MHz, $CDCl_3$) δ 143.1, 112.6, 69.9, 27.4, 24.4, 20.4, 20.3. IR 3318, 1459, 1020, 623 cm^{-1}. HRMS calculated for $C_{12}H_{25}O_2Si^-$ 229.1629 Found 229.1590.

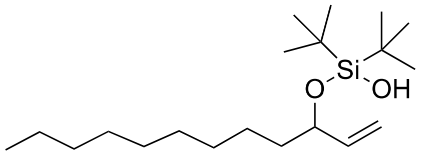

di-*tert*-butyl(dodec-1-en-3-yloxy)silanol

Compound 29: Synthesized using protocol A; Purified using a gradient of 5 to 40% DCM in hexanes; (Colorless oil, 34% isolated yield); ^{1}H NMR (400 MHz, $CDCl_3$) δ 5.76 (ddd, *J* = 17.2, 10.3, 6.8 Hz, 1H), 5.09 (dt, *J* = 17.1, 1.5 Hz, 1H), 4.98 (ddd, *J* = 10.3, 1.9, 1.0 Hz, 1H), 4.40 – 4.13 (m, 1H), 1.59 – 1.32 (m, 2H), 1.20 (d, *J* = 7.9 Hz, 14H), 0.95 (s, 9H), 0.93 (s, 9H), 0.87 – 0.69 (m, 3H). ^{13}C NMR (101 MHz, $CDCl_3$) δ 142.0, 113.9, 74.5, 38.2, 31.9, 29.7, 29.6, 29.5, 29.3, 27.5, 27.4, 24.8, 22.6, 20.5, 20.3, 14.1. IR 1536, 1165, 1018, 842 cm^{-1}. HRMS calculated for $C_{20}H_{41}O_2Si^-$ 341.2881 Found 341.2896.

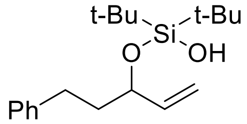

di-*tert*-butyl((5-phenylpent-1-en-3-yl)oxy)silanol

Compound 31: Synthesized using protocol A; Purified using a gradient of 0 to 20% EtOAc/hexanes; (colorless oil, 30% isolated yield); ^{1}H NMR (400 MHz, $CDCl_3$) δ 7.35 – 7.26 (m, 3H), 7.21 (m, 2H), 5.93 (ddd, *J* = 17.1, 10.4, 6.7 Hz, 1H), 5.26 (dt, *J* = 17.2, 1.5 Hz, 1H), 5.16 (ddd, *J* = 10.4, 1.8, 1.0 Hz, 1H), 4.50 (q, *J* = 6.2 Hz, 1H), 2.70 (tt, *J* = 9.7, 7.2 Hz, 2H), 2.01 – 1.81 (m, 2H), 1.07 (s, 9H), 1.04 (s, 9H). ^{13}C NMR (101 MHz, $CDCl_3$) δ 142.3, 141.5, 128.37, 128.35, 125.7, 114.5, 73.9, 39.8, 31.0, 28.0, 27.4, 20.5, 20.4. IR 1553, 1200, 1018, 853 cm^{-1}. HRMS calculated for $C_{19}H_{32}NaO_2Si^+$ 343.2064 Found 343.2084.

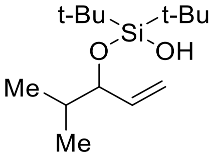

di-*tert*-butyl((4-methylpent-1-en-3-yl)oxy)silanol

Compound 33: Synthesized using protocol A; Purified using a gradient of 0 to 40% DCM in hexanes; (colorless oil, 54% yield); ^{1}H NMR (400 MHz, $CDCl_3$) δ 5.77 (ddd, *J* = 17.4, 10.4, 7.2 Hz, 1H), 5.19 – 4.93 (m, 2H), 4.12 (ddt, *J* = 7.1, 4.9, 1.1 Hz, 1H), 1.69 (qd, *J* = 6.8, 4.8 Hz, 1H), 0.96 (s, 9H), 0.93 (s, 9H), 0.82 (dd, *J* = 6.9, 1.7 Hz, 6H). ^{13}C NMR (101 MHz, $CDCl_3$) δ 139.8, 115.3, 81.5, 37.5, 27.6, 27.5, 20.7, 20.4, 18.0, 17.4. IR 1530, 1241, 1024, 829 cm^{-1}. HRMS calculated for $C_{14}H_{31}O_2Si^+$ 259.2088 Found 259.2049.

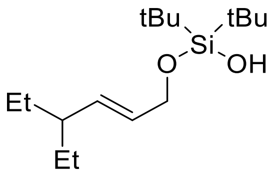

Compound 34: Synthesized using protocol A; Purified using a gradient of 0 to 40% DCM in hexanes; (colorless oil, 15% yield); ^{1}H NMR (400 MHz, $CDCl_3$) δ 5.61 – 5.49 (m, 1H), 5.42 (ddt, *J* = 15.3, 8.6, 1.4 Hz, 1H), 4.33 (dd, *J* = 5.4, 1.4 Hz, 2H), 1.93 (s, 1H), 1.80 (dtd, *J* = 13.5, 8.7, 5.0 Hz, 1H), 1.51 – 1.36 (m, 2H), 1.33 – 1.17 (m, 2H), 1.05 (s, 18H), 0.86 (t, *J* = 7.4 Hz, 6H). ^{13}C NMR (101 MHz, $CDCl_3$) δ 135.4, 129.4, 64.2, 45.7, 27.5, 27.4, 20.4, 11.6. IR 2857, 1468, 1206, 805 cm^{-1}. HRMS calculated for $C_{16}H_{34}NaO_2Si^+$ 309.2220 Found 309.2209.

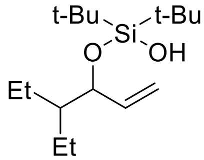

di-*tert*-butyl((4-ethylhex-1-en-3-yl)oxy)silanol

Compound 35: synthesized using protocol A; purified using a gradient of 0 to 40% DCM in hexanes; (colorless oil, 58% yield); ^{1}H NMR (400 MHz, $CDCl_3$) δ 5.78 (ddd, *J* = 17.4, 10.4, 7.2 Hz, 1H), 5.19 – 4.96 (m, 2H), 4.44 – 4.25 (m, 1H), 1.59 – 1.48 (m, 1H), 1.35 – 1.18 (m, 3H), 1.11 – 0.99 (m, 1H), 0.94 (d, *J* = 11.8 Hz, 18H), 0.84 (td, *J* = 7.2, 5.2 Hz, 6H). ^{13}C NMR (101 MHz, $CDCl_3$) δ 139.9, 114.9, 76.1, 48.6, 27.59, 27.50, 22.2, 21.6, 20.6, 20.4, 12.27, 12.19. IR 3373, 2942, 1471, 1030, 820 cm^{-1}. HRMS calculated for $C_{16}H_{34}NaO_2Si^+$ 309.2220 Found 309.2222.

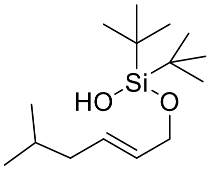

(*E*)-di-*tert*-butyl((5-methylhex-2-en-1-yl)oxy)silanol

Compound 36: synthesized using protocol A; purified using a gradient of 0 to 40% DCM in hexanes; (Colorless oil, 30%); ^{1}H NMR (400 MHz, Chloroform-*d*) δ 5.72 – 5.61 (m, 1H), 5.61 – 5.51 (m, 1H), 4.30 (dd, *J* = 5.2, 1.2 Hz, 2H), 1.92 (td, *J* = 6.8, 1.1 Hz, 2H), 1.62 (tq, *J* = 13.1, 6.6 Hz, 2H), 1.02 (s, 18H), 0.88 (d, *J* = 6.6 Hz, 6H); ^{13}C NMR (101 MHz, Chloroform-*d*) δ 130.4, 130.0, 64.1, 41.5, 28.3, 27.4, 22.2, 20.4; IR 2933, 1472, 1099, 825 cm^{-1}.; HRMS calculated for $C_{15}H_{31}O_2Si^-$ 271.2093 Found 271.2156.

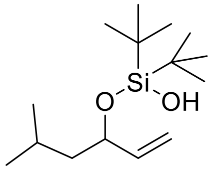

di-*tert*-butyl((5-methylhex-1-en-3-yl)oxy)silanol

Compound 37: synthesized using protocol A; purified using a gradient of 0 to 40% DCM in hexanes; (Colorless oil, 56%); ^{1}H NMR (400 MHz, Chloroform-*d*) δ 5.84 (ddd, *J* = 17.4, 10.3, 7.2 Hz, 1H), 5.24 – 5.02 (m, 2H), 4.42 (td, *J* = 7.2, 6.2 Hz, 1H), 1.71 (d, *J* = 3.5 Hz, 1H), 1.69 – 1.63 (m, 1H), 1.49 (ddd, *J* = 13.8, 7.6, 6.4 Hz, 1H), 1.35 (dt, *J* = 13.4, 6.7 Hz, 1H), 1.04 (s, 9H), 1.00 (s, 9H), 0.93 (d, *J* = 6.8 Hz, 3H), 0.91 (d, *J* = 6.6 Hz, 3H); ^{13}C NMR (101 MHz, Chloroform-*d*) δ 142.4, 113.9, 73.2, 47.5, 27.5, 27.4, 24.2, 23.1, 22.6, 20.5, 20.2; IR 2858, 1470, 1064, 826, 644 cm^{-1}.; HRMS calculated for $C_{15}H_{31}O_2Si^-$ 271.2093 Found 271.2154.

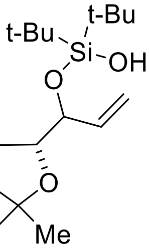

di-*tert*-butyl((1-((*R*)-2,2-dimethyl-1,3-dioxolan-4-yl)allyl)oxy)silanol

Compound 40: synthesized using protocol B; single diastereomer, relative stereochemistry unassigned; purified using a gradient of 0 to 20% EtOAc:hexanes; (colorless oil, 50% yield); ^{1}H NMR (400 MHz, $CDCl_3$) δ 5.75 (ddd, *J* = 17.1, 10.5, 4.8 Hz, 1H), 5.37 (dt, *J* = 17.1, 1.8 Hz, 1H), 5.15 (dt, *J* = 10.5, 1.8 Hz, 1H), 4.71 (ddt, *J* = 5.0, 3.5, 1.8 Hz, 1H), 4.06 (ddd, *J* = 7.6, 6.8, 3.3 Hz, 1H), 3.87 (t, *J* = 7.7 Hz, 1H), 3.79 (dd, *J* = 7.8, 6.8 Hz, 1H), 1.39 (s, 3H), 1.31 – 1.24 (m, 3H), 0.99 (s, 9H), 0.95 (s, 9H). ^{13}C NMR (101 MHz, $CDCl_3$) δ 136.5, 116.3, 109.1, 78.2, 71.6, 63.8, 27.66, 27.60, 26.1, 24.7, 21.0, 20.4. IR 2890, 1477, 1074, 841 cm^{-1}. HRMS calculated for $C_{16}H_{32}NaO_4Si^+$ 339.1962 Found 339.1947.

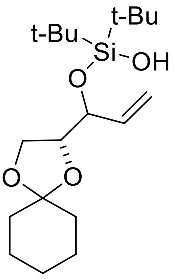

((1-((*R*)-1,4-dioxaspiro[4.5]decan-2-yl)allyl)oxy)di-*tert*-butylsilanol

Compound 41: Synthesized using protocol B; single diastereomer, relative stereochemistry unassigned; purified using a gradient of 0 to 20% EtOAc in Hexanes; (colorless oil, 51% yield); ^{1}H NMR (400 MHz, $CDCl_3$) δ 5.74 (ddd, *J* = 17.0, 10.5, 4.6 Hz, 1H), 5.36 (dt, *J* = 17.1, 1.8 Hz, 1H), 5.14 (dt, *J* = 10.5, 1.8 Hz, 1H), 4.75 (ddt, *J* = 5.0, 3.4, 1.8 Hz, 1H), 4.06 (ddd, *J* = 7.8, 6.8,

3.1 Hz, 1H), 3.84 (t, J = 7.7 Hz, 1H), 3.77 (dd, J = 7.8, 6.8 Hz, 1H), 1.76 – 1.43 (m, 8H), 1.40 – 1.24 (m, 2H), 0.98 (s, 9H), 0.96 (s, 9H). ^{13}C NMR (101 MHz, $CDCl_3$) δ 136.5, 116.2, 109.7, 77.7, 71.5, 63.3, 35.7, 34.0, 27.69, 27.62, 25.1, 24.0, 23.6, 21.1, 20.4. IR 2850, 1154, 1008, 821 cm^{-1}. HRMS calculated for $C_{19}H_{35}O_4Si^-$ 355.2310 Found 355.2305.

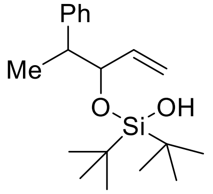

di-*tert*-butyl((4-phenylpent-1-en-3-yl)oxy)silanol

Compound 42: synthesized using protocol A; single diastereomer, relative stereochemistry unassigned; purified using a gradient of 0 to 50% DCM:hexanes; (colorless oil, 75% yield); ^{1}H NMR (400 MHz, $CDCl_3$) δ 7.18 (ttd, J = 17.9, 7.2, 1.3 Hz, 5H), 5.72 (ddd, J = 17.2, 10.4, 6.8 Hz, 1H), 5.16 – 5.06 (m, 2H), 4.39 (tt, J = 6.8, 1.1 Hz, 1H), 2.80 (p, J = 7.0 Hz, 1H), 1.17 (d, J = 7.1 Hz, 3H), 0.88 (s, 9H), 0.83 (s, 9H). ^{13}C NMR (101 MHz, $CDCl_3$) δ 144.0, 139.6, 128.4, 128.0, 126.3, 115.7, 79.4, 46.8, 27.51, 27.49, 20.6, 20.4, 16.0. IR 2895, 1464, 1072, 821 cm^{-1}. HRMS calculated for $C_{19}H_{32}NaO_2Si^+$ 343.2064 Found 343.2042.

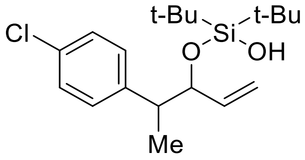

di-*tert*-butyl((4-(4-chlorophenyl)pent-1-en-3-yl)oxy)silanol

Compound 43: synthesized using protocol A; single diastereomer, relative stereochemistry unassigned; purified using a gradient of 0 to 20% EtOAc:hexanes; (colorless oil, 90% yield); ^{1}H NMR (400 MHz, $CDCl_3$) δ 7.23 – 7.15 (m, 2H), 7.14 – 7.03 (m, 2H), 5.66 (ddd, J = 17.4, 10.4, 7.1 Hz, 1H), 5.12 – 4.99 (m, 2H), 4.35 (ddt, J = 7.1, 6.1, 1.0 Hz, 1H), 2.80 (p, J = 7.0 Hz, 1H), 1.17 (t, J = 6.7 Hz, 3H), 0.88 (s, 9H), 0.85 (s, 9H). ^{13}C NMR (101 MHz, $CDCl_3$) δ 142.1, 139.4, 131.9, 129.8, 128.0, 116.0, 79.2, 46.1, 27.4, 20.6, 20.3, 15.8. IR 2943, 2866, 1489, 829 cm^{-1}. HRMS calculated for $C_{19}H_{32}ClO_2Si^+$ 355.1855 Found 355.1821.

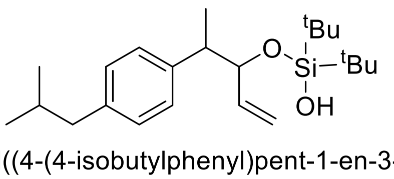

(±) di-*tert*-butyl((4-(4-isobutylphenyl)pent-1-en-3-yl)oxy)silanol

Compound 44: synthesized using protocol A; single diastereomer, relative stereochemistry unassigned; (Colorless oil, 91% yield); ^{1}H NMR (400 MHz, Chloroform-*d*) δ 7.15 (d, J = 8.2 Hz, 2H), 7.08 (d, J = 8.0 Hz, 2H), 5.81 (ddd, J = 17.3, 10.4, 6.9 Hz, 1H), 5.24 – 5.09 (m, 2H), 4.50 – 4.39 (m, 1H), 2.84 (p, J = 7.1 Hz, 1H), 2.45 (d, J = 7.1 Hz, 2H), 1.84 (dt, J = 13.5, 6.8 Hz, 1H), 1.22 (d, J = 7.1 Hz, 3H), 0.96 (s, 9H), 0.90 (d, J = 6.8 Hz, 6H), 0.89 (s, 9H); ^{13}C NMR (101 MHz, Chloroform-*d*) δ 141.2, 139.9, 139.6, 128.7, 128.1, 115.6, 79.5, 46.3, 45.0, 30.2, 27.4, 22.35, 22.33,

20.5, 20.4, 16.1; IR 2932, 1275, 764, 826, 645 cm^{-1}.; HRMS calculated for $C_{23}H_{39}O_2Si^-$ 375.2719 found 375.2737.

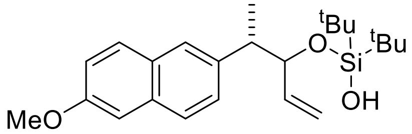

(±) di-*tert*-butyl((4-(6-methoxynaphthalen-2-yl)pent-1-en-3-yl)oxy)silanol

Compound 45: synthesized using protocol A; Single diastereomer, absolute stereochemistry unassigned; purified using a gradient of 0 to 40% DCM in hexanes; (Colorless oil, 74% yield); ^{1}H NMR (400 MHz, Chloroform-*d*) δ 7.69 (d, *J* = 8.4 Hz, 2H), 7.61 (d, *J* = 1.7 Hz, 1H), 7.39 (dd, *J* = 8.5, 1.8 Hz, 1H), 7.15 (d, *J* = 2.5 Hz, 1H), 7.12 (s, 1H), 5.82 (ddd, *J* = 17.2, 10.4, 6.8 Hz, 1H), 5.24 – 5.09 (m, 2H), 4.65 – 4.53 (m, 1H), 3.93 (s, 3H), 3.05 (p, *J* = 7.0 Hz, 1H), 1.35 (d, *J* = 7.1 Hz, 3H), 0.98 (s, 9H), 0.91 (s, 9H); ^{13}C NMR (101 MHz, Chloroform-*d*) δ 157.2, 139.5, 138.9, 133.3, 129.0, 128.8, 127.4, 126.8, 126.3, 118.6, 115.6, 105.5, 79.2, 55.2, 46.6, 27.48, 27.44, 20.5, 20.4, 15.9; IR 2933, 1261, 750, 650 cm^{-1}.; HRMS calculated for $C_{24}H_{35}O_3Si^-$ 399.2355 found 399.2313.

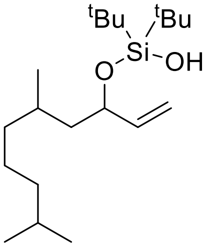

(±) di-*tert*-butyl(-5,9-dimethyldec-1-en-3-yl)oxy)silanol

Compound 46 (*dr = 1:1*): synthesized using protocol A; purified using a gradient of 0 to 40% DCM in hexanes.; (Colorless oil, 66% yield); ^{1}H NMR (400 MHz, Chloroform-*d*) δ 5.84 (dddd, *J* = 18.6, 17.5, 10.3, 7.3 Hz, 1H), 5.23 – 5.12 (m, 1H), 5.06 (ddd, *J* = 10.8, 5.2, 1.6 Hz, 1H), 4.44 (ddd, *J* = 13.1, 7.2, 5.7 Hz, 1H), 1.67 – 1.37 (m, 4H), 1.30 (dtt, *J* = 11.4, 4.4, 2.9 Hz, 2H), 1.25 – 1.19 (m, 1H), 1.19 – 1.08 (m, 3H), 1.04 (d, *J* = 0.9 Hz, 9H), 1.00 (d, *J* = 0.9 Hz, 9H), 0.93 – 0.89 (m, 3H), 0.88 (s, 3H), 0.86 (s, 3H); ^{13}C NMR (101 MHz, Chloroform-*d*) δ 142.7, 142.2, 114.1, 113.8, 73.2, 73.0, 45.9, 45.7, 39.29, 39.20, 37.8, 37.3, 29.0, 28.7, 27.9, 27.58, 27.53, 27.48, 27.45, 24.66, 24.46, 22.68, 22.66, 22.60, 20.1, 19.8; IR 2929, 1470, 1070, 826, 644 cm^{-1}.; HRMS calculated for $C_{20}H_{41}O_2Si^-$ 341.2876 found 341.2909.

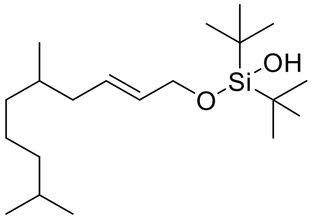

(*E*)-di-*tert*-butyl((5,9-dimethyldec-2-en-1-yl)oxy)silanol

Compound 46 isomer: synthesized using protocol A; purified using a gradient of 0 to 40% DCM in hexanes ; (Colorless oil, 22% yield); ^{1}H NMR (400 MHz, Chloroform-*d*) δ 5.75 – 5.50 (m, 2H), 4.31 (dd, *J* = 5.2, 1.2 Hz, 2H), 2.10 – 1.96 (m, 1H), 1.88 (dtd, *J* = 13.9, 7.2, 1.1 Hz, 1H), 1.51 (dt, *J* = 13.2, 6.6 Hz, 2H), 1.33 – 1.24 (m, 3H), 1.18 – 1.08 (m, 3H), 1.03 (s, 18H), 0.93 – 0.79 (m, 9H); ^{13}C NMR (101 MHz, Chloroform-*d*) δ 130.4, 129.9, 64.1, 39.6, 39.2, 36.7, 33.0, 27.9, 27.4, 24.7, 22.6, 22.5, 20.4, 19.5; IR 2930, 1469, 1104, 826 cm^{-1}.; HRMS calculated for $C_{20}H_{41}O_2Si^-$ 341.2876 found 341.2876.

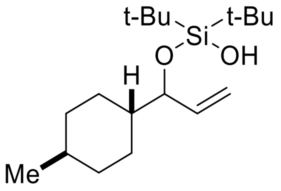

di-*tert*-butyl((4-methylcyclohexyl)allyl)oxy)silanol

Compound 47: synthesized using Protocol A; purified using a gradient of 0 to 50% DCM in hexanes; (colorless oil, 70% yield); ^{1}H NMR (400 MHz, $CDCl_3$) δ 5.78 (ddd, *J* = 17.5, 10.4, 7.3 Hz, 1H), 5.15 – 4.94 (m, 2H), 4.10 (ddd, *J* = 7.3, 5.1, 1.2 Hz, 1H), 1.81 – 1.57 (m, 5H), 1.29 (tdt, *J* = 11.9, 5.1, 3.2 Hz, 1H), 0.94 (d, *J* = 13.4 Hz, 20H), 0.88 – 0.73 (m, 5H). ^{13}C NMR (101 MHz, $CDCl_3$) δ 140.6, 114.9, 79.0, 44.4, 35.08, 35.06, 32.9, 28.3, 28.1, 27.6, 27.5, 22.6, 20.7, 20.4. IR 2864, 1460, 1066, 832 cm^{-1}. HRMS calculated for $C_{18}H_{35}O_2Si^-$ 311.2412 Found 311.2430.

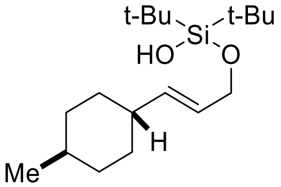

di-*tert*-butyl(((*E*)-3-(4-methylcyclohexyl)allyl)oxy)silanol

Compound 47 isomer: synthesized using Protocol A; purified using a gradient of 0 to 50% DCM:hexanes; (colorless oil, 30% yield); ^{1}H NMR (400 MHz, $CDCl_3$) δ 5.65 (ddt, *J* = 15.5, 6.3, 1.2 Hz, 1H), 5.55 (dtd, *J* = 15.5, 5.2, 1.0 Hz, 1H), 4.31 (dt, *J* = 5.1, 1.1 Hz, 2H), 1.96 – 1.84 (m, 1H), 1.81 – 1.66 (m, 4H), 1.38 – 1.25 (m, 1H), 1.05 (m, 22H), 0.90 (d, *J* = 6.5 Hz, 3H). ^{13}C NMR (101 MHz, $CDCl_3$) δ 137.4, 126.8, 64.3, 40.0, 34.9, 32.8, 32.3, 27.4, 22.7, 20.4. IR 2889, 1484, 1030, 866 cm^{-1}. HRMS calculated for $C_{18}H_{35}O_2Si^-$ 311.2412 found 311.2466.

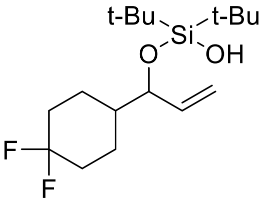

di-*tert*-butyl((1-(4,4-difluorocyclohexyl)allyl)oxy)silanol

Compound 48: synthesized using Protocol A; purified using a gradient of 0 to 50% DCM:hexanes; (colorless oil, 77% yield); ^{1}H NMR (400 MHz, $CDCl_3$) δ 5.76 (ddd, *J* = 17.5, 10.4, 7.4 Hz, 1H), 5.17 – 5.01 (m, 2H), 4.20 – 4.11 (m, 1H), 2.11 – 1.98 (m, 2H), 1.84 – 1.69 (m, 3H), 1.69 – 1.49 (m, 2H), 1.49 – 1.37 (m, 1H), 1.37 – 1.12 (m, 1H), 0.95 (s, 9H), 0.93 (s, 9H). ^{13}C NMR (101 MHz, $CDCl_3$) δ 139.7, 123.7 (t, *J* = 240 Hz), 115.9, 77.8 (d, *J* = 2.5 Hz), 42.8 (d, *J* = 1.6 Hz), 33.5 (dd, *J* = 25.7, 22.4 Hz), 27.6, 27.5, 24.4 (dd, *J* = 35.8, 10.0 Hz), 20.7, 20.4. IR 2849, 1365, 1094, 835 cm^{-1}. HRMS calculated for $C_{17}H_{31}F_2O_2Si^-$ 333.2067 Found 333.2104.

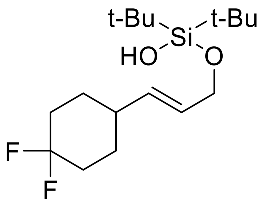

(*E*)-di-*tert*-butyl((3-(4,4-difluorocyclohexyl)allyl)oxy)silanol

Compound 48 isomer: synthesized using protocol A; colorless oil; ^{1}H NMR (400 MHz, $CDCl_3$) δ 5.87 – 5.46 (m, 2H), 4.34 (ddd, *J* = 3.6, 2.6, 1.3 Hz, 2H), 2.15 – 2.05 (m, 3H), 1.90 – 1.76 (m, 2H), 1.71 (dd, *J* = 13.3, 4.5 Hz, 2H), 1.48 (dt, *J* = 17.4, 12.4 Hz, 2H), 1.05 (s, 18H). ^{13}C NMR (101 MHz, $CDCl_3$) δ 133.7, 128.5, 123.4 (t, *J* = 230 Hz), 63.9, 37.9, 33.1 (dd, *J* = 25.3, 22.8 Hz), 28.7 (d, *J* = 9.3 Hz), 27.4, 20.4. IR 2878, 1447, 1100, 829 cm^{-1}. HRMS calculated for $C_{17}H_{33}F_2O_2Si^+$ 335.2212 Found 335.2166.

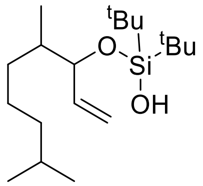

(±)di-*tert*-butyl((4,8-dimethylnon-1-en-3-yl)oxy)silanol

Compound 49 (*dr* = ~2:1): synthesized using protocol A; purified using a gradient of 0 to 40% DCM:hexanes; (Colorless oil, 48%); ^{1}H NMR (400 MHz, Chloroform-*d*) δ 5.77 (dddd, *J* = 17.3, 11.9, 10.4, 7.1 Hz, 1H), 5.15 – 5.00 (m, 2H), 4.26 – 4.16 (m, 1H), 1.74 (s, 1H), 1.58 (dddp, *J* = 11.1, 6.6, 4.3, 2.1 Hz, 1H), 1.51 – 1.40 (m, 2H), 1.34 – 1.22 (m, 2H), 1.17 – 1.03 (m, 3H), 0.96 (s, 9H), 0.93 (s, 9H), 0.80 (d, *J* = 6.5 Hz, 9H); ^{13}C NMR (101 MHz, Chloroform-*d*) δ 140.2, 139.3, 115.3, 114.9, 78.6, 78.1, 39.9, 39.7, 39.30, 39.27, 32.9, 31.9, 27.9, 27.61, 27.57, 27.50, 27.48, 25.22, 25.09, 22.72, 22.67, 22.59, 22.53, 20.73, 20.66, 20.47, 20.44, 14.9, 14.2; IR 2858, 1470, 1075, 826, 644 cm^{-1}.; HRMS calculated for $C_{19}H_{39}O_2Si^-$ 327.2719 found 327.2708.

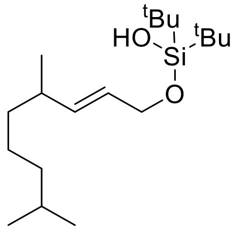

(*E*)-di-*tert*-butyl((4,8-dimethylnon-2-en-1-yl)oxy)silanol

Compound 49 isomer: synthesized using protocol A; purified using a gradient of 0 to 40% DCM:hexanes; (Colorless oil, 19%); ^{1}H NMR (400 MHz, Chloroform-*d*) δ 5.59 – 5.47 (m, 2H), 4.34 – 4.27 (m, 2H), 2.18 – 2.07 (m, 1H), 1.52 (dt, *J* = 13.3, 6.6 Hz, 1H), 1.28 – 1.23 (m, 5H), 1.17 – 1.12 (m, 2H), 1.03 (s, 18H), 0.98 (d, *J* = 6.7 Hz, 3H), 0.86 (dd, *J* = 6.6, 0.8 Hz, 6H); ^{13}C NMR (101 MHz, Chloroform-*d*) δ 137.3, 127.4, 64.2, 39.1, 37.1, 36.2, 27.9, 27.4, 25.0, 22.65, 22.62, 20.5; IR 2930, 1469, 1103, 826 cm^{-1}.; HRMS calculated for $C_{19}H_{39}O_2Si^-$ 327.2719 found 327.2708.

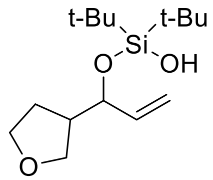

di-*tert*-butyl((1-(tetrahydrofuran-3-yl)allyl)oxy)silanol

Compound 50: synthesized using protocol A; single diastereomer, relative stereochemistry unassigned; purified using a gradient of 0 to 30% EtOAc:hexanes; (light yellow semi-solid, 80% yield); ^{1}H NMR (400 MHz, $CDCl_3$) δ 5.78 (ddd, *J* = 17.4, 10.3, 7.3 Hz, 1H), 5.23 – 4.99 (m, 2H), 4.23 (ddt, *J* = 8.3, 7.3, 1.0 Hz, 1H), 3.82 – 3.74 (m, 2H), 3.74 – 3.61 (m, 2H), 2.38 – 2.25 (m, 1H), 1.79 (dddd, *J* = 12.4, 8.6, 7.4, 4.8 Hz, 1H), 1.62 (dq, *J* = 12.6, 7.7 Hz, 1H), 1.01 – 0.87 (m, 18H). ^{13}C NMR (101 MHz, $CDCl_3$) δ 140.3, 115.6, 76.6, 70.3, 68.2, 46.2, 28.3, 27.6, 27.4, 20.7, 20.4. IR 2849, 1471, 1065, 829 cm^{-1}. HRMS calculated for $C_{15}H_{29}O_3Si^-$ 285.1891 Found 285.1921.

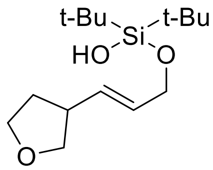

(*E*)-di-*tert*-butyl((3-(tetrahydrofuran-3-yl)allyl)oxy)silanol

Compound 50 isomer: synthesized using protocol A; purified using a gradient of 0 to 30% EtOAc:hexanes; (white semi-solid, 20% yield); ^{1}H NMR (400 MHz, $CDCl_3$) δ 5.70 – 5.53 (m, 2H), 4.29 (d, *J* = 3.5 Hz, 2H), 3.95 – 3.82 (m, 2H), 3.78 (qd, *J* = 7.7, 1.9 Hz, 1H), 3.41 (t, *J* = 8.0 Hz, 1H), 2.92 – 2.76 (m, 1H), 2.07 (dtd, *J* = 12.2, 7.6, 4.5 Hz, 1H), 1.69 (dq, *J* = 12.2, 8.0 Hz, 1H), 1.01 (s, 18H). ^{13}C NMR (101 MHz, $CDCl_3$) δ 130.6, 130.3, 130.2, 129.9, 72.8, 68.1, 63.8, 63.6, 42.2, 33.1, 33.0, 27.8, 27.4, 21.2, 20.5. IR 2855, 1465, 1068, 829 cm^{-1}. HRMS calculated for $C_{15}H_{29}O_3Si^-$ 285.1891 Found 285.1870.

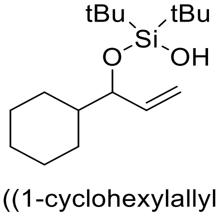

di-*tert*-butyl((1-cyclohexylallyl)oxy)silanol

Compound 51: synthesized using protocol A; Purified using a gradient of 0 to 0.1% acetone/DCM; (colorless oil, 67% isolated yield); ^{1}H NMR (400 MHz, $CDCl_3$) δ 5.78 (ddd, *J* = 17.5, 10.4, 7.3 Hz, 1H), 5.18 – 4.84 (m, 2H), 4.19 – 3.94 (m, 1H), 1.78 – 1.52 (m, 6H), 1.34 (tdt, *J* = 11.7, 5.2, 3.1 Hz, 1H), 1.22 – 1.00 (m, 4H), 0.96 (s, 9H), 0.93 (s, 9H). ^{13}C NMR (101 MHz, $CDCl_3$) δ 140.6, 115.0, 79.1, 44.7, 28.5, 28.2, 27.6, 27.5, 26.7, 26.36, 26.33, 20.7, 20.4. IR 1512, 1159, 853, 494 cm^{-1}. HRMS calculated for $C_{17}H_{33}O_2Si^-$ 297.2255 Found 297.2245.

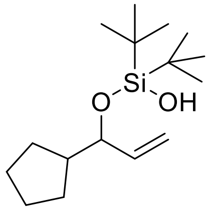

di-*tert*-butyl((1-cyclopentylallyl)oxy)silanol

Compound 52: synthesized using protocol A; purified using a gradient of 0-40% DCM:hexanes.; (Colorless oil, 57%); ^{1}H NMR (400 MHz, Chloroform-*d*) δ 5.88 (ddd, *J* = 17.5, 10.3, 7.5 Hz, 1H), 5.24 – 5.04 (m, 2H), 4.31 – 4.20 (m, 1H), 2.06 – 1.93 (m, 1H), 1.80 – 1.69 (m, 2H), 1.66 – 1.38 (m, 6H), 1.37 – 1.29 (m, 1H), 1.04 (s, 9H), 1.01 (s, 9H); ^{13}C NMR (101 MHz, Chloroform-*d*) δ 141.4, 114.6, 78.3, 46.7, 28.3, 28.2, 27.6, 27.5, 25.6, 25.5, 20.8, 20.3; IR 2858, 1472, 1066, 825, 644 cm^{-1}.; HRMS calculated for $C_{16}H_{31}O_2Si^-$ 283.2093 found 283.2114.

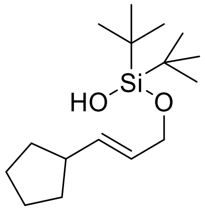

(*E*)-di-*tert*-butyl((3-cyclopentylallyl)oxy)silanol

Compound 52 isomer: synthesized using protocol A; purified using a gradient of 0-40% DCM:hexanes.; (Colorless oil, 10%); ^{1}H NMR (400 MHz, Chloroform-*d*) δ 5.72 – 5.60 (m, 1H), 5.60 – 5.51 (m, 1H), 4.30 (dt, *J* = 5.3, 1.0 Hz, 2H), 2.44 (q, *J* = 7.9 Hz, 1H), 1.83 – 1.74 (m, 2H), 1.69 – 1.60 (m, 3H), 1.59 – 1.53 (m, 2H), 1.40 – 1.23 (m, 2H), 1.03 (s, 18H); ^{13}C NMR (101 MHz, Chloroform-*d*) δ 135.9, 127.4, 64.2, 42.8, 32.9, 27.4, 25.0, 20.4. IR 2934, 1472, 1064, 825 cm^{-1}.; HRMS calculated for $C_{16}H_{31}O_2Si^-$ 283.2093 found 283.2163.

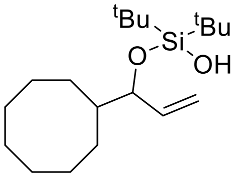

di-*tert*-butyl((1-cyclooctylallyl)oxy)silanol

Compound 53: synthesized using protocol A; purified using a gradient of 0-40% DCM:hexanes; (Colorless oil, 68%);^{1}H NMR (400 MHz, Chloroform-*d*) δ 5.84 (ddd, *J* = 17.3, 10.4, 6.9 Hz, 1H), 5.23 – 5.06 (m, 2H), 4.22 (ddt, *J* = 6.9, 4.8, 1.1 Hz, 1H), 1.79 – 1.64 (m, 5H), 1.63 – 1.53 (m, 4H), 1.53 – 1.38 (m, 5H), 1.31-1.18 (m, 2H), 1.04 (s, 9H), 1.01 (s, 9H); ^{13}C NMR (101 MHz, Chloroform-*d*) δ 139.9, 115.1, 79.6, 44.2, 29.3, 28.1, 27.6, 27.5, 26.77, 26.68, 26.60, 26.44, 26.14, 20.6, 20.4; IR 2928, 1472, 1246, 826, 738, 644 cm^{-1}.; HRMS calculated for $C_{19}H_{39}O_2Si^+$ 327.2719 found 327.2703.

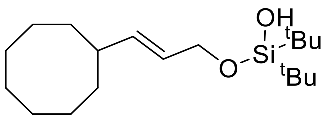

(*E*)-di-*tert*-butyl((3-cyclooctylallyl)oxy)silanol

Compound 53 isomer: synthesized using protocol A; purified using a gradient of 0-40% DCM:hexanes.; (Colorless oil, 16%); ^{1}H NMR (400 MHz, Chloroform-*d*) δ 5.59 (ddt, *J* = 15.4, 7.1, 1.4 Hz, 1H), 5.42 (dtd, *J* = 15.4, 5.5, 1.2 Hz, 1H), 4.22 (dt, *J* = 5.5, 1.1 Hz, 2H), 2.21 – 2.08 (m, 1H), 1.60 (m, 4H), 1.54 – 1.29 (m, 10H), 0.95 (s, 18H); ^{13}C NMR (101 MHz, Chloroform-*d*) δ 138.2, 126.3, 64.4, 40.3, 31.7, 29.7, 27.4, 25.9, 25.0, 20.4; IR 2922, 1471, 1099, 826 cm^{-1}.; HRMS calculated for $C_{19}H_{39}O_2Si^+$ 327.2719 found 327.2706.

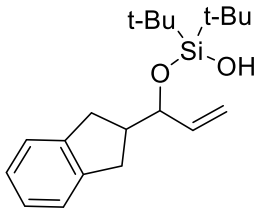

di-*tert*-butyl((1-(2,3-dihydro-1*H*-inden-2-yl)allyl)oxy)silanol

Compound 54: synthesized using protocol A; purified using a gradient of 0 to 50% DCM:hexanes; (colorless oil, 71% yield); ^{1}H NMR (400 MHz, $CDCl_3$) δ 7.16 – 6.88 (m, 4H), 5.80 (ddd, *J* = 17.4, 10.3, 7.3 Hz, 1H), 5.26 – 5.13 (m, 1H), 5.05 (ddd, *J* = 10.3, 1.8, 0.9 Hz, 1H), 4.37 (ddt, *J* = 7.3, 6.3, 1.0 Hz, 1H), 3.03 – 2.70 (m, 4H), 2.65 – 2.46 (m, 1H), 0.95 (s, 9H), 0.93 (s, 9H). ^{13}C NMR (101 MHz, $CDCl_3$) δ 143.17, 143.13, 140.4, 126.1, 126.0, 124.4, 124.3, 115.4, 77.7, 46.1, 35.1, 34.9, 27.6, 27.5, 20.7, 20.4. IR 2949, 2860, 1477, 1086 cm^{-1}. HRMS calculated for $C_{20}H_{31}O_2Si^-$ 331.2099 Found 331.2130.

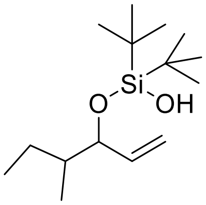

(±)di-*tert*-butyl(-4-methylhex-1-en-3-yl)oxy)silanol

Compound 55 (dr = ~1.5:1): synthesized using protocol A; purified using a gradient of 0-40% DCM:hexanes; (Colorless oil, 51% yield); ^{1}H NMR (400 MHz, Chloroform-*d*) δ 5.85 (dddd, *J* = 17.4, 12.6, 10.4, 7.1 Hz, 1H), 5.24 – 5.08 (m, 2H), 4.30 (dddt, *J* = 8.9, 6.7, 4.2, 1.1 Hz, 1H), 1.69 – 1.52 (m, 1H), 1.52 – 1.39 (m, 1H), 1.20 – 1.06 (m, 2H), 1.04 (s, 9H), 1.01 (s, 9H), 0.94 – 0.86 (m, 6H); ^{13}C NMR (101 MHz, Chloroform-*d*) δ 140.2, 139.3, 115.3, 114.9, 78.5, 77.9, 41.8, 41.6, 27.60, 27.57, 27.50, 27.48, 25.3, 24.5, 20.7, 20.6, 20.48, 20.44, 14.4, 13.8, 12.0, 11.9; IR 2963, 1472, 1072, 826, 644 cm^{-1}.; HRMS calculated for $C_{15}H_{31}O_2Si^-$ 271.2093 found 271.2119.

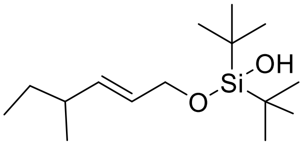

(*E*)-di-*tert*-butyl((4-methylhex-2-en-1-yl)oxy)silanol

Compound 55 isomer: synthesized using protocol A; purified using a gradient of 0-40% DCM:hexanes; (Colorless oil, 25% yield); ^{1}H NMR (400 MHz, Chloroform-*d*) δ 5.61 – 5.48 (m, 2H), 4.35 – 4.25 (m, 2H), 2.10 – 1.99 (m, 1H), 1.32 (p, *J* = 7.3 Hz, 2H), 1.03 (s, 18H), 0.98 (d, *J* = 6.7 Hz, 3H), 0.87 (t, *J* = 7.4 Hz, 3H); ^{13}C NMR (101 MHz, Chloroform-*d*) δ 137.0, 127.6, 64.2, 37.8, 29.6, 27.4, 20.4, 19.9, 11.6; IR 2962, 1472, 1089, 825 cm^{-1}.; HRMS calculated for $C_{15}H_{31}O_2Si^-$ 271.2093 found 271.2102.

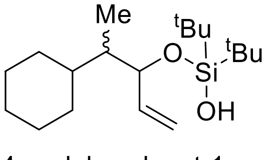

(±)di-*tert*-butyl((4-cyclohexylpent-1-en-3-yl)oxy)silanol

Compound 56 (*dr* = 2.5:1): synthesized using protocol A; purified using a gradient of 0-40% DCM:hexanes; (Colorless oil, 34% yield); ^{1}H NMR (400 MHz, Chloroform-*d*) δ 5.79 (dddd, *J* = 33.6, 17.4, 10.4, 7.3 Hz, 1H), 5.17 – 5.00 (m, 2H), 4.44 – 4.25 (m, 1H), 1.71 – 1.54 (m, 5H), 1.38 (qd, *J* = 7.0, 5.5 Hz, 1H), 1.29 – 1.23 (m, 1H), 1.16 – 0.99 (m, 5H), 0.96 (d, *J* = 1.8 Hz, 9H), 0.93 (s, 9H), 0.82 (d, *J* = 6.9 Hz, 2H), 0.72 (d, *J* = 6.9 Hz, 2H); ^{13}C NMR (101 MHz, Chloroform-*d*) δ 142.0, 139.6, 115.3, 114.7, 75.6, 45.2, 45.0, 38.6, 38.4, 32.3, 31.8, 29.7, 29.3, 28.8, 27.68, 27.56, 27.51, 27.48, 26.72, 26.62, 26.52, 20.88, 20.64, 20.40, 11.1, 10.8. IR 2857, 1472, 1066, 827, 764, 645 cm^{-1}; HRMS calculated for $C_{19}H_{39}O_2Si^+$ 327.2719 found 327.2703.

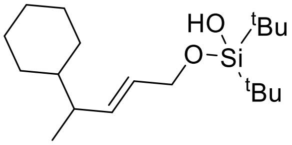

(*E*)-di-*tert*-butyl((4-cyclohexylpent-2-en-1-yl)oxy)silanol

Compound 56 isomer: synthesized using protocol A; purified using a gradient of 0-40% DCM:hexanes; (Colorless oil, 42% yield); ^{1}H NMR (400 MHz, Chloroform-*d*) δ 5.65 – 5.45 (m, 2H), 4.33 (dd, *J* = 5.4, 1.1 Hz, 2H), 1.99 (q, *J* = 6.8 Hz, 1H), 1.78 – 1.62 (m, 7H), 1.29 – 1.12 (m, 5H), 1.05 (s, 18H), 0.98 (d, *J* = 6.9 Hz, 3H); ^{13}C NMR (101 MHz, Chloroform-*d*) δ 135.9, 128.2, 64.3, 43.1, 41.8, 30.3, 27.5, 27.4, 26.6, 20.4, 17.4; IR 2926, 1472, 1096, 826 cm^{-1}.; HRMS calculated for $C_{19}H_{39}O_2Si^+$ 327.2719 found 327.2703.

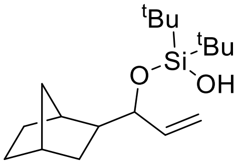

((1-((1*S*,4*R*)-bicyclo[2.2.1]heptan-2-yl)allyl)oxy)di-*tert*-butylsilanol

Compound 57 (Mixture of diastereomers): synthesized using protocol A; purified using a gradient of 0-40% DCM:hexanes; (Colorless oil, 72% yield);^{1}H NMR (400 MHz, Chloroform-*d*) δ 5.85 (ddt, *J* = 17.4, 10.3, 8.3 Hz, 0.8H), 5.76 – 5.66 (m, 0.2 H), 5.25 – 5.02 (m, 2H), 4.25 – 4.08 (m, 0.8H), 3.98 – 3.88 (m, 0.2H), 2.48 – 2.37 (m, 0.6H), 2.26 – 2.17 (m, 0.6H), 2.16-2.12 (m, 0.4H), 2.11 – 2.02 (m, 0.4H), 1.91 – 1.76 (m, 2H), 1.75-1.65 (m, 0.5H), 1.55-1.45 (m, 2H), 1.44-1.38 (m, 0.4H), 1.37-1.25 (m, 2.4H), 1.24-1.10 (m, 2H), 1.05 (s, 2H), 1.04 (s, 3.6H), 1.03 (s, 3H), 1.02 (s, 3.5H), 1.00 (s, 2H), 0.99 (s, 3H), 0.95-0.8 (m, 0.4H) 0.59 (ddd, *J* = 12.2, 6.1, 2.3 Hz, 1H); ^{13}C NMR (101 MHz, Chloroform-*d*) δ 142.3, 115.2, 114.8, 114.2, 78.2, 47.9, 47.8, 39.8, 39.7, 38.6, 38.3, 37.8, 37.1, 36.7, 36.5, 35.3, 35.1, 32.8, 30.5, 30.1, 28.8, 27.67, 27.65, 27.55, 27.51, 27.48, 23.1, 22.7, 20.7, 20.3; IR 2946, 1472, 1070, 825, 544 cm^{-1}.; HRMS calculated for $C_{18}H_{33}O_2Si^-$ 309.2250 found 309.2209.

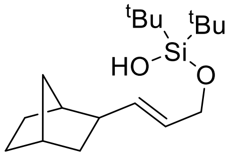

(((*E*)-3-((1*S*,4*R*)-bicyclo[2.2.1]heptan-2-yl)allyl)oxy)di-*tert*-butylsilanol

Compound 57 isomer (*dr* = 5:1): synthesized using protocol A; purified using a gradient of 0-40% DCM:hexanes; (Colorless oil, 8% yield); ^{1}H NMR (400 MHz, Chloroform-*d*) δ 5.72 (ddt, *J* = 15.3, 7.4, 1.4 Hz, 0.8H), 5.62 – 5.43 (m, 1H), 5.50 (dtd, *J* = 15.2, 5.3, 0.9 Hz, 0.2H), 4.35 (dt, *J* = 5.3, 1.1 Hz, 1.6H), 4.31 (dt, *J* = 5.3, 1.1 Hz, 0.4H), 2.55 – 2.44 (m, 1H), 2.26 – 2.03 (m, 2H), 1.86 (s, 1H), 1.82 – 1.70 (m, 1H), 1.51 (m, 2H), 1.41 – 1.23 (m, 3H), 1.19 – 1.09 (m, 1H), 1.06 (s, 13H), 1.05 (s, 5H),0.92 (m, 1H); ^{13}C NMR (101 MHz, Chloroform-*d*) δ 136.8, 134.4, 129.0, 126.6, 64.2, 44.4, 42.7, 42.4, 42.2, 39.9, 37.7, 37.3, 36.6, 35.9, 35.6, 30.2, 29.7, 29.0, 27.4, 23.0, 20.4; IR 2947, 1473, 1116, 827 cm^{-1}.; HRMS calculated for $C_{18}H_{33}O_2Si^-$ 309.2250 found 309.2219.

V. Procedure for dihydroxylation

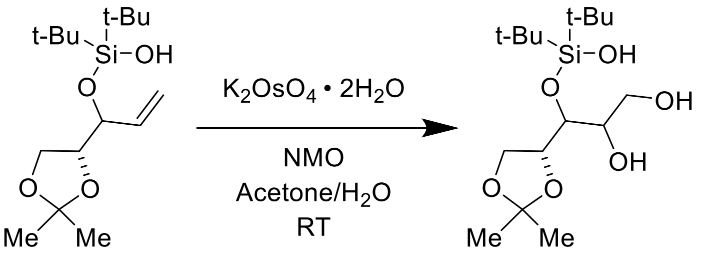

8 **58**

8 (33.4 mg, 0.106 mmol) was dissolved in 1 mL of Acetone and 0.1 mL of H_2O and transferred to a 10 mL round-bottom flask with magnetic stir-bar. 5 mg of K_2OsO_4•$2H_2O$ (0.014 mmol, 0.12 equiv.) was added followed by 31 mg of NMO (0.229 mmol, 2.2 equiv.). The mixture was stirred for 40 h upon which it was transferred to a separatory funnel with EtOAc and diluted with additional H_2O. The organic layer was separated, and the water layer was extracted twice with EtOAc. The organic fractions were combined, dried over $MgSO_4$, and concentrated under reduced pressure. The resulting residue was purified by chromatography on silica gel (gradient of 1 to 6% MeOH in CH_2Cl_2) to yield 15 mg of **58** as a single diastereomer (40% yield).

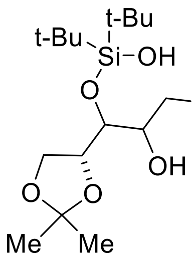

3-((di-*tert*-butyl(hydroxy)silyl)oxy)-3-((*R*)-2,2-dimethyl-1,3-dioxolan-4-yl)propane-1,2-diol

Compound 58: single diastereomer, relative stereochemistry unassigned; purified using a gradient of 0 to 5% MeOH in DCM; (colorless oil, 40% yield); ^{1}H NMR (400 MHz, $CDCl_3$) δ 4.19 (dd, *J* = 6.2, 3.4 Hz, 1H), 4.06 (q, *J* = 6.3 Hz, 1H), 4.04 – 3.98 (m, 1H), 3.98 – 3.92 (m, 1H), 3.92 – 3.85 (m, 1H), 3.71 (d, *J* = 5.4 Hz, 2H), 1.38 (s, 3H), 1.28 (s, 3H), 0.99 (s, 9H), 0.96 (s, 9H). ^{13}C NMR (101 MHz, $CDCl_3$) δ 109.23, 75.97, 75.13, 73.46, 66.43, 62.28, 27.95, 27.59, 26.52, 25.13, 21.08, 20.68. IR 3396, 2943, 2855, 1383, 1077 cm^{-1}. HRMS calculated for $C_{16}H_{33}O_6Si^-$ 349.2052 Found 349.2057. $[\alpha]_D$ = +12.57 (c = 0.56, $CHCl_3$)

VI. Crystal Structure Data for 18 (CCDC Number: 2052702)

Crystals grown from EtOAc/pentane.

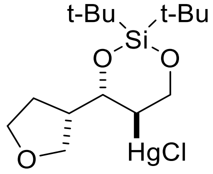

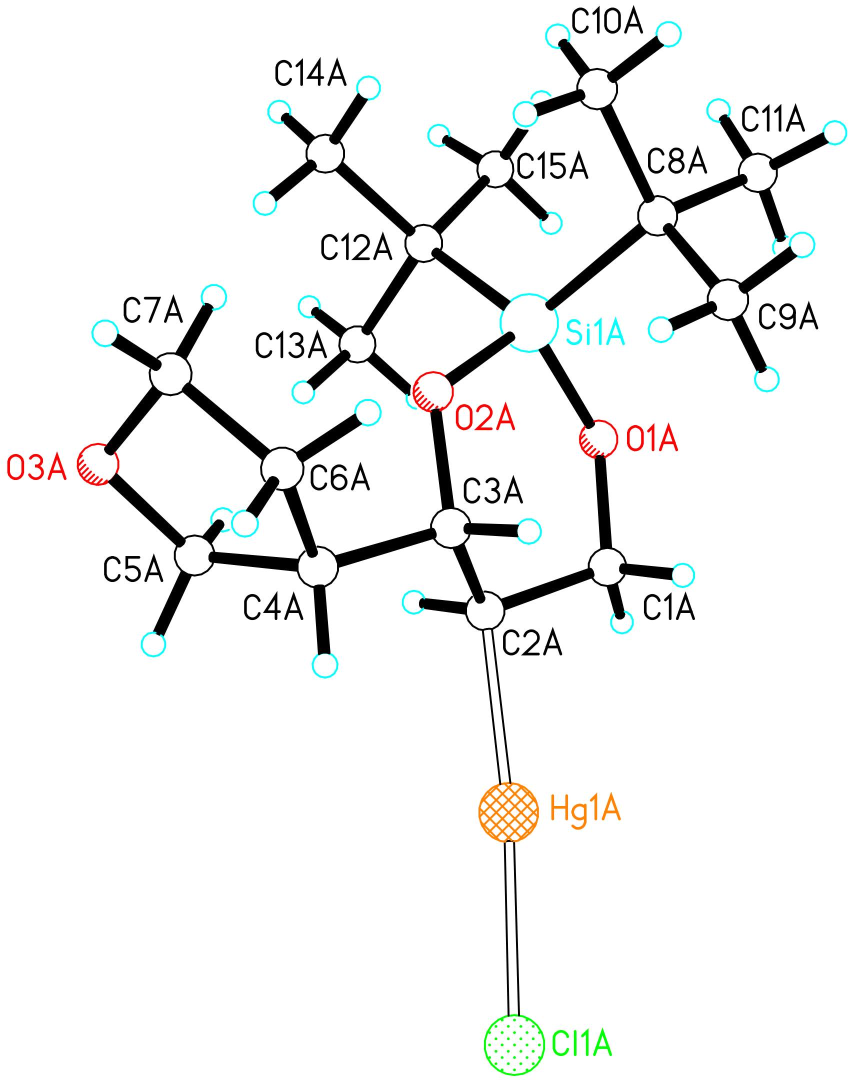

Table S1. Crystal data and structure refinement for HgCl(C15H29O3Si).

Identification code	q20l	
Empirical formula	C30 H58 Cl2 Hg2 O6 Si2	
Formula weight	1043.02	
Temperature	200(2) K	
Wavelength	1.54178 Å	
Crystal system	Triclinic	
Space group	P-1	
Unit cell dimensions	a = 8.9987(4) Å	α= 80.030(2)°.
	b = 11.7133(5) Å	β= 89.554(2)°.
	c = 18.4226(8) Å	γ = 86.392(3)°.
Volume	1908.70(14) Å^3	
Z	2	
Density (calculated)	1.815 Mg/m^3	
Absorption coefficient	16.407 mm^{-1}	
F(000)	1016	
Crystal size	0.098 x 0.032 x 0.023 mm^3	
Theta range for data collection	2.435 to 70.344°.	
Index ranges	-10<=h<=10, -13<=k<=13, -21<=l<=21	
Reflections collected	24512	
Independent reflections	6719 [R(int) = 0.0575]	
Completeness to theta = 66.000°	96.3 %	
Absorption correction	Numerical face-indexed	
Max. and min. transmission	0.3912 and 0.0621	
Refinement method	Full-matrix least-squares on F^2	
Data / restraints / parameters	6719 / 0 / 391	
Goodness-of-fit on F^2	1.052	
Final R indices [I>2sigma(I)]	R1 = 0.0536, wR2 = 0.1457	
R indices (all data)	R1 = 0.0612, wR2 = 0.1560	
Extinction coefficient	n/a	
Largest diff. peak and hole	3.396 and -4.154 e.Å^{-3}	

Table S2. Atomic coordinates (x 10^4) and equivalent isotropic displacement parameters (Å^{2}x 10^3) for HgCl(C15H29O3Si). U(eq) is defined as one third of the trace of the orthogonalized U^{ij} tensor.

	x	y	z	U(eq)
Hg(1A)	1943(1)	-941(1)	5481(1)	37(1)
Cl(1A)	1162(2)	-1277(2)	4335(1)	42(1)
Si(1A)	3261(2)	-269(2)	8044(1)	33(1)
O(1A)	3079(8)	-1512(5)	7771(3)	47(2)
O(2A)	3105(6)	735(5)	7298(3)	34(1)
O(3A)	4818(9)	2821(7)	6032(5)	67(2)
C(1A)	2424(12)	-1588(8)	7077(5)	46(2)
C(2A)	2863(9)	-618(7)	6466(4)	33(2)
C(3A)	2394(9)	574(7)	6626(4)	34(2)
C(4A)	2799(9)	1604(7)	6036(4)	36(2)
C(5A)	4475(10)	1686(8)	5909(5)	44(2)
C(6A)	2301(11)	2775(8)	6253(5)	47(2)
C(7A)	3702(12)	3223(9)	6500(6)	52(2)
C(8A)	1719(11)	-12(10)	8707(5)	52(2)
C(9A)	274(14)	120(20)	8273(8)	112(7)
C(10A)	1907(17)	1090(12)	9025(8)	80(4)
C(11A)	1636(19)	-1028(14)	9348(8)	98(5)
C(12A)	5219(11)	-277(9)	8392(6)	50(2)
C(13A)	6260(13)	-747(13)	7839(7)	73(4)
C(14A)	5635(13)	955(12)	8468(8)	75(4)
C(15A)	5489(17)	-1073(12)	9141(7)	84(4)
Hg(1B)	7265(1)	3910(1)	5461(1)	38(1)
Cl(1B)	6484(3)	3589(2)	4314(1)	46(1)
Si(1B)	8444(2)	4687(2)	8018(1)	34(1)
O(1B)	8582(9)	3438(6)	7732(4)	56(2)
O(2B)	7996(6)	5679(5)	7284(3)	36(1)
O(3B)	9318(10)	7981(7)	5935(5)	68(2)
C(1B)	7932(12)	3271(8)	7051(5)	48(2)
C(2B)	8116(10)	4302(7)	6443(4)	33(2)
C(3B)	7378(9)	5435(7)	6616(4)	33(2)
C(4B)	7558(9)	6515(7)	6029(5)	36(2)

C(5B)	9196(10)	6787(8)	5860(5)	41(2)
C(6B)	6867(11)	7623(8)	6269(5)	46(2)
C(7B)	8215(12)	8243(8)	6452(6)	50(2)
C(8B)	6905(11)	4672(10)	8721(5)	52(2)
C(9B)	5462(14)	4570(19)	8328(8)	107(7)
C(10B)	6843(17)	5768(13)	9067(8)	86(4)
C(11B)	7142(16)	3616(13)	9342(7)	83(4)
C(12B)	10330(10)	5001(10)	8318(5)	50(2)
C(13B)	11464(12)	4710(17)	7731(7)	100(6)
C(14B)	10406(17)	6283(13)	8395(8)	88(5)
C(15B)	10857(13)	4274(12)	9052(6)	68(3)

Table S3. Bond lengths [Å] and angles [°] for HgCl(C15H29O3Si).

Hg(1A)-C(2A)	2.103(8)
Hg(1A)-Cl(1A)	2.335(2)
Si(1A)-O(1A)	1.638(6)
Si(1A)-O(2A)	1.646(6)
Si(1A)-C(12A)	1.879(10)
Si(1A)-C(8A)	1.886(10)
O(1A)-C(1A)	1.432(10)
O(2A)-C(3A)	1.445(9)
O(3A)-C(7A)	1.429(12)
O(3A)-C(5A)	1.440(11)
C(1A)-C(2A)	1.523(12)
C(1A)-H(1AA)	0.9900
C(1A)-H(1AB)	0.9900
C(2A)-C(3A)	1.510(11)
C(2A)-H(2AA)	1.0000
C(3A)-C(4A)	1.538(11)
C(3A)-H(3AA)	1.0000
C(4A)-C(5A)	1.530(11)
C(4A)-C(6A)	1.533(13)
C(4A)-H(4AA)	1.0000
C(5A)-H(5AA)	0.9900
C(5A)-H(5AB)	0.9900
C(6A)-C(7A)	1.499(13)
C(6A)-H(6AA)	0.9900
C(6A)-H(6AB)	0.9900
C(7A)-H(7AA)	0.9900
C(7A)-H(7AB)	0.9900
C(8A)-C(9A)	1.517(17)
C(8A)-C(10A)	1.526(15)
C(8A)-C(11A)	1.529(16)
C(9A)-H(9AA)	0.9800
C(9A)-H(9AB)	0.9800
C(9A)-H(9AC)	0.9800
C(10A)-H(10A)	0.9800
C(10A)-H(10B)	0.9800
C(10A)-H(10C)	0.9800
C(11A)-H(11A)	0.9800
C(11A)-H(11B)	0.9800
C(11A)-H(11C)	0.9800
C(12A)-C(13A)	1.527(14)
C(12A)-C(15A)	1.537(16)
C(12A)-C(14A)	1.543(15)
C(13A)-H(13A)	0.9800
C(13A)-H(13B)	0.9800
C(13A)-H(13C)	0.9800
C(14A)-H(14A)	0.9800
C(14A)-H(14B)	0.9800
C(14A)-H(14C)	0.9800
C(15A)-H(15A)	0.9800
C(15A)-H(15B)	0.9800
C(15A)-H(15C)	0.9800
Hg(1B)-C(2B)	2.102(8)
Hg(1B)-Cl(1B)	2.332(2)
Si(1B)-O(1B)	1.637(7)
Si(1B)-O(2B)	1.657(6)
Si(1B)-C(12B)	1.867(9)
Si(1B)-C(8B)	1.887(10)
O(1B)-C(1B)	1.436(10)
O(2B)-C(3B)	1.435(9)
O(3B)-C(7B)	1.426(12)
O(3B)-C(5B)	1.441(12)
C(1B)-C(2B)	1.515(12)
C(1B)-H(1BA)	0.9900
C(1B)-H(1BB)	0.9900
C(2B)-C(3B)	1.529(11)
C(2B)-H(2BA)	1.0000
C(3B)-C(4B)	1.532(11)
C(3B)-H(3BA)	1.0000

C(4B)-C(6B)	1.536(12)
C(4B)-C(5B)	1.545(11)
C(4B)-H(4BA)	1.0000
C(5B)-H(5BA)	0.9900
C(5B)-H(5BB)	0.9900
C(6B)-C(7B)	1.522(13)
C(6B)-H(6BA)	0.9900
C(6B)-H(6BB)	0.9900
C(7B)-H(7BA)	0.9900
C(7B)-H(7BB)	0.9900
C(8B)-C(9B)	1.513(15)
C(8B)-C(10B)	1.527(17)
C(8B)-C(11B)	1.538(16)
C(9B)-H(9BA)	0.9800
C(9B)-H(9BB)	0.9800
C(9B)-H(9BC)	0.9800
C(10B)-H(10D)	0.9800
C(10B)-H(10E)	0.9800
C(10B)-H(10F)	0.9800
C(11B)-H(11D)	0.9800
C(11B)-H(11E)	0.9800
C(11B)-H(11F)	0.9800
C(12B)-C(15B)	1.529(14)
C(12B)-C(14B)	1.538(17)
C(12B)-C(13B)	1.547(15)
C(13B)-H(13D)	0.9800
C(13B)-H(13E)	0.9800
C(13B)-H(13F)	0.9800
C(14B)-H(14D)	0.9800
C(14B)-H(14E)	0.9800
C(14B)-H(14F)	0.9800
C(15B)-H(15D)	0.9800
C(15B)-H(15E)	0.9800
C(15B)-H(15F)	0.9800
C(2A)-Hg(1A)-Cl(1A)	174.2(2)

O(1A)-Si(1A)-O(2A)	106.2(3)
O(1A)-Si(1A)-C(12A)	107.5(4)
O(2A)-Si(1A)-C(12A)	106.8(4)
O(1A)-Si(1A)-C(8A)	109.5(4)
O(2A)-Si(1A)-C(8A)	109.7(4)
C(12A)-Si(1A)-C(8A)	116.6(5)
C(1A)-O(1A)-Si(1A)	122.4(6)
C(3A)-O(2A)-Si(1A)	123.8(5)
C(7A)-O(3A)-C(5A)	108.5(7)
O(1A)-C(1A)-C(2A)	112.3(7)
O(1A)-C(1A)-H(1AA)	109.1
C(2A)-C(1A)-H(1AA)	109.1
O(1A)-C(1A)-H(1AB)	109.1
C(2A)-C(1A)-H(1AB)	109.1
H(1AA)-C(1A)-H(1AB)	107.9
C(3A)-C(2A)-C(1A)	112.5(7)
C(3A)-C(2A)-Hg(1A)	112.8(6)
C(1A)-C(2A)-Hg(1A)	107.0(5)
C(3A)-C(2A)-H(2AA)	108.1
C(1A)-C(2A)-H(2AA)	108.1
Hg(1A)-C(2A)-H(2AA)	108.1
O(2A)-C(3A)-C(2A)	109.2(7)
O(2A)-C(3A)-C(4A)	105.9(6)
C(2A)-C(3A)-C(4A)	115.9(7)
O(2A)-C(3A)-H(3AA)	108.6
C(2A)-C(3A)-H(3AA)	108.6
C(4A)-C(3A)-H(3AA)	108.6
C(5A)-C(4A)-C(6A)	103.7(7)
C(5A)-C(4A)-C(3A)	114.0(7)
C(6A)-C(4A)-C(3A)	112.1(7)
C(5A)-C(4A)-H(4AA)	109.0
C(6A)-C(4A)-H(4AA)	109.0
C(3A)-C(4A)-H(4AA)	109.0
O(3A)-C(5A)-C(4A)	106.3(8)
O(3A)-C(5A)-H(5AA)	110.5
C(4A)-C(5A)-H(5AA)	110.5

O(3A)-C(5A)-H(5AB)	110.5	C(8A)-C(11A)-H(11C)	109.5
C(4A)-C(5A)-H(5AB)	110.5	H(11A)-C(11A)-H(11C)	109.5
H(5AA)-C(5A)-H(5AB)	108.7	H(11B)-C(11A)-H(11C)	109.5
C(7A)-C(6A)-C(4A)	104.5(8)	C(13A)-C(12A)-C(15A)	107.0(10)
C(7A)-C(6A)-H(6AA)	110.8	C(13A)-C(12A)-C(14A)	109.4(10)
C(4A)-C(6A)-H(6AA)	110.8	C(15A)-C(12A)-C(14A)	108.1(10)
C(7A)-C(6A)-H(6AB)	110.8	C(13A)-C(12A)-Si(1A)	107.7(7)
C(4A)-C(6A)-H(6AB)	110.8	C(15A)-C(12A)-Si(1A)	113.1(9)
H(6AA)-C(6A)-H(6AB)	108.9	C(14A)-C(12A)-Si(1A)	111.4(7)
O(3A)-C(7A)-C(6A)	103.6(8)	C(12A)-C(13A)-H(13A)	109.5
O(3A)-C(7A)-H(7AA)	111.0	C(12A)-C(13A)-H(13B)	109.5
C(6A)-C(7A)-H(7AA)	111.0	H(13A)-C(13A)-H(13B)	109.5
O(3A)-C(7A)-H(7AB)	111.0	C(12A)-C(13A)-H(13C)	109.5
C(6A)-C(7A)-H(7AB)	111.0	H(13A)-C(13A)-H(13C)	109.5
H(7AA)-C(7A)-H(7AB)	109.0	H(13B)-C(13A)-H(13C)	109.5
C(9A)-C(8A)-C(10A)	109.1(12)	C(12A)-C(14A)-H(14A)	109.5
C(9A)-C(8A)-C(11A)	108.9(12)	C(12A)-C(14A)-H(14B)	109.5
C(10A)-C(8A)-C(11A)	108.3(11)	H(14A)-C(14A)-H(14B)	109.5
C(9A)-C(8A)-Si(1A)	106.8(7)	C(12A)-C(14A)-H(14C)	109.5
C(10A)-C(8A)-Si(1A)	111.5(7)	H(14A)-C(14A)-H(14C)	109.5
C(11A)-C(8A)-Si(1A)	112.2(9)	H(14B)-C(14A)-H(14C)	109.5
C(8A)-C(9A)-H(9AA)	109.5	C(12A)-C(15A)-H(15A)	109.5
C(8A)-C(9A)-H(9AB)	109.5	C(12A)-C(15A)-H(15B)	109.5
H(9AA)-C(9A)-H(9AB)	109.5	H(15A)-C(15A)-H(15B)	109.5
C(8A)-C(9A)-H(9AC)	109.5	C(12A)-C(15A)-H(15C)	109.5
H(9AA)-C(9A)-H(9AC)	109.5	H(15A)-C(15A)-H(15C)	109.5
H(9AB)-C(9A)-H(9AC)	109.5	H(15B)-C(15A)-H(15C)	109.5
C(8A)-C(10A)-H(10A)	109.5	C(2B)-Hg(1B)-Cl(1B)	174.6(2)
C(8A)-C(10A)-H(10B)	109.5	O(1B)-Si(1B)-O(2B)	106.1(3)
H(10A)-C(10A)-H(10B)	109.5	O(1B)-Si(1B)-C(12B)	108.2(5)
C(8A)-C(10A)-H(10C)	109.5	O(2B)-Si(1B)-C(12B)	106.6(4)
H(10A)-C(10A)-H(10C)	109.5	O(1B)-Si(1B)-C(8B)	109.3(5)
H(10B)-C(10A)-H(10C)	109.5	O(2B)-Si(1B)-C(8B)	109.4(4)
C(8A)-C(11A)-H(11A)	109.5	C(12B)-Si(1B)-C(8B)	116.7(4)
C(8A)-C(11A)-H(11B)	109.5	C(1B)-O(1B)-Si(1B)	122.6(6)
H(11A)-C(11A)-H(11B)	109.5	C(3B)-O(2B)-Si(1B)	124.7(5)

C(7B)-O(3B)-C(5B)	107.1(7)
O(1B)-C(1B)-C(2B)	111.7(7)
O(1B)-C(1B)-H(1BA)	109.3
C(2B)-C(1B)-H(1BA)	109.3
O(1B)-C(1B)-H(1BB)	109.3
C(2B)-C(1B)-H(1BB)	109.3
H(1BA)-C(1B)-H(1BB)	107.9
C(1B)-C(2B)-C(3B)	113.2(7)
C(1B)-C(2B)-Hg(1B)	108.3(5)
C(3B)-C(2B)-Hg(1B)	111.1(6)
C(1B)-C(2B)-H(2BA)	108.0
C(3B)-C(2B)-H(2BA)	108.0
Hg(1B)-C(2B)-H(2BA)	108.0
O(2B)-C(3B)-C(2B)	109.5(6)
O(2B)-C(3B)-C(4B)	106.4(6)
C(2B)-C(3B)-C(4B)	115.7(7)
O(2B)-C(3B)-H(3BA)	108.4
C(2B)-C(3B)-H(3BA)	108.4
C(4B)-C(3B)-H(3BA)	108.4
C(3B)-C(4B)-C(6B)	112.1(7)
C(3B)-C(4B)-C(5B)	113.9(7)
C(6B)-C(4B)-C(5B)	104.0(7)
C(3B)-C(4B)-H(4BA)	108.9
C(6B)-C(4B)-H(4BA)	108.9
C(5B)-C(4B)-H(4BA)	108.9
O(3B)-C(5B)-C(4B)	106.4(7)
O(3B)-C(5B)-H(5BA)	110.4
C(4B)-C(5B)-H(5BA)	110.4
O(3B)-C(5B)-H(5BB)	110.4
C(4B)-C(5B)-H(5BB)	110.4
H(5BA)-C(5B)-H(5BB)	108.6
C(7B)-C(6B)-C(4B)	103.4(7)
C(7B)-C(6B)-H(6BA)	111.1
C(4B)-C(6B)-H(6BA)	111.1
C(7B)-C(6B)-H(6BB)	111.1
C(4B)-C(6B)-H(6BB)	111.1
H(6BA)-C(6B)-H(6BB)	109.0
O(3B)-C(7B)-C(6B)	104.3(8)
O(3B)-C(7B)-H(7BA)	110.9
C(6B)-C(7B)-H(7BA)	110.9
O(3B)-C(7B)-H(7BB)	110.9
C(6B)-C(7B)-H(7BB)	110.9
H(7BA)-C(7B)-H(7BB)	108.9
C(9B)-C(8B)-C(10B)	111.0(13)
C(9B)-C(8B)-C(11B)	108.5(11)
C(10B)-C(8B)-C(11B)	108.1(10)
C(9B)-C(8B)-Si(1B)	107.2(8)
C(10B)-C(8B)-Si(1B)	111.3(8)
C(11B)-C(8B)-Si(1B)	110.7(8)
C(8B)-C(9B)-H(9BA)	109.5
C(8B)-C(9B)-H(9BB)	109.5
H(9BA)-C(9B)-H(9BB)	109.5
C(8B)-C(9B)-H(9BC)	109.5
H(9BA)-C(9B)-H(9BC)	109.5
H(9BB)-C(9B)-H(9BC)	109.5
C(8B)-C(10B)-H(10D)	109.5
C(8B)-C(10B)-H(10E)	109.5
H(10D)-C(10B)-H(10E)	109.5
C(8B)-C(10B)-H(10F)	109.5
H(10D)-C(10B)-H(10F)	109.5
H(10E)-C(10B)-H(10F)	109.5
C(8B)-C(11B)-H(11D)	109.5
C(8B)-C(11B)-H(11E)	109.5
H(11D)-C(11B)-H(11E)	109.5
C(8B)-C(11B)-H(11F)	109.5
H(11D)-C(11B)-H(11F)	109.5
H(11E)-C(11B)-H(11F)	109.5
C(15B)-C(12B)-C(14B)	106.9(9)
C(15B)-C(12B)-C(13B)	106.3(10)
C(14B)-C(12B)-C(13B)	109.4(12)
C(15B)-C(12B)-Si(1B)	114.5(8)
C(14B)-C(12B)-Si(1B)	111.7(8)

C(13B)-C(12B)-Si(1B)	107.8(7)
C(12B)-C(13B)-H(13D)	109.5
C(12B)-C(13B)-H(13E)	109.5
H(13D)-C(13B)-H(13E)	109.5
C(12B)-C(13B)-H(13F)	109.5
H(13D)-C(13B)-H(13F)	109.5
H(13E)-C(13B)-H(13F)	109.5
C(12B)-C(14B)-H(14D)	109.5
C(12B)-C(14B)-H(14E)	109.5
H(14D)-C(14B)-H(14E)	109.5
C(12B)-C(14B)-H(14F)	109.5
H(14D)-C(14B)-H(14F)	109.5
H(14E)-C(14B)-H(14F)	109.5
C(12B)-C(15B)-H(15D)	109.5
C(12B)-C(15B)-H(15E)	109.5
H(15D)-C(15B)-H(15E)	109.5
C(12B)-C(15B)-H(15F)	109.5
H(15D)-C(15B)-H(15F)	109.5
H(15E)-C(15B)-H(15F)	109.5

Symmetry transformations used to generate equivalent atoms:

Table S4. Anisotropic displacement parameters ($Å^2 x 10^3$) for HgCl(C15H29O3Si). The anisotropic displacement factor exponent takes the form: $-2\pi^2[h^2 a^{*2}U^{11} + ... + 2 h k a^* b^* U^{12}]$

	U^{11}	U^{22}	U^{33}	U^{23}	U^{13}	U^{12}
Hg(1A)	33(1)	38(1)	42(1)	-12(1)	-3(1)	-11(1)
Cl(1A)	42(1)	42(1)	43(1)	-8(1)	-7(1)	-7(1)
Si(1A)	28(1)	35(1)	38(1)	-10(1)	-2(1)	-4(1)
O(1A)	62(4)	38(3)	41(3)	-9(3)	-12(3)	-5(3)
O(2A)	36(3)	33(3)	35(3)	-8(2)	-2(2)	-7(2)
O(3A)	62(5)	65(5)	83(5)	-37(4)	27(4)	-30(4)
C(1A)	59(6)	43(5)	38(4)	-12(4)	-3(4)	-17(4)
C(2A)	34(4)	36(4)	31(4)	-7(3)	-1(3)	-5(3)
C(3A)	26(4)	35(4)	41(4)	-9(3)	-3(3)	-6(3)
C(4A)	32(4)	41(5)	36(4)	-6(3)	-3(3)	-4(3)
C(5A)	40(5)	44(5)	51(5)	-11(4)	7(4)	-16(4)
C(6A)	44(5)	44(5)	50(5)	-2(4)	-1(4)	-1(4)
C(7A)	57(6)	39(5)	61(6)	-15(4)	5(5)	-7(4)
C(8A)	43(5)	62(6)	51(5)	-13(5)	9(4)	-8(5)
C(9A)	34(7)	220(20)	87(10)	-48(12)	17(6)	-12(10)
C(10A)	90(10)	69(8)	90(9)	-34(7)	43(8)	-15(7)
C(11A)	118(13)	93(11)	76(9)	5(8)	50(9)	-22(9)
C(12A)	38(5)	55(6)	60(6)	-26(5)	-13(4)	6(4)
C(13A)	44(6)	114(11)	67(7)	-37(7)	-6(5)	2(7)
C(14A)	44(6)	78(8)	115(10)	-43(8)	-25(6)	-9(6)
C(15A)	96(11)	88(9)	66(7)	-25(7)	-38(7)	39(8)
Hg(1B)	38(1)	36(1)	43(1)	-12(1)	-4(1)	-11(1)
Cl(1B)	52(1)	45(1)	44(1)	-10(1)	-8(1)	-6(1)
Si(1B)	30(1)	34(1)	39(1)	-10(1)	-4(1)	-3(1)
O(1B)	83(5)	36(3)	49(4)	-11(3)	-20(3)	4(3)
O(2B)	33(3)	37(3)	40(3)	-9(2)	-3(2)	-4(2)
O(3B)	73(5)	58(4)	85(5)	-36(4)	35(4)	-36(4)
C(1B)	70(7)	33(5)	41(5)	-7(4)	-14(4)	-10(4)
C(2B)	38(4)	30(4)	35(4)	-9(3)	0(3)	-14(3)
C(3B)	24(4)	36(4)	41(4)	-13(3)	-4(3)	0(3)
C(4B)	31(4)	37(4)	41(4)	-7(3)	2(3)	-6(3)

C(5B)	33(5)	45(5)	47(5)	-7(4)	7(3)	-14(4)
C(6B)	47(6)	37(5)	53(5)	-9(4)	-6(4)	12(4)
C(7B)	55(6)	38(5)	60(6)	-14(4)	11(4)	-10(4)
C(8B)	42(5)	63(6)	50(5)	-5(4)	3(4)	-13(5)
C(9B)	38(7)	210(20)	68(8)	-11(10)	3(5)	-30(10)
C(10B)	86(10)	88(10)	84(9)	-25(8)	35(8)	9(8)
C(11B)	79(9)	100(10)	62(7)	12(7)	18(6)	-22(8)
C(12B)	27(5)	75(7)	51(5)	-19(5)	-9(4)	0(4)
C(13B)	19(5)	220(20)	71(8)	-53(10)	-10(5)	10(8)
C(14B)	83(10)	86(10)	101(10)	-17(8)	-31(8)	-42(8)
C(15B)	54(7)	88(9)	59(6)	-15(6)	-18(5)	20(6)

Table S5. Hydrogen coordinates (x 10^4) and isotropic displacement parameters (Å^{2}x 10^3) for HgCl(C15H29O3Si).

	x	y	z	U(eq)
H(1AA)	1326	-1551	7126	55
H(1AB)	2740	-2347	6940	55
H(2AA)	3972	-674	6419	40
H(3AA)	1291	621	6699	40
H(4AA)	2309	1543	5561	43
H(5AA)	5040	1075	6256	53
H(5AB)	4734	1587	5399	53
H(6AA)	1852	3314	5827	56
H(6AB)	1564	2672	6658	56
H(7AA)	3629	4082	6432	62
H(7AB)	3918	2904	7024	62
H(9AA)	-561	258	8599	167
H(9AB)	141	-586	8072	167
H(9AC)	308	784	7868	167
H(10A)	1045	1236	9331	120
H(10B)	1983	1749	8621	120
H(10C)	2815	993	9325	120
H(11A)	702	-942	9616	146
H(11B)	2479	-1034	9682	146
H(11C)	1673	-1759	9156	146
H(13A)	7288	-794	8018	109
H(13B)	6177	-226	7362	109
H(13C)	5983	-1523	7782	109
H(14A)	6633	914	8684	113
H(14B)	4912	1285	8787	113
H(14C)	5626	1447	7980	113
H(15A)	6529	-1053	9290	125
H(15B)	5280	-1870	9098	125
H(15C)	4831	-804	9511	125
H(1BA)	6859	3151	7127	57

H(1BB)	8410	2564	6901	57
H(2BA)	9205	4405	6373	40
H(3BA)	6290	5332	6693	40
H(4BA)	7063	6407	5563	43
H(5BA)	9873	6269	6210	49
H(5BB)	9459	6675	5353	49
H(6BA)	6273	8104	5865	55
H(6BB)	6223	7433	6706	55
H(7BA)	7980	9091	6394	60
H(7BB)	8557	7949	6964	60
H(9BA)	4648	4489	8685	161
H(9BB)	5550	3886	8086	161
H(9BC)	5255	5268	7956	161
H(10D)	5929	5808	9357	129
H(10E)	6852	6453	8678	129
H(10F)	7710	5746	9390	129
H(11D)	6210	3486	9614	124
H(11E)	7918	3763	9679	124
H(11F)	7448	2927	9130	124
H(13D)	12431	4995	7828	150
H(13E)	11110	5084	7240	150
H(13F)	11568	3867	7753	150
H(14D)	11407	6415	8556	132
H(14E)	9675	6473	8760	132
H(14F)	10184	6778	7918	132
H(15D)	11863	4472	9163	101
H(15E)	10869	3447	9018	101
H(15F)	10178	4435	9445	101

Table S6. Torsion angles [°] for HgCl(C15H29O3Si).

O(2A)-Si(1A)-O(1A)-C(1A)	-18.5(8)
C(12A)-Si(1A)-O(1A)-C(1A)	-132.4(8)
C(8A)-Si(1A)-O(1A)-C(1A)	99.9(8)
O(1A)-Si(1A)-O(2A)-C(3A)	21.1(7)
C(12A)-Si(1A)-O(2A)-C(3A)	135.6(6)
C(8A)-Si(1A)-O(2A)-C(3A)	-97.1(7)
Si(1A)-O(1A)-C(1A)-C(2A)	38.5(11)
O(1A)-C(1A)-C(2A)-C(3A)	-59.8(10)
O(1A)-C(1A)-C(2A)-Hg(1A)	175.7(6)
Si(1A)-O(2A)-C(3A)-C(2A)	-42.5(8)
Si(1A)-O(2A)-C(3A)-C(4A)	-168.0(5)
C(1A)-C(2A)-C(3A)-O(2A)	60.4(9)
Hg(1A)-C(2A)-C(3A)-O(2A)	-178.4(5)
C(1A)-C(2A)-C(3A)-C(4A)	179.8(7)
Hg(1A)-C(2A)-C(3A)-C(4A)	-59.0(8)
O(2A)-C(3A)-C(4A)-C(5A)	59.5(9)
C(2A)-C(3A)-C(4A)-C(5A)	-61.7(10)
O(2A)-C(3A)-C(4A)-C(6A)	-57.9(8)
C(2A)-C(3A)-C(4A)-C(6A)	-179.1(7)
C(7A)-O(3A)-C(5A)-C(4A)	23.7(11)
C(6A)-C(4A)-C(5A)-O(3A)	-1.3(10)
C(3A)-C(4A)-C(5A)-O(3A)	-123.4(8)
C(5A)-C(4A)-C(6A)-C(7A)	-20.0(9)
C(3A)-C(4A)-C(6A)-C(7A)	103.4(8)
C(5A)-O(3A)-C(7A)-C(6A)	-36.6(11)
C(4A)-C(6A)-C(7A)-O(3A)	34.4(10)
O(1A)-Si(1A)-C(8A)-C(9A)	-65.4(11)
O(2A)-Si(1A)-C(8A)-C(9A)	50.7(11)
C(12A)-Si(1A)-C(8A)-C(9A)	172.3(10)
O(1A)-Si(1A)-C(8A)-C(10A)	175.5(9)
O(2A)-Si(1A)-C(8A)-C(10A)	-68.4(10)
C(12A)-Si(1A)-C(8A)-C(10A)	53.2(11)
O(1A)-Si(1A)-C(8A)-C(11A)	53.8(10)
O(2A)-Si(1A)-C(8A)-C(11A)	170.0(9)
C(12A)-Si(1A)-C(8A)-C(11A)	-68.5(11)
O(1A)-Si(1A)-C(12A)-C(13A)	44.9(9)
O(2A)-Si(1A)-C(12A)-C(13A)	-68.7(9)
C(8A)-Si(1A)-C(12A)-C(13A)	168.2(8)
O(1A)-Si(1A)-C(12A)-C(15A)	-73.1(8)
O(2A)-Si(1A)-C(12A)-C(15A)	173.2(7)
C(8A)-Si(1A)-C(12A)-C(15A)	50.2(9)
O(1A)-Si(1A)-C(12A)-C(14A)	164.9(8)
O(2A)-Si(1A)-C(12A)-C(14A)	51.2(9)
C(8A)-Si(1A)-C(12A)-C(14A)	-71.8(10)
O(2B)-Si(1B)-O(1B)-C(1B)	-18.8(9)
C(12B)-Si(1B)-O(1B)-C(1B)	-132.9(8)
C(8B)-Si(1B)-O(1B)-C(1B)	99.1(8)
O(1B)-Si(1B)-O(2B)-C(3B)	18.7(7)
C(12B)-Si(1B)-O(2B)-C(3B)	133.8(6)
C(8B)-Si(1B)-O(2B)-C(3B)	-99.1(7)
Si(1B)-O(1B)-C(1B)-C(2B)	40.3(12)
O(1B)-C(1B)-C(2B)-C(3B)	-60.4(11)
O(1B)-C(1B)-C(2B)-Hg(1B)	176.0(7)
Si(1B)-O(2B)-C(3B)-C(2B)	-38.6(8)
Si(1B)-O(2B)-C(3B)-C(4B)	-164.2(5)
C(1B)-C(2B)-C(3B)-O(2B)	58.5(9)
Hg(1B)-C(2B)-C(3B)-O(2B)	-179.4(5)
C(1B)-C(2B)-C(3B)-C(4B)	178.6(7)
Hg(1B)-C(2B)-C(3B)-C(4B)	-59.3(8)
O(2B)-C(3B)-C(4B)-C(6B)	-54.7(9)
C(2B)-C(3B)-C(4B)-C(6B)	-176.5(7)
O(2B)-C(3B)-C(4B)-C(5B)	63.0(8)
C(2B)-C(3B)-C(4B)-C(5B)	-58.8(9)
C(7B)-O(3B)-C(5B)-C(4B)	27.8(11)
C(3B)-C(4B)-C(5B)-O(3B)	-127.4(8)
C(6B)-C(4B)-C(5B)-O(3B)	-5.1(9)
C(3B)-C(4B)-C(6B)-C(7B)	106.0(8)
C(5B)-C(4B)-C(6B)-C(7B)	-17.5(9)
C(5B)-O(3B)-C(7B)-C(6B)	-39.4(11)

C(4B)-C(6B)-C(7B)-O(3B)	34.7(10)
O(1B)-Si(1B)-C(8B)-C(9B)	-64.6(11)
O(2B)-Si(1B)-C(8B)-C(9B)	51.2(11)
C(12B)-Si(1B)-C(8B)-C(9B)	172.3(11)
O(1B)-Si(1B)-C(8B)-C(10B)	173.8(9)
O(2B)-Si(1B)-C(8B)-C(10B)	-70.4(10)
C(12B)-Si(1B)-C(8B)-C(10B)	50.7(11)
O(1B)-Si(1B)-C(8B)-C(11B)	53.6(9)
O(2B)-Si(1B)-C(8B)-C(11B)	169.4(8)
C(12B)-Si(1B)-C(8B)-C(11B)	-69.5(10)
O(1B)-Si(1B)-C(12B)-C(15B)	-73.9(8)
O(2B)-Si(1B)-C(12B)-C(15B)	172.4(7)
C(8B)-Si(1B)-C(12B)-C(15B)	49.8(10)
O(1B)-Si(1B)-C(12B)-C(14B)	164.4(8)
O(2B)-Si(1B)-C(12B)-C(14B)	50.7(9)
C(8B)-Si(1B)-C(12B)-C(14B)	-71.9(10)
O(1B)-Si(1B)-C(12B)-C(13B)	44.2(10)
O(2B)-Si(1B)-C(12B)-C(13B)	-69.5(10)
C(8B)-Si(1B)-C(12B)-C(13B)	167.9(9)

Symmetry transformations used to generate equivalent atoms:

Table S7. Hydrogen bonds for HgCl(C15H29O3Si) [Å and °].

D-H...A	d(D-H)	d(H...A)	d(D...A)	<(DHA)
C(3A)-H(3AA)...Cl(1A)#1	1.00	2.90	3.647(8)	131.7
C(7A)-H(7AA)...Cl(1B)#2	0.99	2.83	3.768(11)	158.5
C(1B)-H(1BB)...Cl(1A)#3	0.99	2.95	3.799(10)	144.4
C(7B)-H(7BA)...Cl(1A)#2	0.99	2.83	3.674(10)	143.9

Symmetry transformations used to generate equivalent atoms:

#1 -x,-y,-z+1 #2 -x+1,-y+1,-z+1 #3 -x+1,-y,-z+1

VII: Computational Procedures and Results

Calculations were performed at home on a desktop gaming PC running Windows 10 with an Intel® Core™ i7-8700K CPU @ 3.70 GHz (6 CPUs) and 16 GB of RAM. The ORCA DFT package[1,2] was used for all DFT calculations. Calculation inputs were prepared using Avogadro[3], and when multiple conformers were possible, a systematic rotor search was performed to identify the lowest energy conformer as a starting point. DFT outputs were analyzed and visualized using Chemcraft[4]. DFT calculations were performed using the B3LYP[5,6] functional with the RIJCOSX approximation and D3BJ[7,8] dispersion correction using the def2-TZVP[9] basis set and def2/J[10] auxiliary basis set for RIJCOSX. For calculations involving mercury, the DEF2-ECP was automatically applied by ORCA, replacing 60 core electrons.[11] The SMD solvation module[12] was used to model implicit water solvation. The larger solvent accessible surface (SAS) was used instead of the default smaller solvent excluded surface (SES) on account of geometry convergence issues with the latter. For cationic mercuronium species, the chloride counterion was not explicitly modeled.

Example ORCA Input card for a groundstate opt-freq job:

```
! RKS RIJCOSX B3LYP D3BJ def2-TZVP def2/J          #Level of theory
! Grid5 FinalGrid6 GridX7 tightSCF slowconv         #Tight grids lead to best results
! OPT FREQ
! pal6                                              #6 CPUs used on local machine
%maxcore 2048                                       #2GB RAM allocated per CPU
! CPCM(Water)                                       #Implicit water solvation
%cpcm
smd true SMDsolvent "water"
surfacetype gepol_sas                               #Solvent accessible surface (SAS) used
end

* xyz 0 1
#Atomic coordinates go here
*
```

[1] Neese, F. The ORCA program system. Wiley Interdiscip. Rev.: Comput. Mol. Sci. **2012**, 2, 73–78.
[2] Neese, F. Software update: the ORCA program system, version 4.0. Wiley Interdiscip. Rev.: Comput. Mol. Sci. **2017**, 8, e1327.
[3] Hanwell, M. D.; Curtis, D. E.; Lonie, D. C.; Vandermeersch, T.; Zurek, E.; Hutchinson, G. R. Avogadro: an advanced semantic chemical editor, visualization, and analysis platform. *Journal of Cheminformatics* **2012**, 4:17.
[4] https://www.chemcraftprog.com
[5] Becke, A. D. A new mixing of Hartree–Fock and local density-functional theories. J. Chem. Phys. **1993**, 98, 5648–5653.
[6] Lee, C.; Yang, W.; Parr, R. G. Development of the Colle-Salvetti correlation-energy formula into a functional of the electron density. Phys. Rev. B **1988**, 37, 785–789.
[7] Grimme, S.; Ehrlich, S.; Goerigk, L. Effect of the damping function in dispersion corrected density functional theory. *J. Comput. Chem.* **2011**, 32, 1456–1465.
[8] Grimme, S.; Antony, J.; Ehrlich, S.; Krieg, H. A consistent and accurate ab initio parametrization of density functional dispersion correction (DFT-D) for the 94 elements H-Pu. *J. Chem. Phys.* **2010**, 132, 154104.
[9] Weigend, F.; Ahlrichs, R. Balanced basis sets of split valence, triple zeta valence and quadruple zeta valence quality for H to Rn: Design and assessment of accuracy. *Phys. Chem. Chem. Phys.* **2005**, 7, 3297–3305.
[10] Weigend, F. Accurate Coulomb-fitting basis sets for H to Rn. *Phys. Chem. Chem. Phys.* **2006**, 8, 1057–1065.
[11] Andraw, D.; Haeussermann, U.; Dolg, M.; Stoll, H.; Preuss, H. Energy-adjusted *ab initio* pseudopotentials for the second and third row transition elements. *Theor. Chim. Acta*, **1990**, 77, 123–141.
[12] Marenich, A. V.; Cramer, C. J.; Truhlar, D. G. Universal Solvation Model Based on Solute Electron Density and on a Continuum Model of the Solvent Defined by the Bulk Dielectric Constant and Atomic Surface Tensions. *J. Phys. Chem. B*, **2009**, 113, 6378–6396.

HCl
Level of theory: B3LYP D3BJ RIJCOSX def2/TZVP SMD H2O (SAS)

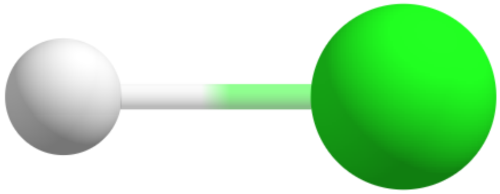

Electronic Energy (Hartree): -460.765563
Gibbs Free Energy (Hartree): -460.776775
Negative Frequencies (cm^{-1}): None
Molecular Dipole (Debye): 1.30
Coordinates (Charge 0, Multiplicity 1):

H	-7.49248	0.50985	0.00000
Cl	-6.21134	0.56348	0.00000

$HgCl_2$
Level of theory: B3LYP D3BJ RIJCOSX def2/TZVP SMD H2O (SAS)

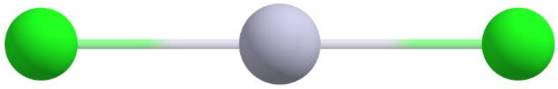

Electronic Energy (Hartree): -1073.868082
Gibbs Free Energy (Hartree): -1073.892515
Negative Frequencies (cm^{-1}): None
Molecular Dipole (Debye): 0.00
Coordinates (Charge 0, Multiplicity 1):

Cl	0.00000	0.00000	-2.28629
Hg	0.00000	0.00000	0.00000
Cl	0.00000	0.00000	2.28629

(*E*)-2-buten-1-ol
Level of theory: B3LYP D3BJ RIJCOSX def2/TZVP SMD H2O (SAS)

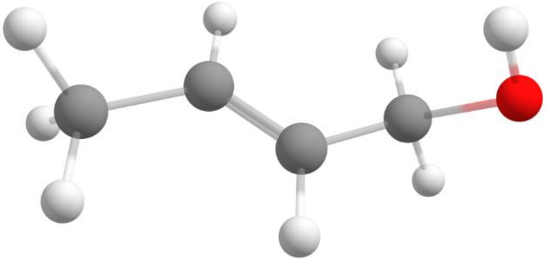

Electronic Energy (Hartree): -232.399713
Gibbs Free Energy (Hartree): -232.316197
Negative Frequencies (cm^{-1}): None
Molecular Dipole (Debye): 2.34
Coordinates (Charge 0, Multiplicity 1):

C	-9.88860	4.12688	-1.66922
C	-6.93634	2.54293	0.32562
O	-6.44475	1.25241	-0.05782
H	-7.20325	0.73302	-0.34847
C	-7.75553	3.17726	-0.74947
C	-9.03707	3.50351	-0.61326
H	-10.27177	5.09791	-1.34175
H	-9.33579	4.27501	-2.59777
H	-10.76273	3.50585	-1.88583
H	-6.04105	3.13296	0.53450
H	-7.51593	2.47686	1.25417
H	-7.24939	3.34820	-1.69655
H	-9.52066	3.31382	0.34299

(*R*)-3-Buten-2-ol
Level of theory: B3LYP D3BJ RIJCOSX def2/TZVP SMD H2O (SAS)

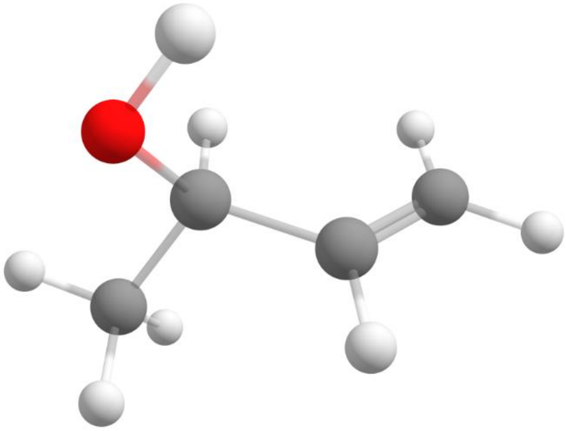

Electronic Energy (Hartree): -232.399350
Gibbs Free Energy (Hartree): -232.315705
Negative Frequencies (cm^{-1}): None
Molecular Dipole (Debye): 1.61
Coordinates (Charge 0, Multiplicity 1):

O	-7.66265	-1.01086	0.07424
C	-8.12187	-3.28977	-0.38280
C	-9.38108	-2.26548	2.80438
H	-7.65891	-0.32263	0.74910
C	-9.27950	-2.09474	1.49329
C	-8.00665	-2.24983	0.71643
H	-8.32827	-4.27284	0.04191
H	-8.92913	-3.03139	-1.07075
H	-7.19130	-3.34308	-0.94950
H	-10.32144	-2.13791	3.32560
H	-8.52382	-2.54586	3.40691
H	-10.14901	-1.80842	0.90710
H	-7.20320	-2.53965	1.40555

(*E*)-4-methylpent-2-en-1-ol
Level of theory: B3LYP D3BJ RIJCOSX def2/TZVP SMD H2O (SAS)

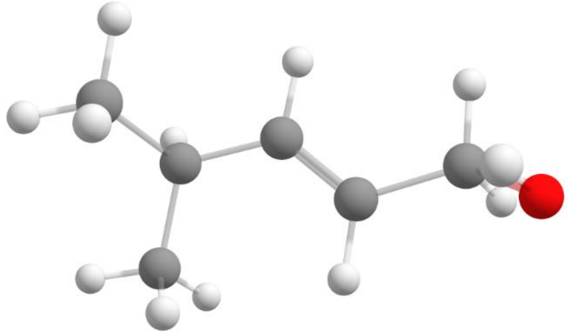

Electronic Energy (Hartree): -311.006746
Gibbs Free Energy (Hartree): -310.869951
Negative Frequencies (cm^{-1}): None
Molecular Dipole (Debye): 2.43
Coordinates (Charge 0, Multiplicity 1):

C	-9.02855	5.09646	-0.17725
C	-9.34797	1.47277	-1.63555
O	-8.98213	0.35682	-0.81368
H	-8.24284	0.63940	-0.26300
C	-9.59387	2.70713	-0.83108
C	-8.88041	3.82168	-0.95920
C	-10.16848	5.07931	0.83572
C	-7.69612	5.44809	0.49901
H	-9.23687	5.89073	-0.90630
H	-10.25672	1.15444	-2.15146
H	-8.58081	1.66130	-2.39597
H	-10.39085	2.63845	-0.09778
H	-8.08501	3.83565	-1.70293
H	-10.24225	6.04441	1.33992
H	-11.13017	4.87867	0.36054
H	-10.00543	4.31859	1.60295
H	-7.75554	6.41915	0.99446
H	-6.87963	5.49101	-0.22465
H	-7.43701	4.69931	1.25138

(*R*)-4-methylpent-1-en-3-ol
Level of theory: B3LYP D3BJ RIJCOSX def2/TZVP SMD H2O (SAS)

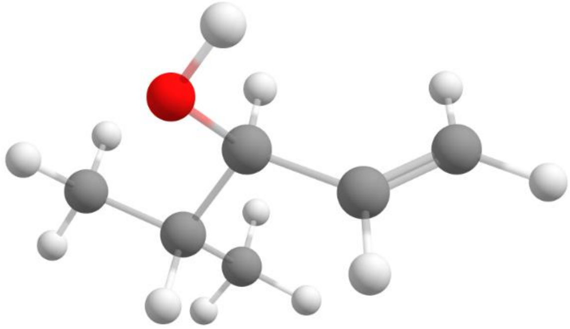

Electronic Energy (Hartree): -311.006503
Gibbs Free Energy (Hartree): -310.869977
Negative Frequencies (cm^{-1}): None
Molecular Dipole (Debye): 1.53
Coordinates (Charge 0, Multiplicity 1):

O	-5.70623	-0.28706	0.36927
C	-6.78241	-2.42326	0.31385
C	-4.00264	-2.27956	2.59899
H	-4.93498	0.19937	0.68033
C	-5.15118	-1.79784	2.14392
C	-5.50143	-1.67268	0.69258
C	-7.12123	-2.21437	-1.16097
C	-6.65992	-3.91101	0.63894
H	-7.58959	-1.99594	0.91955
H	-3.78723	-2.33700	3.65855
H	-3.23376	-2.64108	1.92492
H	-5.90771	-1.43803	2.83660
H	-4.67462	-2.07219	0.08837
H	-8.05436	-2.72101	-1.41607
H	-7.22900	-1.15766	-1.39984
H	-6.33545	-2.62699	-1.80058
H	-7.57391	-4.43865	0.36071
H	-6.48010	-4.08602	1.70001
H	-5.83447	-4.36567	0.08391

Methyl substrate – Neutral SM (1)
Level of theory: B3LYP D3BJ RIJCOSX def2/TZVP SMD H2O (SAS)

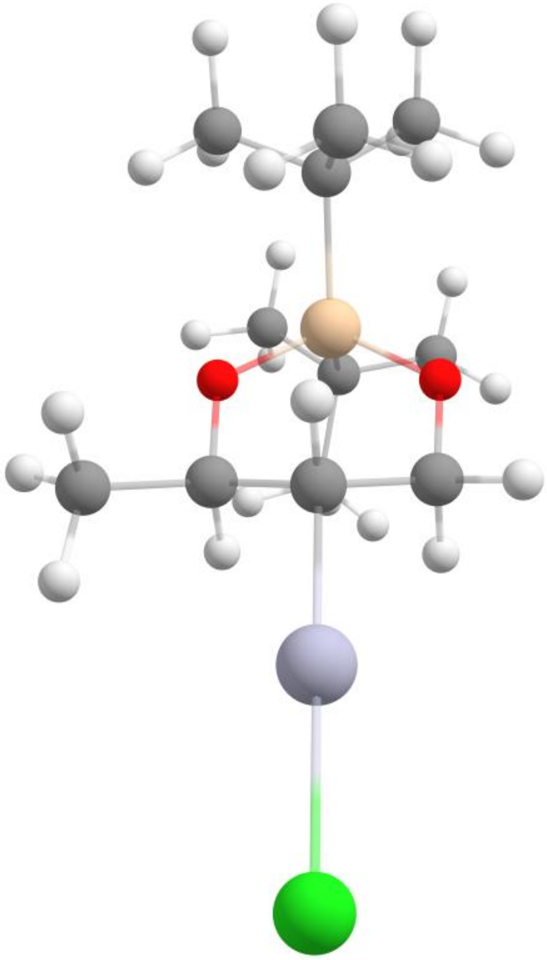

Electronic Energy (Hartree): -1525.935219
Gibbs Free Energy (Hartree): -1525.628521
Negative Frequencies (cm^{-1}): None
Molecular Dipole (Debye): 3.04
Coordinates (Charge 0, Multiplicity 1):

Hg	-0.03506	-1.37049	-2.53174
Cl	-0.58460	-1.87400	-4.75003
Si	0.97942	0.31910	2.18189
O	1.92306	0.49527	0.83047
O	-0.35843	-0.53638	1.70733
C	-0.75569	-0.72150	0.34395
C	0.48561	-0.95193	-0.52187
C	1.45933	0.22159	-0.48707
C	0.45840	2.05181	2.75007
C	1.90367	-0.77783	3.41688
C	-1.74528	-1.87129	0.32292
H	0.99357	-1.85346	-0.17058
H	2.33949	0.00862	-1.09629
H	0.98661	1.12210	-0.89559
H	-1.25959	0.19263	0.00633
C	2.42229	-2.00161	2.64213
C	0.95697	-1.27070	4.52304
C	3.09720	-0.04713	4.04761
C	-0.50356	2.60591	1.68365
C	1.67327	2.98806	2.84011
C	-0.26997	2.02325	4.10044
H	3.11459	-1.71751	1.84838
H	1.60479	-2.57001	2.19390
H	2.95257	-2.67484	3.32377
H	3.77843	0.35322	3.29349
H	3.67175	-0.73839	4.67352
H	2.78085	0.77772	4.68793
H	0.08493	-1.77945	4.10880
H	0.60012	-0.45945	5.15808
H	1.48077	-1.98246	5.17022
H	-0.03667	2.65808	0.69744
H	-0.80703	3.62421	1.94882
H	-1.41075	2.00496	1.60177
H	-1.11439	1.33038	4.09747
H	-0.66385	3.01798	4.33593
H	0.39524	1.74038	4.91763
H	2.22115	3.02854	1.89720
H	2.37428	2.68555	3.61870
H	1.34387	4.00544	3.07778
H	-2.22337	-1.97540	-0.65334
H	-2.52707	-1.69394	1.06123
H	-1.24846	-2.81113	0.57128

Methyl Substrate – Internal Alkene Product (26)

Level of theory: B3LYP D3BJ RIJCOSX def2/TZVP SMD H2O (SAS)

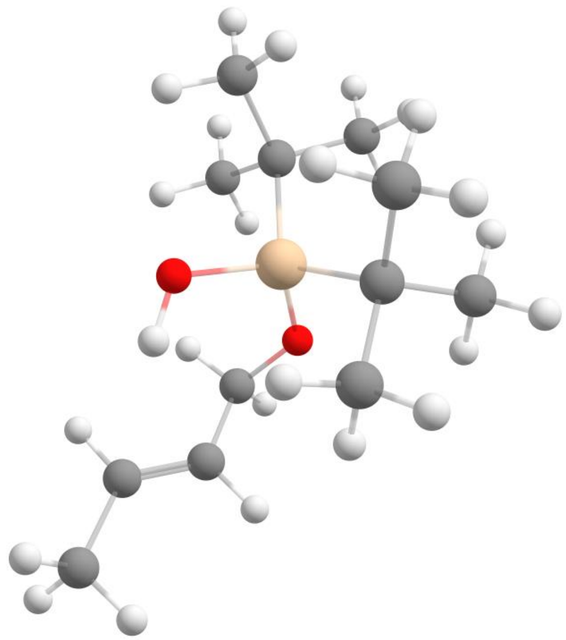

Electronic Energy (Hartree): -912.848375

Gibbs Free Energy (Hartree): -912.531882

Negative Frequencies (cm^{-1}): None

Molecular Dipole (Debye): 2.17

Coordinates (Charge 0, Multiplicity 1):

O	-8.83096	0.19218	-0.30482
C	-9.81970	4.24897	-1.75372
C	-6.98953	2.59210	0.36769
O	-6.39953	1.37176	-0.07325
Si	-7.19316	-0.07286	-0.25305
C	-6.57496	-0.75385	-1.91287
C	-6.94260	-1.14186	1.29157
C	-7.75231	3.25725	-0.73499
C	-9.04240	3.57327	-0.67181
C	-7.18317	0.12980	-3.01644
C	-7.02991	-2.20219	-2.13699
C	-7.22229	-0.25029	2.51468
C	-7.92371	-2.32375	1.31487
C	-5.50321	-1.66599	1.38656
C	-5.04456	-0.67264	-2.01445
H	-9.10823	0.96059	-0.81453
H	-10.21284	5.21053	-1.41106
H	-9.20706	4.42806	-2.63806
H	-10.68529	3.65020	-2.05163
H	-6.16595	3.23176	0.69727
H	-7.64258	2.42716	1.22972
H	-7.18473	3.47643	-1.63573
H	-9.58870	3.33942	0.24001
H	-6.82157	-0.19484	-3.99789
H	-6.90597	1.17904	-2.89682
H	-8.27329	0.06717	-3.03550
H	-6.76060	-2.52925	-3.14731
H	-6.55520	-2.89361	-1.43900
H	-8.11189	-2.31215	-2.03609
H	-7.12011	-0.83656	3.43434
H	-6.51971	0.58262	2.57811
H	-8.23520	0.15681	2.49676
H	-7.81112	-2.88356	2.25007
H	-7.75023	-3.02344	0.49651
H	-8.95924	-1.98699	1.25092
H	-5.27375	-2.38239	0.59616
H	-4.76910	-0.85897	1.33305
H	-5.35370	-2.18141	2.34166
H	-4.68670	0.34623	-1.86105
H	-4.54623	-1.31351	-1.28610
H	-4.71762	-0.99644	-3.00893

Methyl Substrate – Terminal Alkene Product (27)

Level of theory: B3LYP D3BJ RIJCOSX def2/TZVP SMD H2O (SAS)

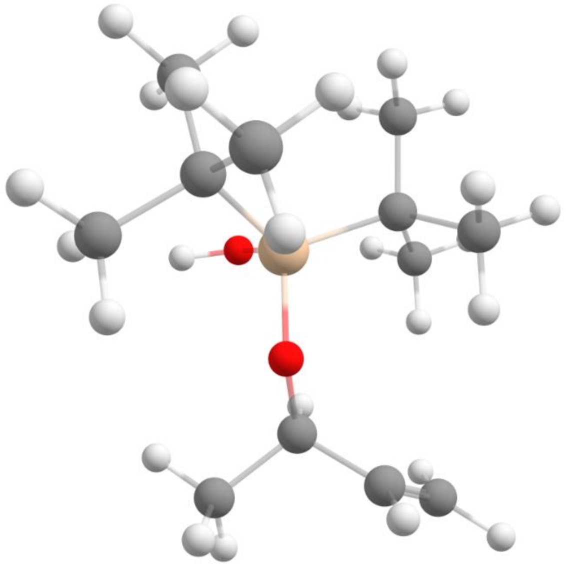

Electronic Energy (Hartree): -912.846245

Gibbs Free Energy (Hartree): -912.530497

Negative Frequencies (cm^{-1}): None

Molecular Dipole (Debye): 1.17

Coordinates (Charge 0, Multiplicity 1):

O	-7.89236	-0.93656	0.07691
C	-8.11966	-3.28644	-0.30692
C	-9.53000	-2.43674	2.78097
O	-5.23801	-0.64139	0.59642
Si	-6.66935	0.14899	0.29796
C	-6.97202	1.17538	1.86411
C	-6.56430	1.04349	-1.37472
C	-9.38982	-2.11527	1.50356
C	-8.09971	-2.18361	0.74264
C	-6.68656	0.28912	3.09026
C	-8.43531	1.63911	1.93281
C	-6.49132	-0.03909	-2.46716
C	-7.80682	1.90941	-1.62534
C	-5.30389	1.91582	-1.47381
C	-6.04751	2.40001	1.92155
H	-8.27289	-4.26060	0.15998
H	-8.92407	-3.11328	-1.02413
H	-7.17400	-3.30554	-0.85100
H	-10.49021	-2.38641	3.27853
H	-8.68941	-2.76994	3.37956
H	-4.83049	-1.10159	-0.14141
H	-10.24433	-1.77824	0.92346
H	-7.27895	-2.37419	1.44181
H	-6.84481	0.86882	4.00640
H	-5.66017	-0.07818	3.09802
H	-7.35691	-0.57023	3.13667
H	-8.60592	2.19349	2.86223
H	-8.70170	2.30139	1.10775
H	-9.12617	0.79536	1.92011
H	-6.41679	0.43043	-3.45362
H	-5.61151	-0.68063	-2.35723
H	-7.37191	-0.68112	-2.46179
H	-7.77908	2.32519	-2.63864
H	-7.86237	2.75228	-0.93435
H	-8.72968	1.33413	-1.53059
H	-5.31967	2.75190	-0.77517
H	-4.39371	1.34403	-1.28106
H	-5.21917	2.33631	-2.48169
H	-6.28269	3.12784	1.14357
H	-6.16453	2.91023	2.88387
H	-4.99527	2.12617	1.82180

Isopropyl Substrate – Neutral SM (4)
Level of theory: B3LYP D3BJ RIJCOSX def2/TZVP SMD H2O (SAS)

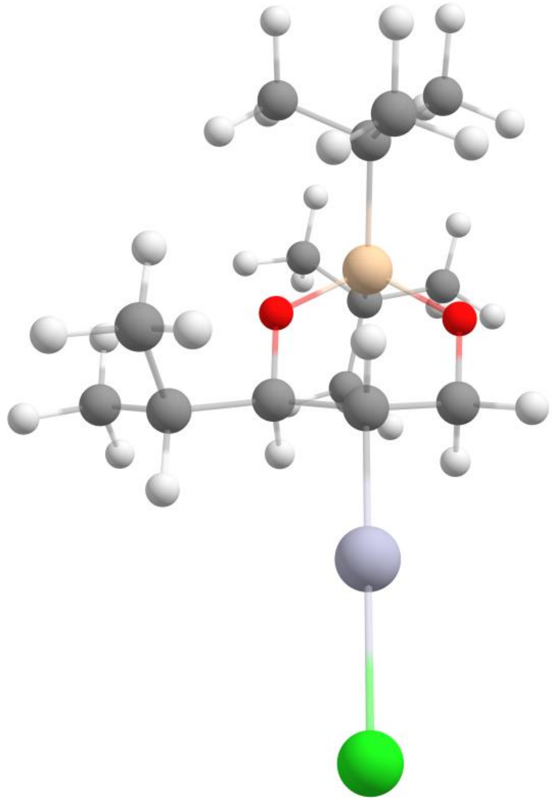

Electronic Energy (Hartree): -1604.544340
Gibbs Free Energy (Hartree): -1604.183764
Negative Frequencies (cm^{-1}): None
Molecular Dipole (Debye): 3.22
Coordinates (Charge 0, Multiplicity 1):

Hg	-0.05238	-1.42501	-2.54001
Cl	-0.60992	-1.96279	-4.74876
Si	0.96287	0.31561	2.15115
O	1.90114	0.49601	0.79683
O	-0.38022	-0.52865	1.67707
C	-0.77568	-0.70725	0.31404
C	0.46737	-0.97017	-0.53808
C	1.44122	0.20383	-0.51880
C	0.44661	2.04533	2.73038
C	1.88174	-0.79419	3.37920
C	-1.85176	-1.80113	0.27893
C	-1.32142	-3.16654	0.71042
C	-3.05119	-1.38595	1.12860
H	0.97991	-1.86011	-0.17110
H	2.32342	-0.01844	-1.12165
H	0.97036	1.09913	-0.94047
H	-2.19240	-1.87232	-0.76156
H	-2.12093	-3.90872	0.68908
H	-0.52486	-3.53192	0.05981
H	-0.93271	-3.12283	1.72869
H	-3.44367	-0.41574	0.81552
H	-2.77034	-1.31089	2.17948
H	-3.85766	-2.11594	1.04217
H	-1.23999	0.22452	-0.03444
C	2.40943	-2.00821	2.59534
C	0.92668	-1.30104	4.47168
C	3.06833	-0.06840	4.02814
C	-0.51351	2.60592	1.66582
C	1.66200	2.97958	2.82842
C	-0.28449	2.00666	4.07881
H	3.10799	-1.71383	1.81093
H	1.59834	-2.57425	2.13311
H	2.93528	-2.68768	3.27420
H	3.75376	0.34374	3.28427
H	3.64072	-0.76586	4.64908
H	2.74474	0.74712	4.67664
H	0.06229	-1.81260	4.04490
H	0.55655	-0.49706	5.10828
H	1.44757	-2.01409	5.11969
H	-0.04549	2.66183	0.68040
H	-0.81611	3.62319	1.93572
H	-1.42099	2.00613	1.58025
H	-1.13069	1.31610	4.06736
H	-0.67663	3.00018	4.32209
H	0.37790	1.71488	4.89508
H	2.21262	3.02381	1.88724
H	2.36022	2.67262	3.60769
H	1.33326	3.99632	3.06959

Isopropyl Substrate – Internal Alkene Product (32)
Level of theory: B3LYP D3BJ RIJCOSX def2/TZVP SMD H2O (SAS)

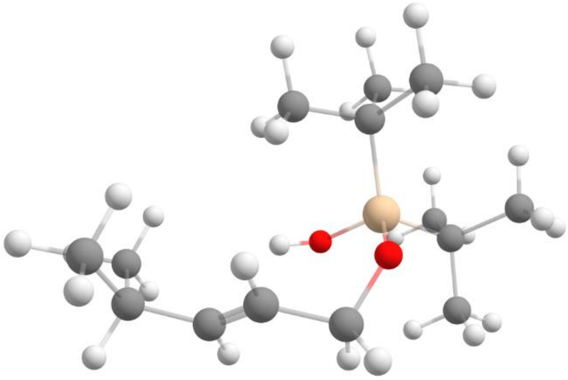

Electronic Energy (Hartree): -991.456758
Gibbs Free Energy (Hartree): -991.086458
Negative Frequencies (cm^{-1}): None
Molecular Dipole (Debye): 2.34
Coordinates (Charge 0, Multiplicity 1):

O	-7.06922	0.74742	-1.05139
C	-8.59109	4.69287	-0.10669
C	-10.02454	1.41329	-1.71399
O	-9.73261	0.19082	-1.03958
Si	-8.21580	-0.41504	-0.75224
C	-8.21715	-0.88697	1.08682
C	-7.83948	-1.77308	-2.01797
C	-9.81669	2.58101	-0.80076
C	-8.92229	3.54073	-1.01390
C	-6.98180	-1.71667	1.46138
C	-9.48970	-1.66196	1.45945
C	-8.68782	-3.02660	-1.76383
C	-8.19639	-1.20934	-3.40509
C	-6.35019	-2.14840	-2.01157
C	-8.19187	0.42317	1.89402
C	-9.48908	4.79843	1.12113
C	-7.11308	4.60552	0.30246
H	-7.25068	1.61696	-0.68049
H	-8.70909	5.61090	-0.69586
H	-11.06949	1.35148	-2.02924
H	-9.41248	1.52209	-2.61414
H	-10.42651	2.58245	0.09625
H	-8.32814	3.49259	-1.92557
H	-6.94925	-1.87540	2.54492
H	-6.05193	-1.21953	1.17655
H	-6.99300	-2.70224	0.99337
H	-10.38917	-1.09658	1.21273
H	-9.55233	-2.62446	0.95004
H	-9.50438	-1.86294	2.53652
H	-8.53817	-3.75182	-2.57117
H	-8.41621	-3.52405	-0.83129
H	-9.75504	-2.79743	-1.72494
H	-7.97369	-1.95264	-4.17827
H	-7.62043	-0.31148	-3.63781
H	-9.25708	-0.96371	-3.48203
H	-5.71628	-1.27820	-2.18728
H	-6.04023	-2.60120	-1.06911
H	-6.14532	-2.87669	-2.80418
H	-7.27407	0.99066	1.72418
H	-9.03811	1.06905	1.65222
H	-8.24289	0.20364	2.96570
H	-9.21853	5.67304	1.71491
H	-10.54078	4.89495	0.84672
H	-9.38633	3.92119	1.76443
H	-6.81371	5.48363	0.87760
H	-6.45760	4.53873	-0.56827
H	-6.93837	3.72472	0.92662

Isopropyl Substrate – Terminal Alkene Product (33)

Level of theory: B3LYP D3BJ RIJCOSX def2/TZVP SMD H2O (SAS)

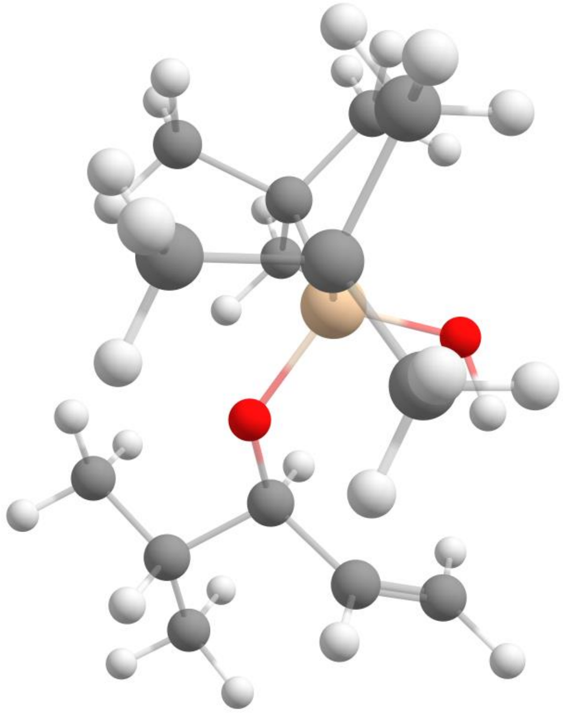

Electronic Energy (Hartree): -991.457734

Gibbs Free Energy (Hartree): -991.087273

Negative Frequencies (cm^{-1}): None

Molecular Dipole (Debye): 1.87

Coordinates (Charge 0, Multiplicity 1):

O	-5.69151	-0.29578	0.45786
C	-6.79604	-2.42769	0.34661
C	-4.00479	-2.43576	2.57893
O	-3.11643	0.38334	0.99498
Si	-4.53265	0.87561	0.28669
C	-5.26323	2.36654	1.20619
C	-4.07920	1.10299	-1.53971
C	-5.12678	-1.87048	2.15285
C	-5.50043	-1.68773	0.70952
C	-5.23749	2.02461	2.70638
C	-6.72061	2.61734	0.79087
C	-3.79326	-0.29131	-2.12497
C	-5.22592	1.74306	-2.33348
C	-2.81069	1.95767	-1.68758
C	-4.43806	3.63997	0.97866
C	-7.19293	-2.15847	-1.10271
C	-6.66246	-3.92731	0.60512
H	-7.57914	-2.02463	0.99855
H	-3.78794	-2.54543	3.63411
H	-3.26793	-2.82482	1.88481
H	-3.20982	-0.14231	1.79669
H	-5.85163	-1.48933	2.86694
H	-4.69025	-2.07513	0.08006
H	-5.68273	2.84144	3.28430
H	-4.21983	1.88143	3.07548
H	-5.80846	1.12039	2.92642
H	-7.14561	3.43087	1.38938
H	-6.80935	2.90684	-0.25697
H	-7.33860	1.73192	0.94469
H	-3.47147	-0.19761	-3.16791
H	-2.99755	-0.80447	-1.58153
H	-4.67981	-0.92598	-2.11495
H	-4.97883	1.76336	-3.40058
H	-5.41213	2.77373	-2.02760
H	-6.15970	1.18657	-2.22624
H	-2.95440	2.98040	-1.33785
H	-1.97312	1.53078	-1.13428
H	-2.52057	2.01320	-2.74274
H	-4.49848	3.98842	-0.05358
H	-4.81362	4.44984	1.61377
H	-3.38367	3.49512	1.22415
H	-8.14723	-2.63626	-1.33302
H	-7.29248	-1.09168	-1.29644
H	-6.44843	-2.56456	-1.79315
H	-7.58521	-4.44477	0.33737
H	-6.44628	-4.14718	1.65077
H	-5.85710	-4.35708	0.00274

Isopropyl Substrate – Protonated SM (towards terminal alkene product) (59)
Level of theory: B3LYP D3BJ RIJCOSX def2/TZVP SMD H2O (SAS)

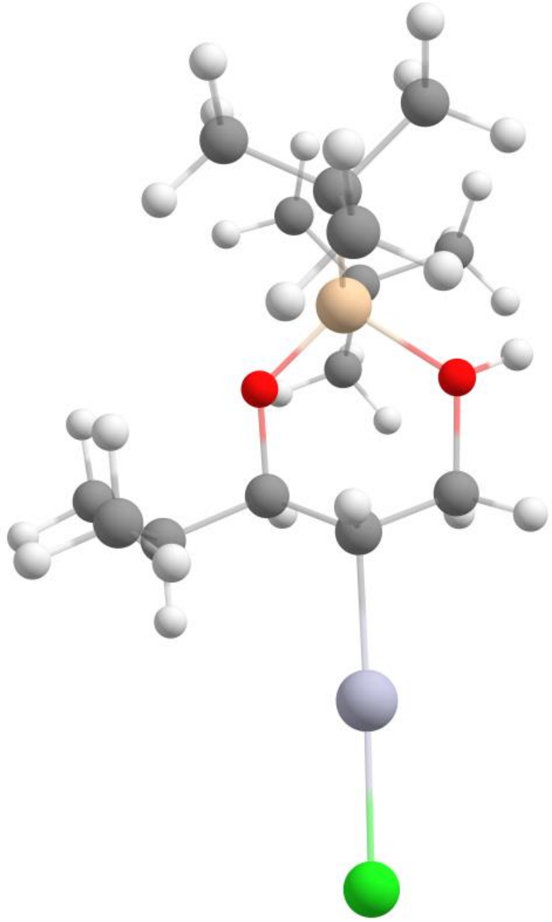

Electronic Energy (Hartree): -1604.931457
Gibbs Free Energy (Hartree): -1604.559848
Negative Frequencies (cm^{-1}): None
Molecular Dipole (Debye): 12.46
Coordinates (Charge +1, Multiplicity 1):

Hg	0.01496	-1.41474	-2.58982
Cl	-0.55616	-1.98933	-4.74852
Si	0.88087	0.29838	2.30006
O	1.93248	0.51543	0.82498
O	-0.31956	-0.56253	1.64135
C	-0.72804	-0.70517	0.26667
C	0.52111	-0.94914	-0.57611
C	1.45159	0.22322	-0.59799
C	0.42135	2.06374	2.74211
C	1.87369	-0.78920	3.45316
C	-1.79503	-1.80223	0.20947
C	-1.25659	-3.17688	0.59902
C	-2.99585	-1.41316	1.06888
H	1.05052	-1.83177	-0.21803
H	2.37171	0.03859	-1.14263
H	0.98315	1.14610	-0.93168
H	-2.13560	-1.84093	-0.83250
H	-2.05197	-3.92008	0.54352
H	-0.45329	-3.52047	-0.05516
H	-0.88140	-3.17145	1.62314
H	-3.38487	-0.43110	0.79214
H	-2.72687	-1.38626	2.12511
H	-3.80218	-2.13640	0.94618
H	-1.19040	0.23714	-0.04408
H	2.55226	1.26128	0.83522
C	-0.61103	2.56772	1.71828
C	1.65024	2.98899	2.71919
C	-0.20956	2.08729	4.14596
C	2.39887	-1.99669	2.65632
C	0.92716	-1.29887	4.55833
C	3.05443	-0.03581	4.08306
H	-0.21765	2.58532	0.69893
H	-0.89529	3.59458	1.96122
H	-1.52018	1.96584	1.72401
H	-1.06526	1.41404	4.22688
H	-0.56996	3.09573	4.36481
H	0.50676	1.82427	4.92462
H	2.07076	3.10762	1.71521
H	2.44563	2.66141	3.38852
H	1.35700	3.99304	3.03560
H	0.07299	-1.83910	4.14712
H	0.54856	-0.49531	5.19014
H	1.47224	-1.99093	5.20553
H	3.74609	0.35955	3.33533
H	3.62790	-0.72024	4.71339
H	2.73009	0.78883	4.71836
H	3.10425	-1.70636	1.87641
H	1.59003	-2.56685	2.19737
H	2.92710	-2.67184	3.33409

Isopropyl Substrate – Mercuronium Rearrangement Transition State (towards terminal alkene product) (60)
Level of theory: B3LYP D3BJ RIJCOSX def2/TZVP SMD H2O (SAS)

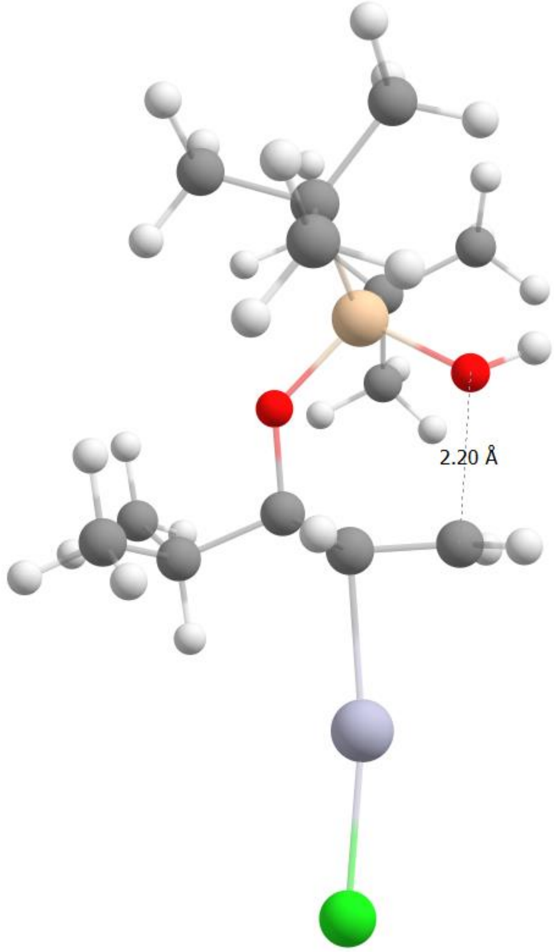

Electronic Energy (Hartree): -1604.918103
Gibbs Free Energy (Hartree): -1604.550816
Negative Frequencies (cm^{-1}): -181.51
Molecular Dipole (Debye): 5.64
Coordinates (Charge +1, Multiplicity 1):

Hg	0.04839	-1.42500	-2.96579
Cl	-0.63072	-2.03164	-5.06076
Si	0.80430	0.29977	2.07075
O	1.92849	0.63402	0.83999
O	-0.33996	-0.62466	1.30955
C	-0.74975	-0.69859	-0.03982
C	0.50731	-1.07416	-0.81998
C	1.38687	-0.05593	-1.17905
C	0.08650	1.95125	2.63438
C	1.61111	-0.85656	3.31552
C	-1.91069	-1.68966	-0.16270
C	-1.52808	-3.10395	0.26718
C	-3.11610	-1.17505	0.61988
H	0.96347	-2.01671	-0.52972
H	2.41438	-0.26612	-1.44338
H	1.02557	0.94559	-1.36609
H	-2.18785	-1.70673	-1.22690
H	-2.38636	-3.76890	0.17360
H	-0.72808	-3.53322	-0.33960
H	-1.20651	-3.11797	1.30873
H	-3.41067	-0.17621	0.29228
H	-2.89378	-1.13338	1.68645
H	-3.97170	-1.83567	0.48022
H	-1.09223	0.28345	-0.38469
H	2.44765	1.44253	0.90516
C	-0.85293	2.46921	1.53099
C	1.20700	2.98140	2.85786
C	-0.71860	1.78928	3.93293
C	2.29070	-1.98859	2.52321
C	0.55769	-1.47718	4.24818
C	2.67013	-0.11603	4.14592
H	-0.34366	2.58790	0.57109
H	-1.24090	3.45406	1.80582
H	-1.71169	1.81174	1.38759
H	-1.49736	1.02924	3.84419
H	-1.21287	2.73327	4.18039
H	-0.08343	1.52662	4.77931
H	1.74110	3.22391	1.93469
H	1.93966	2.65636	3.59754
H	0.77913	3.92118	3.21806
H	-0.22495	-1.99363	3.69018
H	0.08007	-0.73802	4.89062
H	1.03566	-2.21361	4.90103
H	3.43557	0.35028	3.52108
H	3.18275	-0.82069	4.80709
H	2.23170	0.65645	4.77942
H	3.05289	-1.61489	1.83790
H	1.56661	-2.57161	1.95040
H	2.78164	-2.67780	3.21595

Isopropyl Substrate – Protonated SM (towards internal alkene product)
Level of theory: B3LYP D3BJ RIJCOSX def2/TZVP SMD H2O (SAS)

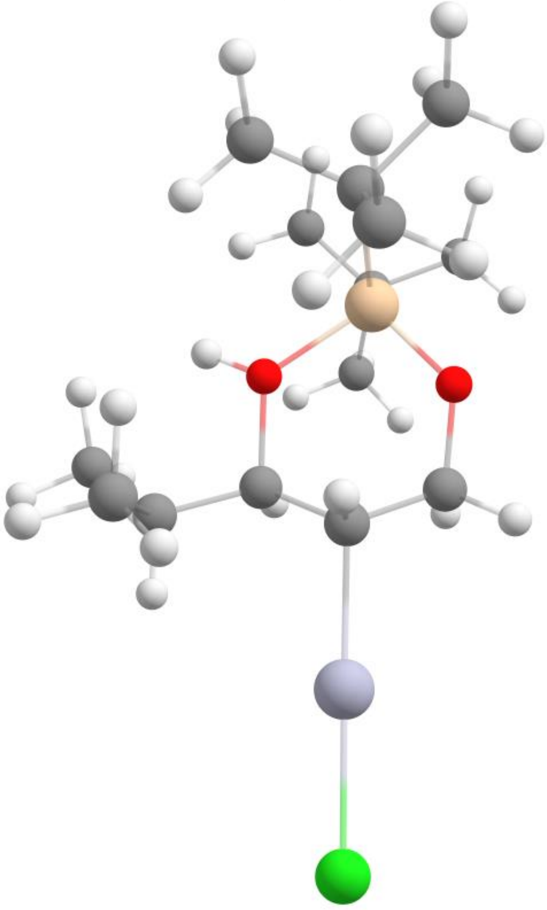

Electronic Energy (Hartree): -1604.931954
Gibbs Free Energy (Hartree): -1604.560381
Negative Frequencies (cm^{-1}): None
Molecular Dipole (Debye): 11.63
Coordinates (Charge +1, Multiplicity 1):

Hg	-0.08159	-1.43216	-2.57997
Cl	-0.58994	-1.95251	-4.76842
Si	1.09563	0.38871	2.21053
O	1.87546	0.43268	0.79053
O	-0.37900	-0.52038	1.69210
C	-0.82727	-0.73471	0.22956
C	0.42760	-0.98204	-0.56438
C	1.39534	0.19117	-0.53452
C	0.47565	2.07717	2.73852
C	1.90581	-0.78991	3.42231
C	-1.88914	-1.82893	0.27925
C	-1.32946	-3.21203	0.60274
C	-3.02747	-1.44580	1.22891
H	0.93328	-1.87648	-0.20114
H	2.27297	-0.01507	-1.14406
H	0.92753	1.10291	-0.91407
H	-2.31199	-1.84619	-0.73039
H	-2.13772	-3.94196	0.62563
H	-0.60667	-3.55549	-0.13682
H	-0.84565	-3.23129	1.58114
H	-3.39281	-0.43290	1.05050
H	-2.74553	-1.53961	2.28527
H	-3.86877	-2.12474	1.09972
H	-1.27828	0.22057	-0.03661
H	-1.14353	-0.57796	2.28734
C	2.46343	-1.98562	2.63091
C	0.88573	-1.30601	4.45283
C	3.05839	-0.07684	4.15106
C	-0.45903	2.62321	1.64395
C	1.69228	3.01546	2.86091
C	-0.27952	2.03014	4.07457
H	3.21197	-1.68147	1.89979
H	1.67821	-2.53406	2.10596
H	2.93603	-2.68829	3.32180
H	3.79318	0.33809	3.45840
H	3.58253	-0.79351	4.78867
H	2.70356	0.72805	4.79571
H	0.07958	-1.87931	3.98812
H	0.44128	-0.51039	5.05011
H	1.38644	-1.98584	5.14675
H	0.02543	2.66762	0.66713
H	-0.75810	3.64324	1.89723
H	-1.37932	2.04267	1.54955
H	-1.13555	1.34960	4.05537
H	-0.67448	3.02256	4.30664
H	0.36465	1.73781	4.90362
H	2.25338	3.08011	1.92741
H	2.38021	2.70613	3.64788
H	1.34778	4.02294	3.10859

Isopropyl Substrate – Mercuronium Rearrangement Transition State (towards internal alkene product)
Level of theory: B3LYP D3BJ RIJCOSX def2/TZVP SMD H2O (SAS)

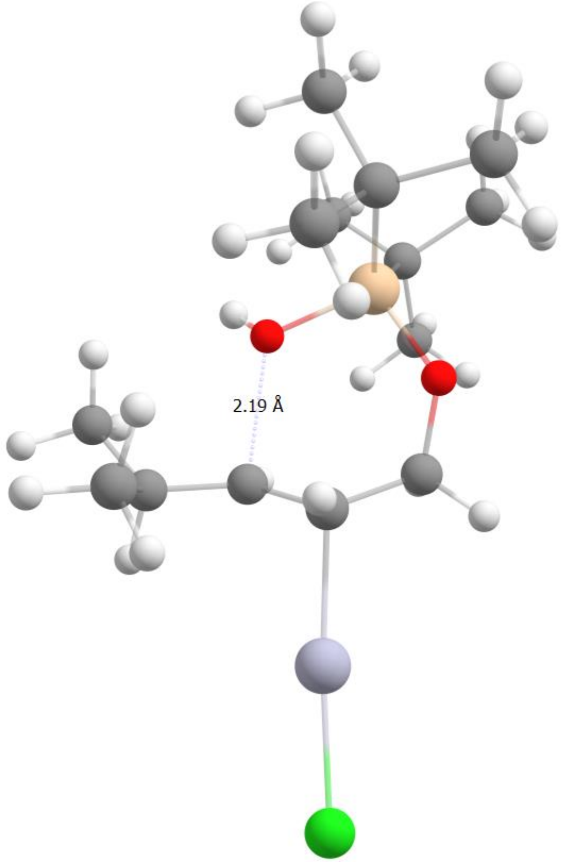

Electronic Energy (Hartree): -1604.923855
Gibbs Free Energy (Hartree): -1604.554830
Negative Frequencies (cm^{-1}): -77.07
Molecular Dipole (Debye): 6.93
Coordinates (Charge +1, Multiplicity 1):

Hg	-0.22550	-1.25925	-2.98588
Cl	-0.47768	-1.59836	-5.23539
Si	0.86083	0.45254	2.01596
O	1.62401	0.36203	0.55379
O	-0.62901	-0.31074	1.69664
C	-1.12626	-0.89803	-0.35575
C	0.20031	-0.94494	-0.86727
C	1.05777	0.31366	-0.73556
C	0.46356	2.23276	2.50107
C	1.85894	-0.63532	3.18155
C	-1.99624	-2.07629	-0.09843
C	-1.25015	-3.38851	0.11575
C	-3.05044	-1.81674	0.98602
H	0.74765	-1.84950	-0.61067
H	1.88804	0.29305	-1.44001
H	0.45986	1.20459	-0.94752
H	-2.56683	-2.14457	-1.04057
H	-1.96465	-4.19275	0.28357
H	-0.64261	-3.67471	-0.74372
H	-0.60134	-3.32752	0.98990
H	-3.51345	-0.83464	0.87994
H	-2.60974	-1.90257	1.97801
H	-3.84029	-2.56174	0.91010
H	-1.63668	0.05730	-0.41911
H	-1.39843	0.00885	2.17994
C	1.72277	-2.09052	2.69856
C	1.33772	-0.53940	4.62326
C	3.34425	-0.23732	3.13830
C	-0.03226	2.97867	1.24949
C	1.71683	2.94427	3.03476
C	-0.64072	2.27527	3.57095
H	-1.59084	1.86877	3.21055
H	-0.84304	3.31280	3.85144
H	-0.37013	1.74066	4.48151
H	0.74261	3.05130	0.48516
H	-0.32014	3.99934	1.51653
H	-0.91398	2.50954	0.80493
H	2.54432	2.90747	2.32358
H	2.06145	2.51952	3.97801
H	1.49231	3.99911	3.21817
H	2.06912	-2.21466	1.67058
H	0.69360	-2.44581	2.76466
H	2.33480	-2.74445	3.32594
H	3.75076	-0.29948	2.12811
H	3.92288	-0.91672	3.77125
H	3.51585	0.77370	3.50828
H	0.27781	-0.79322	4.70018
H	1.48072	0.45420	5.04945
H	1.88171	-1.24315	5.25986

VIII. NMR Spectra

Compound 5 (^{1}H NMR: 400 MHz, ^{13}C NMR: 100 MHz)

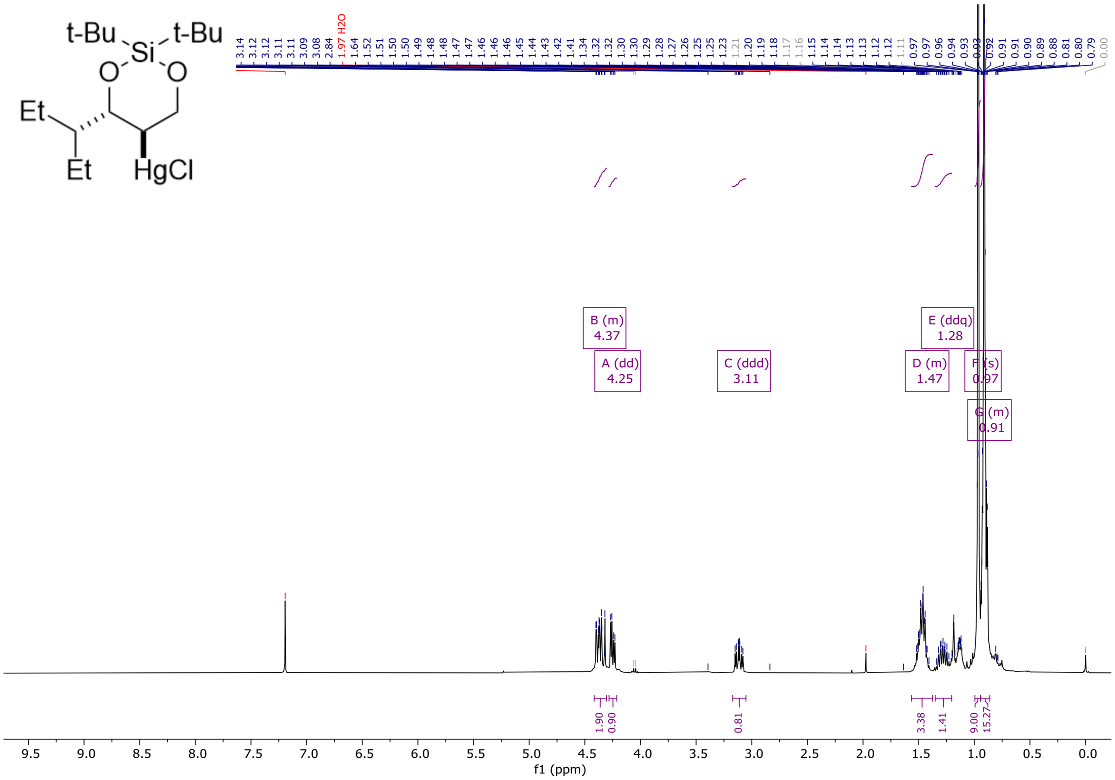

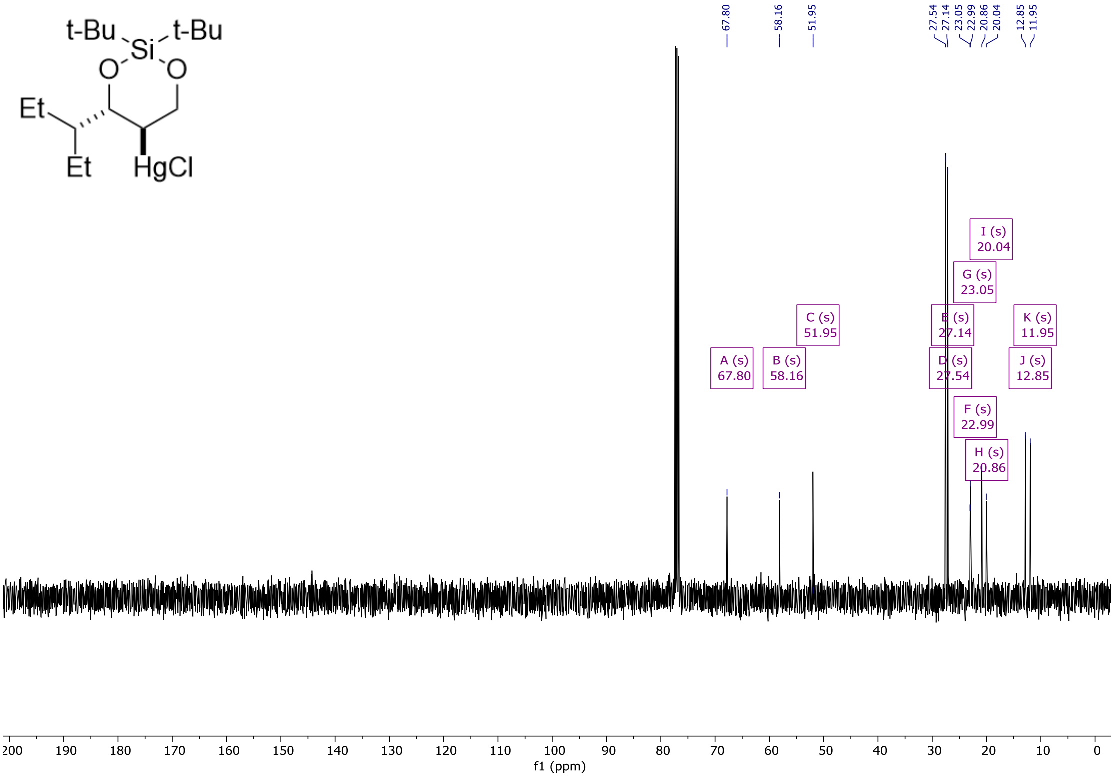

Compound 6 (^{1}H NMR: 400 MHz, ^{13}C NMR: 100 MHz)

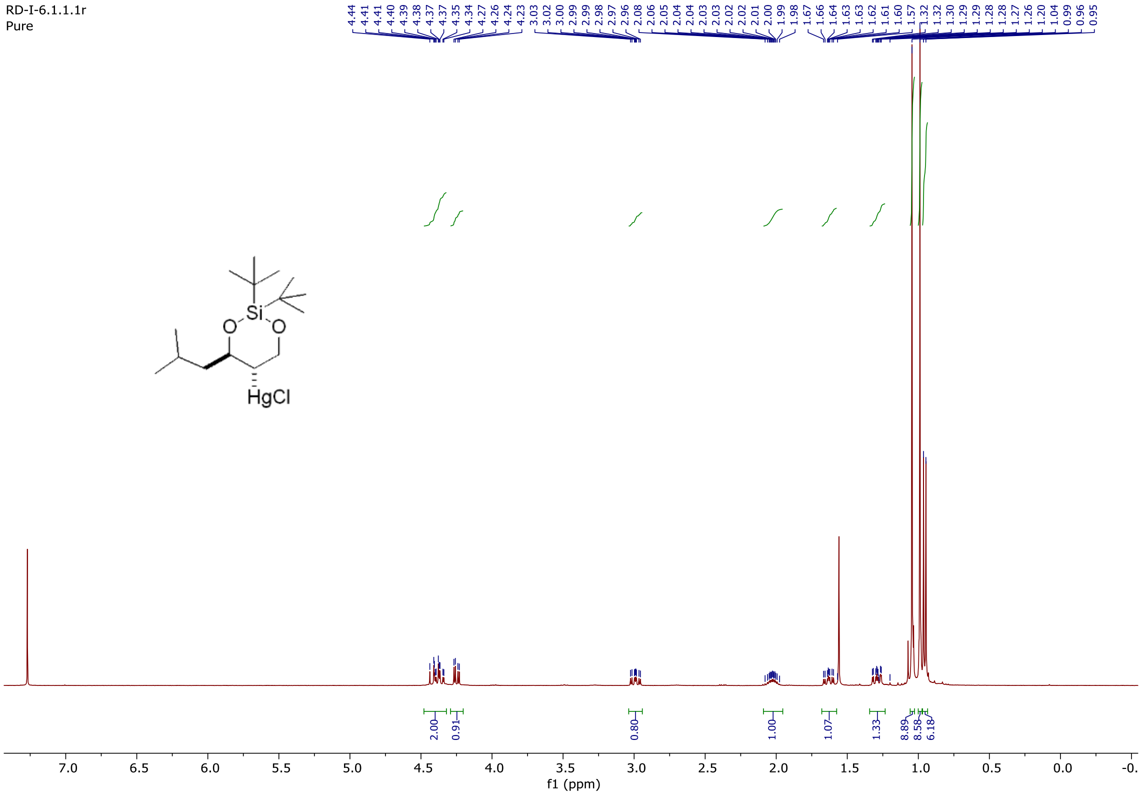

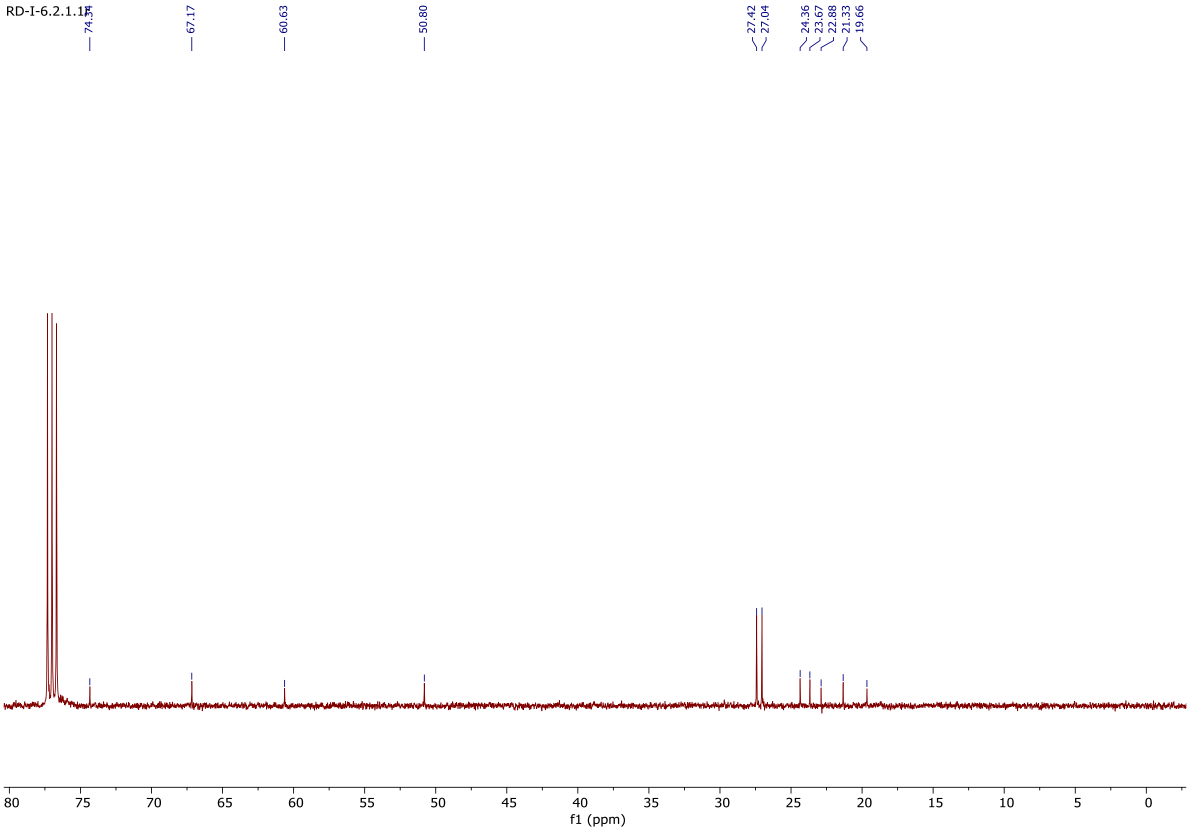

Compound 8 (^{1}H NMR: 400 MHz, ^{13}C NMR: 100 MHz)

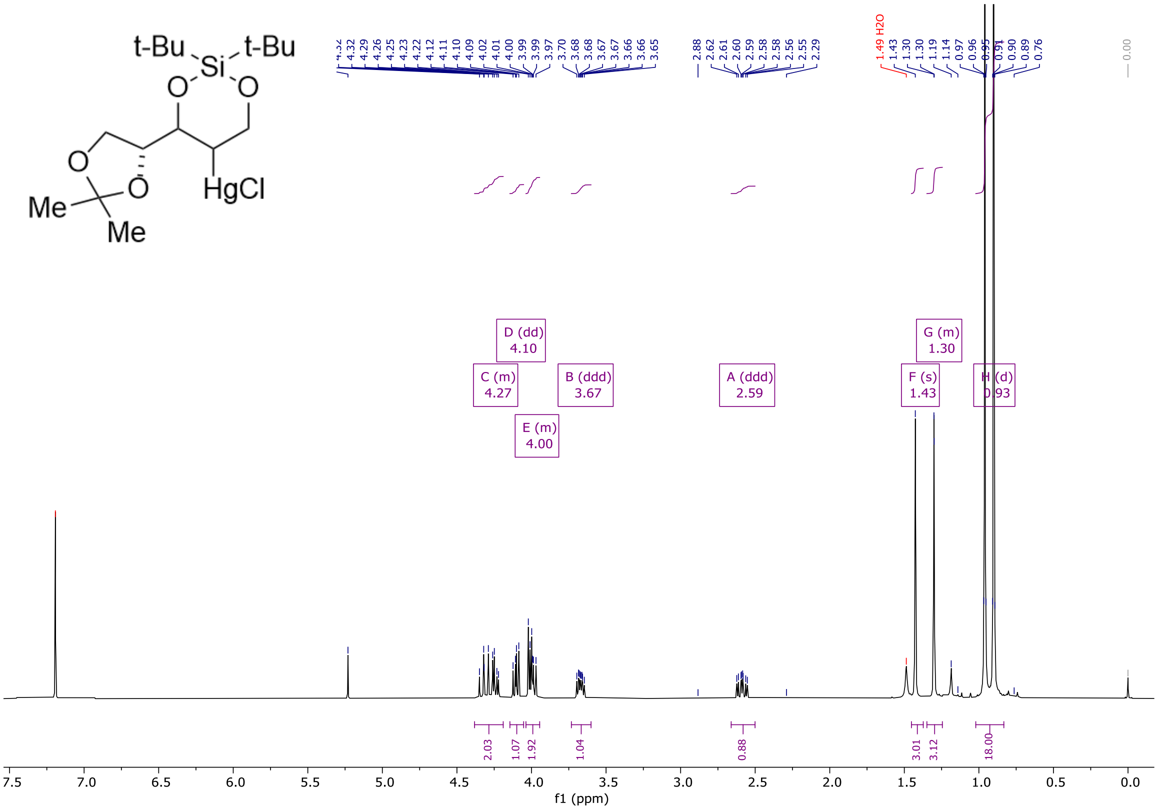

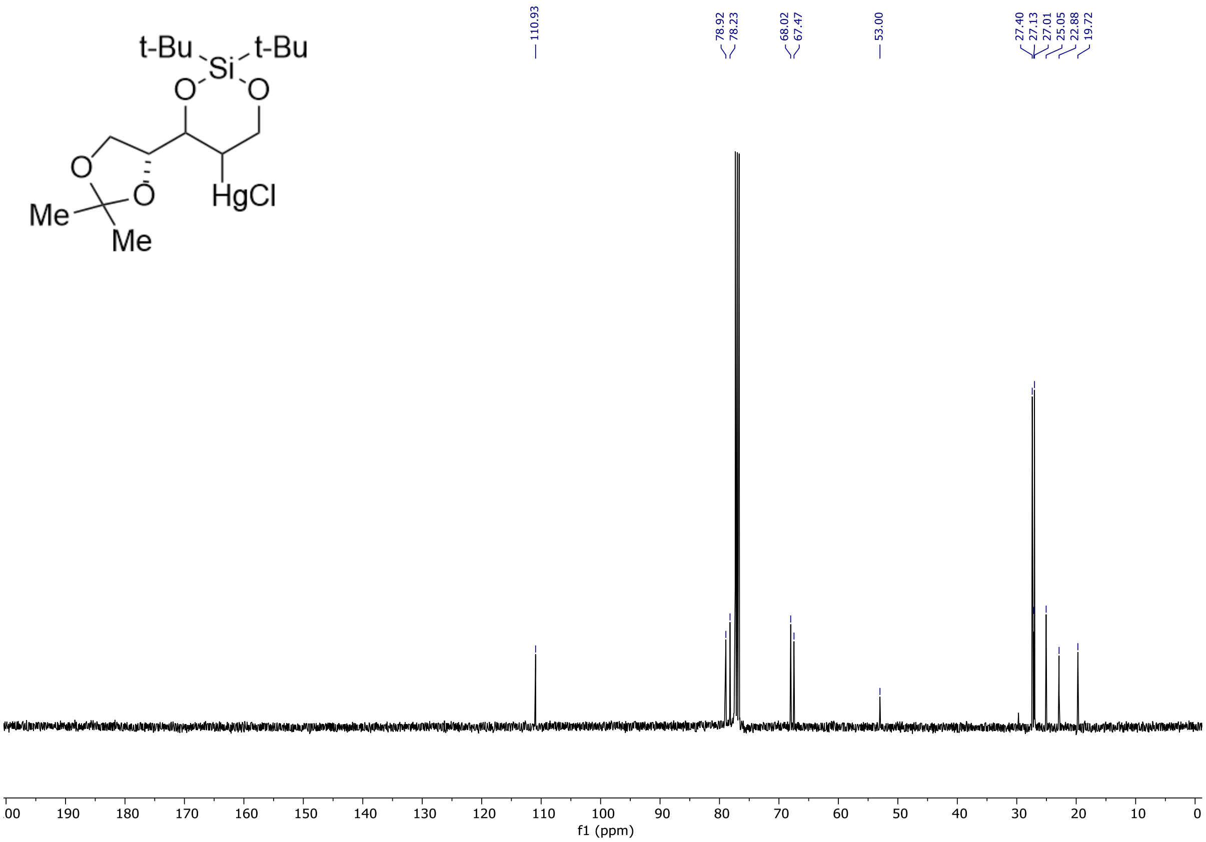

Compound 9 (^{1}H NMR: 400 MHz, ^{13}C NMR: 100 MHz)

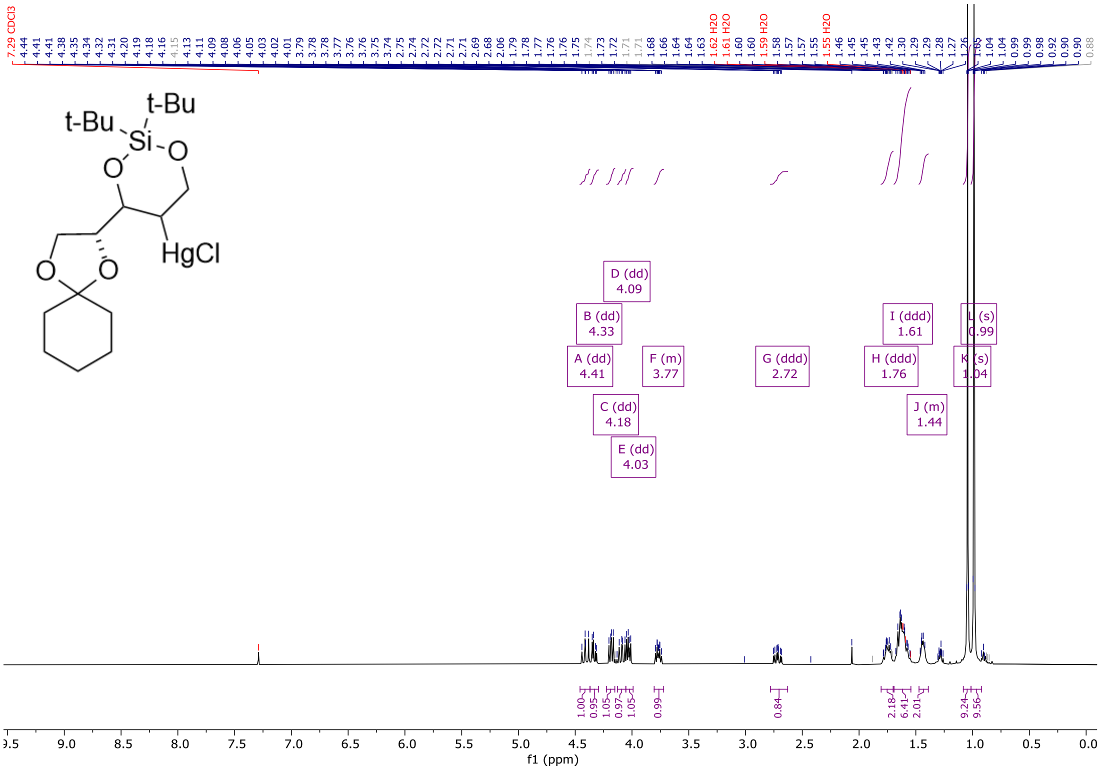

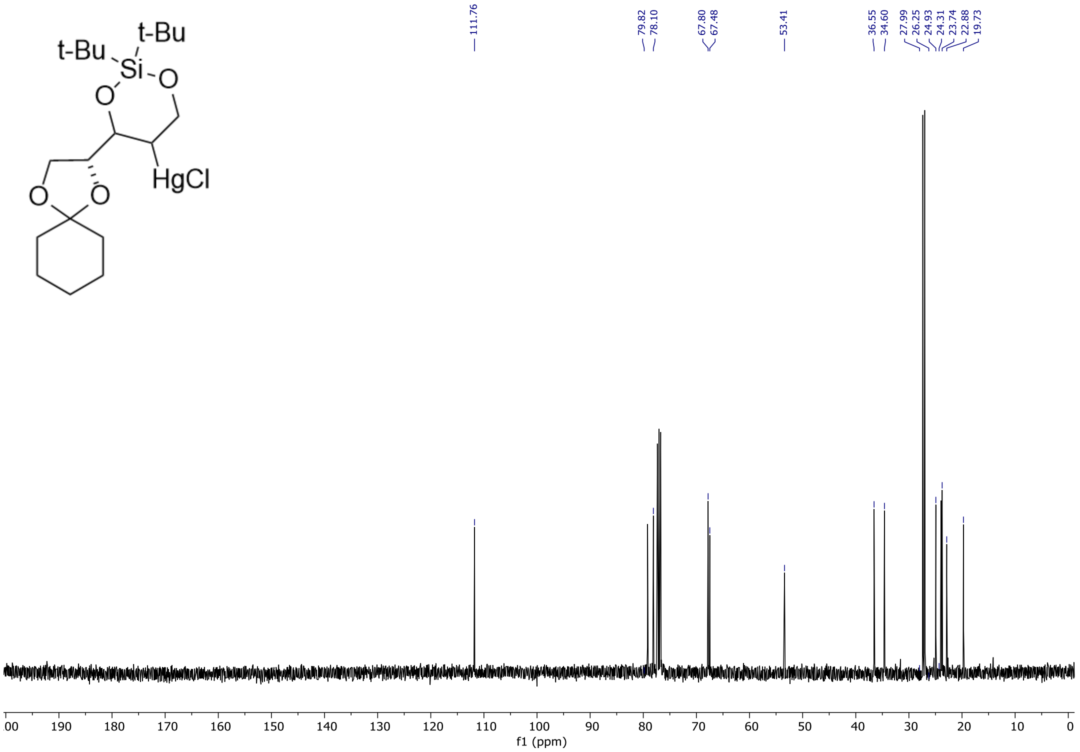

Compound 10 (^{1}H NMR: 400 MHz, ^{13}C NMR: 100 MHz)

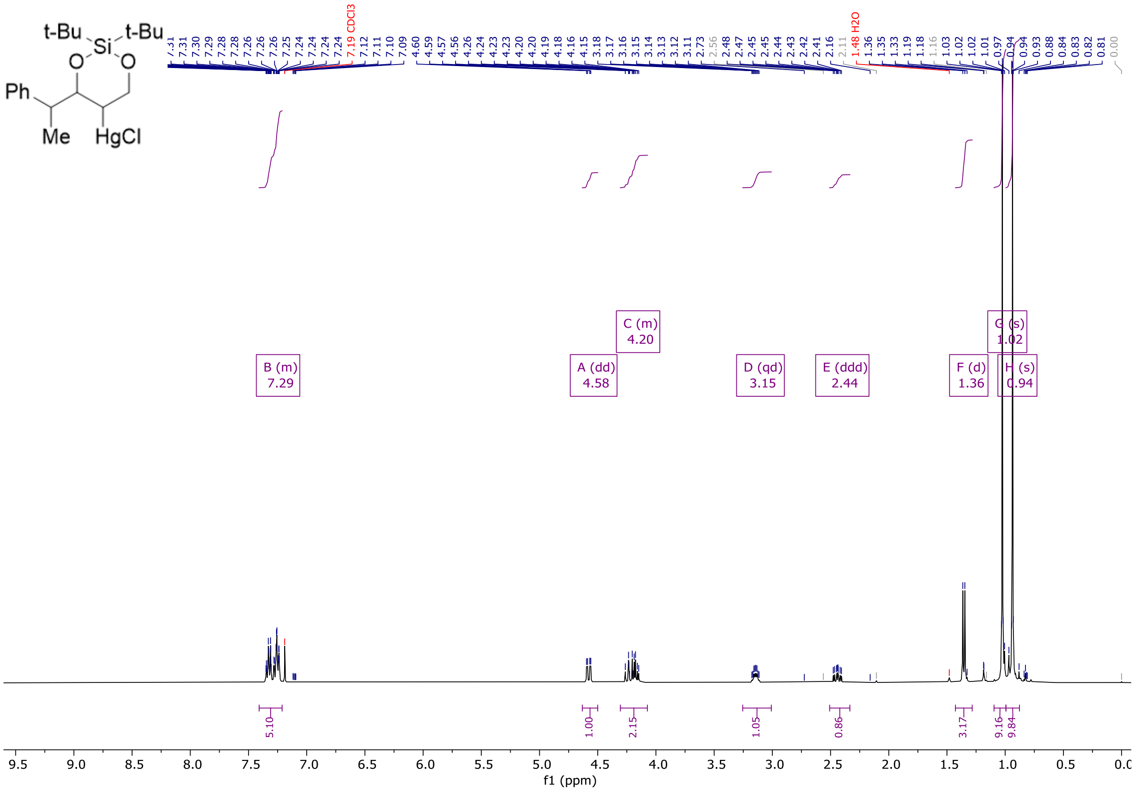

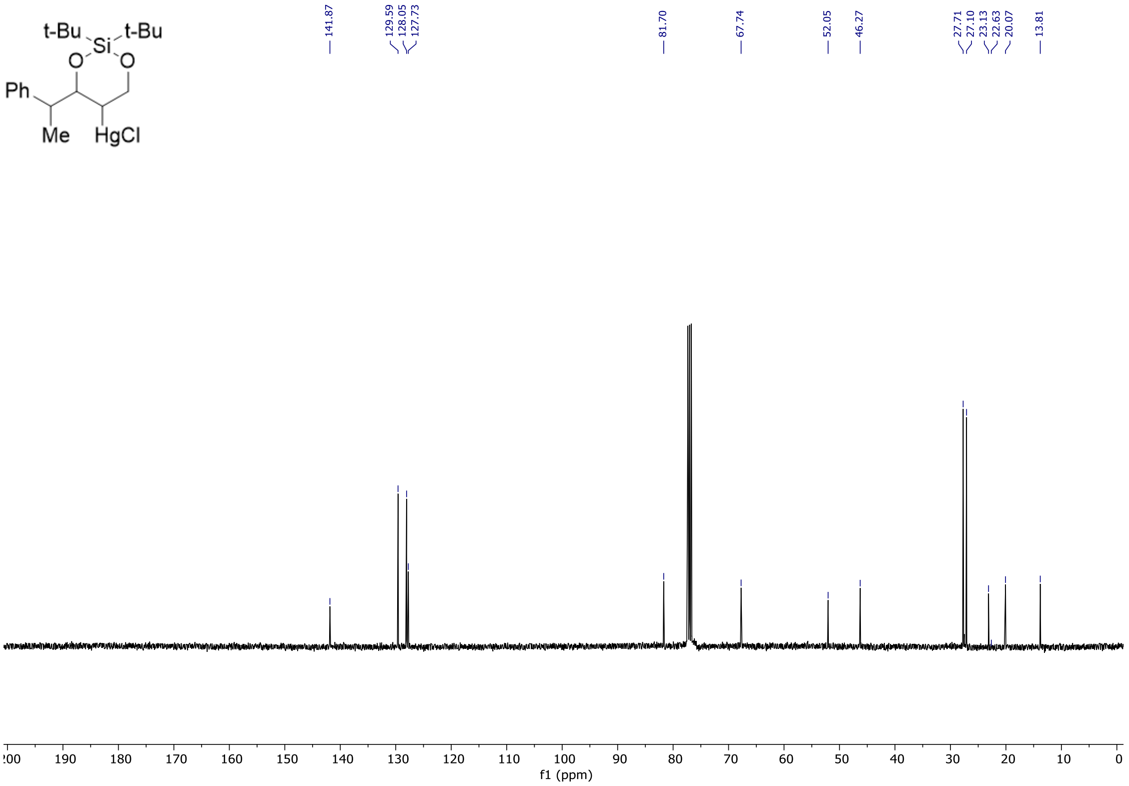

Compound 11 (^{1}H NMR: 400 MHz, ^{13}C NMR: 100 MHz)

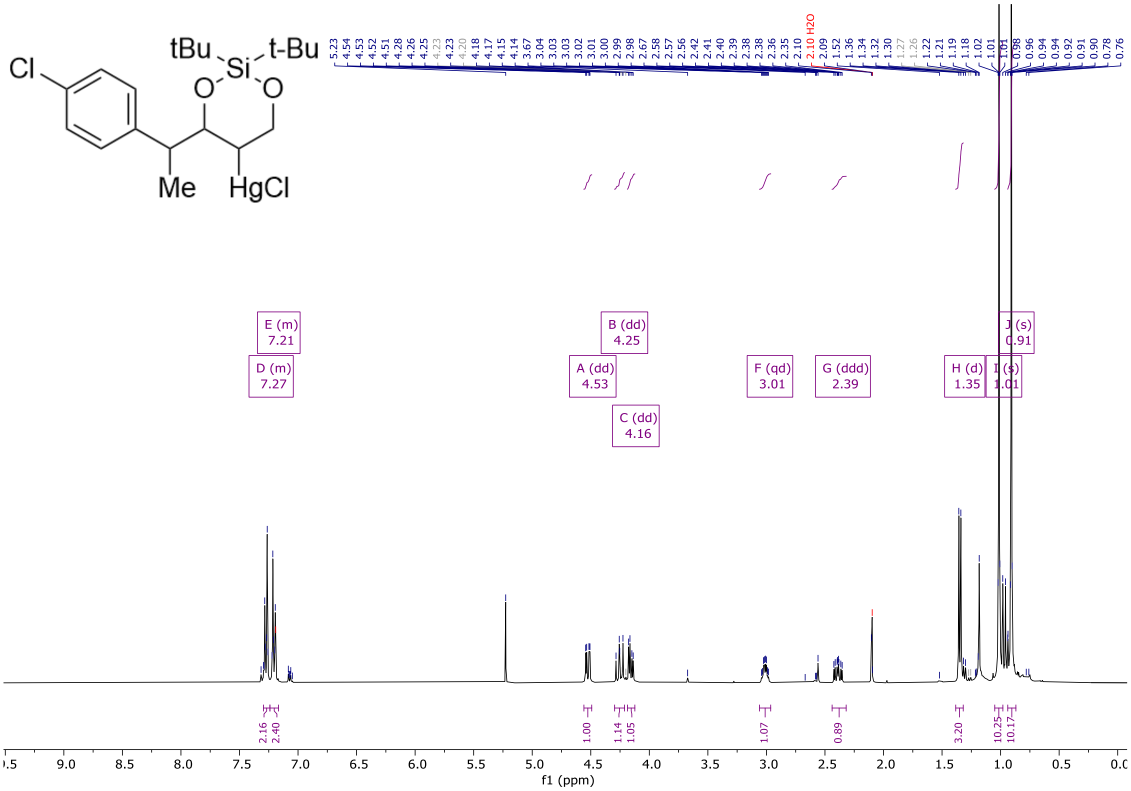

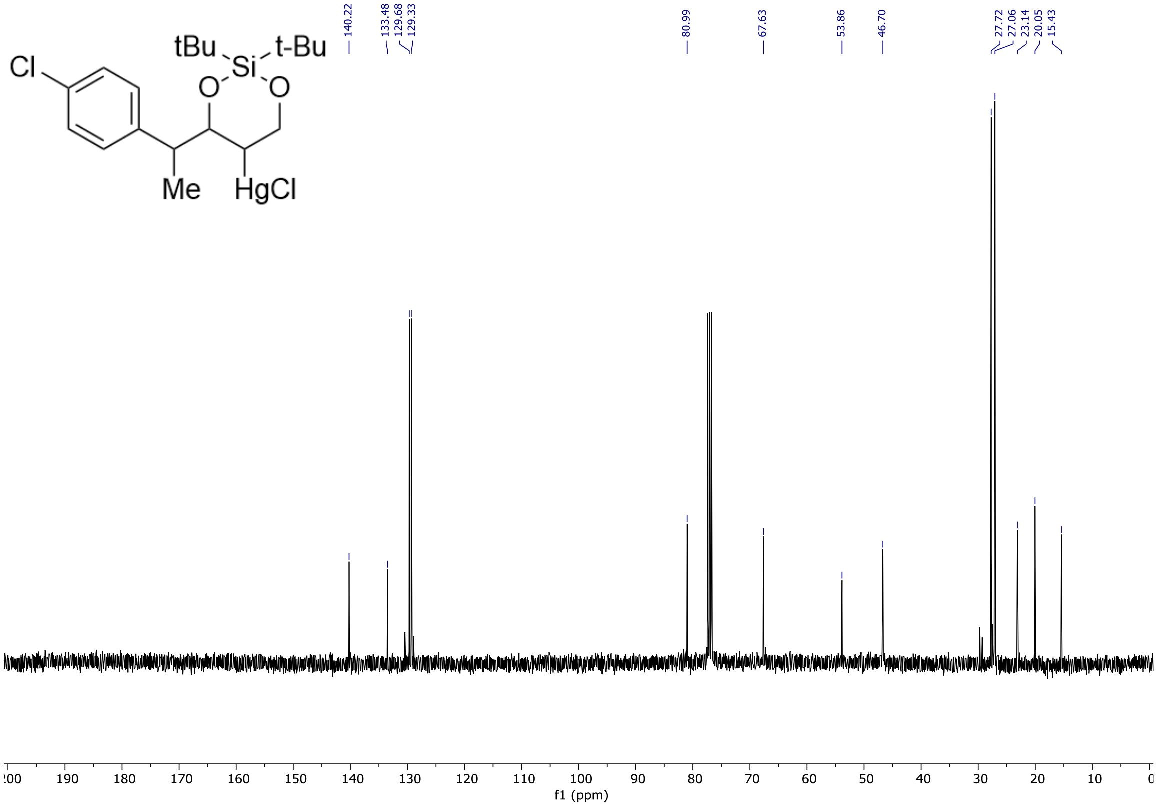

Compound 12 (^{1}H NMR: 400 MHz, ^{13}C NMR: 100 MHz)

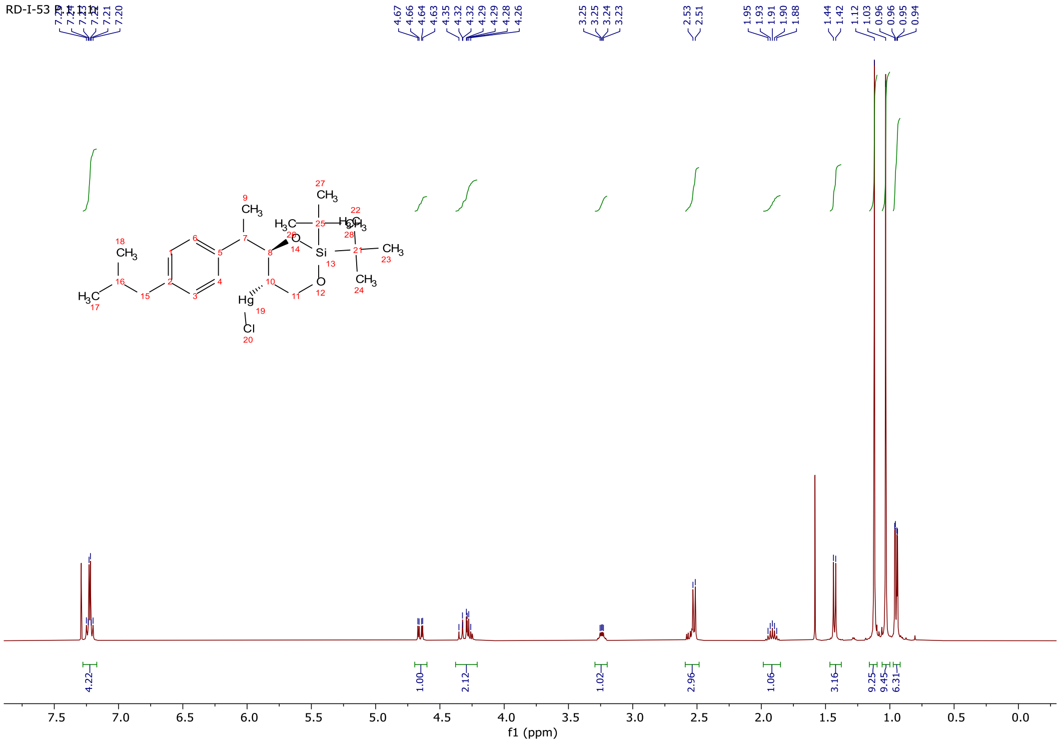

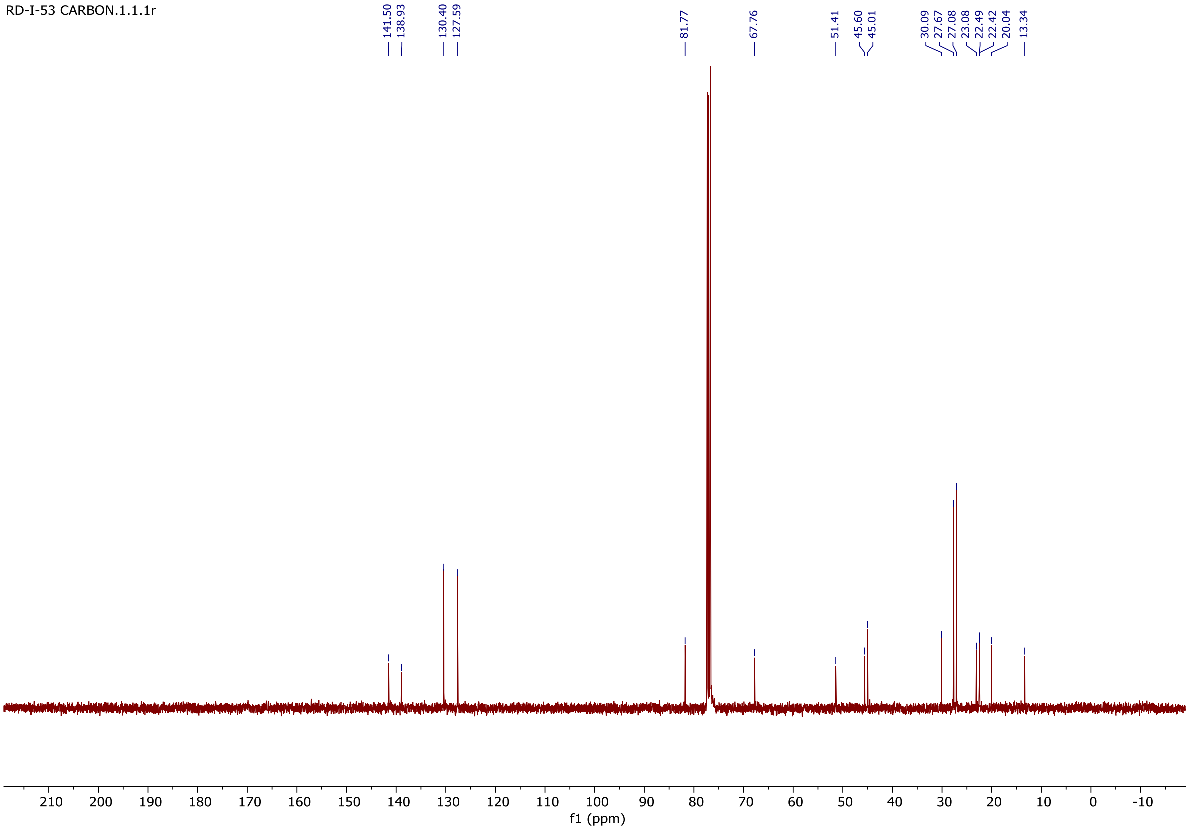

Compound 13 (^{1}H NMR: 400 MHz, ^{13}C NMR: 100 MHz)

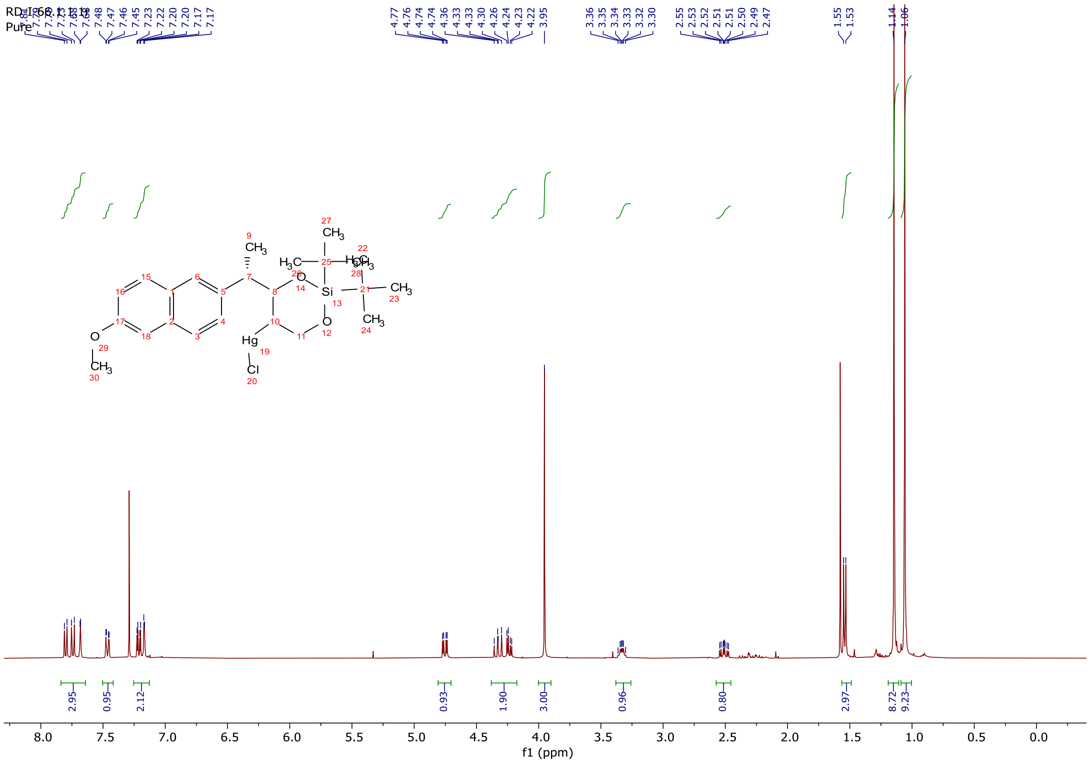

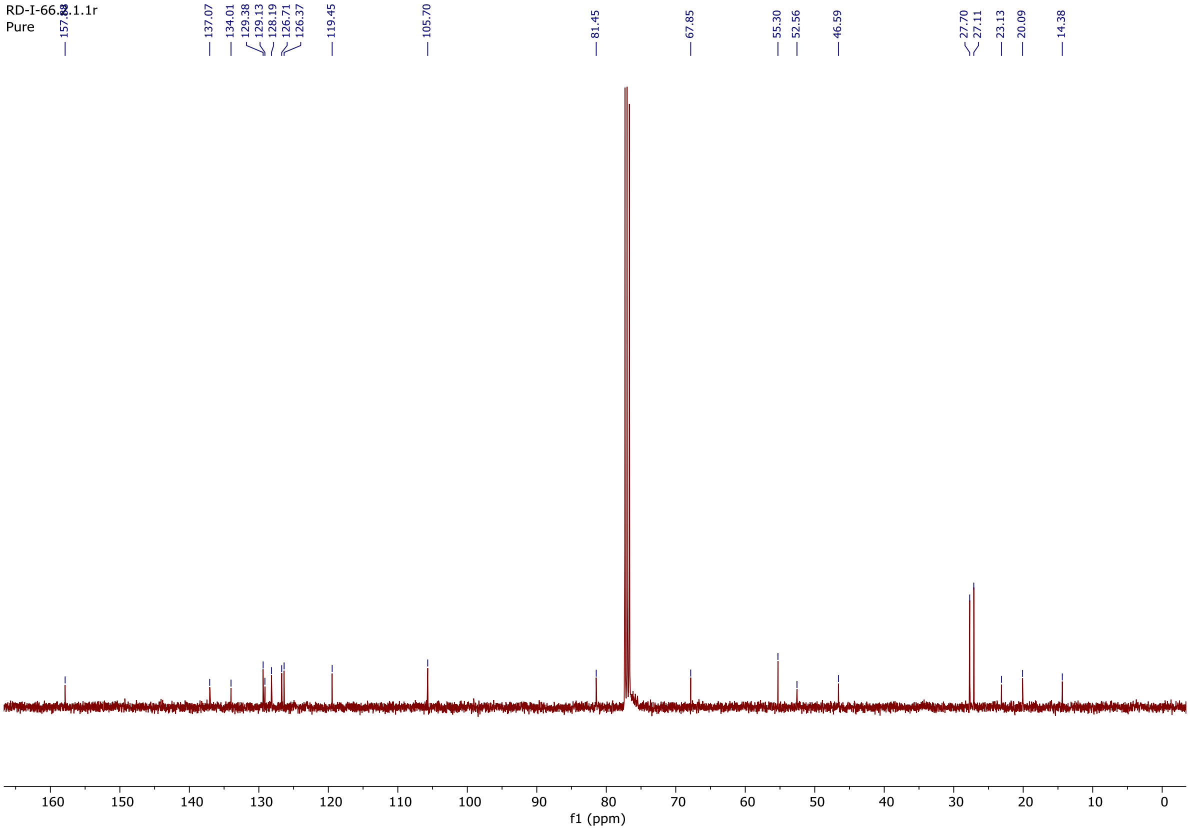

Compound 14 (^{1}H NMR: 400 MHz, ^{13}C NMR: 100 MHz)

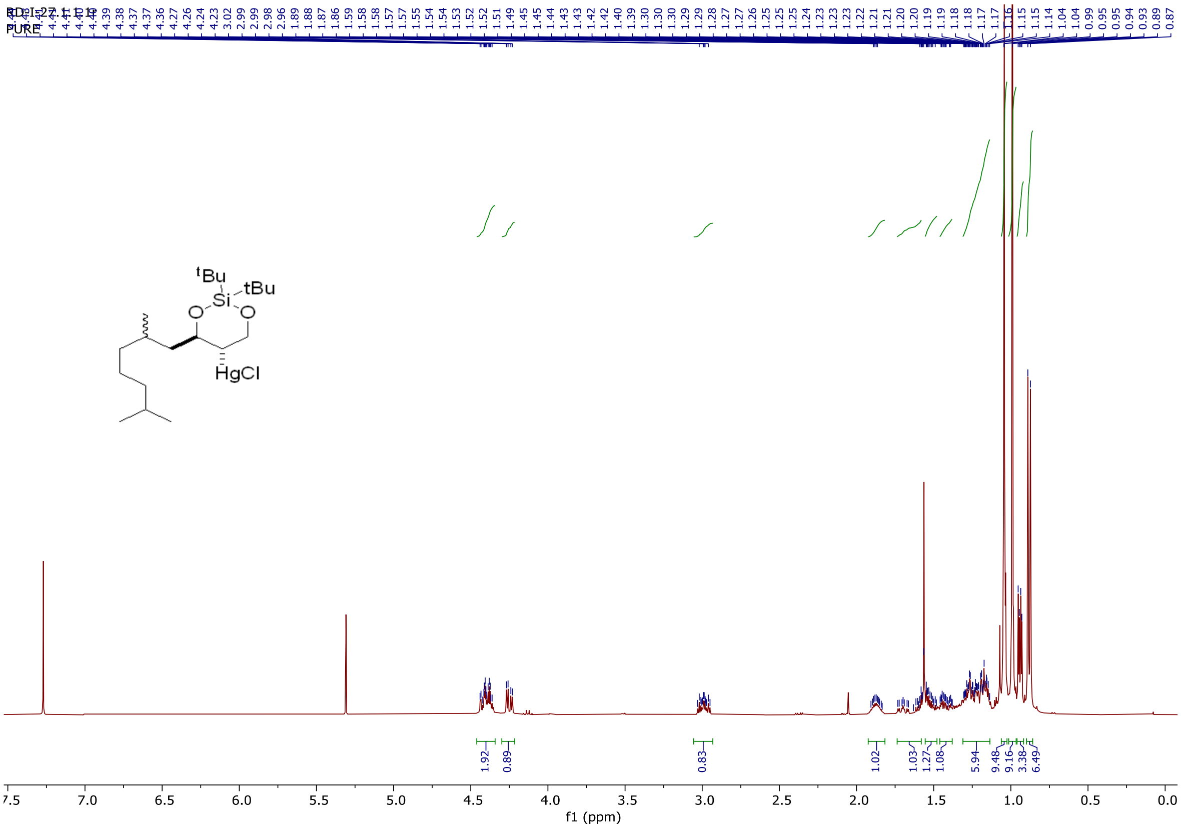

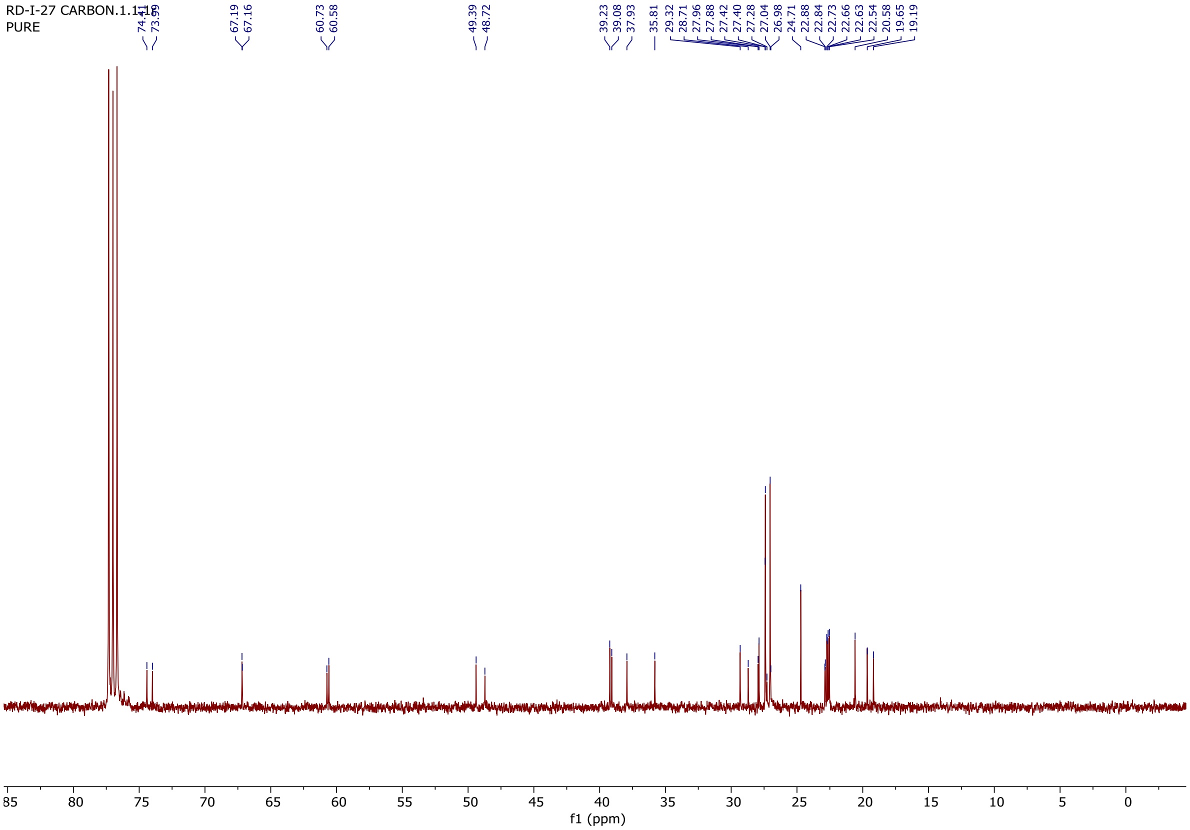

Compound 15 (^{1}H NMR: 400 MHz, ^{13}C NMR: 100 MHz)

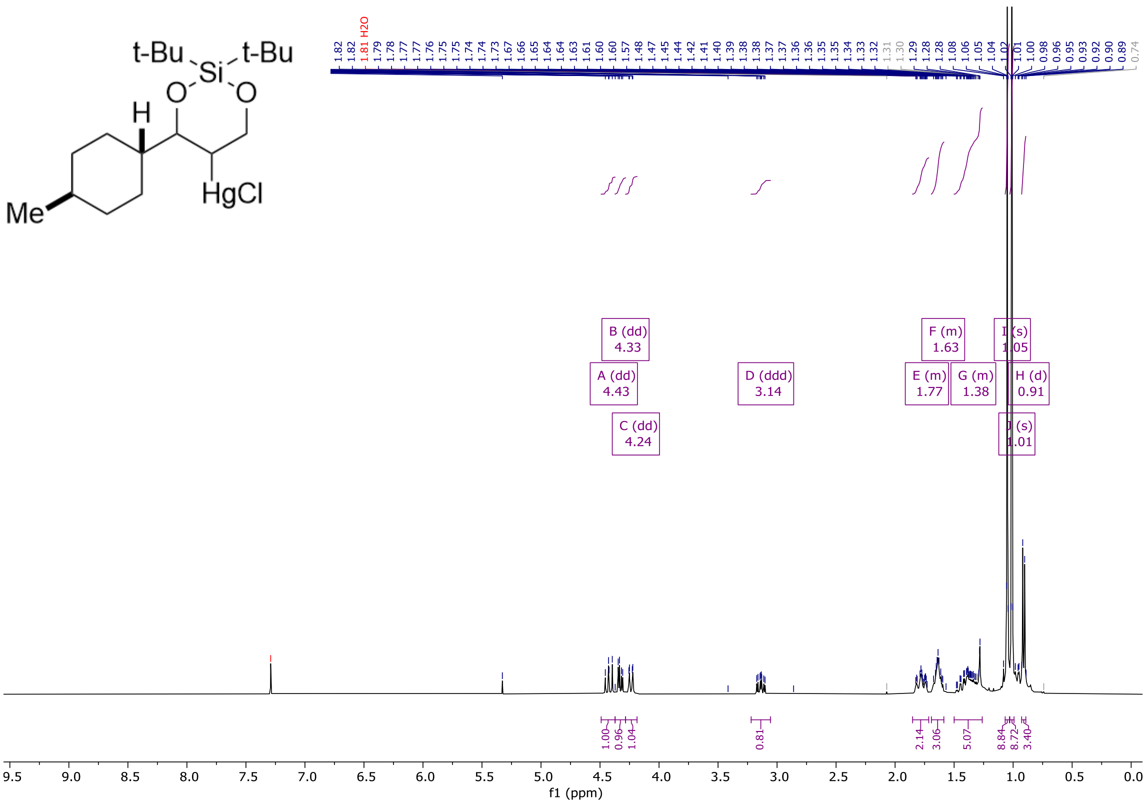

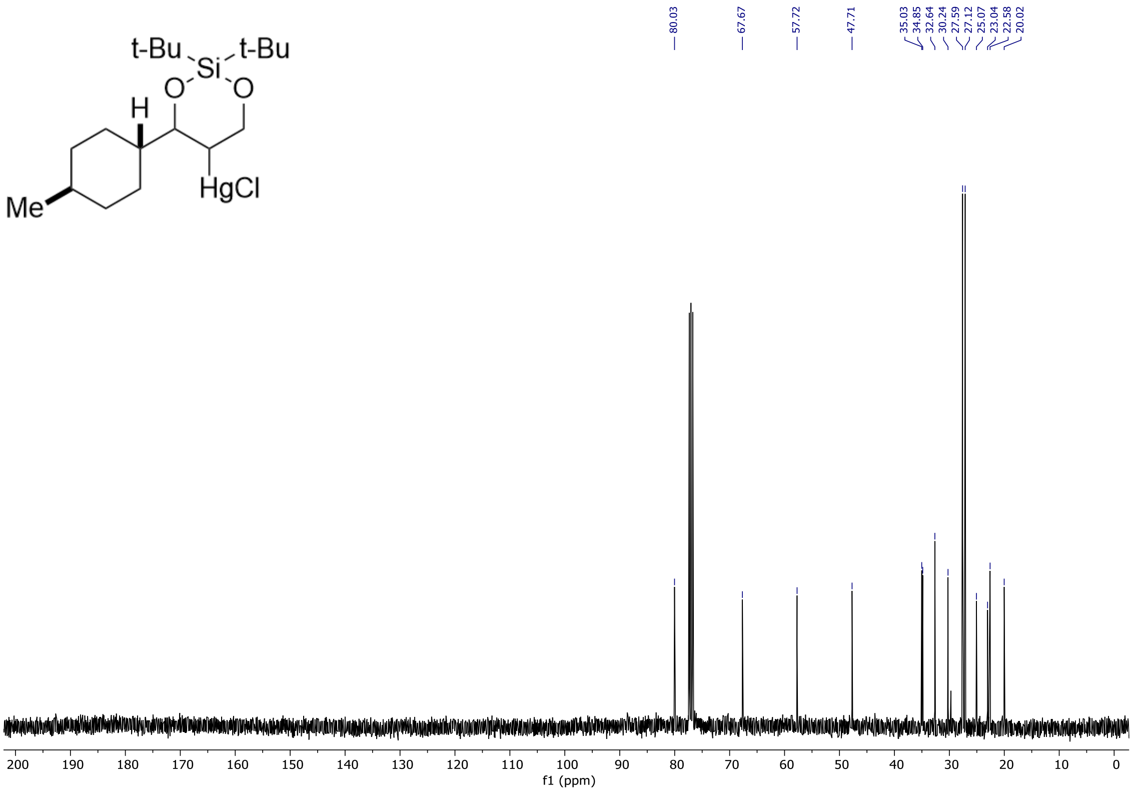

Compound 16 (^{1}H NMR: 400 MHz, ^{13}C NMR: 100 MHz)

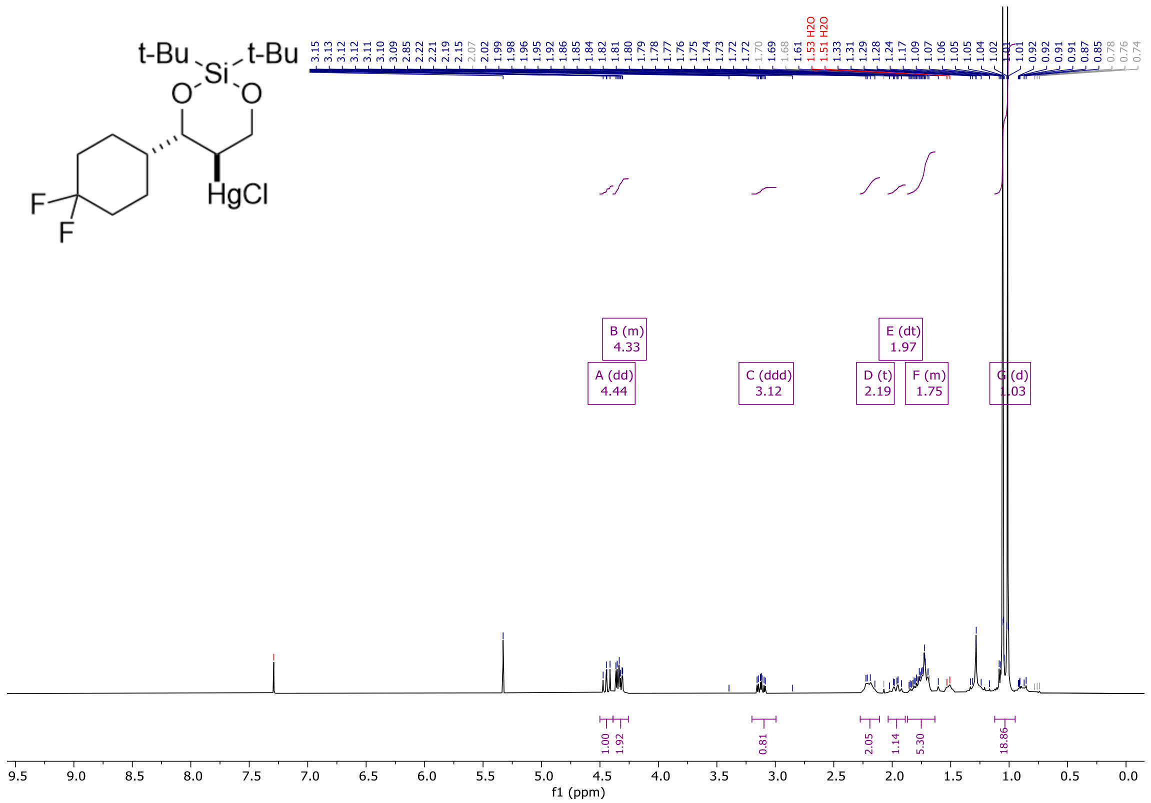

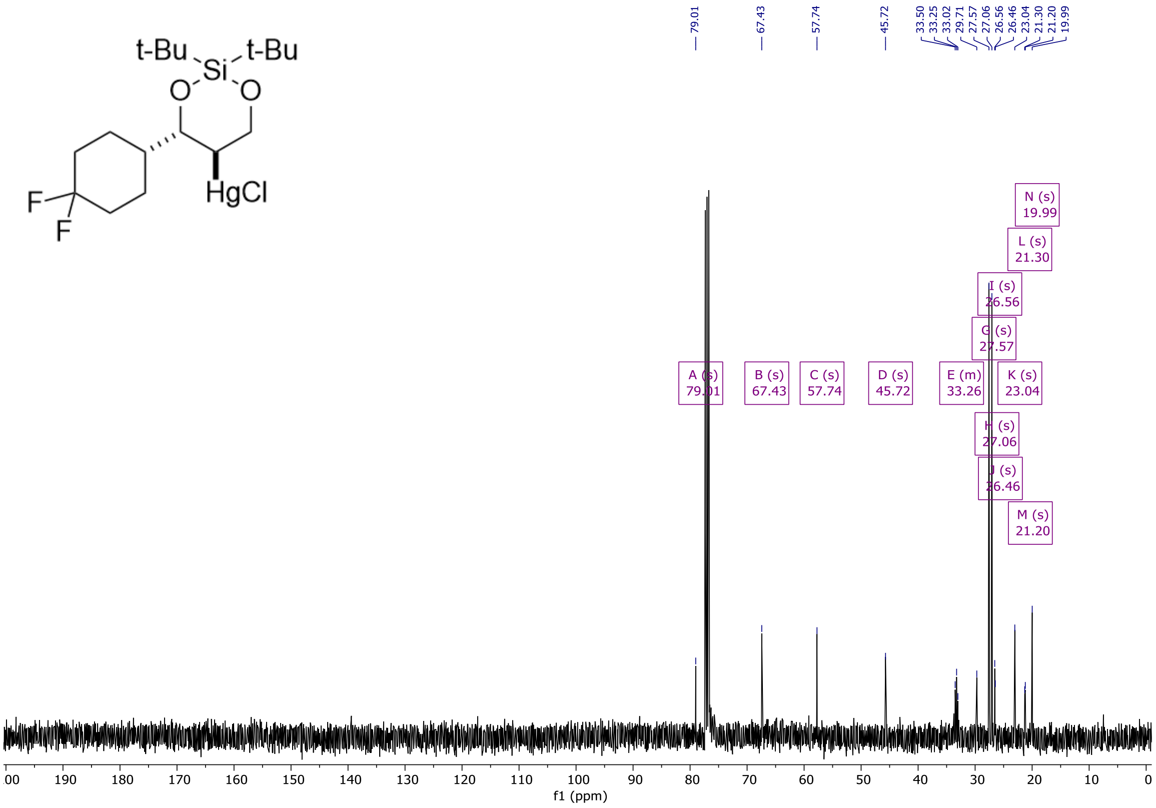

Compound 17 (^{1}H NMR: 400 MHz, ^{13}C NMR: 100 MHz)

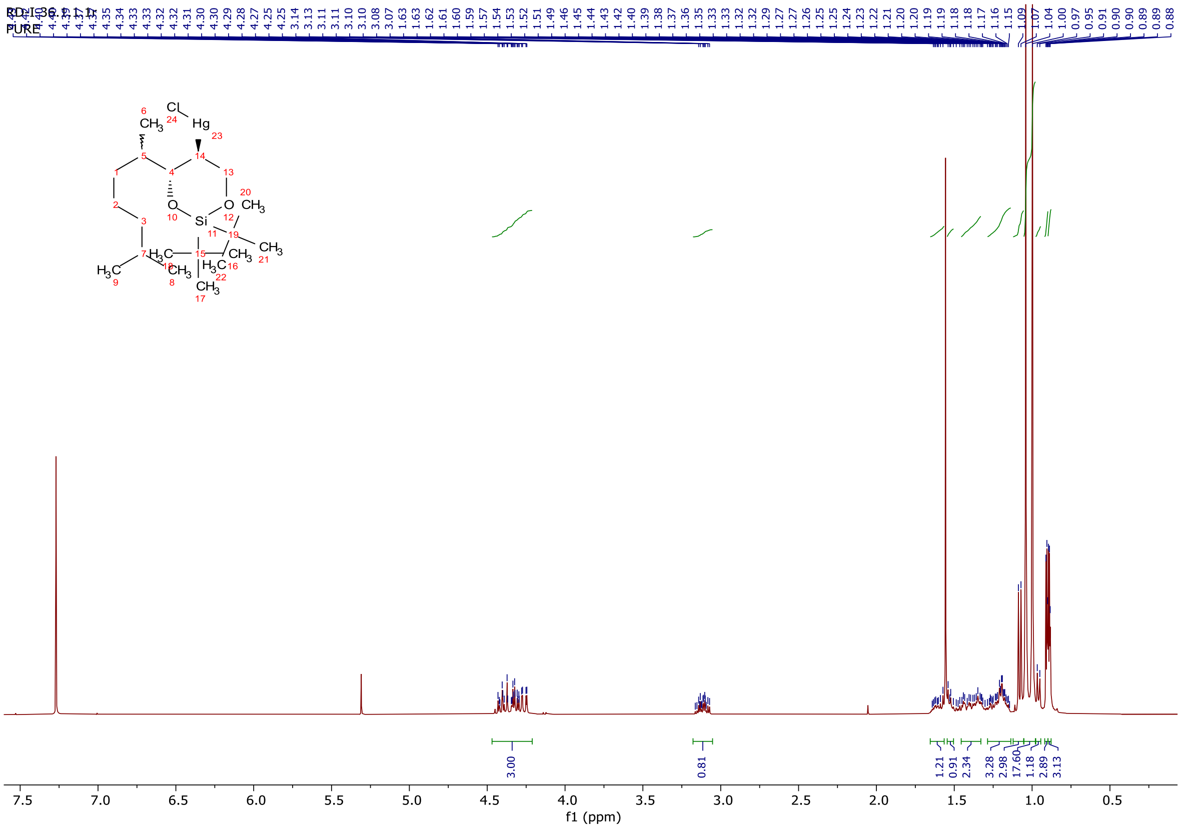

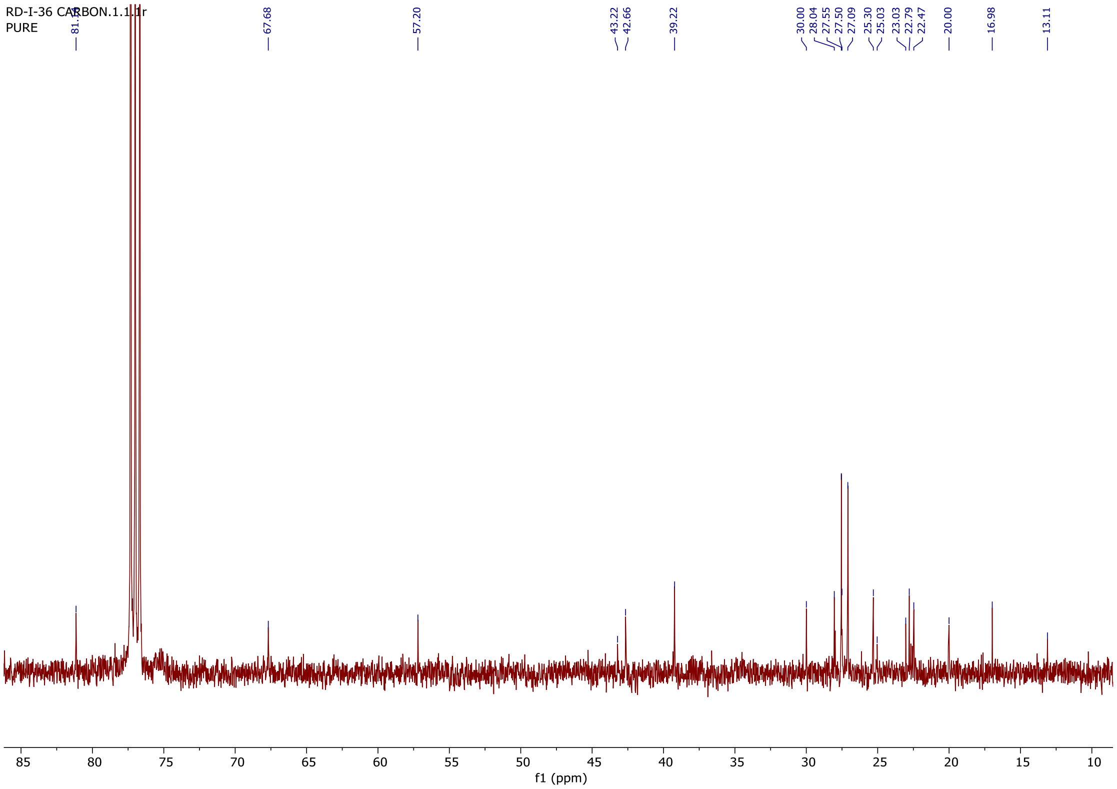

Compound 18 (^{1}H NMR: 400 MHz, ^{13}C NMR: 100 MHz)

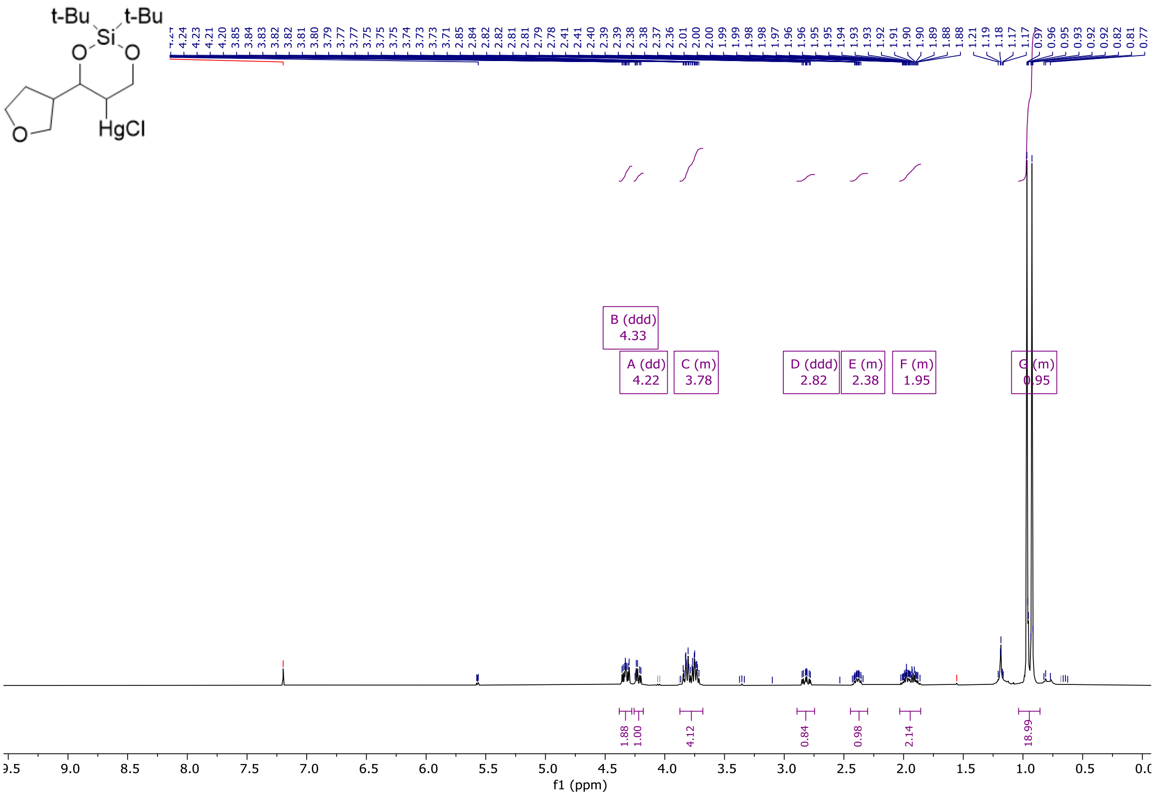

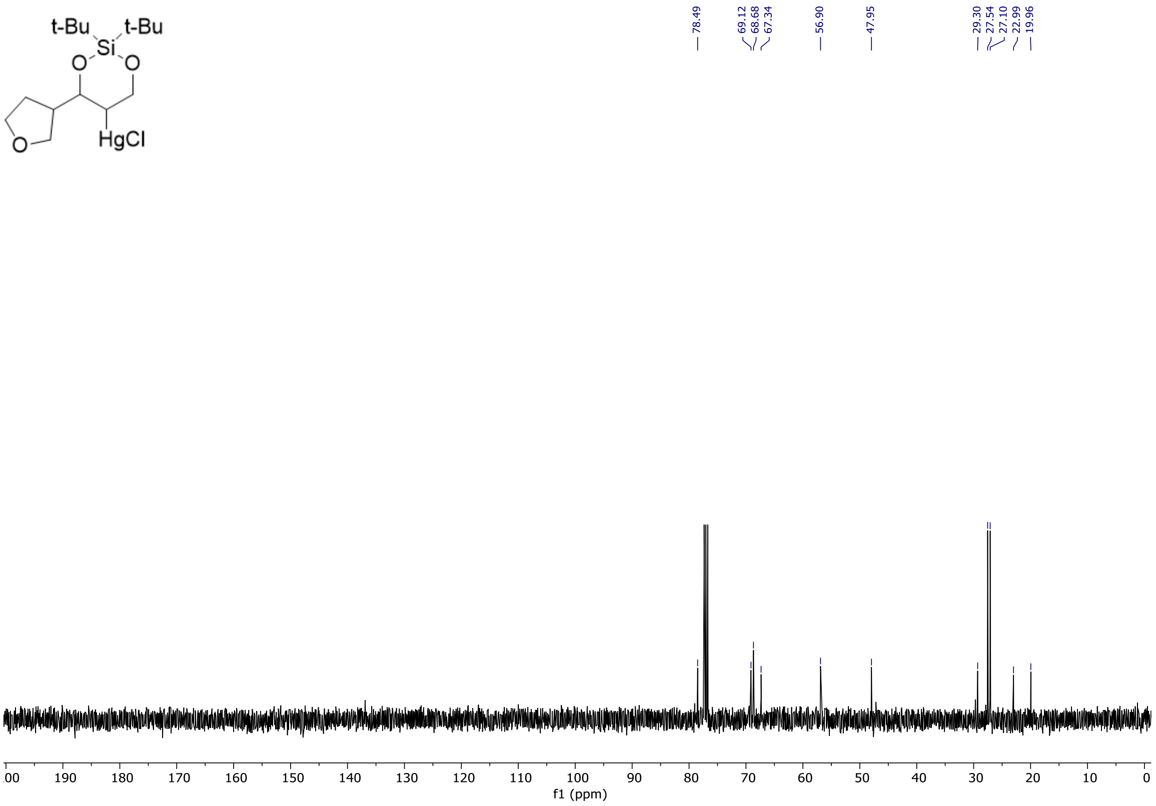

Compound 20 (^{1}H NMR: 400 MHz, ^{13}C NMR: 100 MHz)

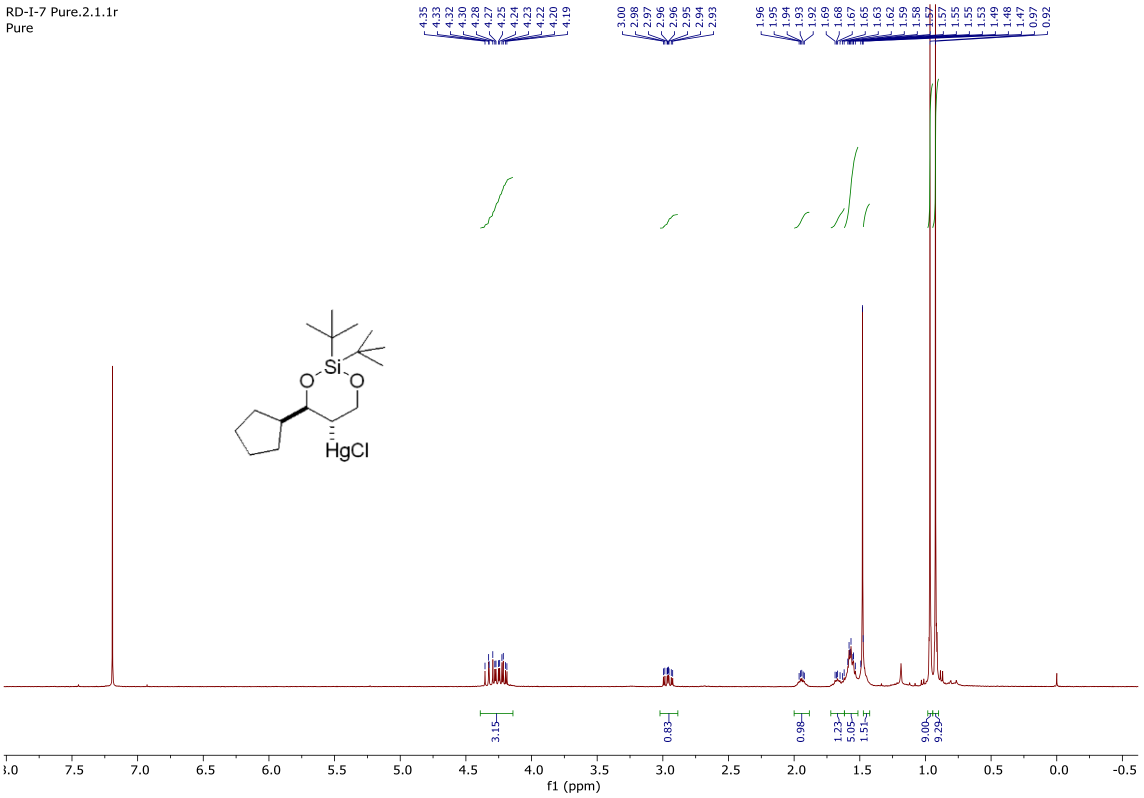

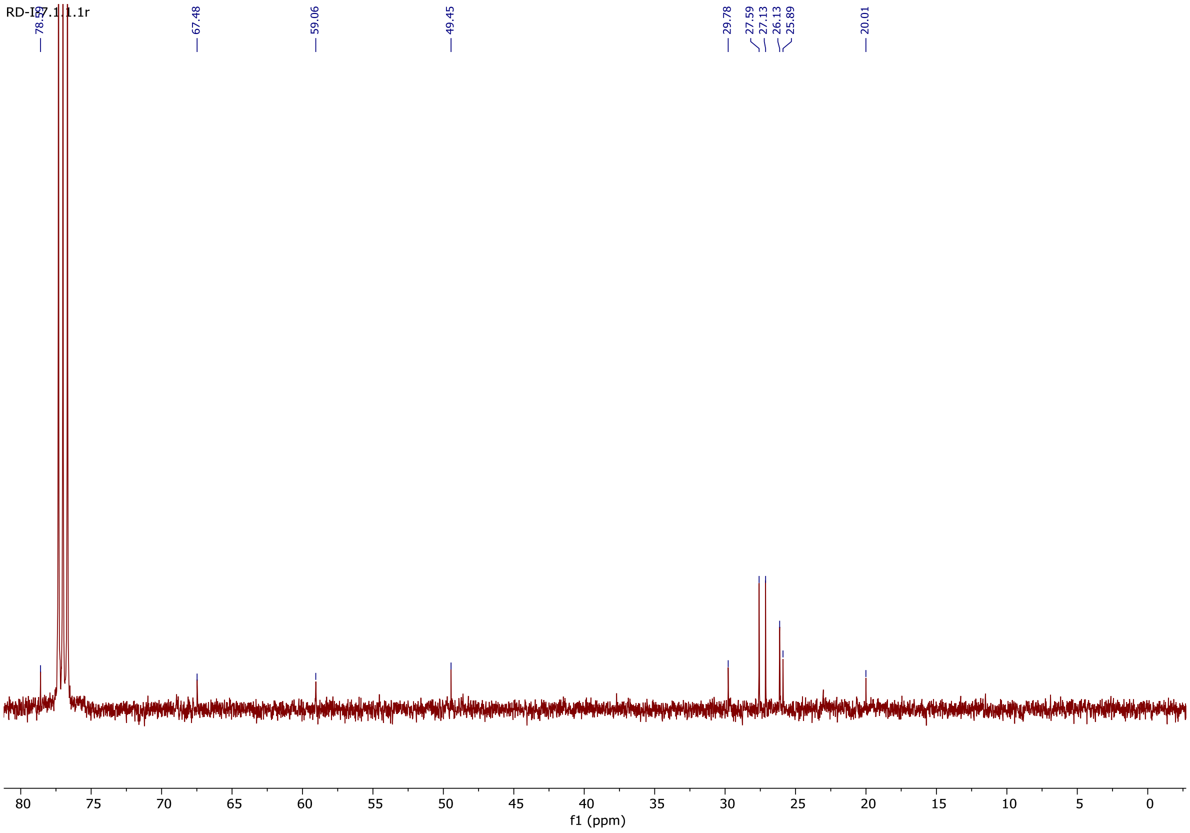

Compound 21 (^{1}H NMR: 400 MHz, ^{13}C NMR: 100 MHz)

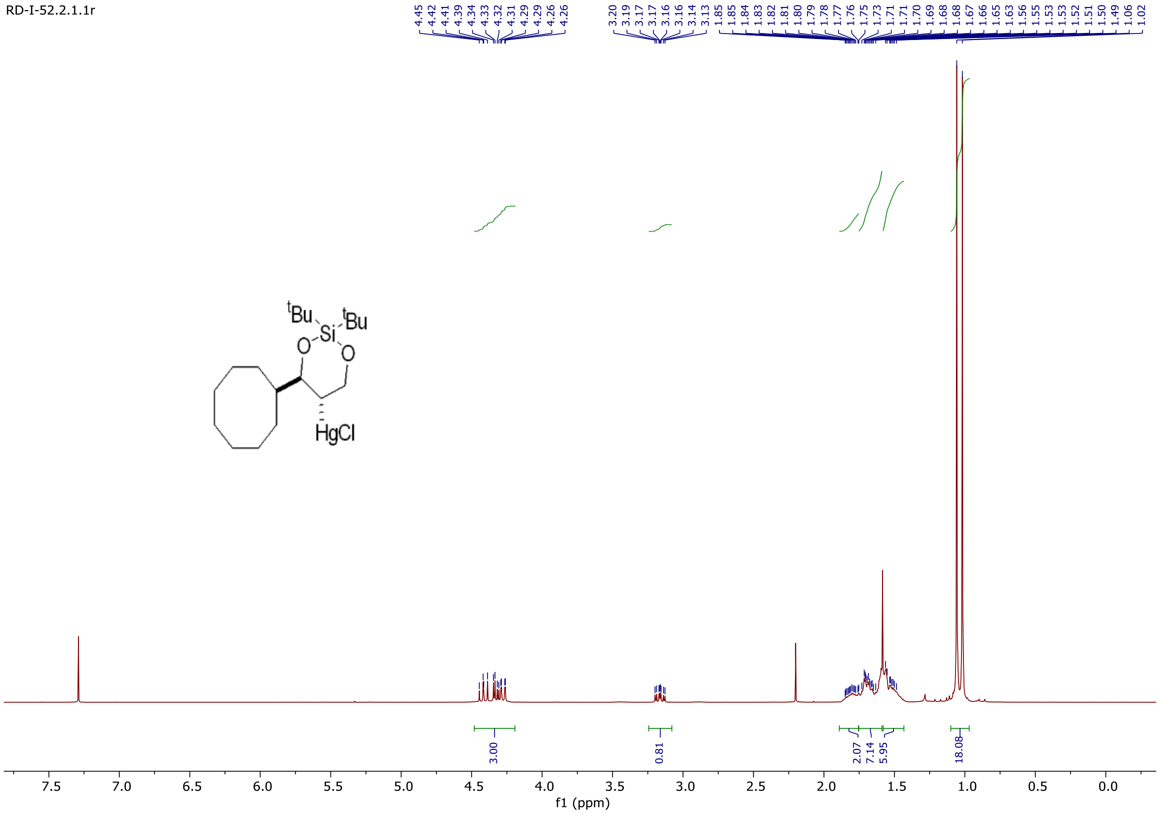

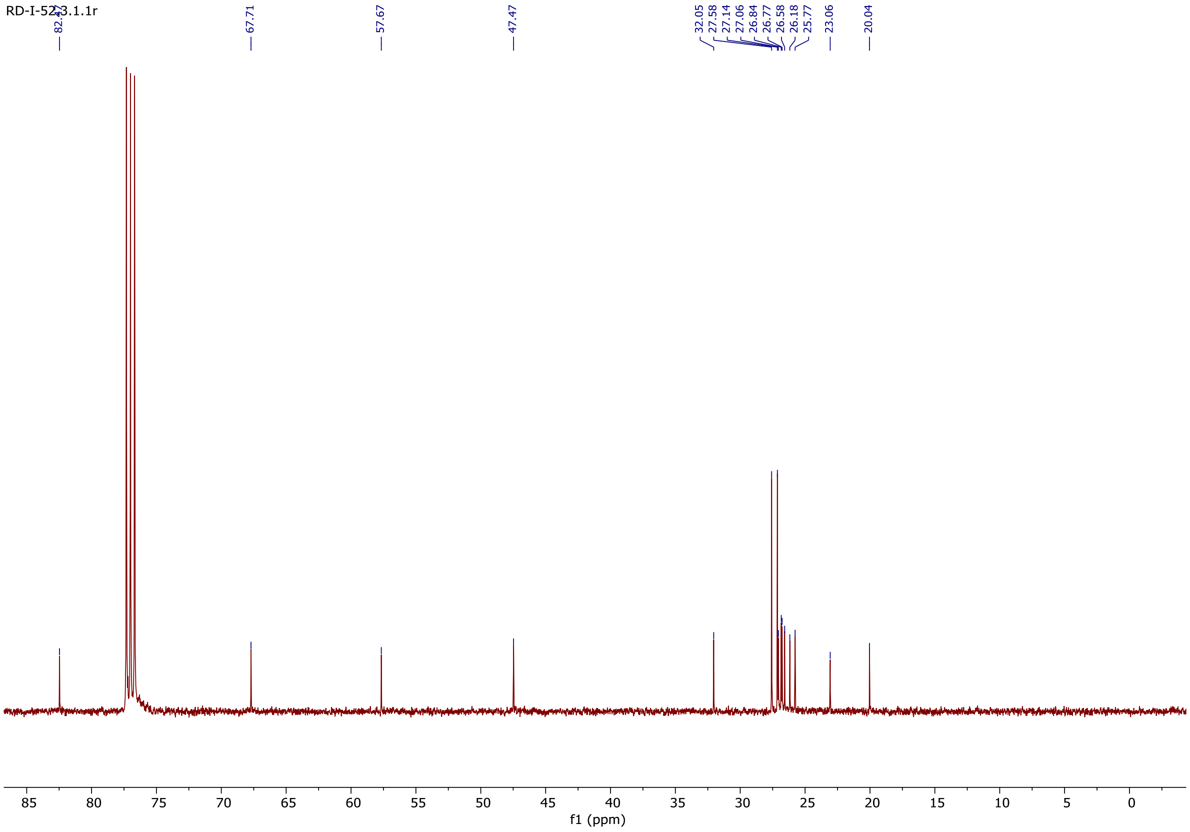

Compound 22 (^{1}H NMR: 400 MHz, ^{13}C NMR: 100 MHz)

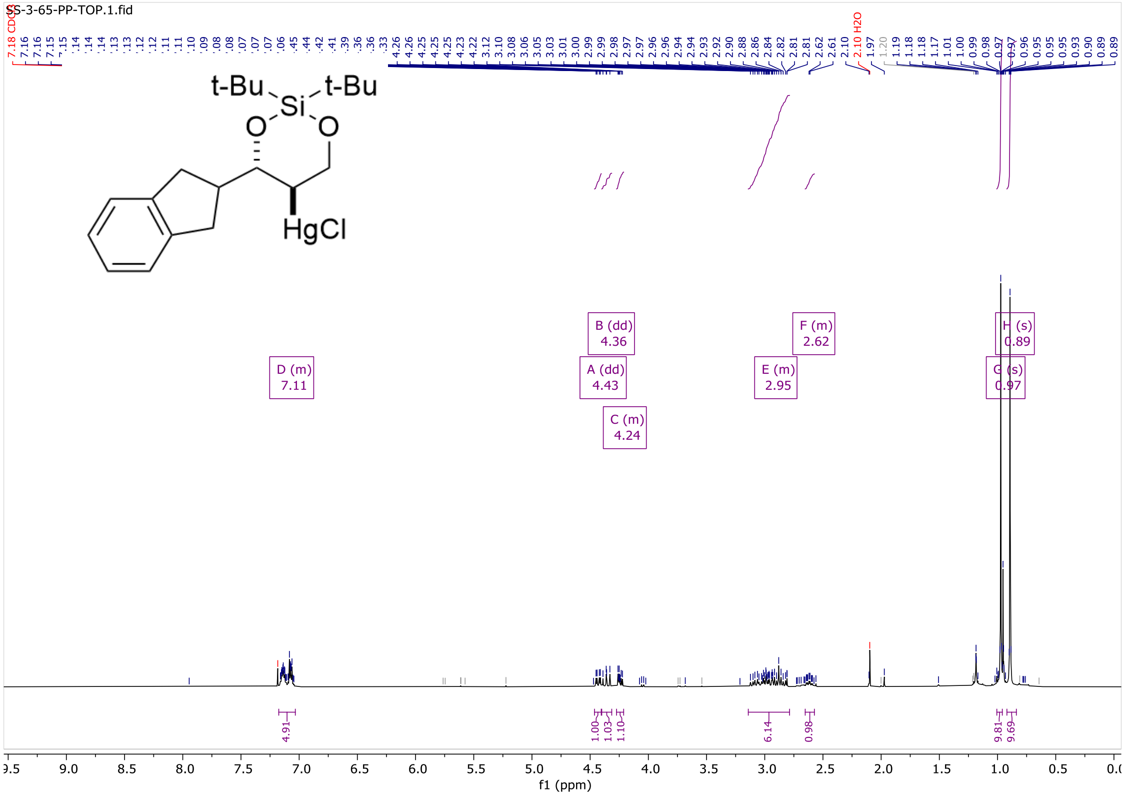

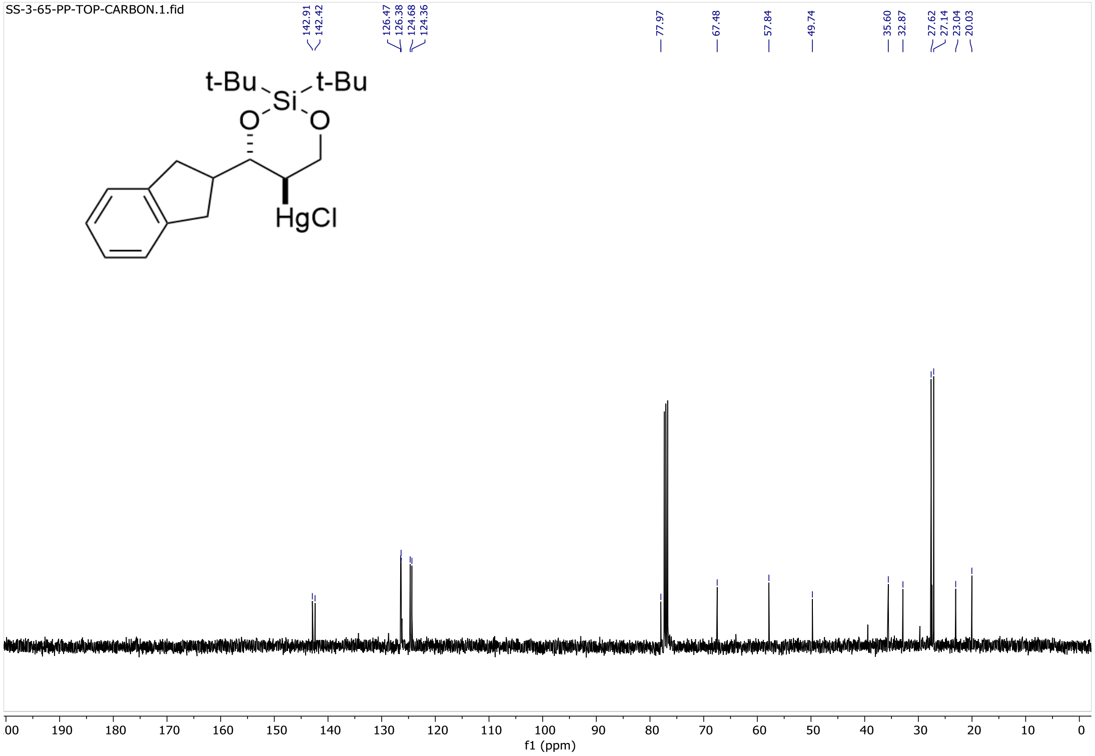

Compound 23 (^{1}H NMR: 400 MHz, ^{13}C NMR: 100 MHz)

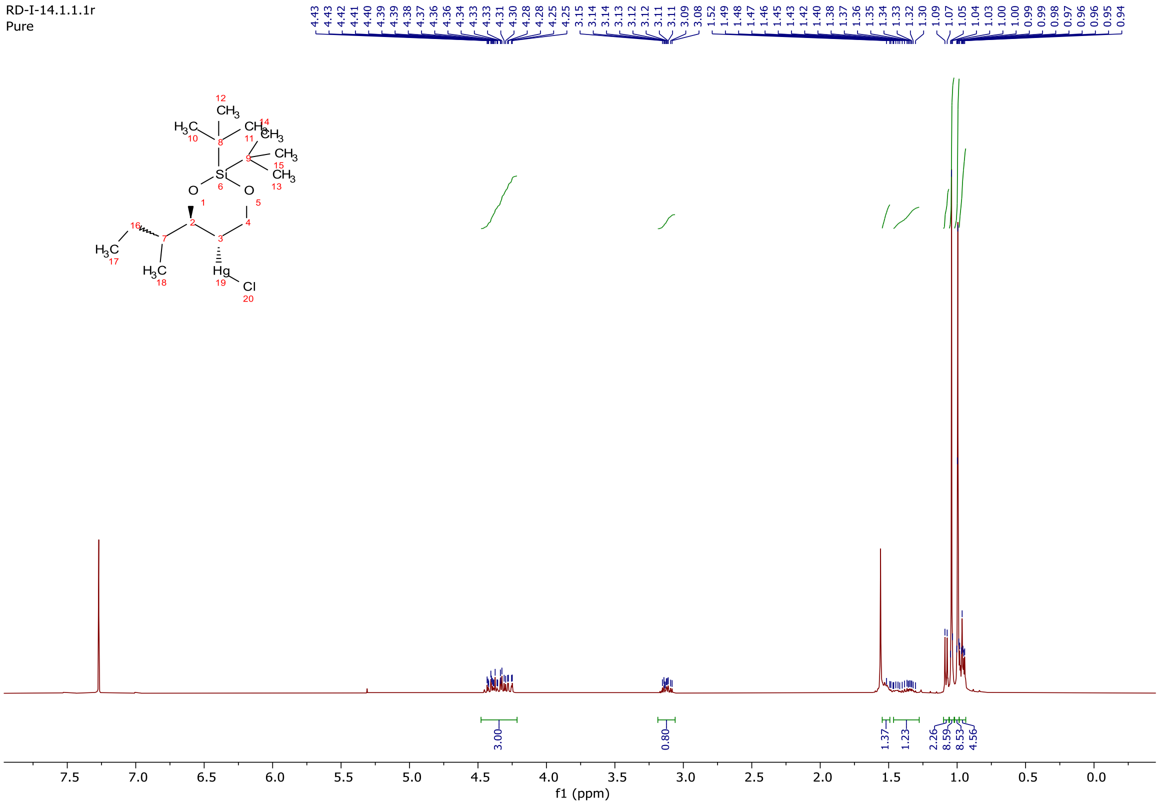

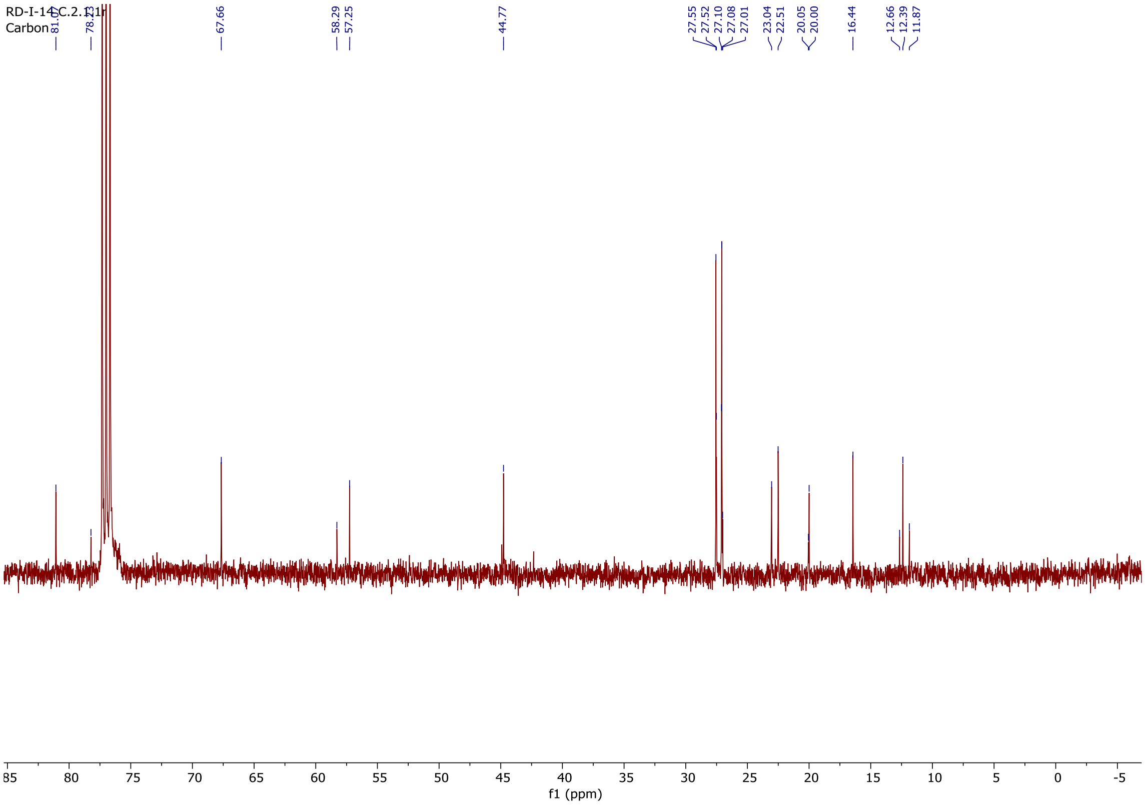

Compound 24 (^{1}H NMR: 400 MHz, ^{13}C NMR: 100 MHz)

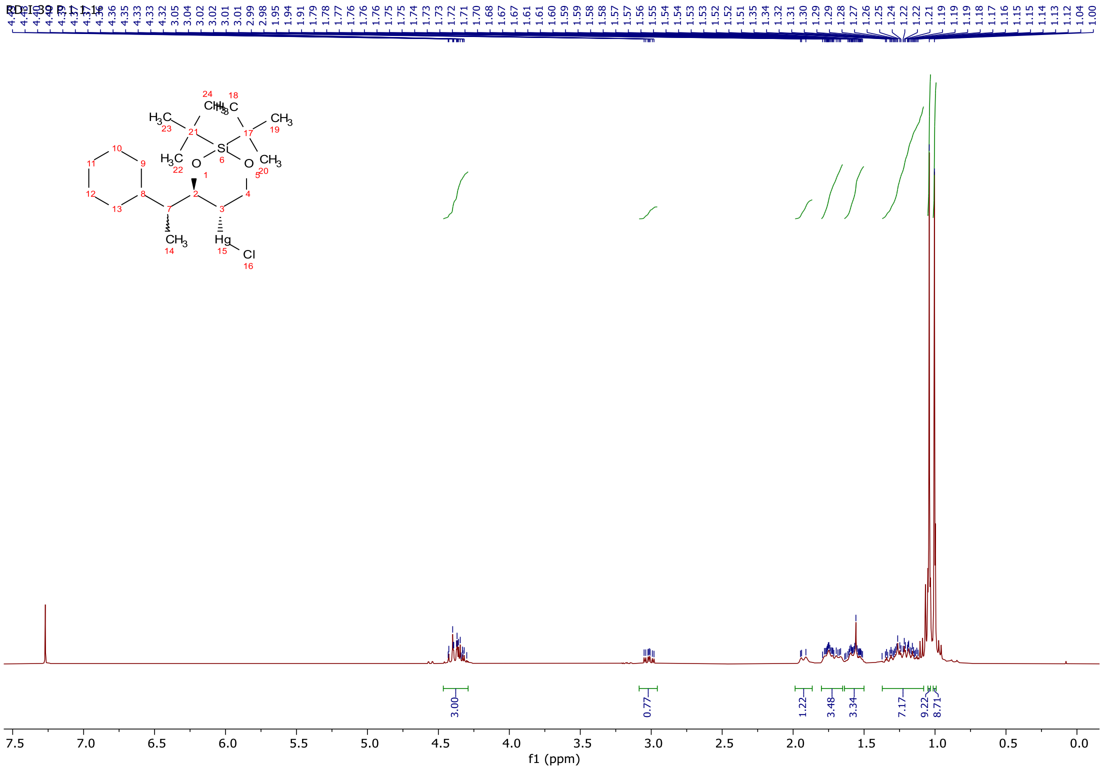

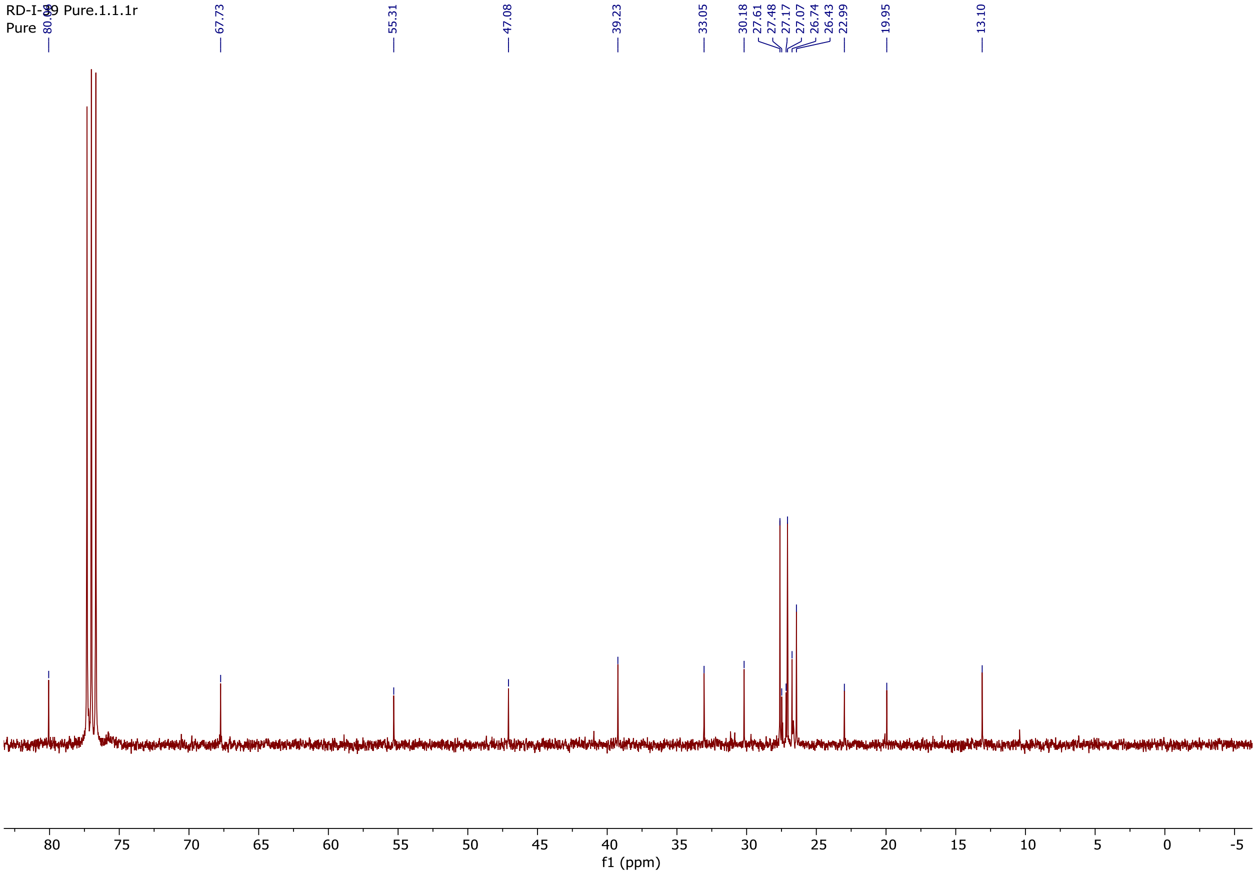

Compound 25 (^{1}H NMR: 400 MHz, ^{13}C NMR: 100 MHz)

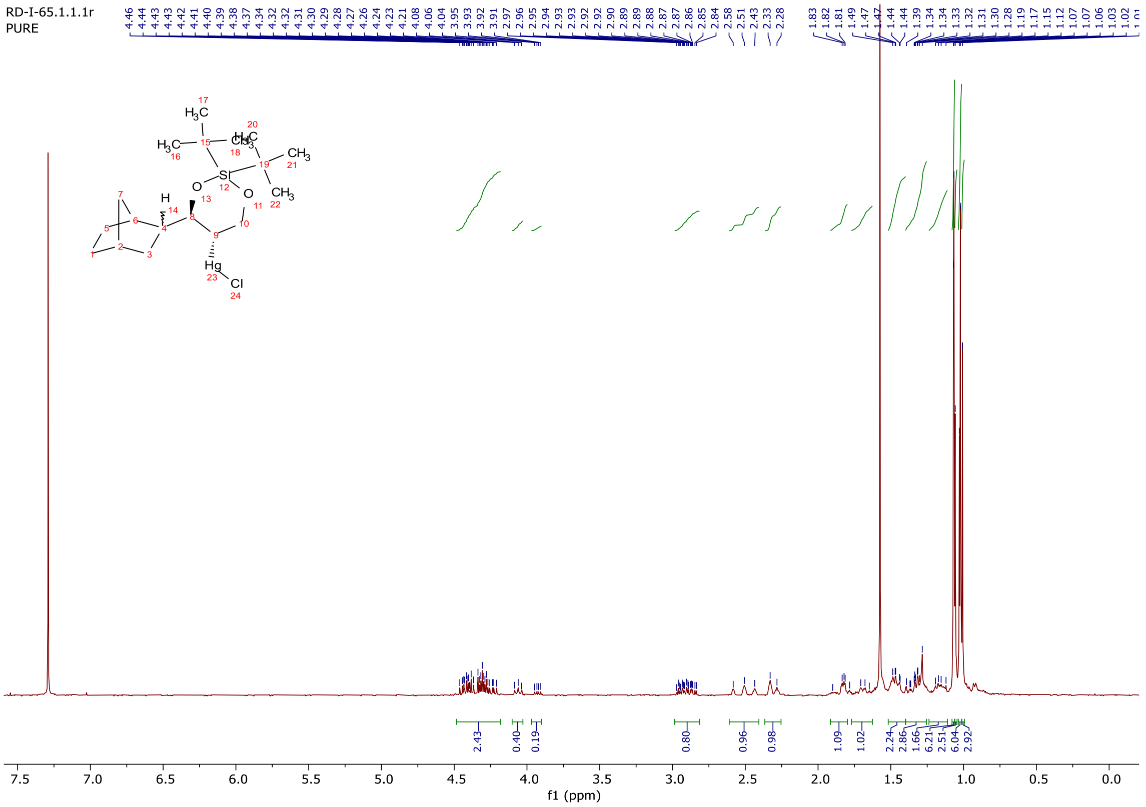

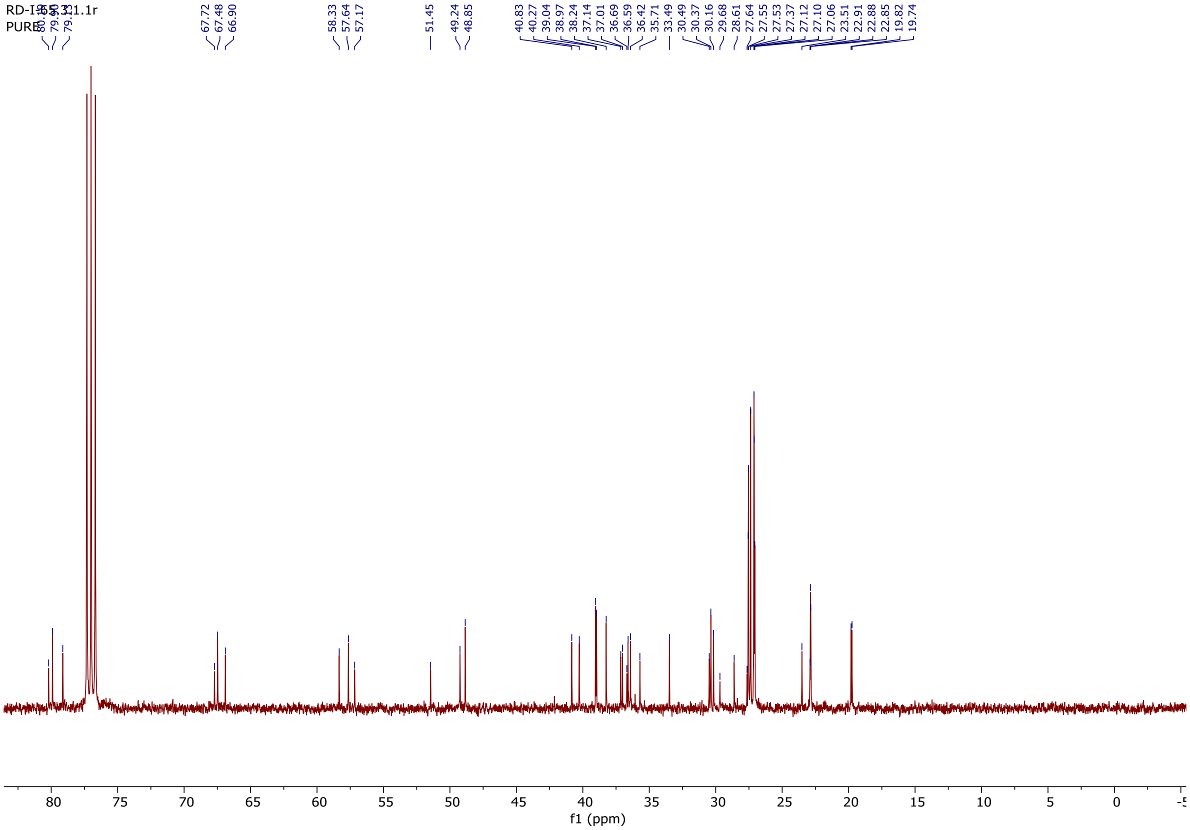

Compound 27 (^{1}H NMR: 400 MHz, ^{13}C NMR: 100 MHz)

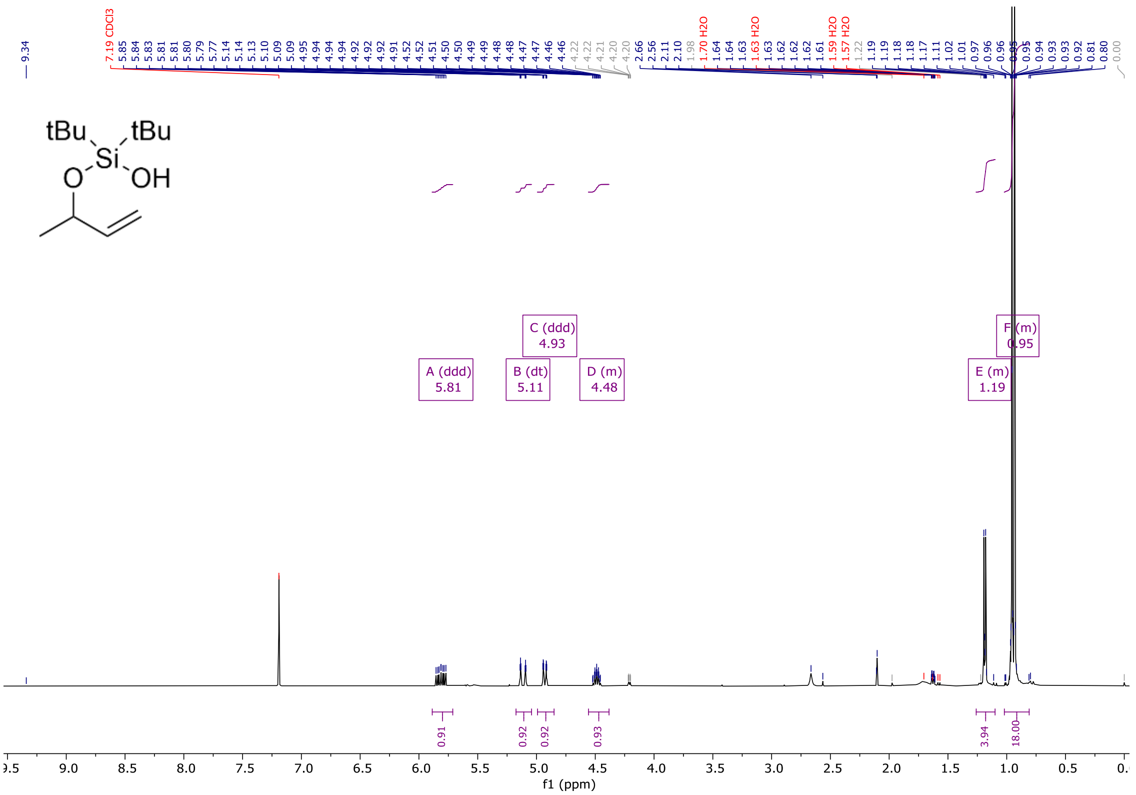

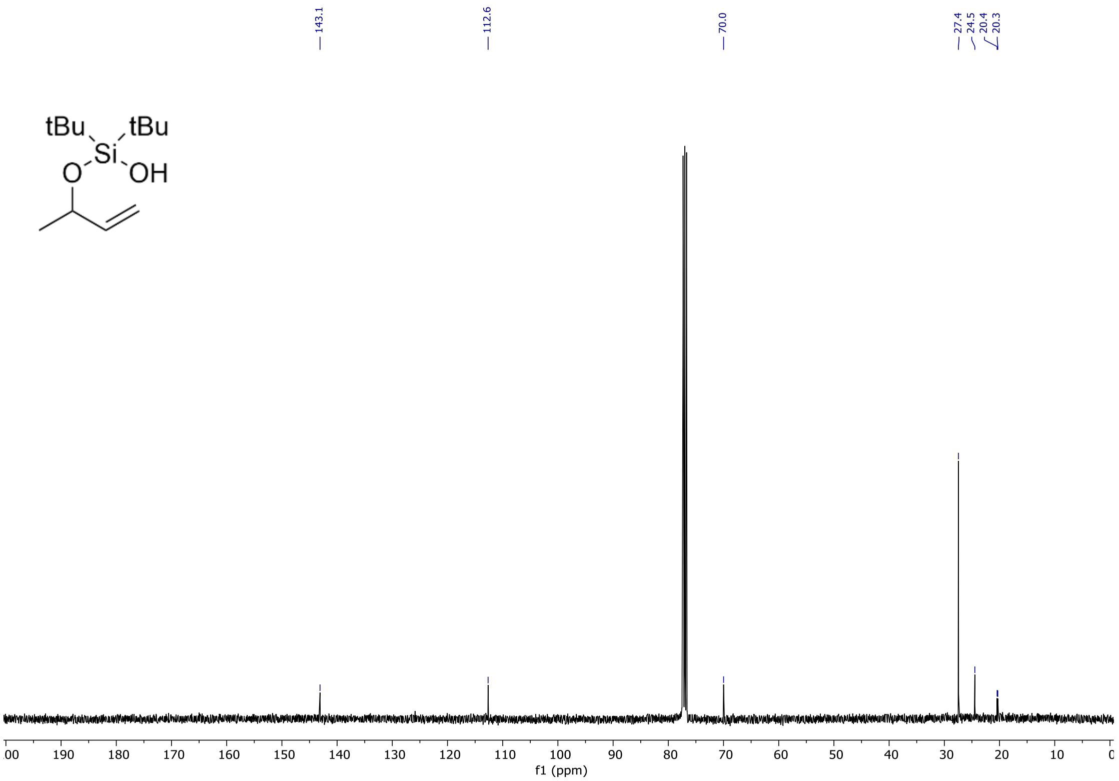

Compound 29 (^{1}H NMR: 400 MHz, ^{13}C NMR: 100 MHz)

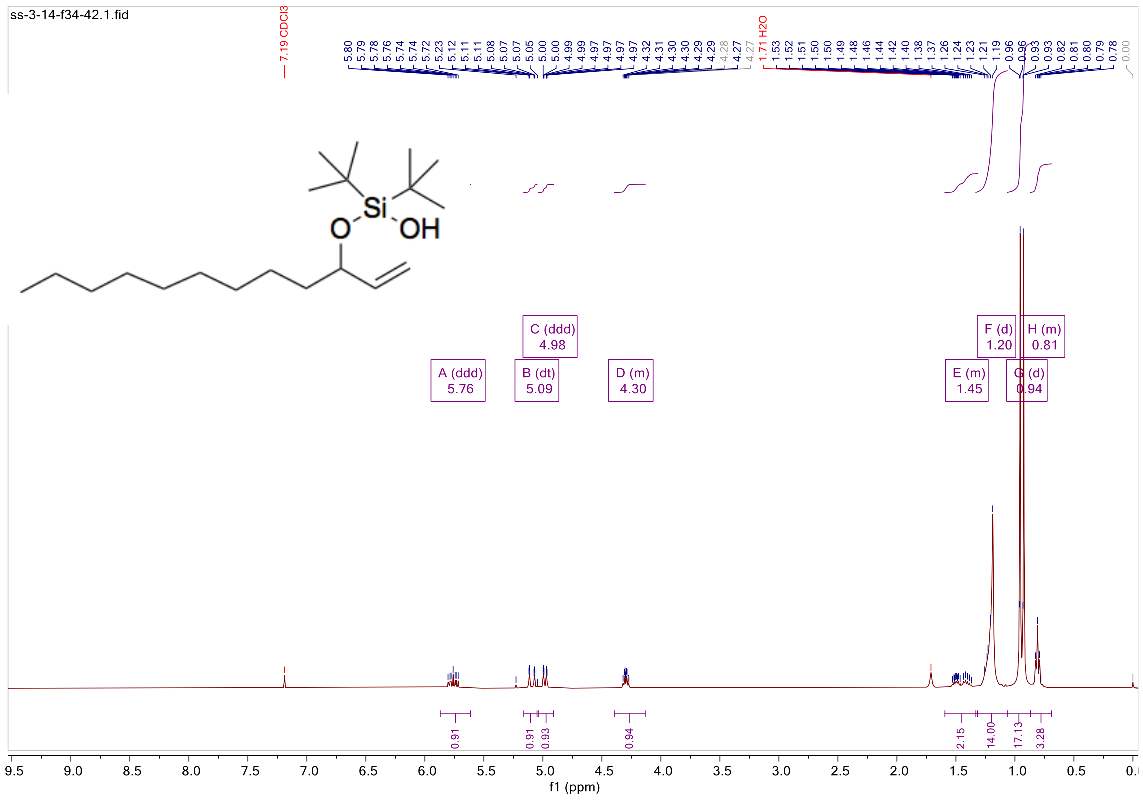

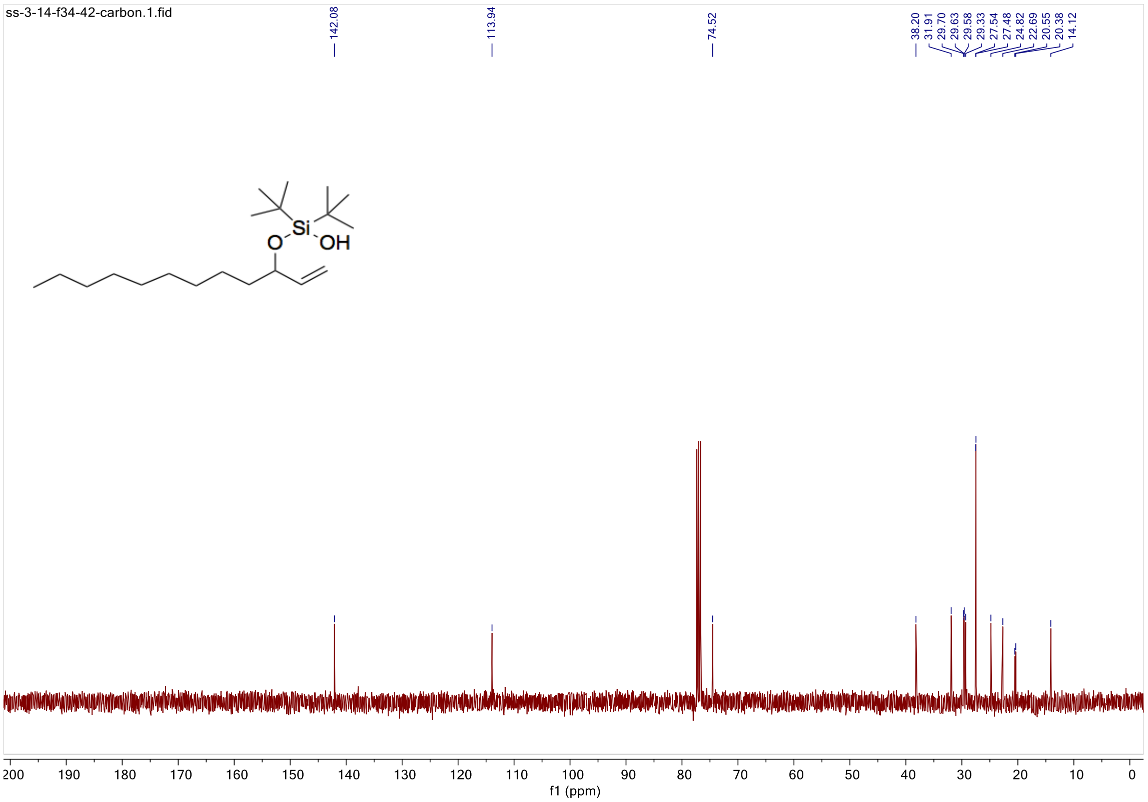

Compound 31 (^{1}H NMR: 400 MHz, ^{13}C NMR: 100 MHz)

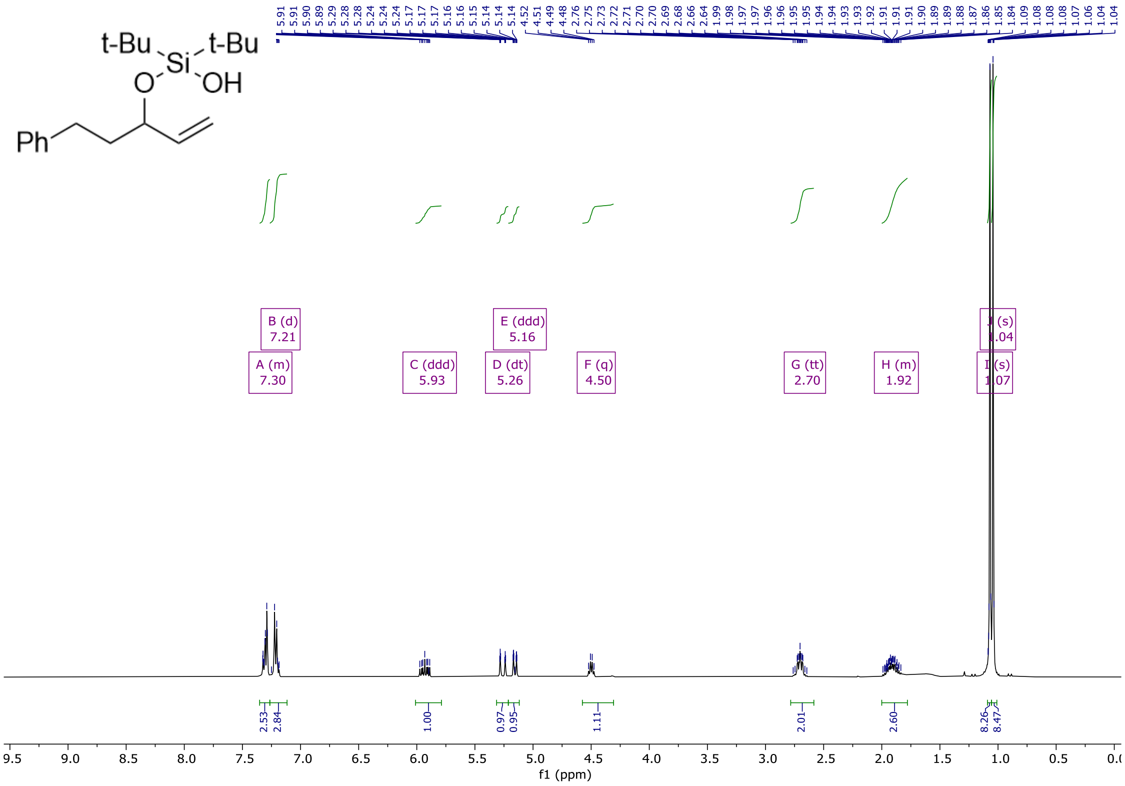

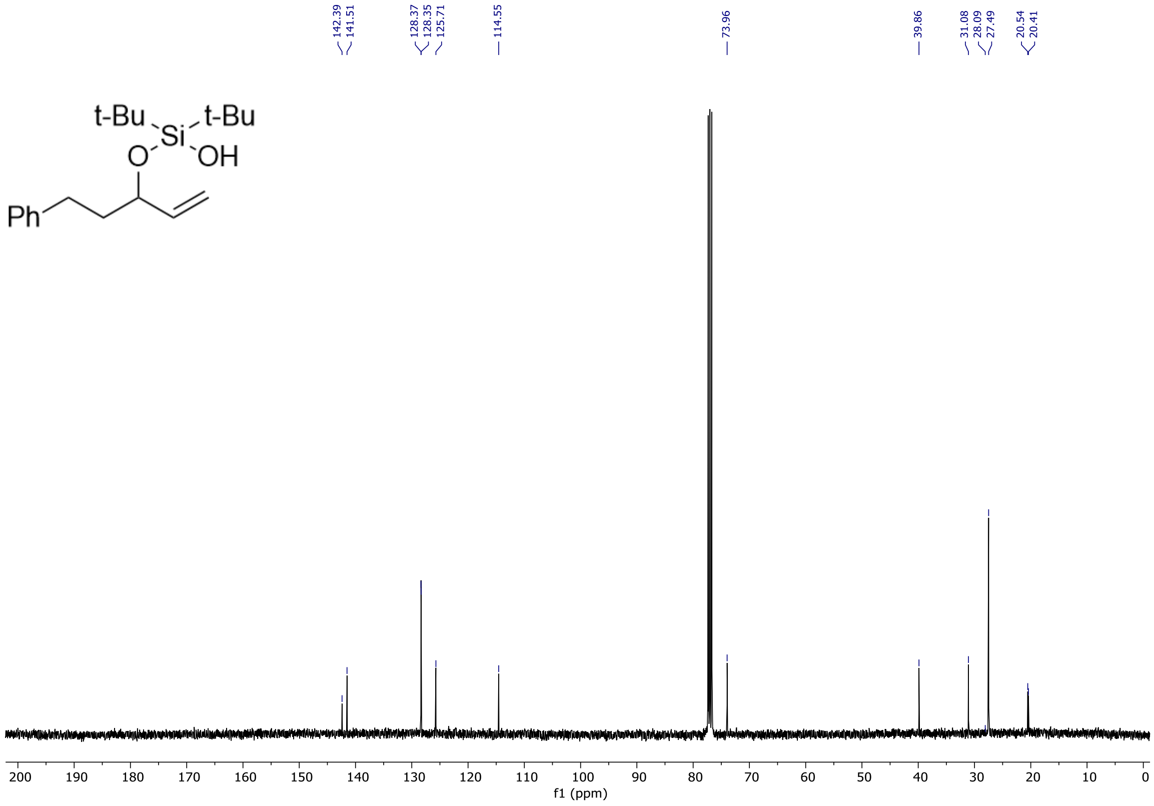

Compound 33 (^{1}H NMR: 400 MHz, ^{13}C NMR: 100 MHz)

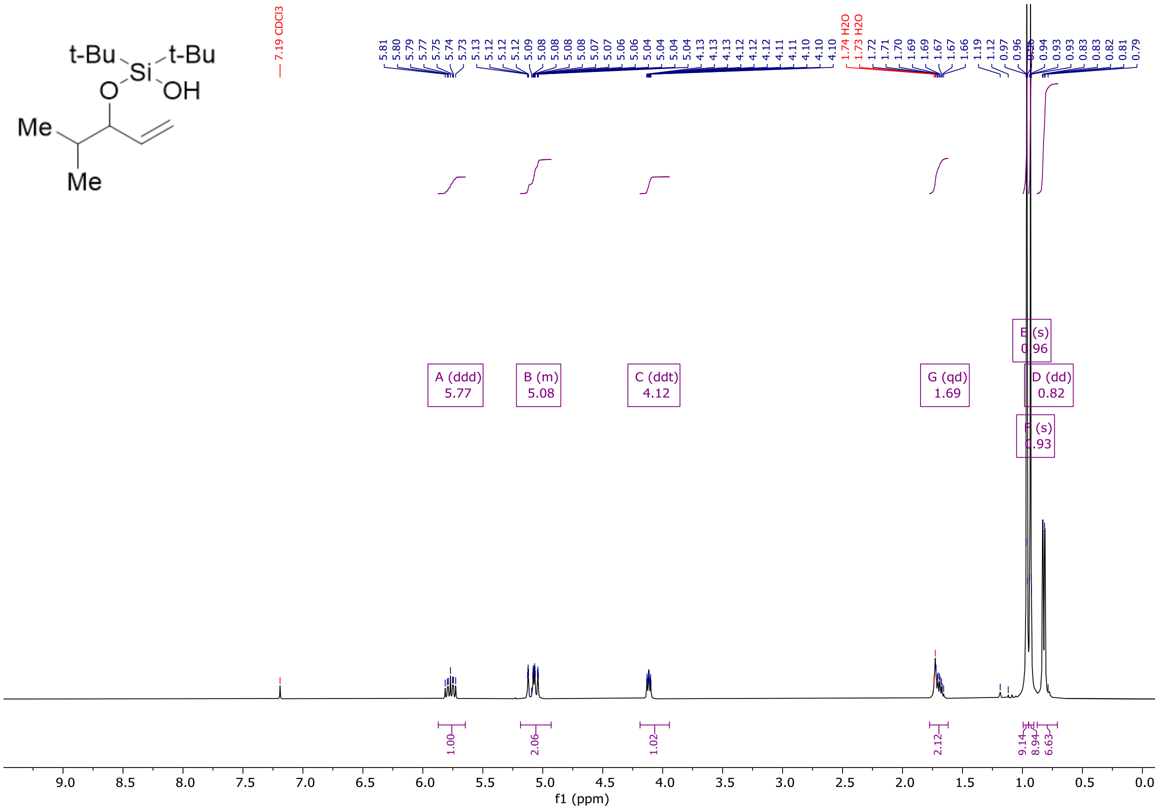

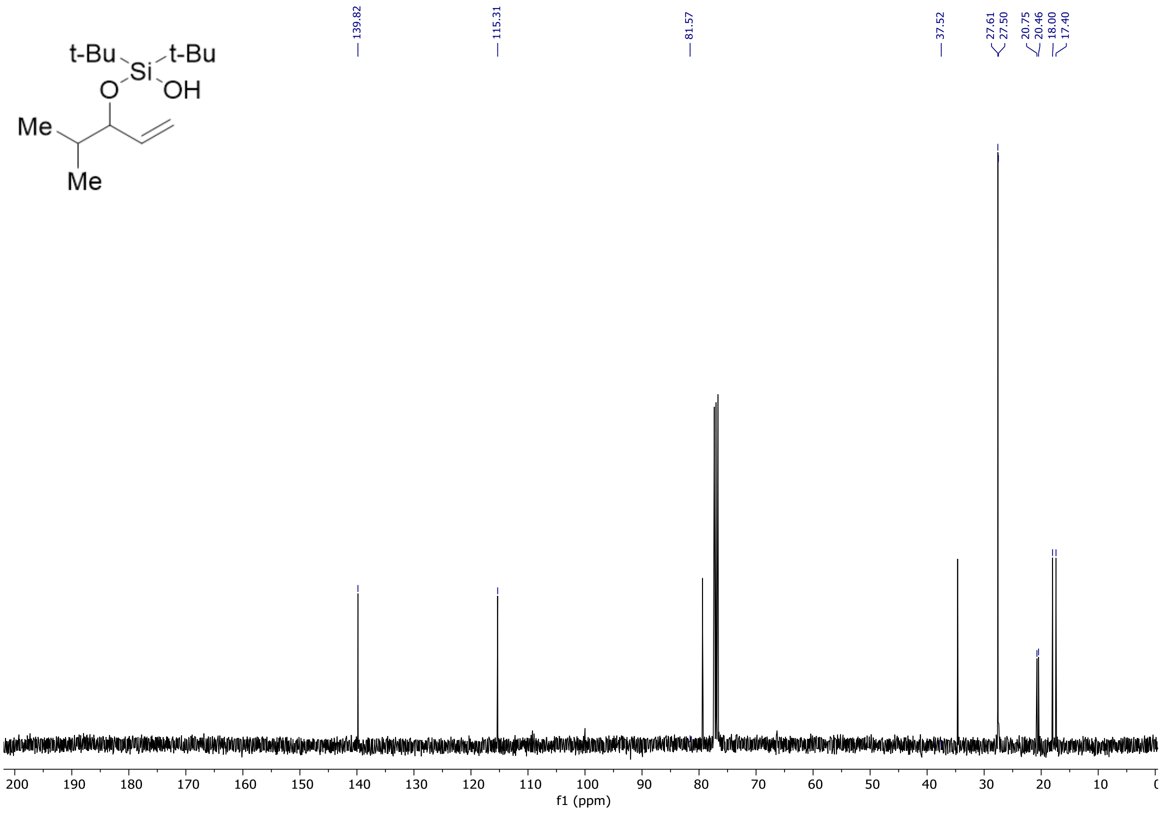

Compound 34 (^{1}H NMR: 400 MHz, ^{13}C NMR: 100 MHz)

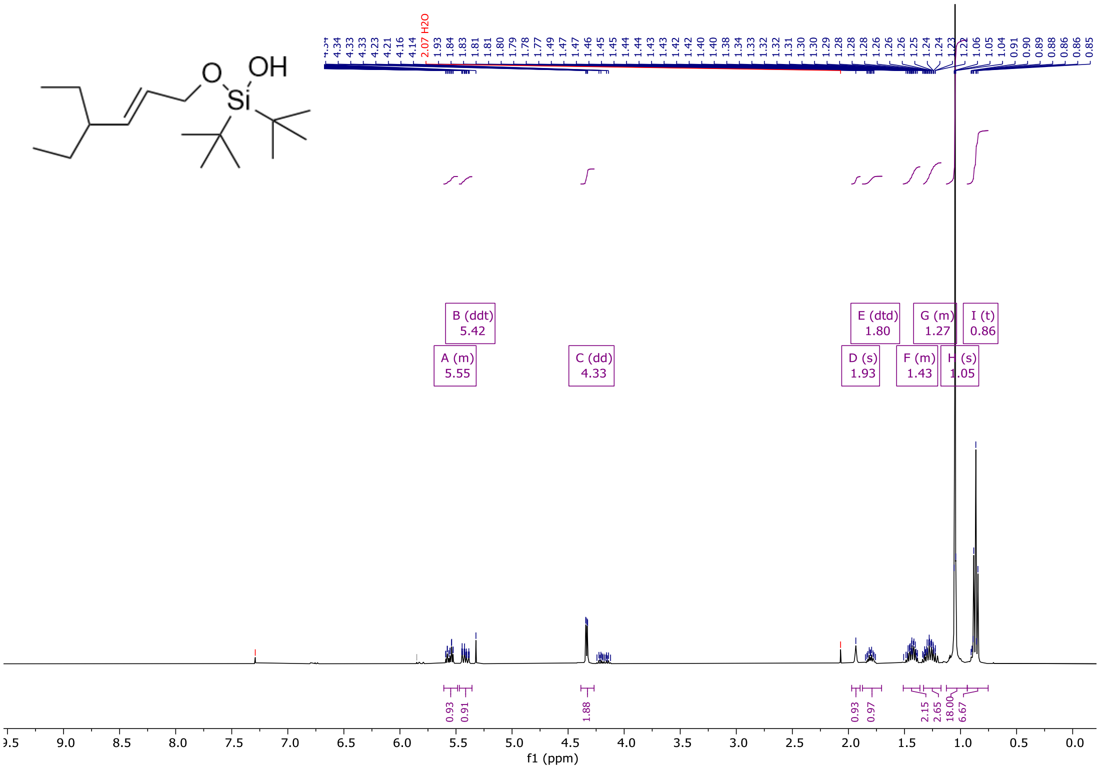

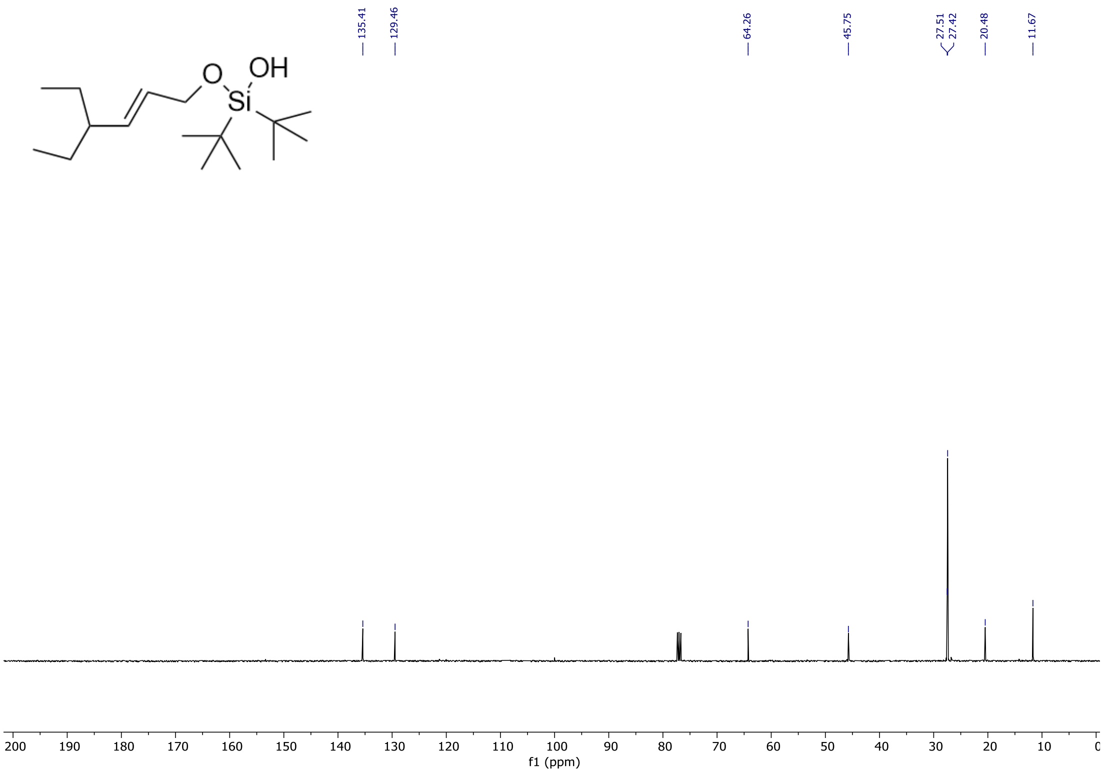

Compound 35 (^{1}H NMR: 400 MHz, ^{13}C NMR: 100 MHz)

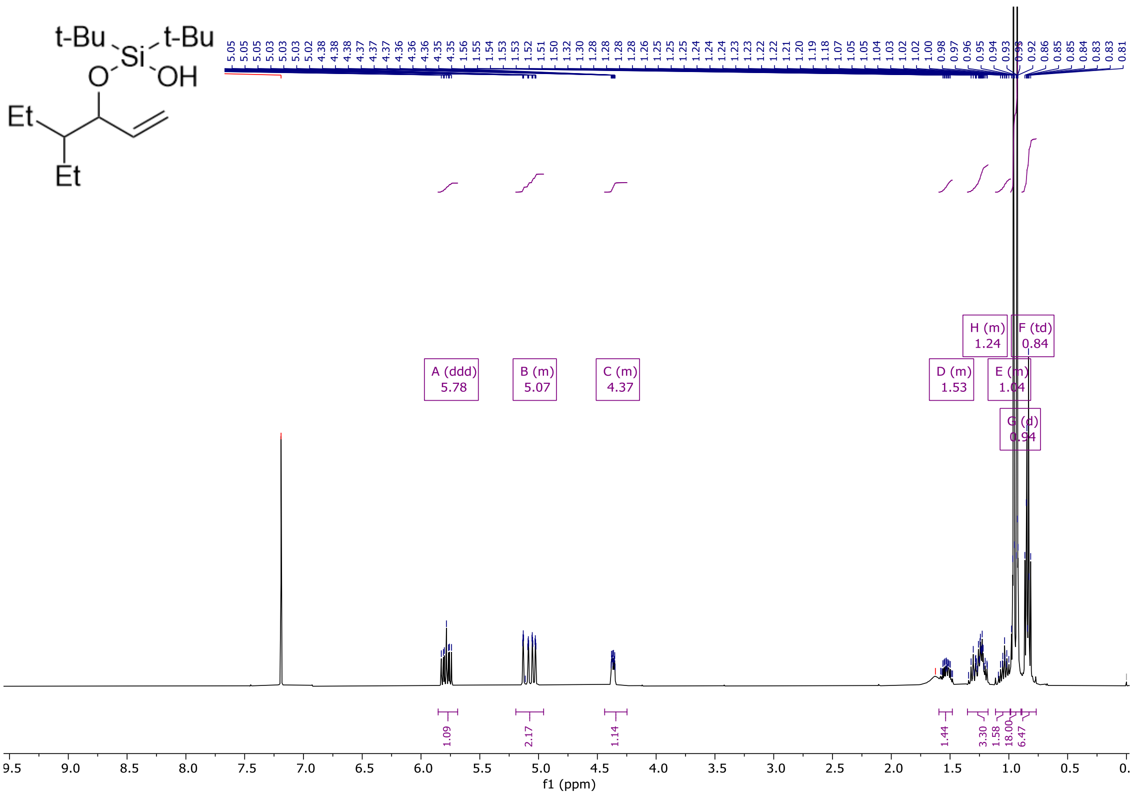

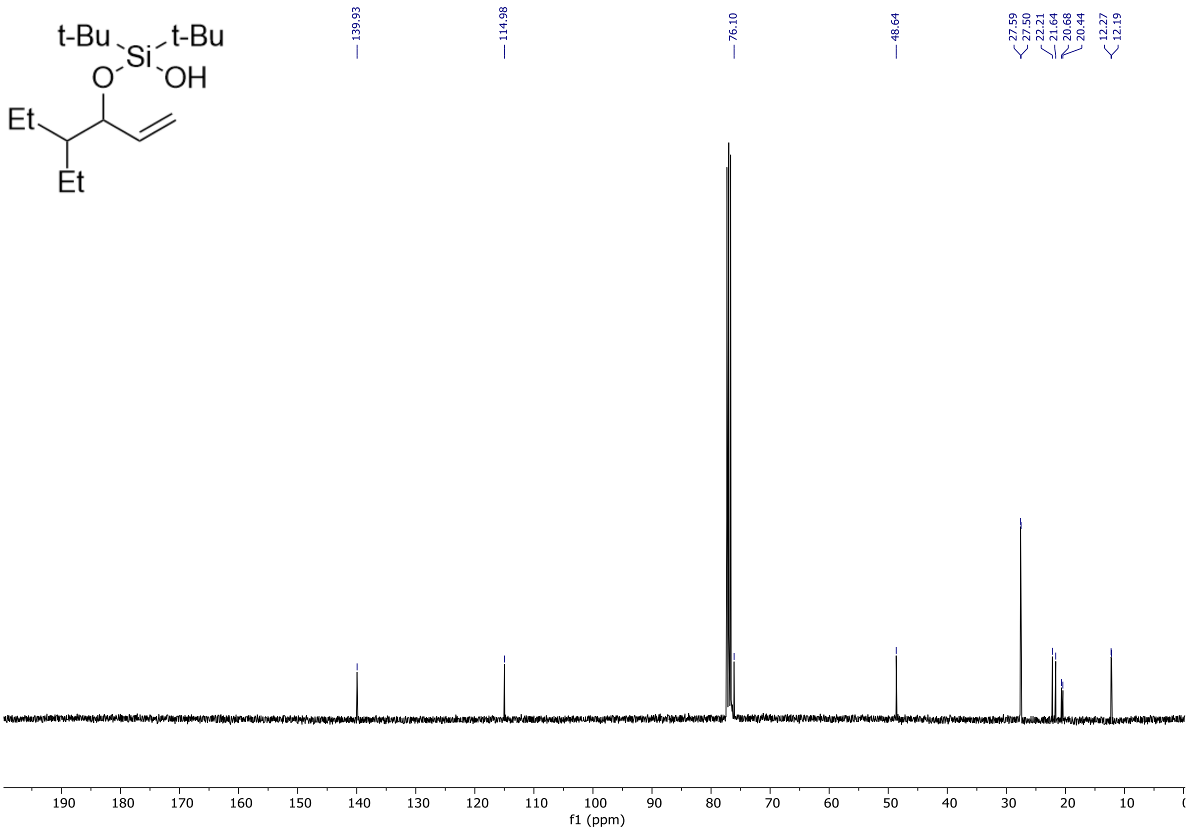

Compound 36 (^{1}H NMR: 400 MHz, ^{13}C NMR: 100 MHz)

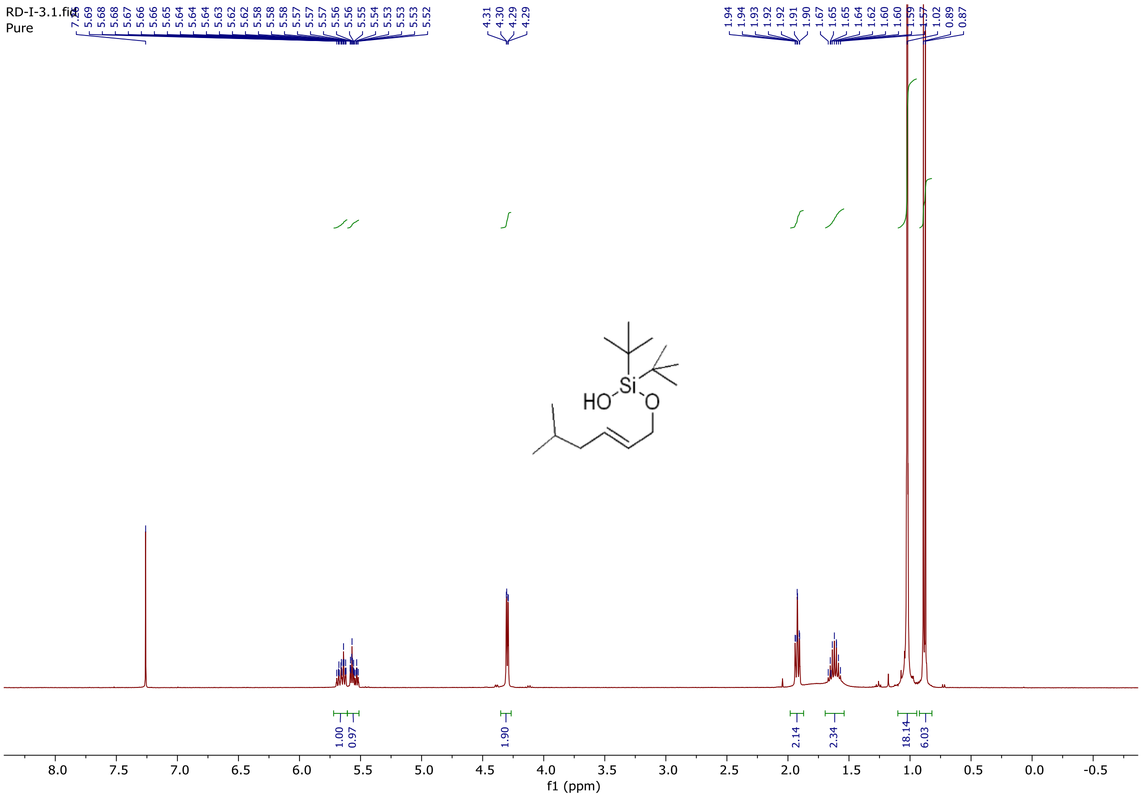

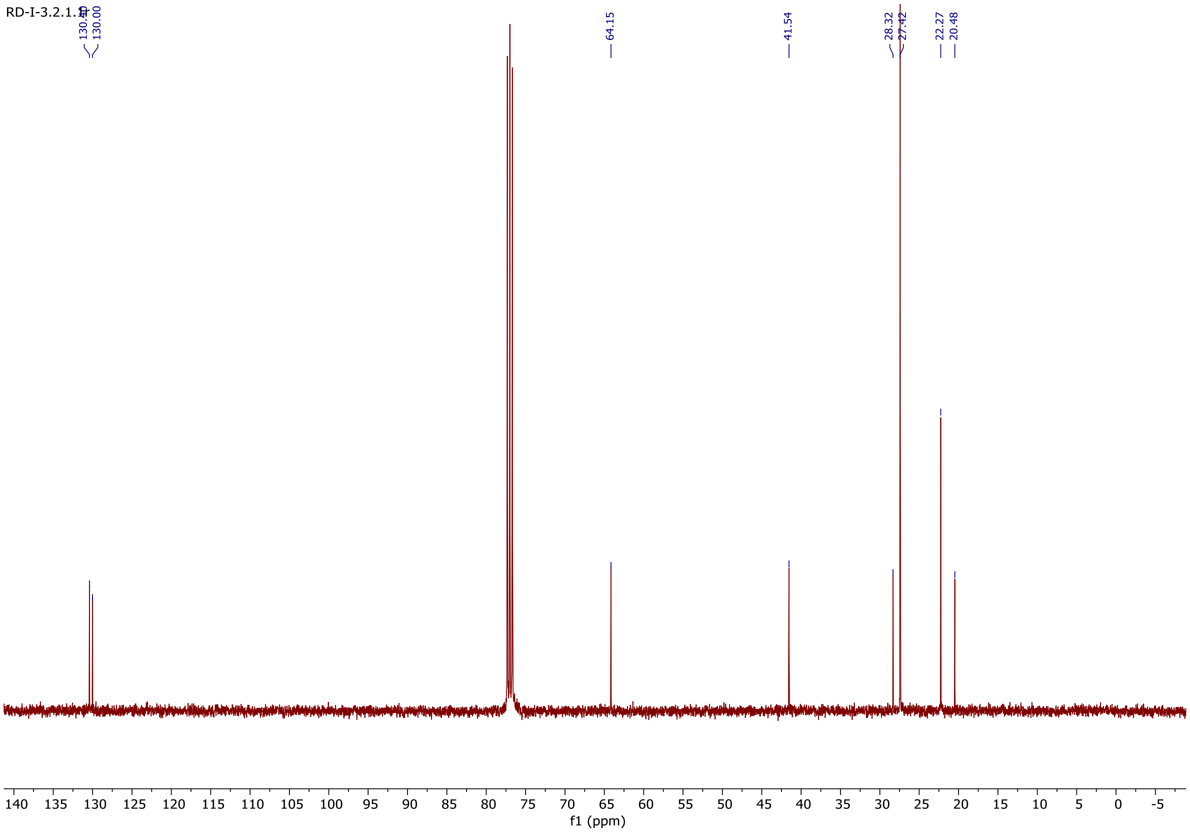

Compound 37 (^{1}H NMR: 400 MHz, ^{13}C NMR: 100 MHz)

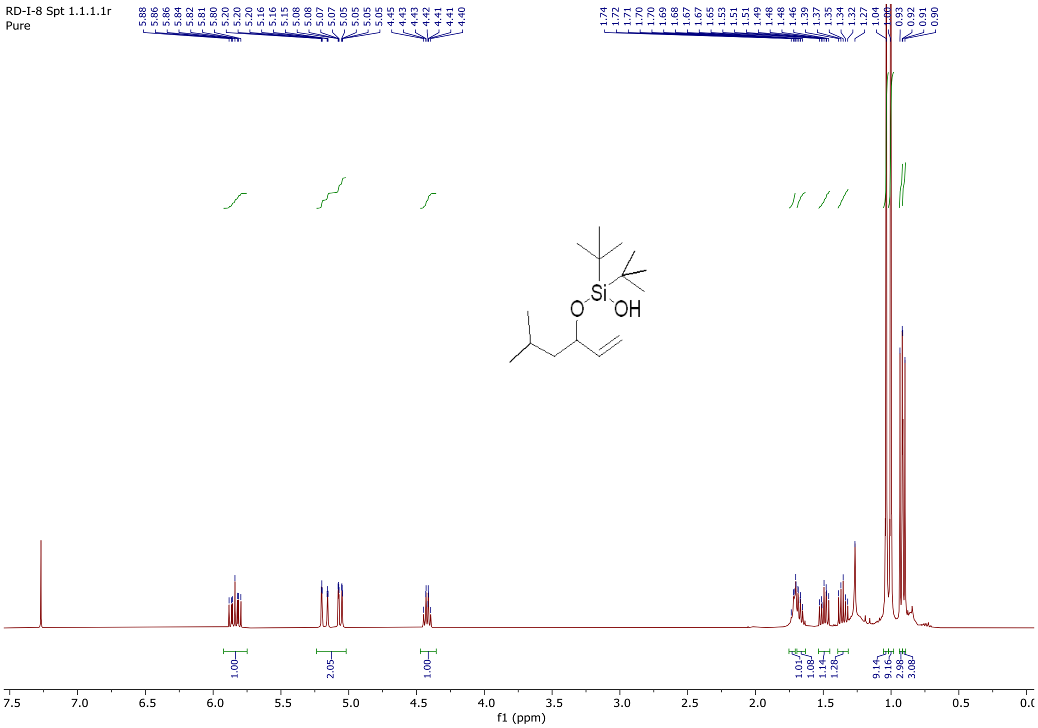

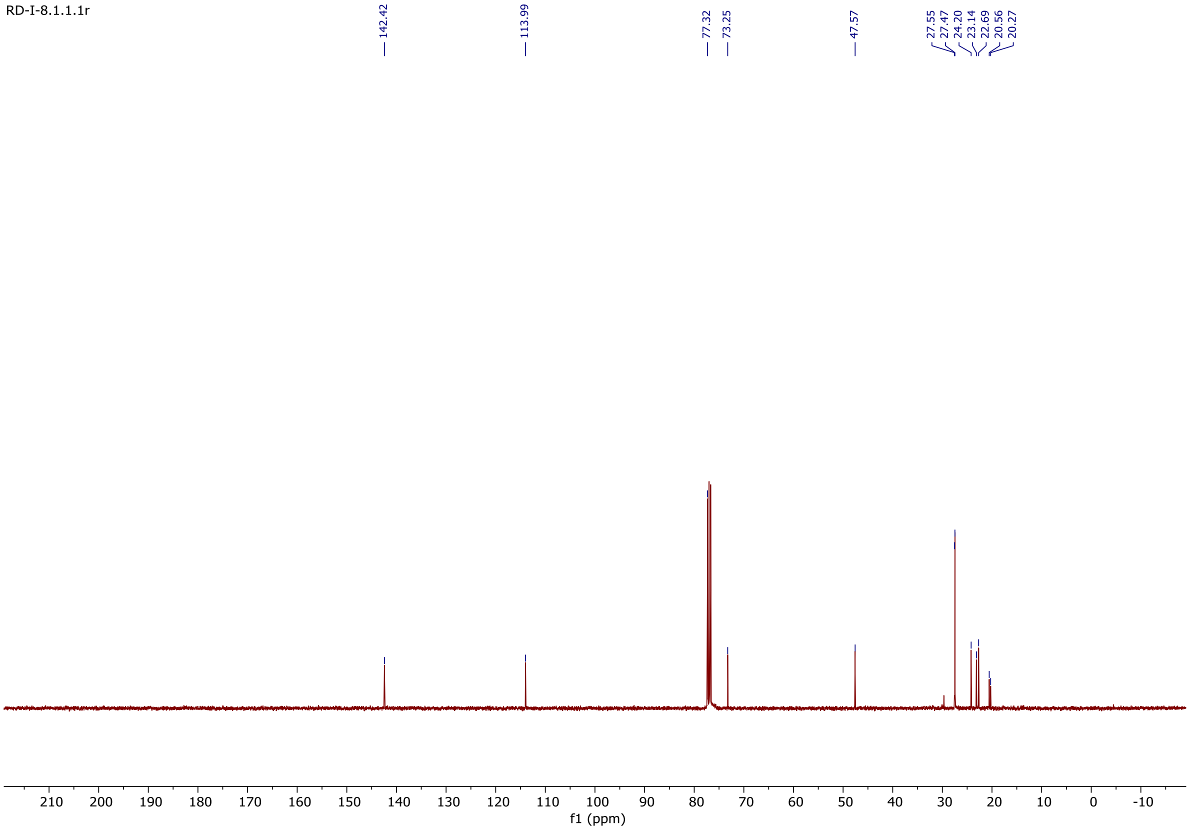

Compound 40 (^{1}H NMR: 400 MHz, ^{13}C NMR: 100 MHz)

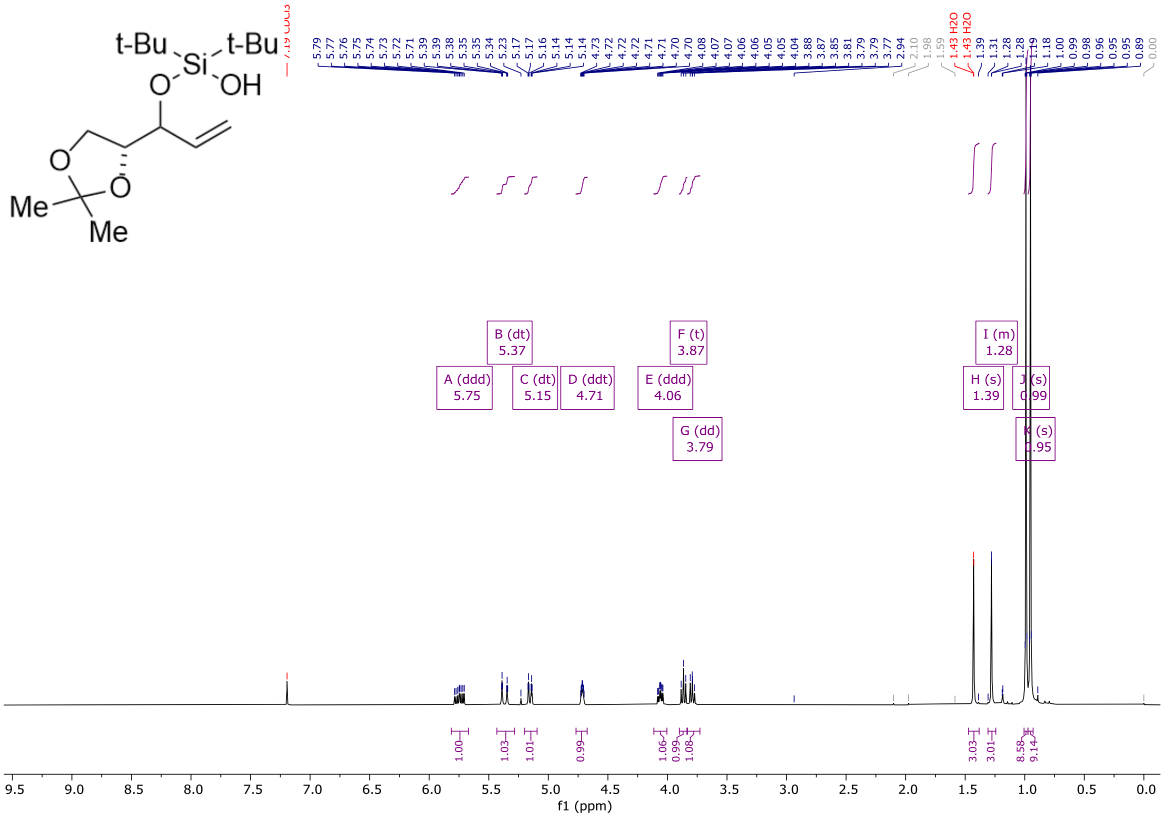

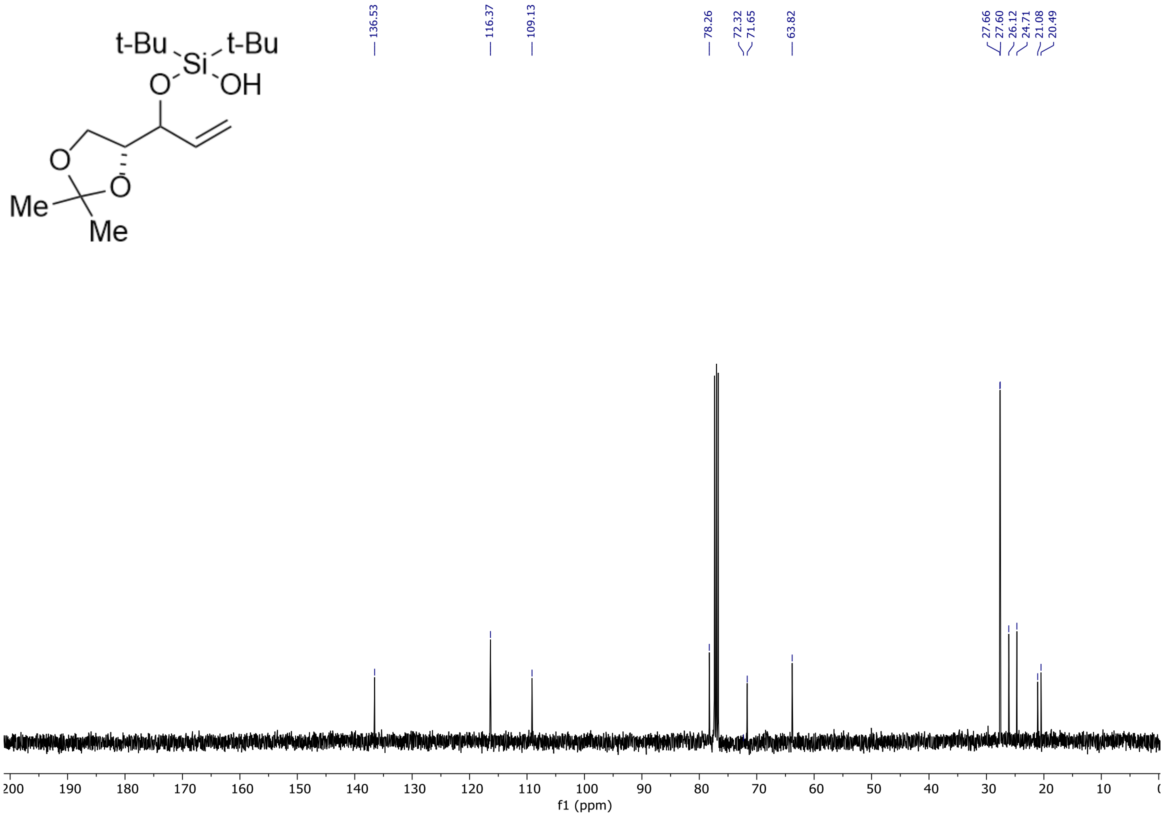

Compound 41 (^{1}H NMR: 400 MHz, ^{13}C NMR: 100 MHz)

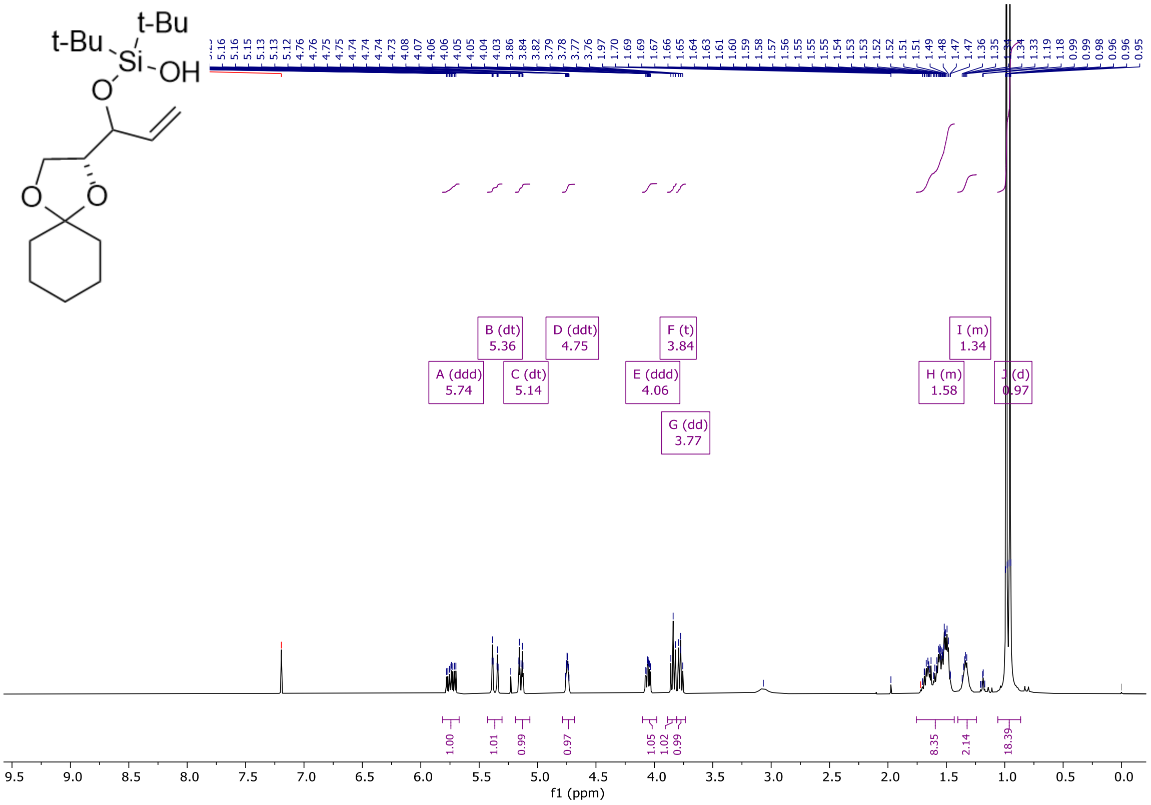

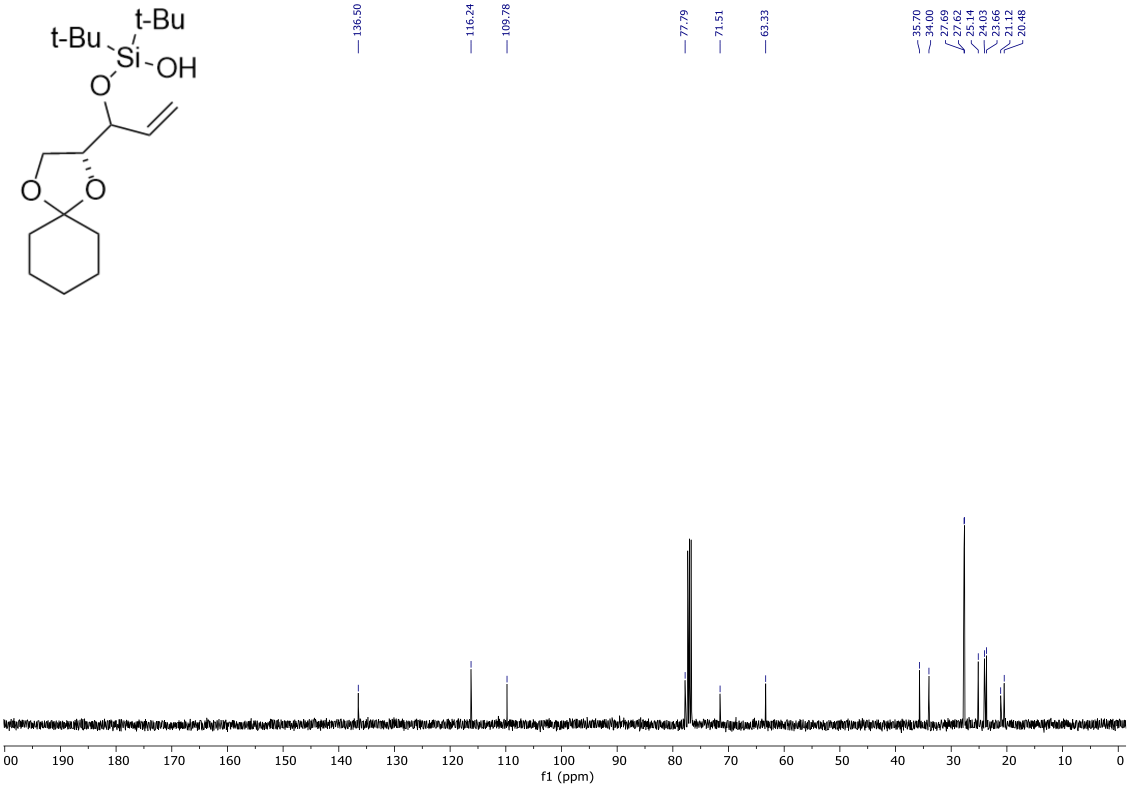

Compound 42 (^{1}H NMR: 400 MHz, ^{13}C NMR: 100 MHz)

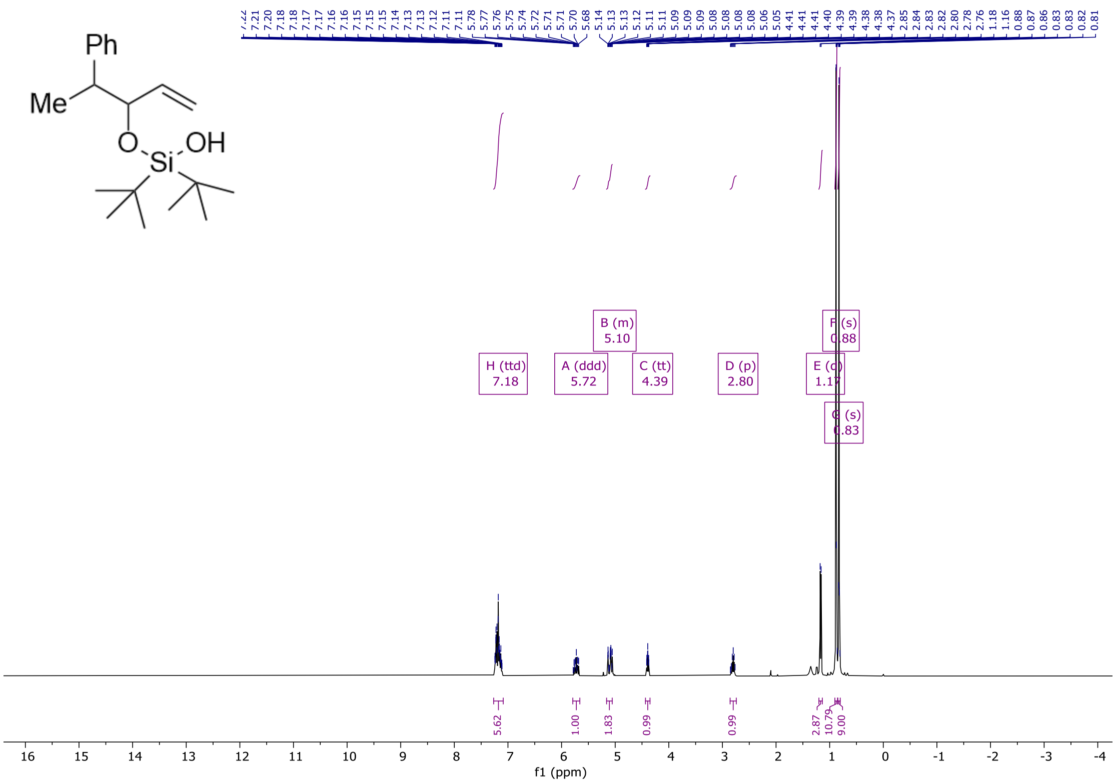

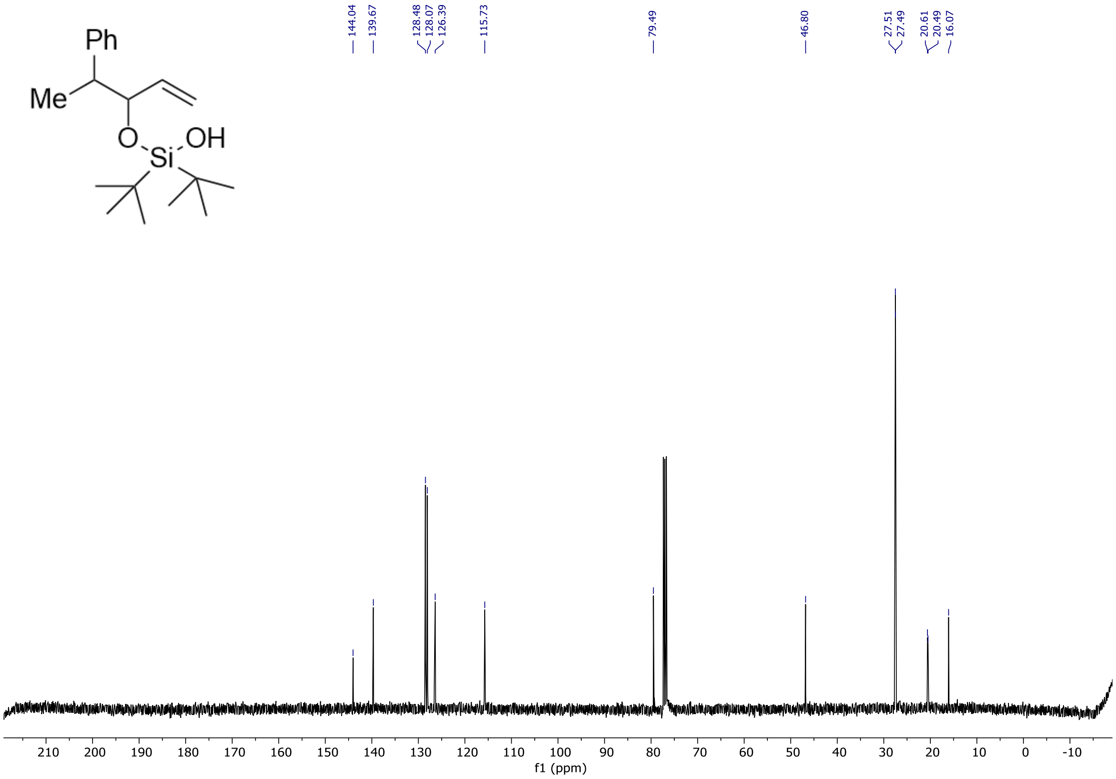

Compound 43 (^{1}H NMR: 400 MHz, ^{13}C NMR: 100 MHz)

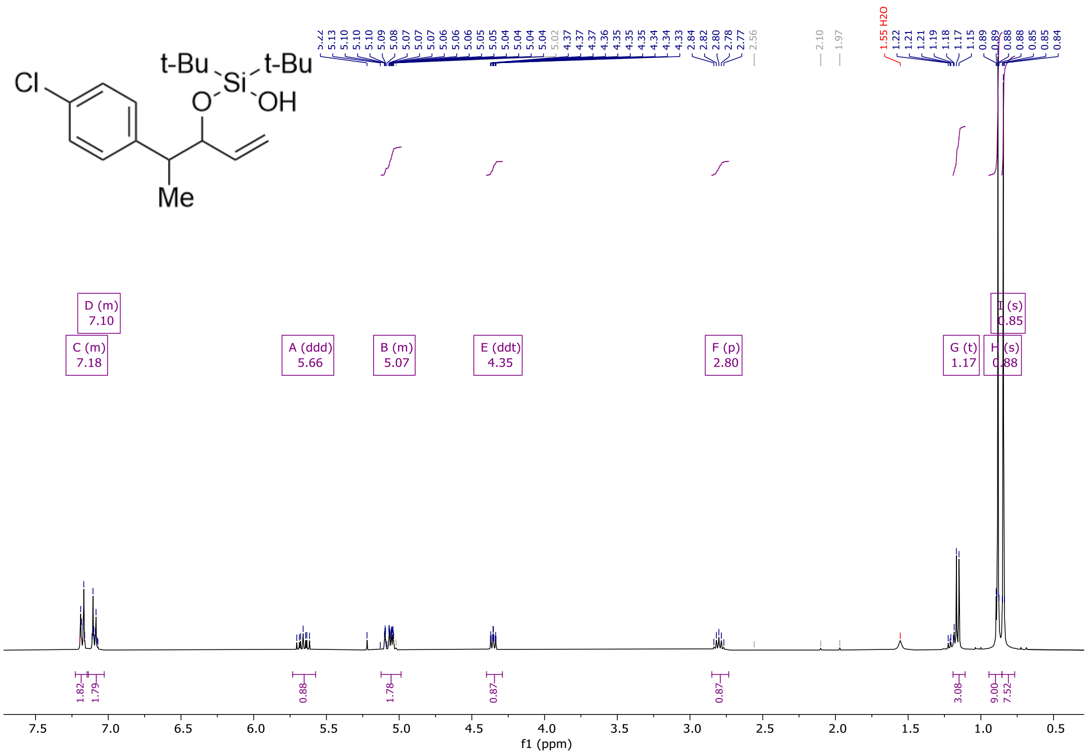

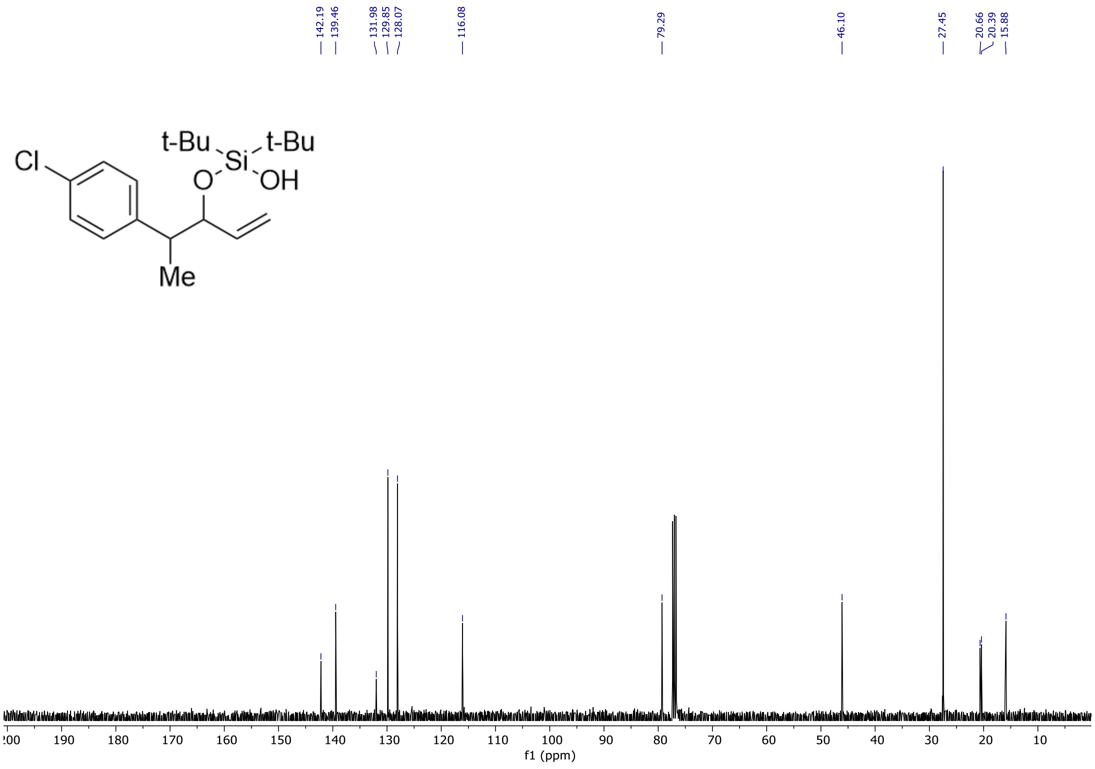

Compound 44 (^{1}H NMR: 400 MHz, ^{13}C NMR: 100 MHz)

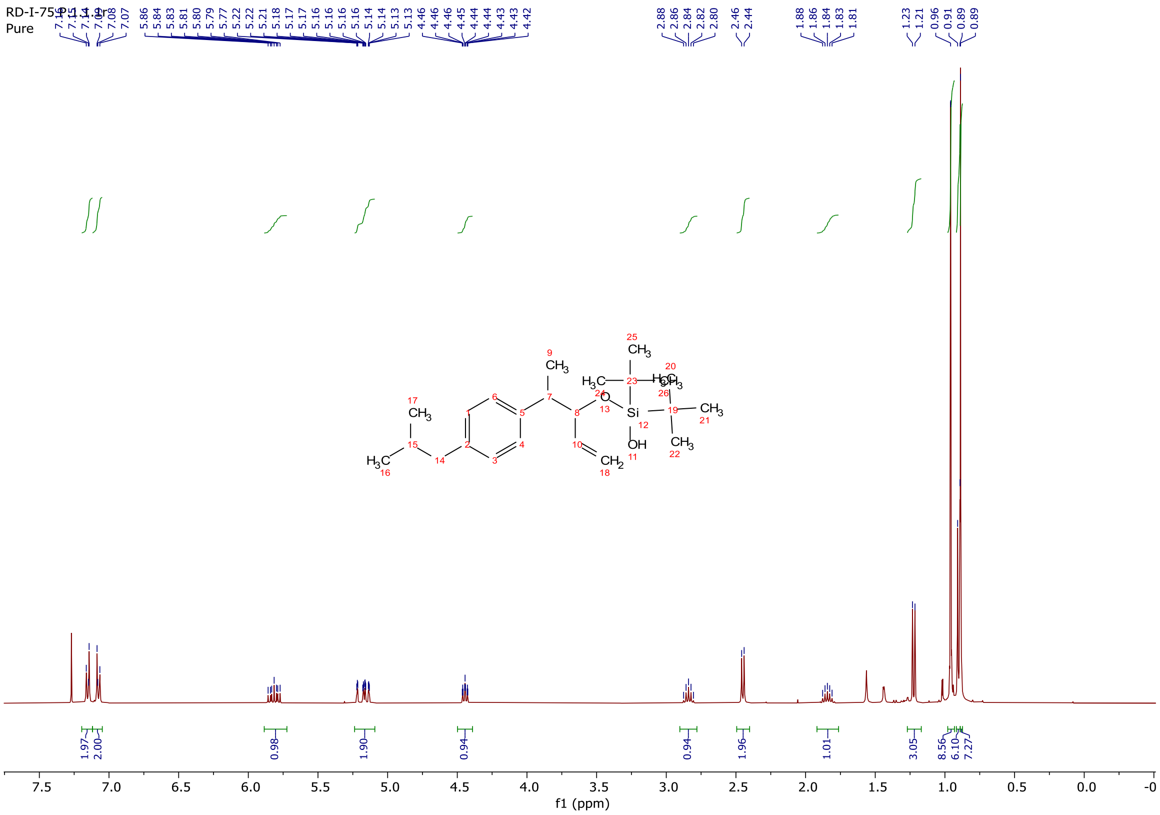

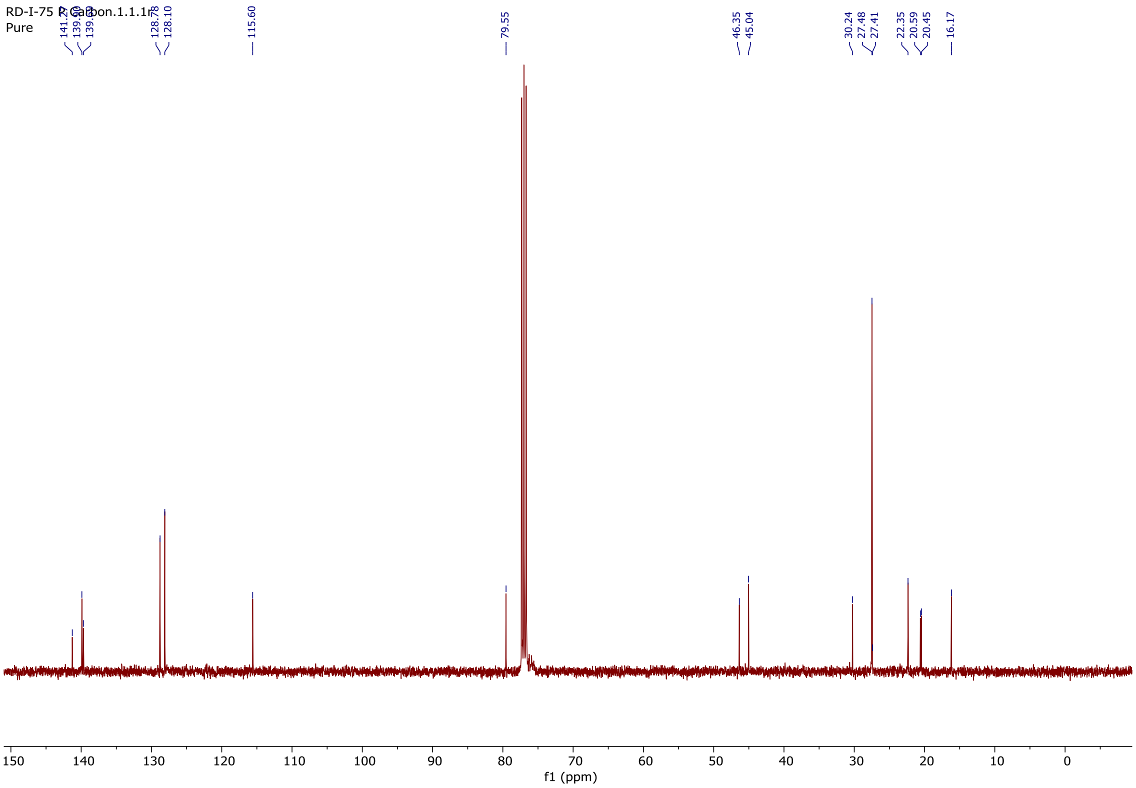

Compound 45 (^{1}H NMR: 400 MHz, ^{13}C NMR: 100 MHz)

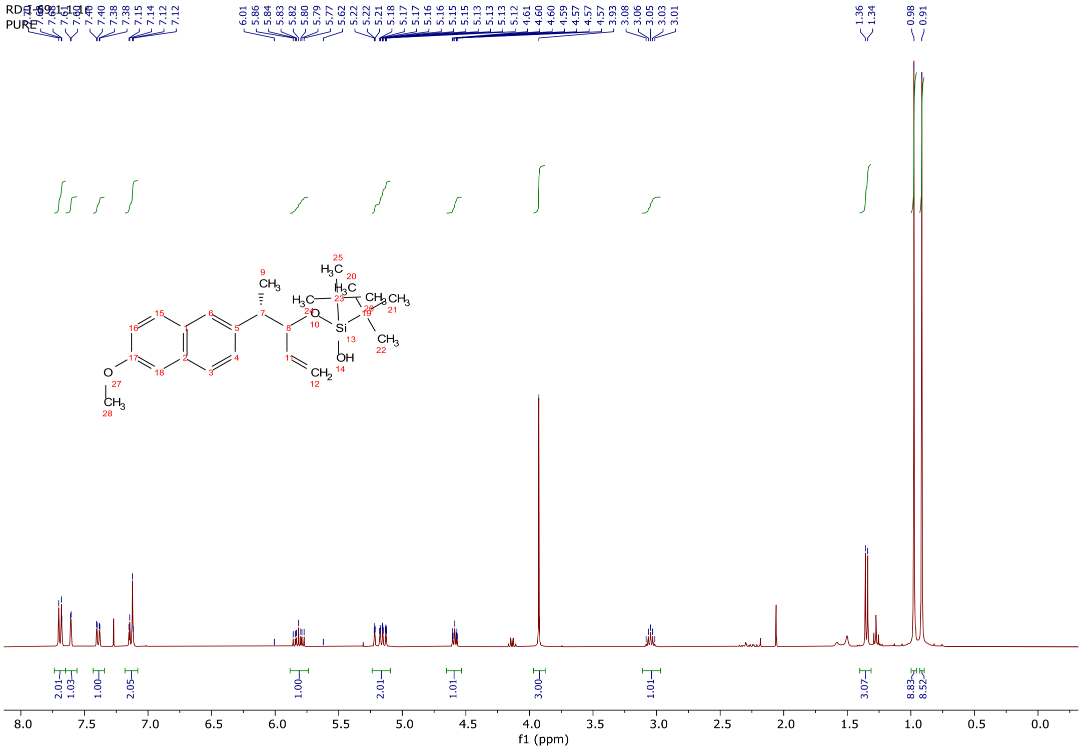

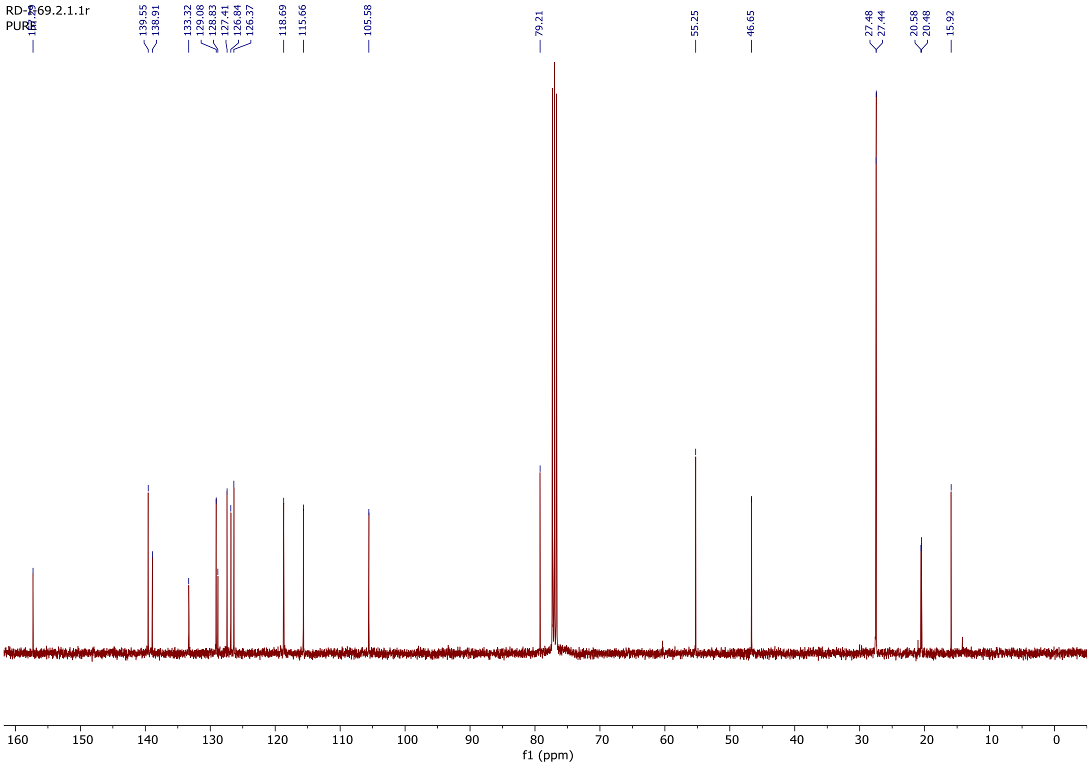

Compound 46 (^{1}H NMR: 400 MHz, ^{13}C NMR: 100 MHz)

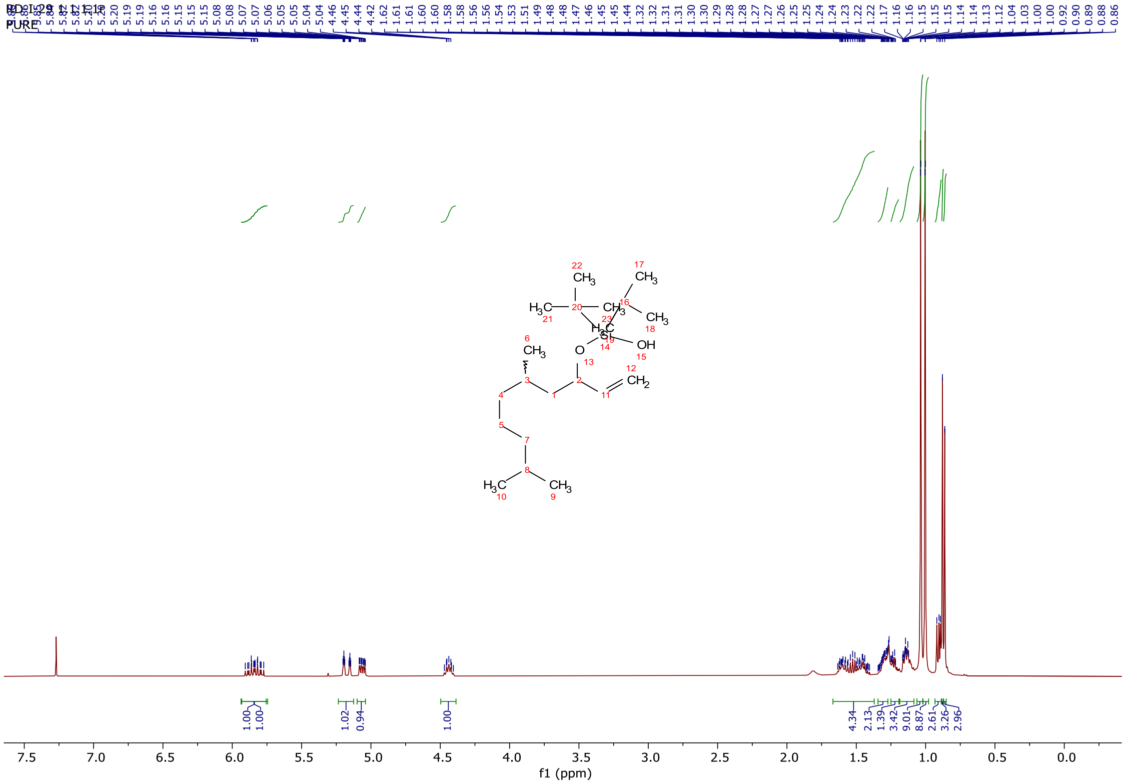

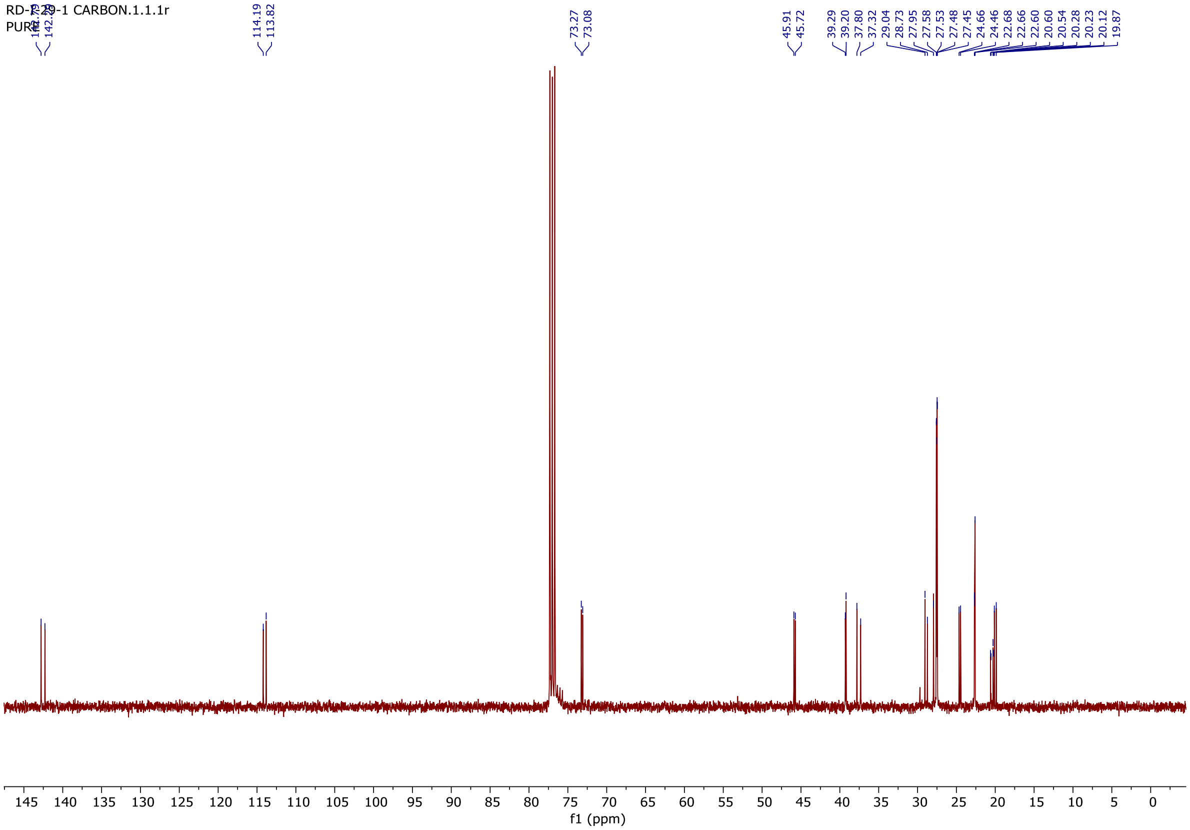

Compound 46 isomer (^{1}H NMR: 400 MHz, ^{13}C NMR: 100 MHz)

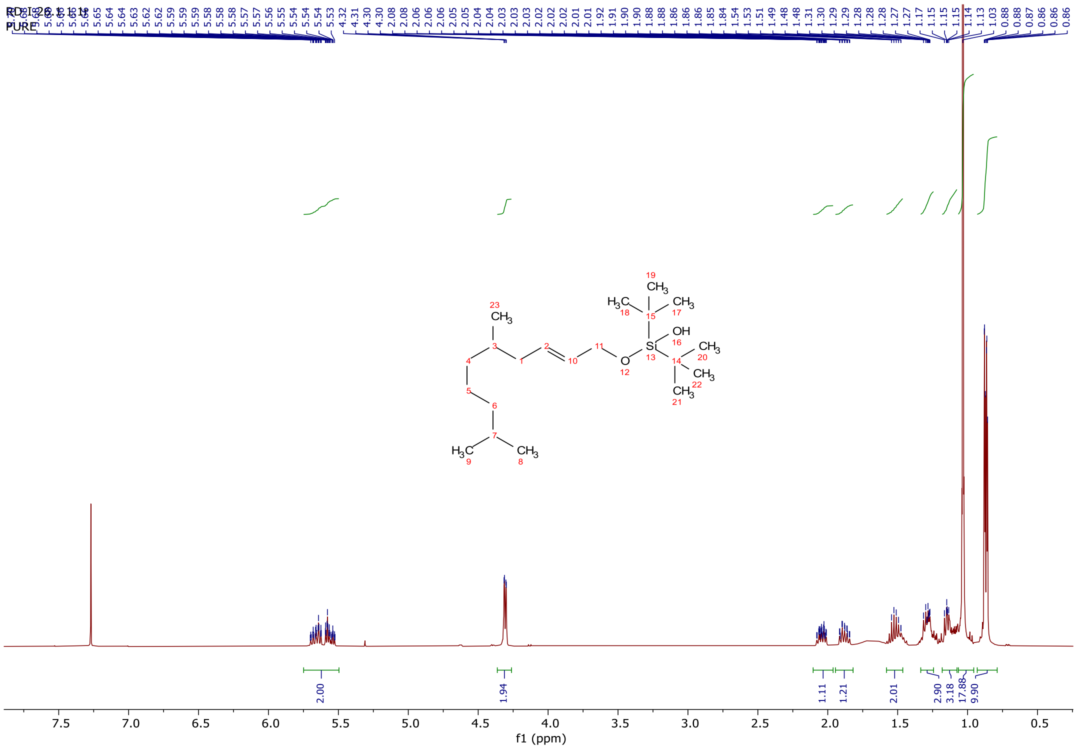

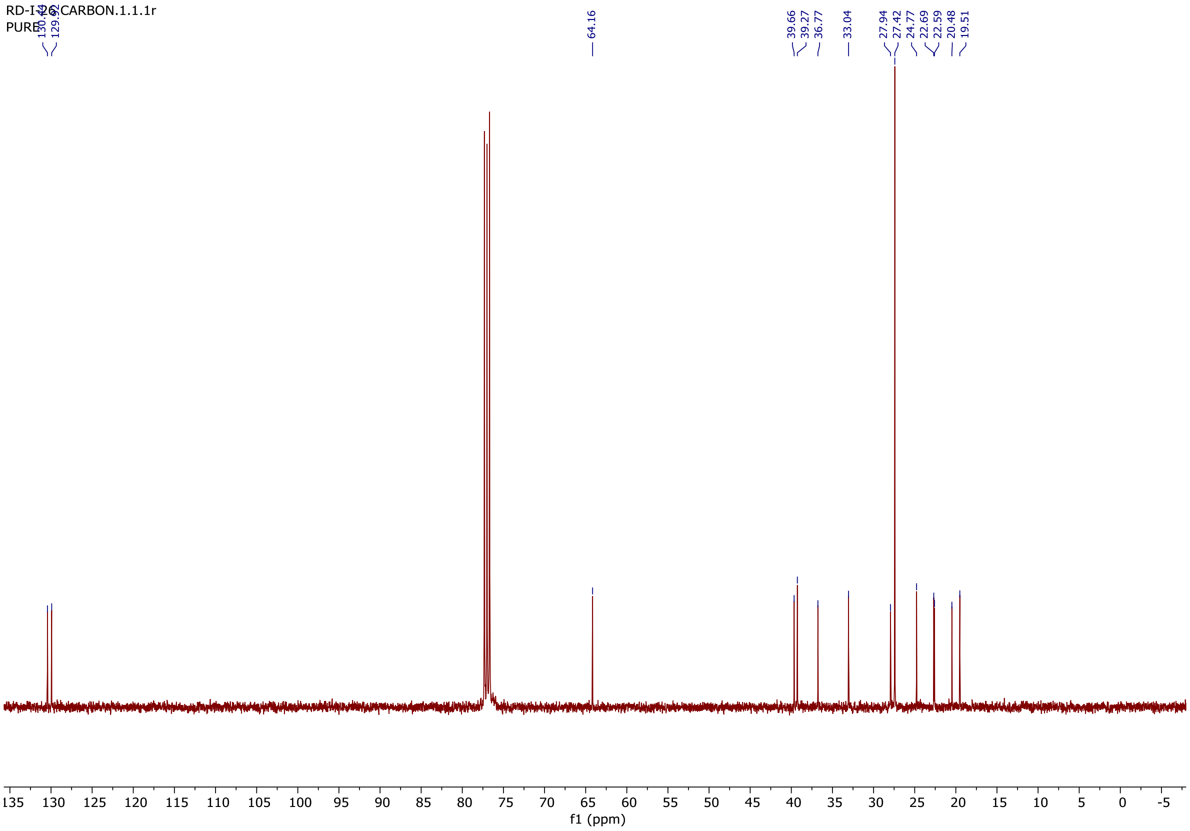

Compound 47 (^{1}H NMR: 400 MHz, ^{13}C NMR: 100 MHz)

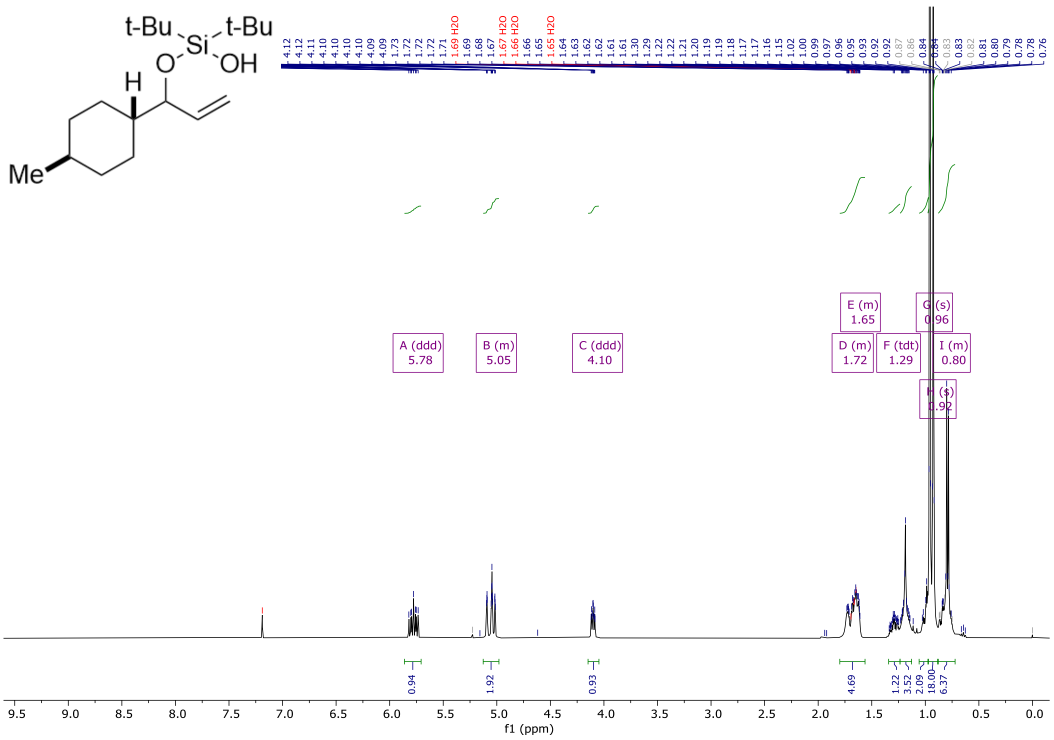

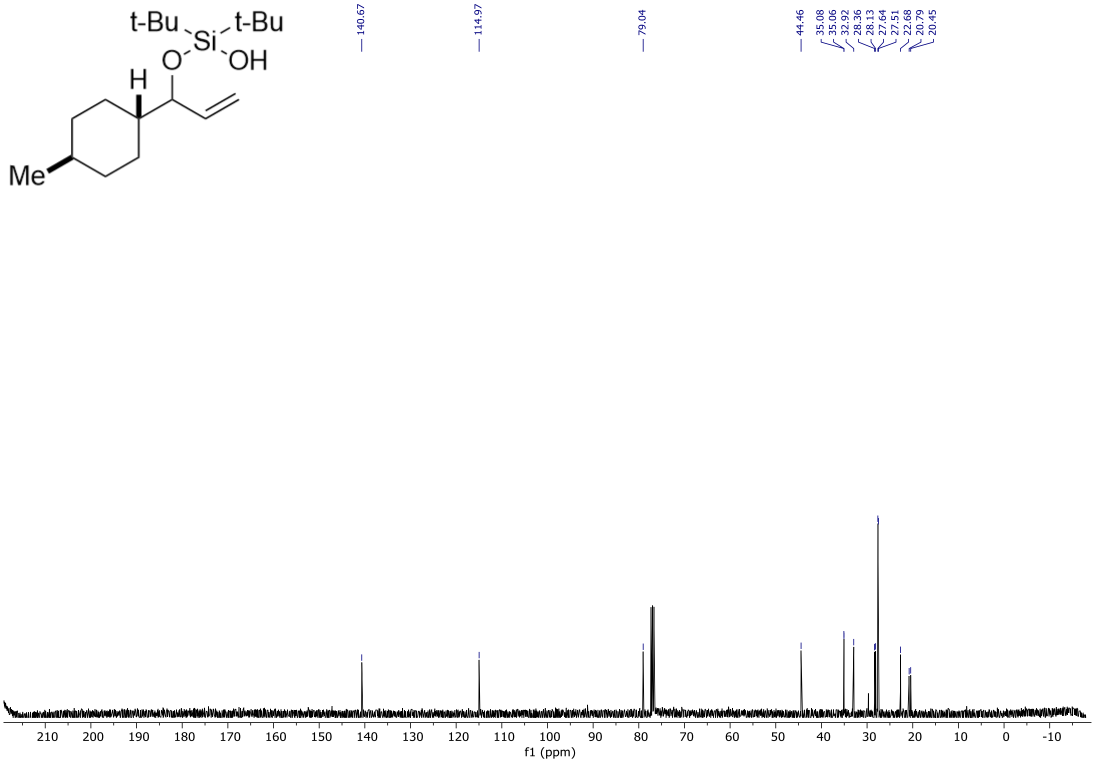

Compound 47 isomer (^{1}H NMR: 400 MHz, ^{13}C NMR: 100 MHz)

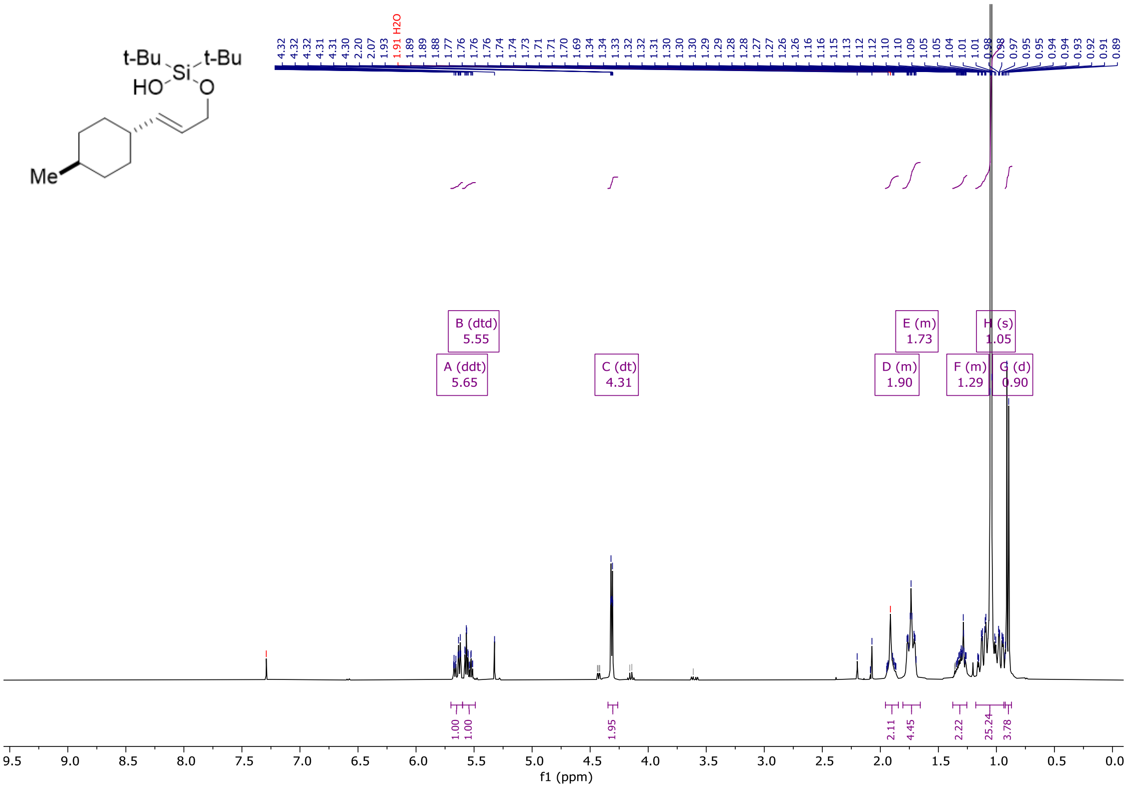

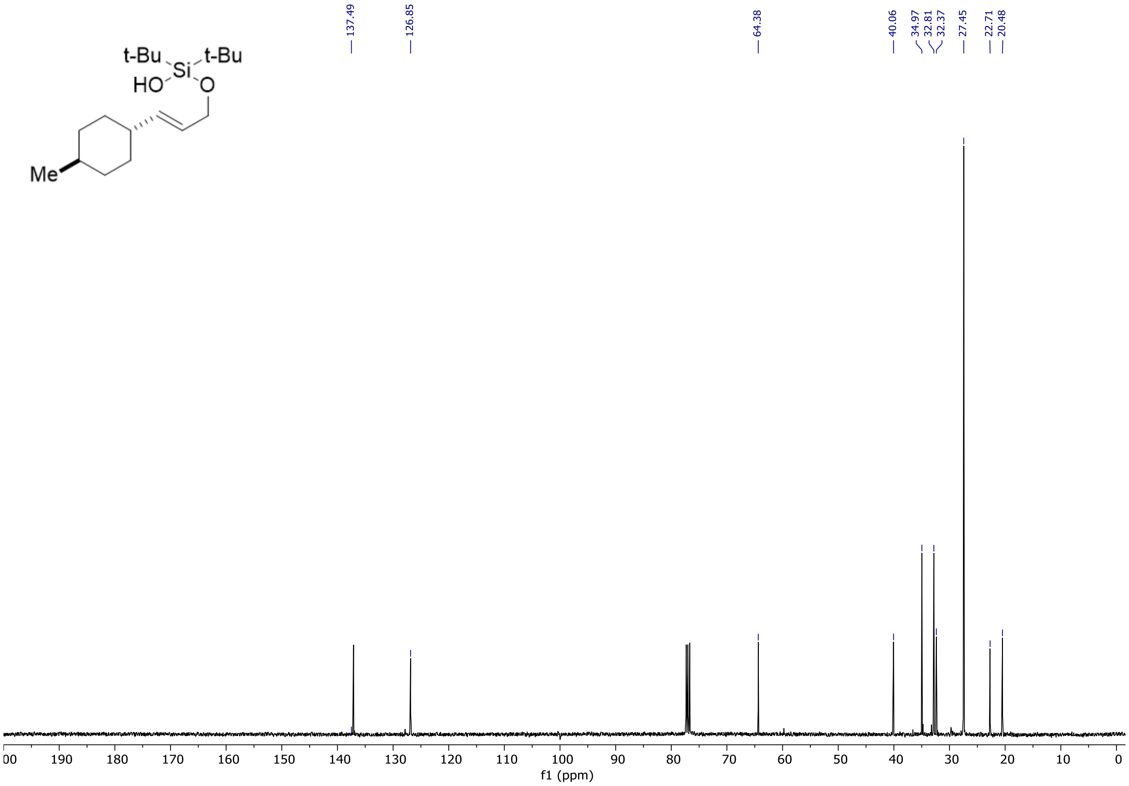

Compound 48 (^{1}H NMR: 400 MHz, ^{13}C NMR: 100 MHz)

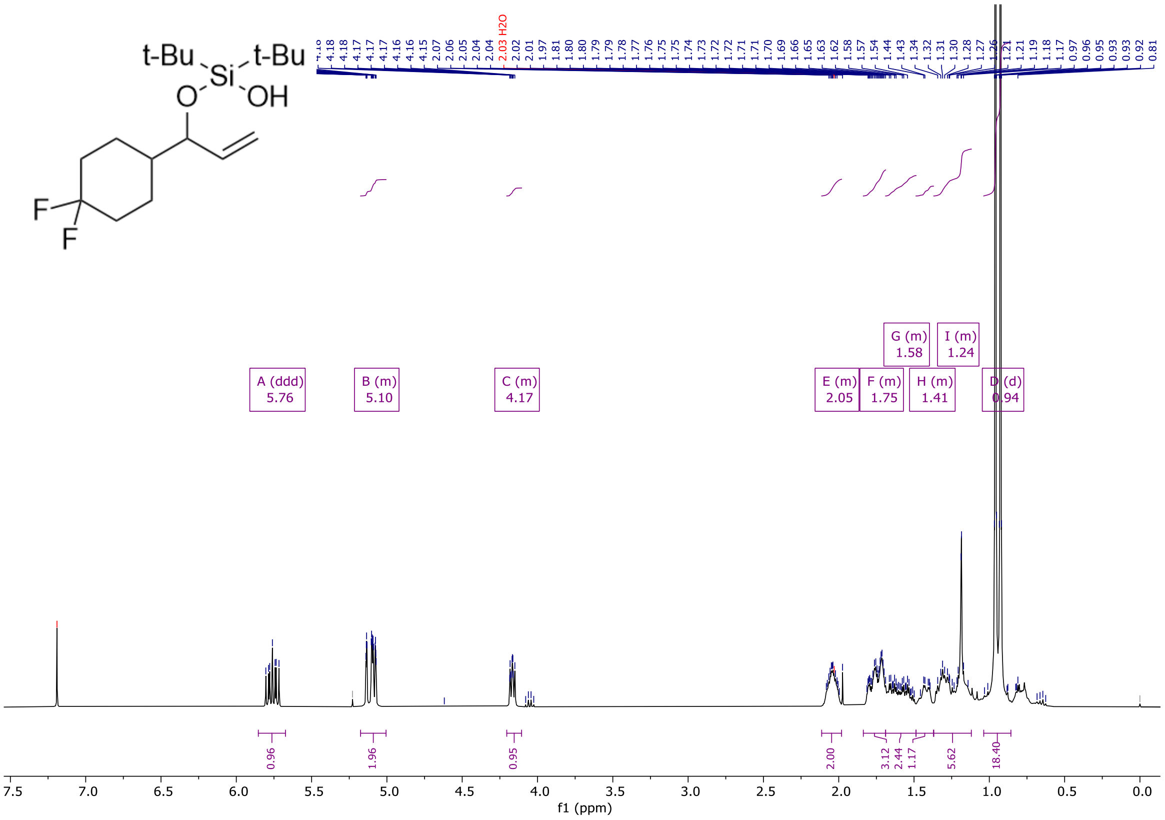

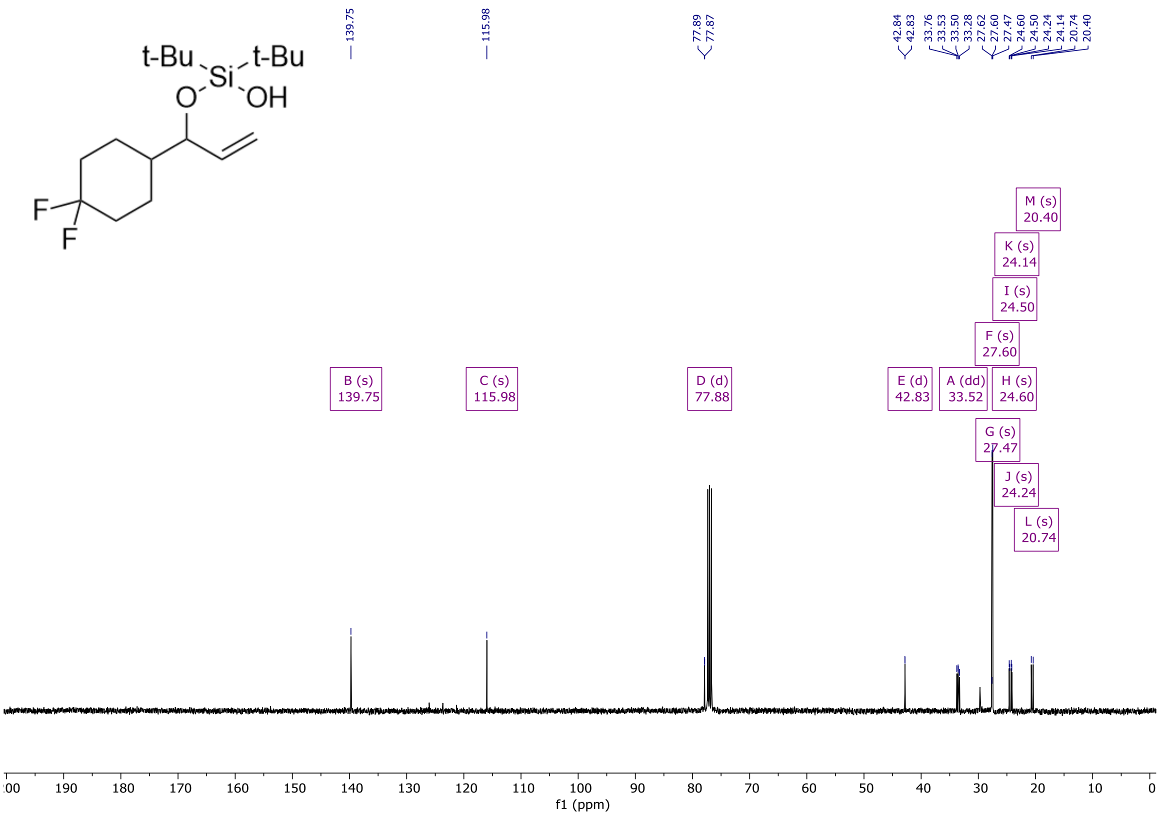

Compound 48 isomer (^{1}H NMR: 400 MHz, ^{13}C NMR: 100 MHz)

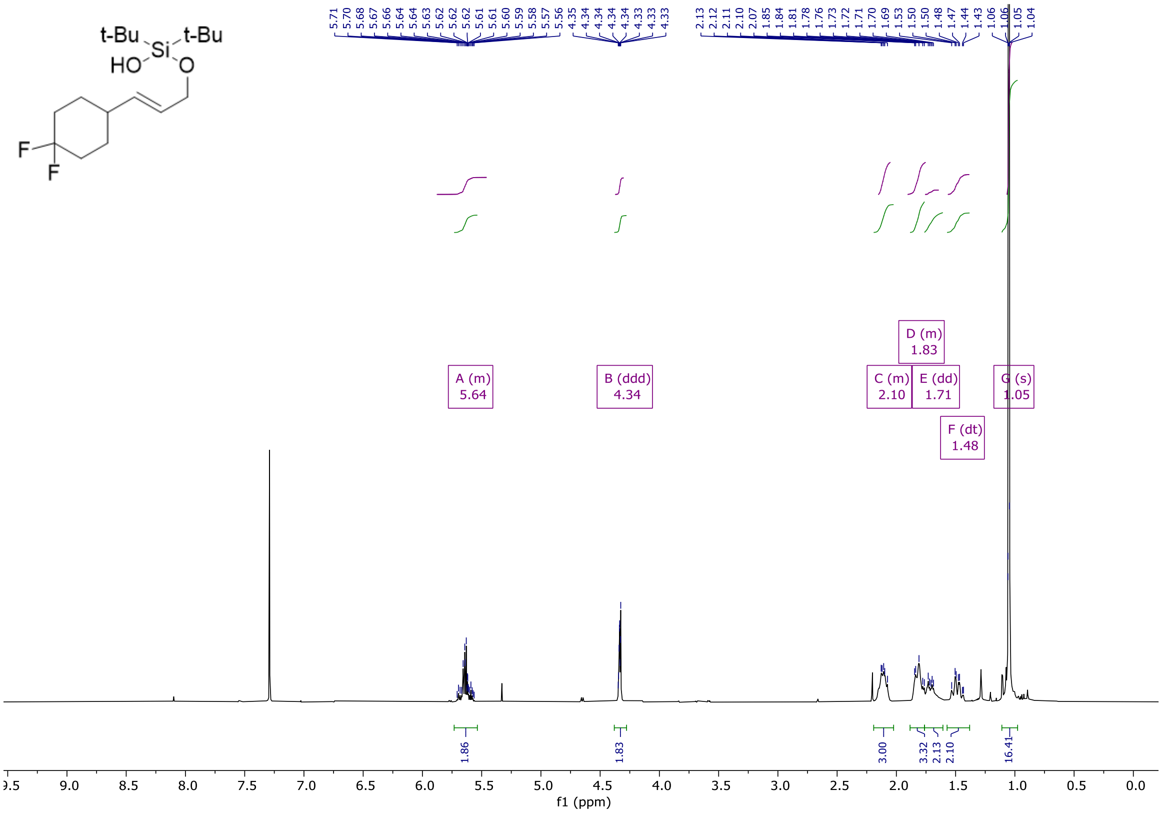

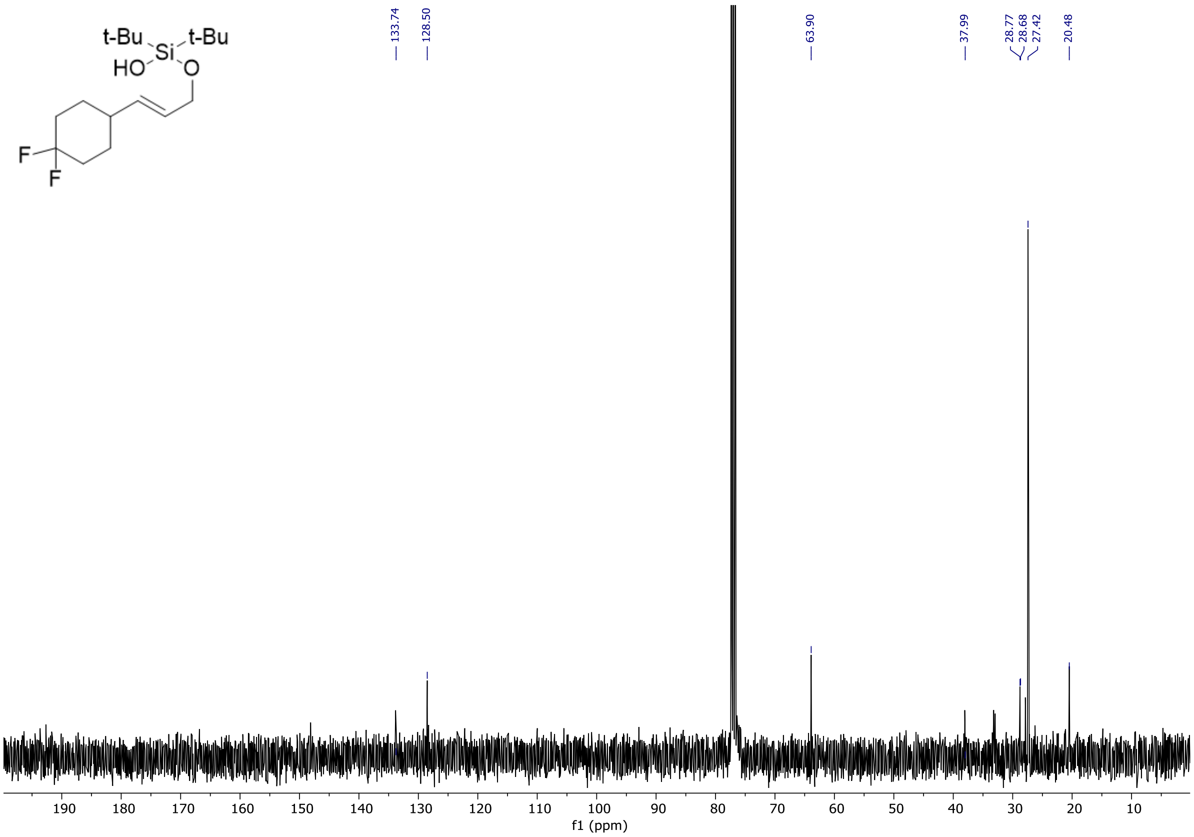

Compound 49 (^{1}H NMR: 400 MHz, ^{13}C NMR: 100 MHz)

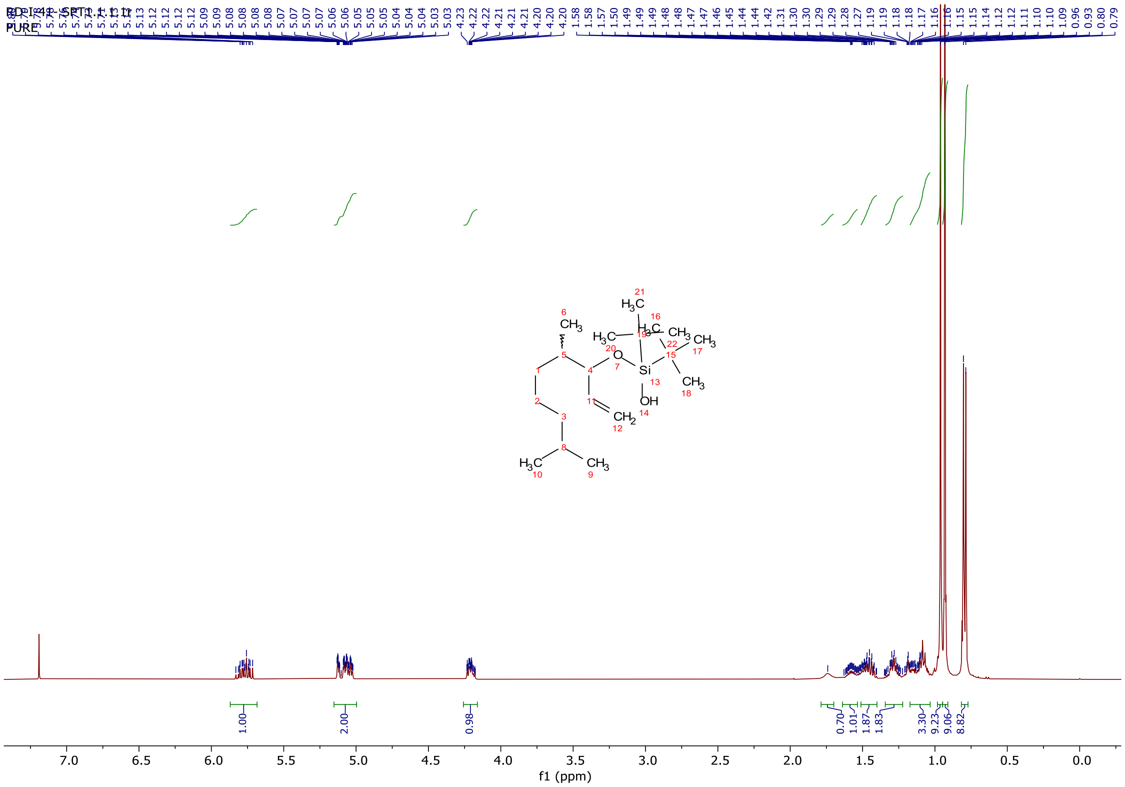

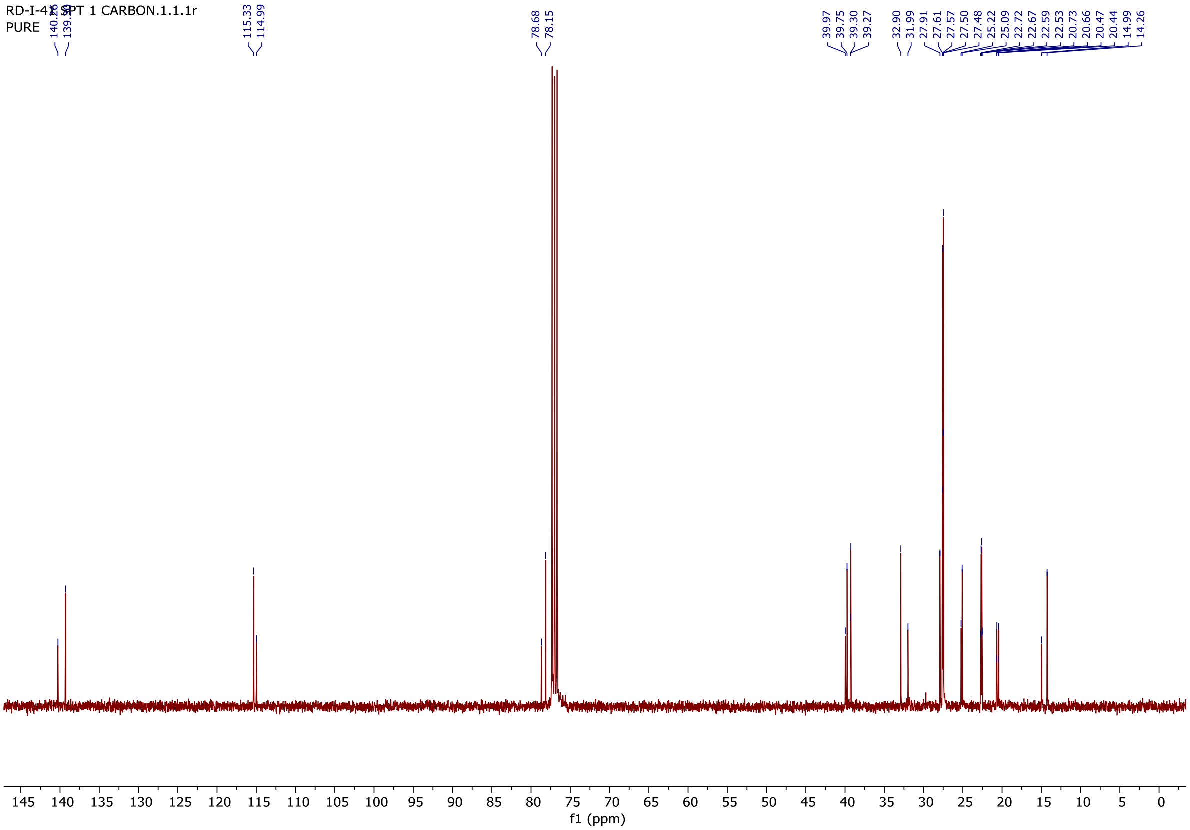

Compound 49 isomer (^{1}H NMR: 400 MHz, ^{13}C NMR: 100 MHz)

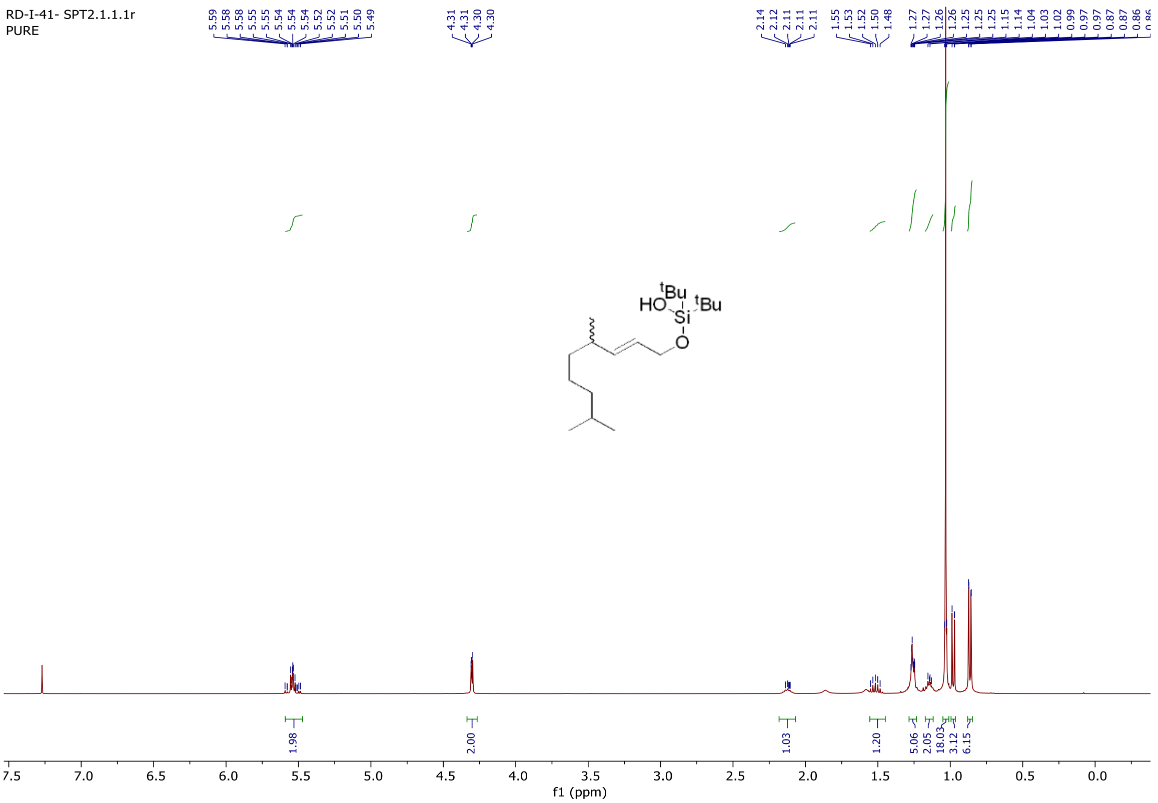

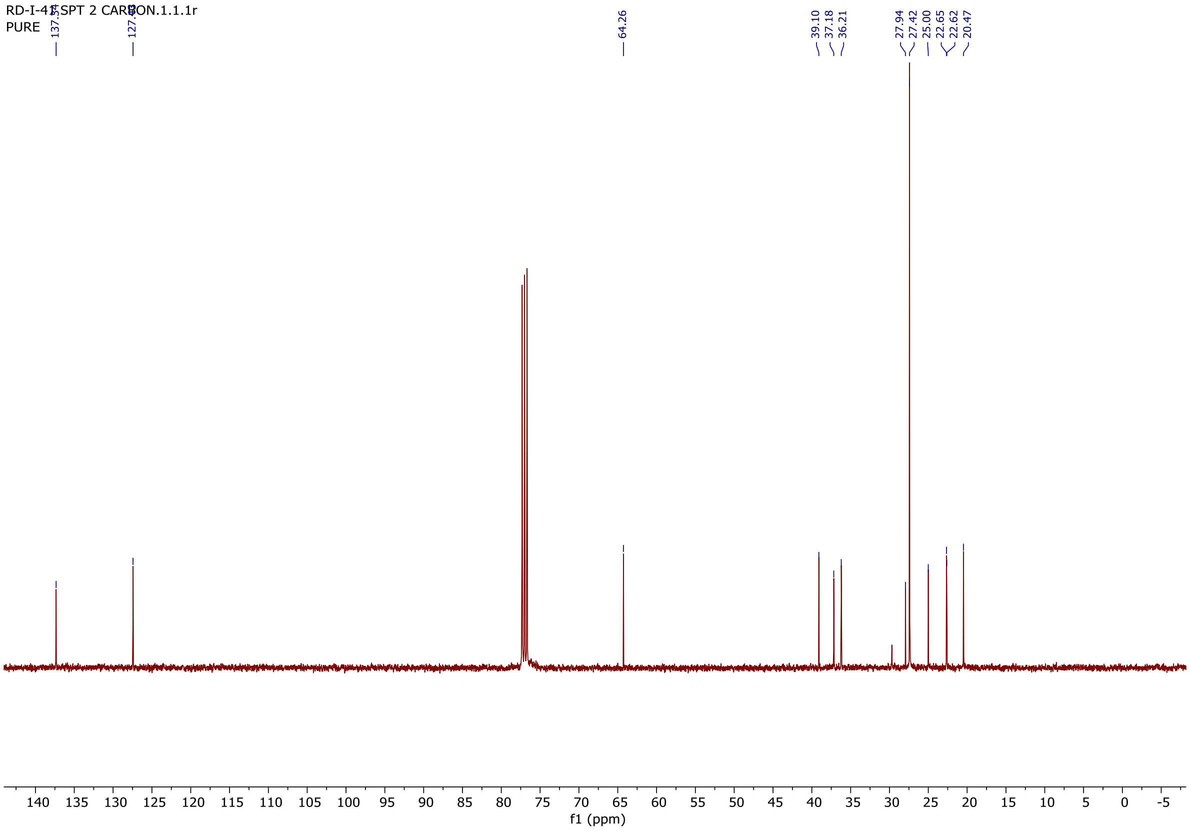

Compound 50 (^{1}H NMR: 400 MHz, ^{13}C NMR: 100 MHz)

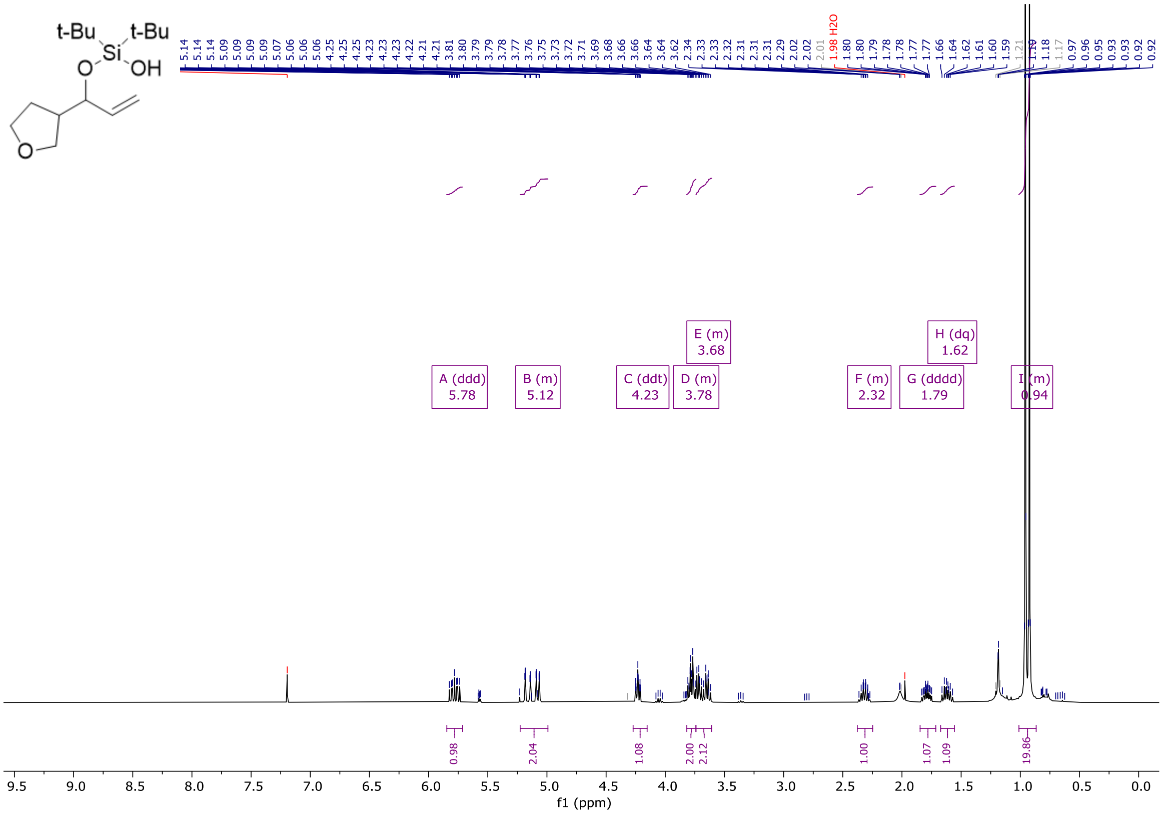

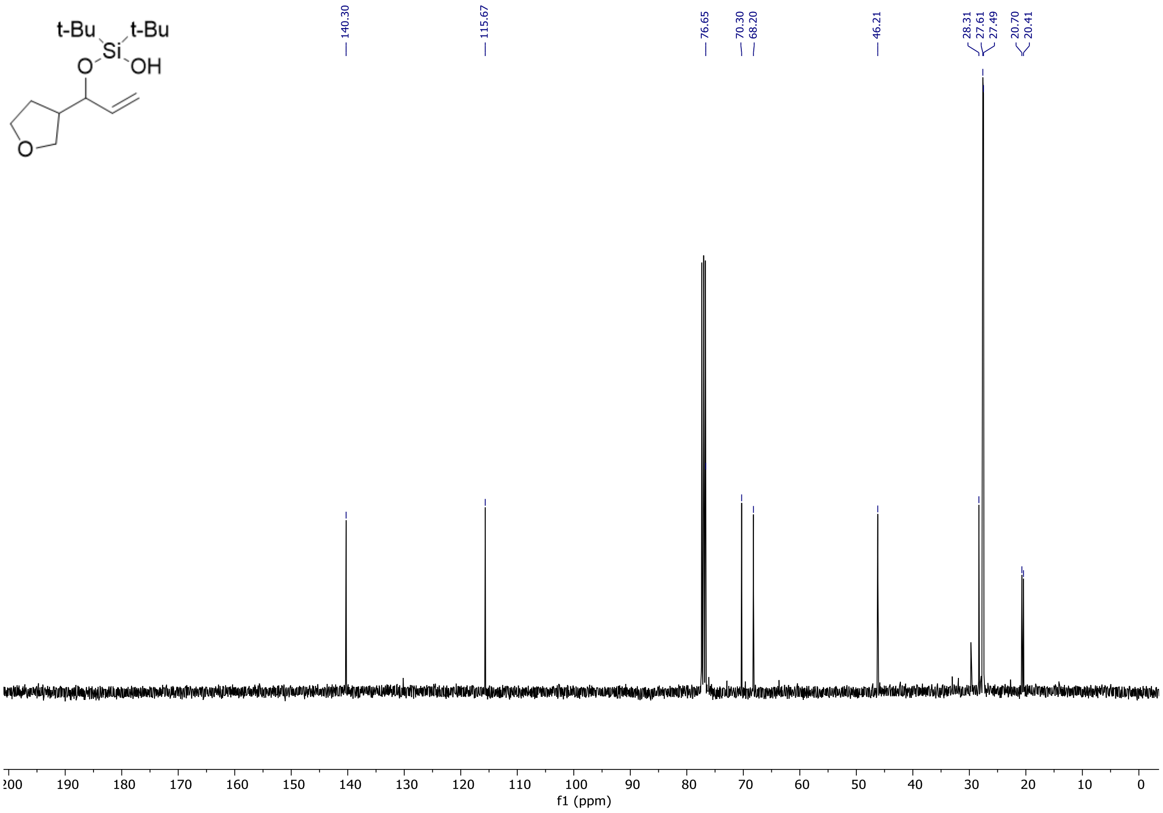

Compound 50 isomer (^{1}H NMR: 400 MHz, ^{13}C NMR: 100 MHz)

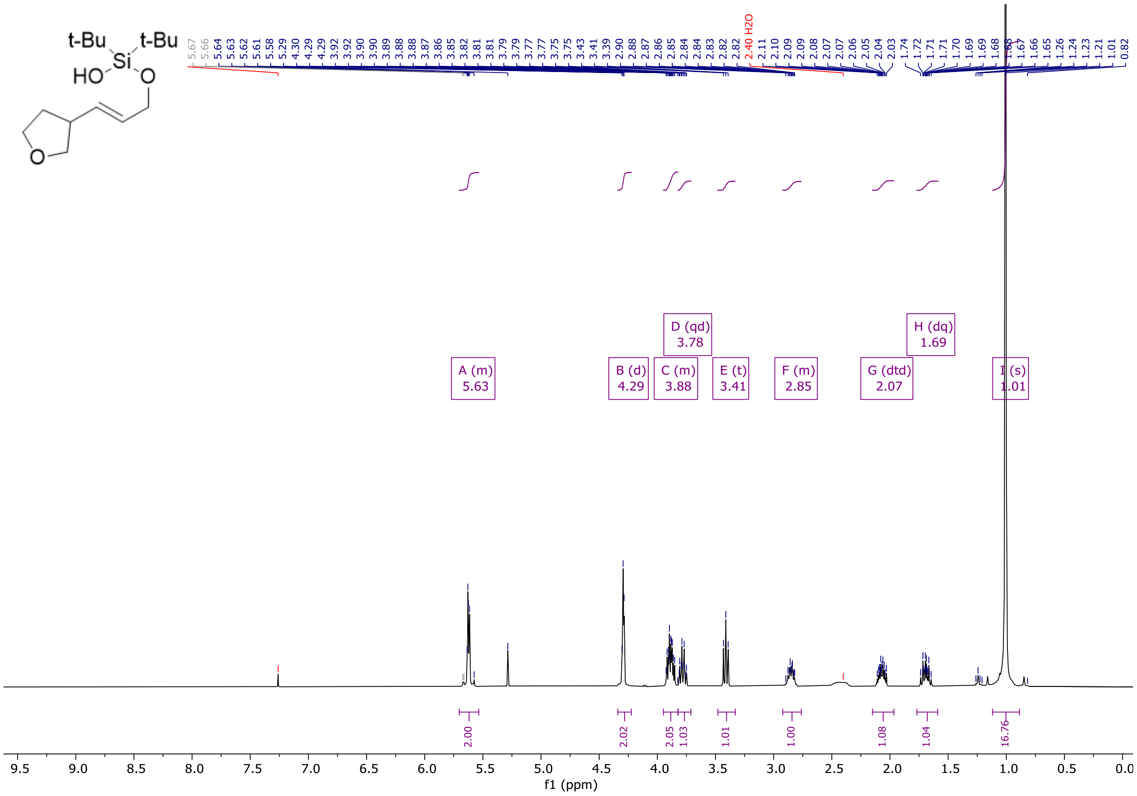

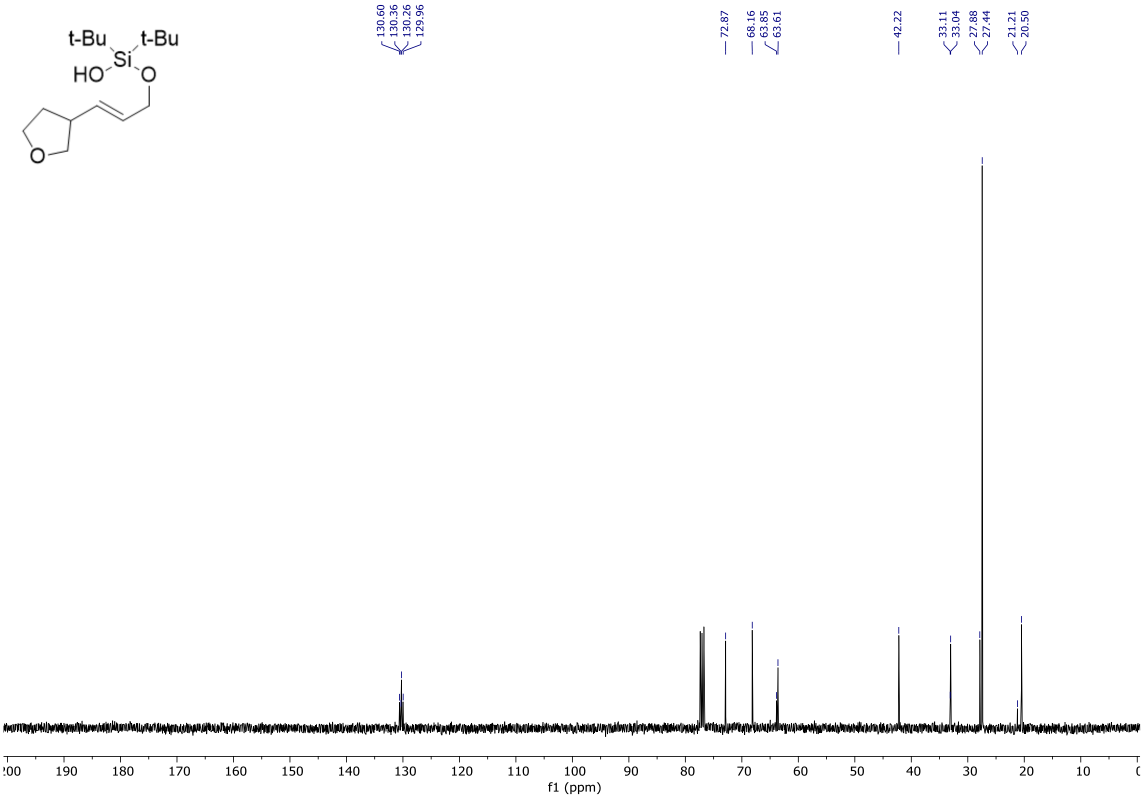

Compound 51 (^1H NMR: 400 MHz, ^{13}C NMR: 100 MHz)

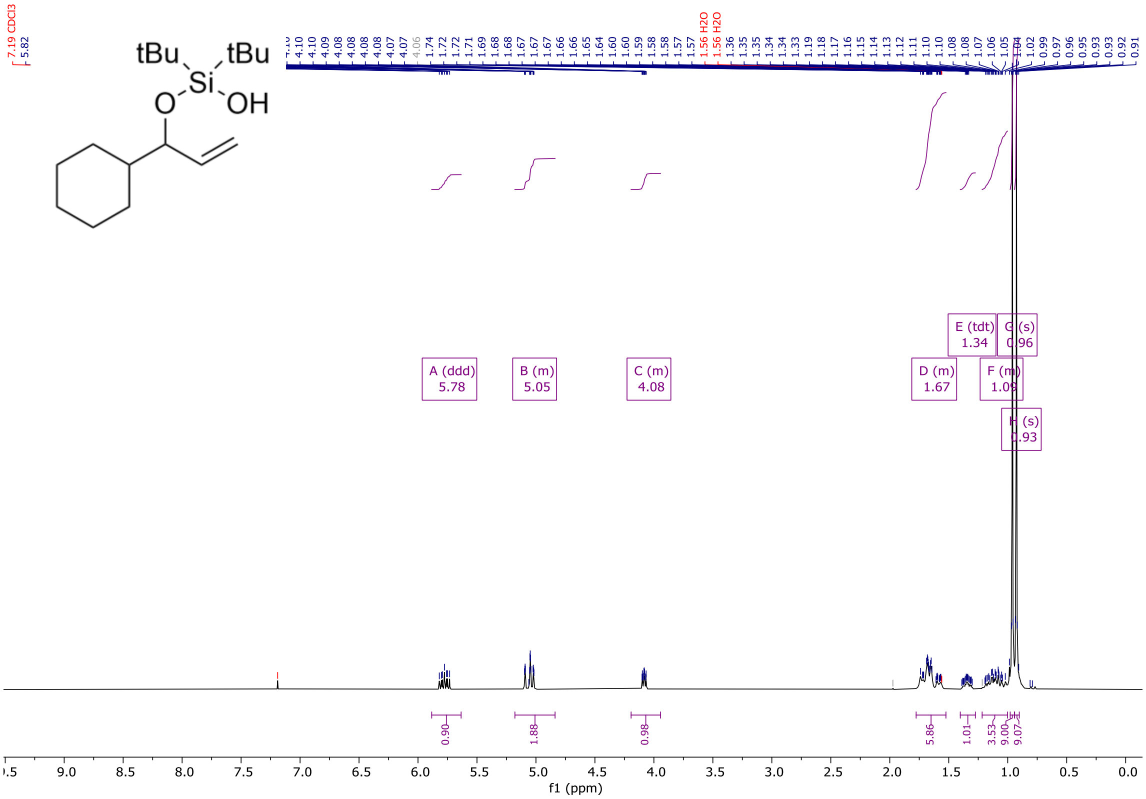

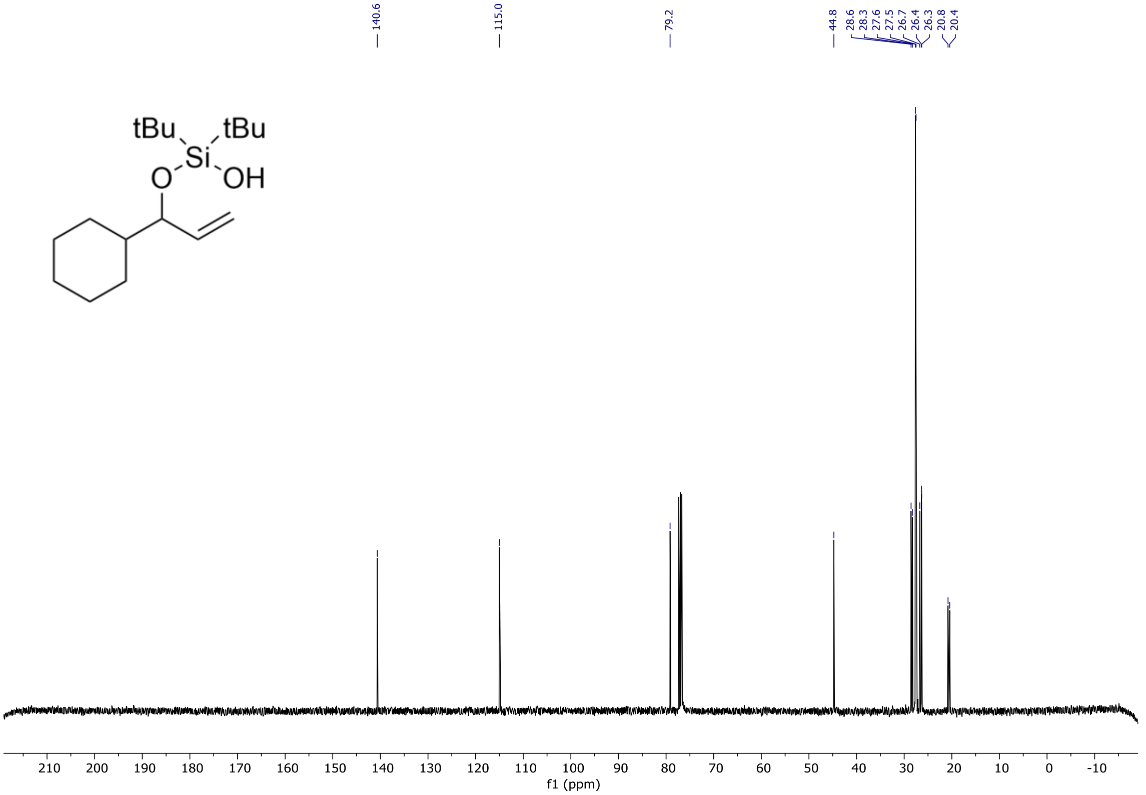

Compound 52 (^{1}H NMR: 400 MHz, ^{13}C NMR: 100 MHz)

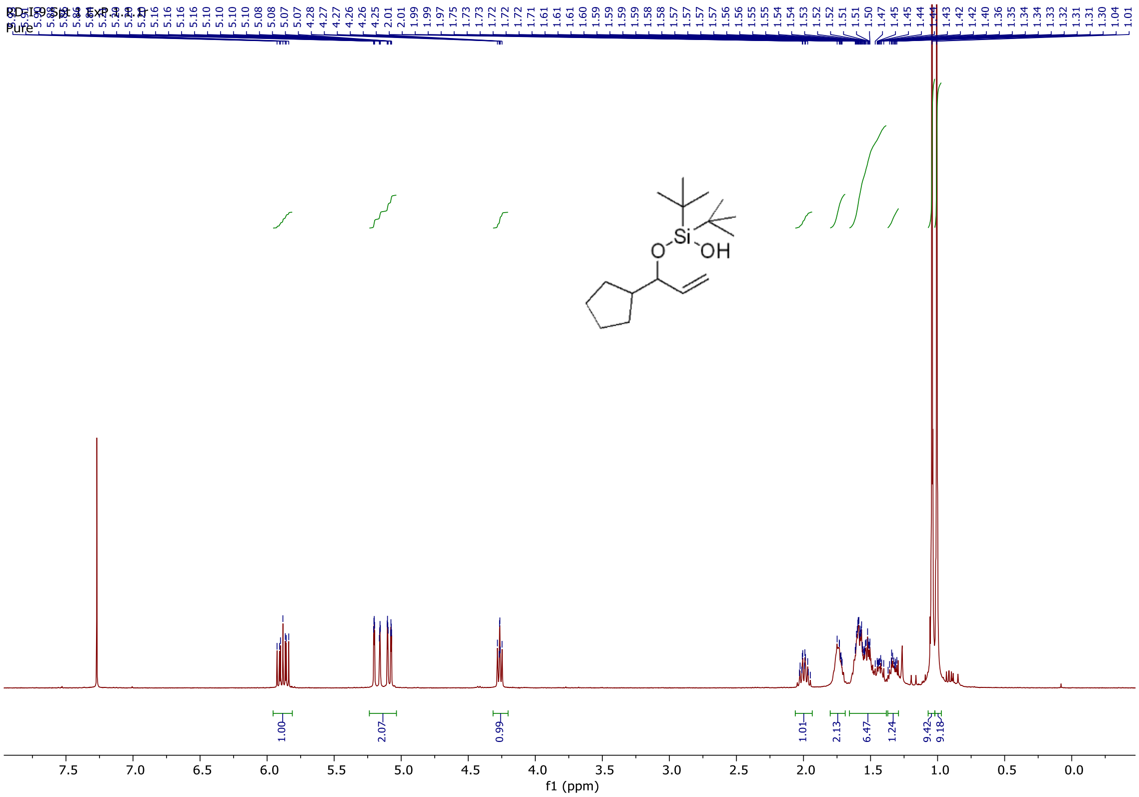

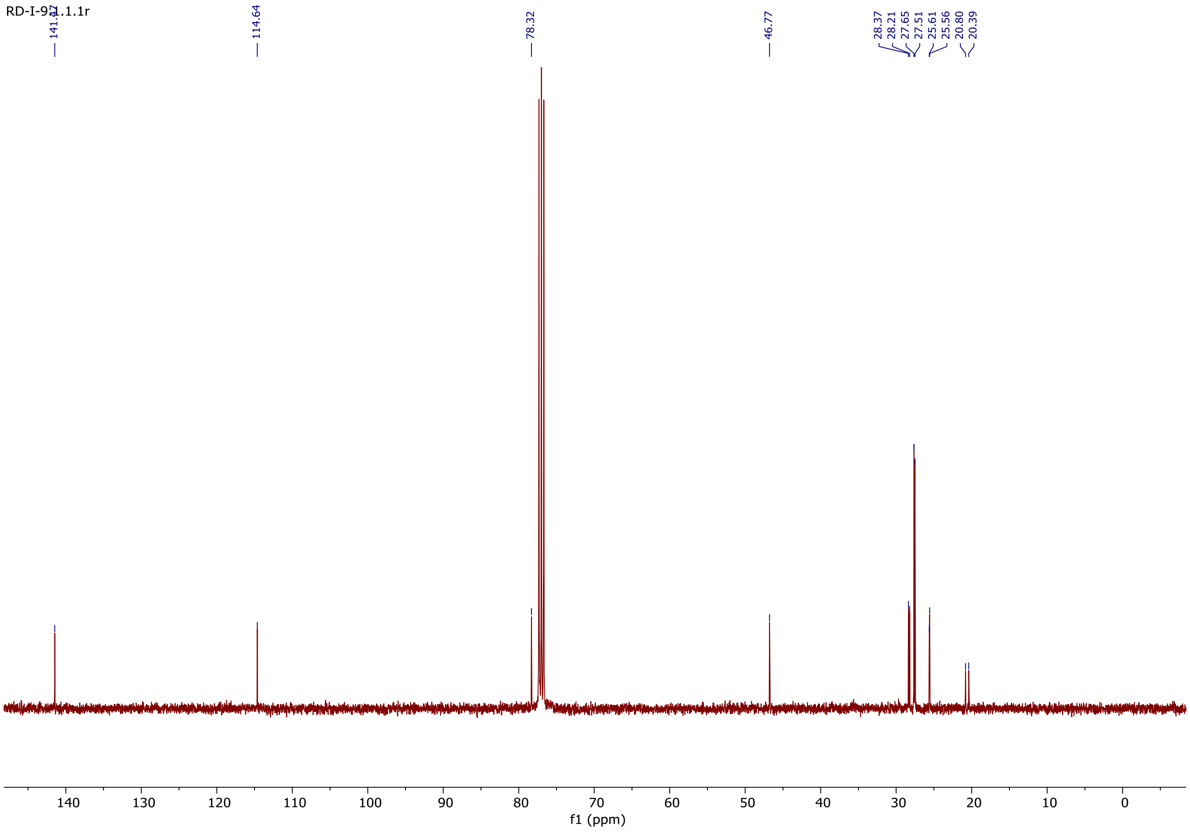

Compound 52 isomer (^{1}H NMR: 400 MHz, ^{13}C NMR: 100 MHz)

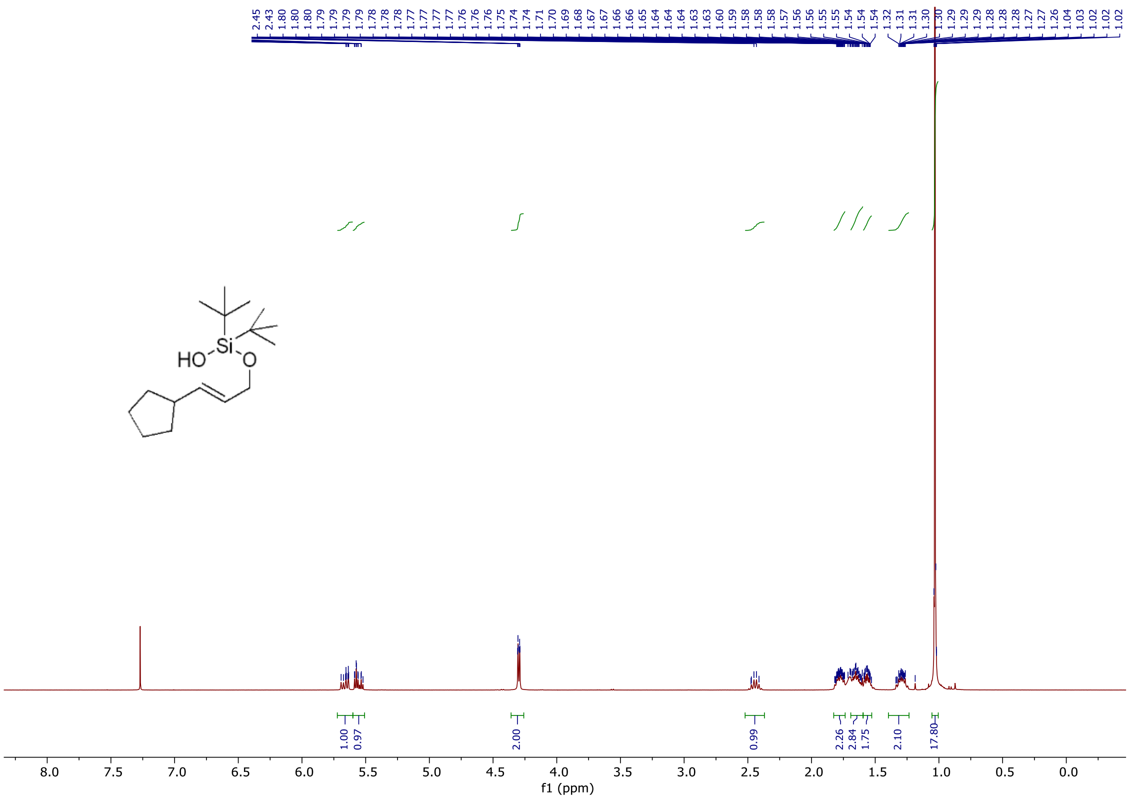

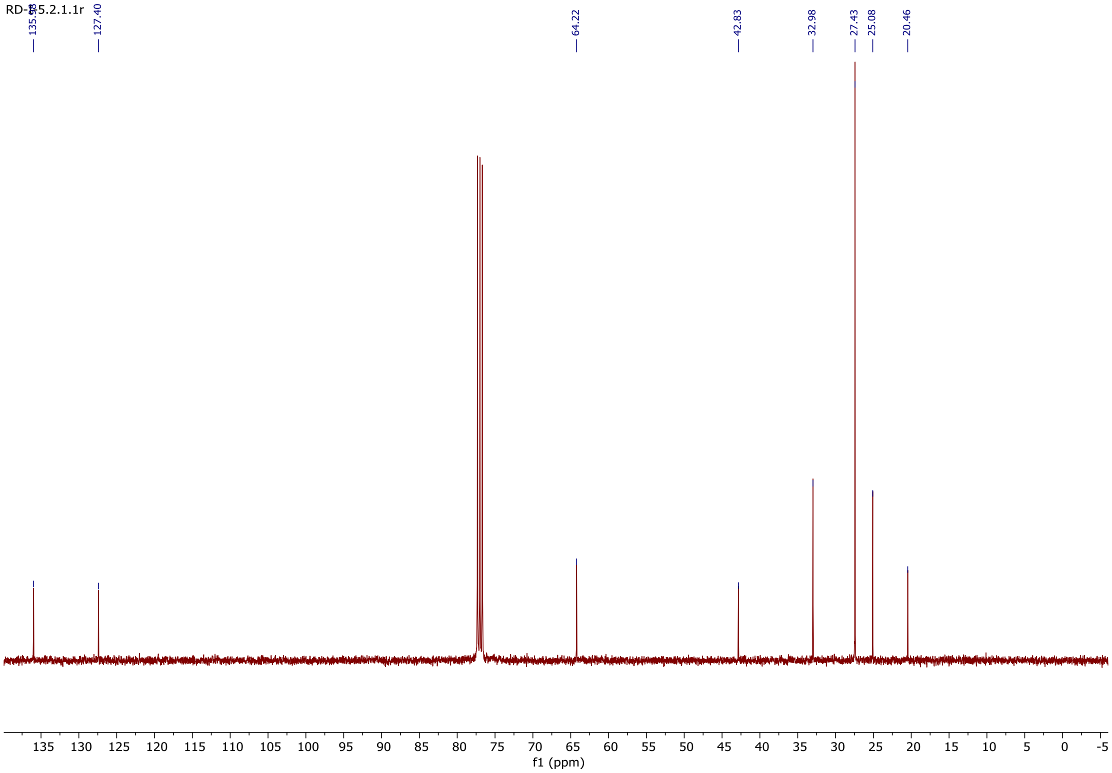

Compound 53 (^{1}H NMR: 400 MHz, ^{13}C NMR: 100 MHz)

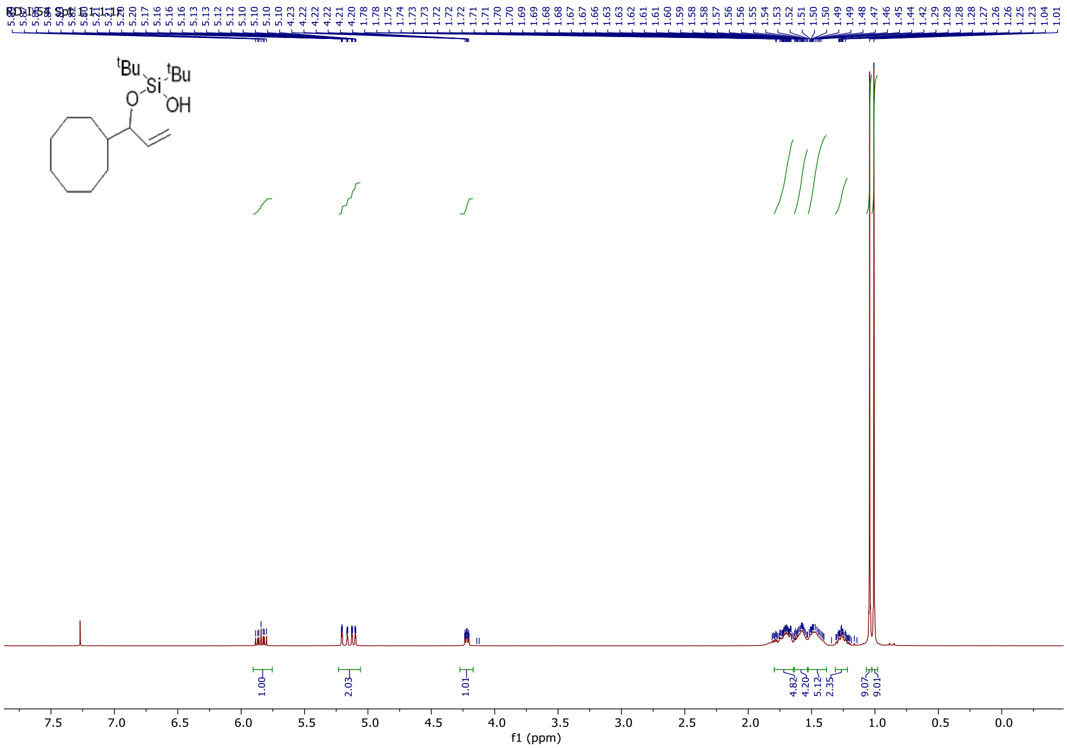

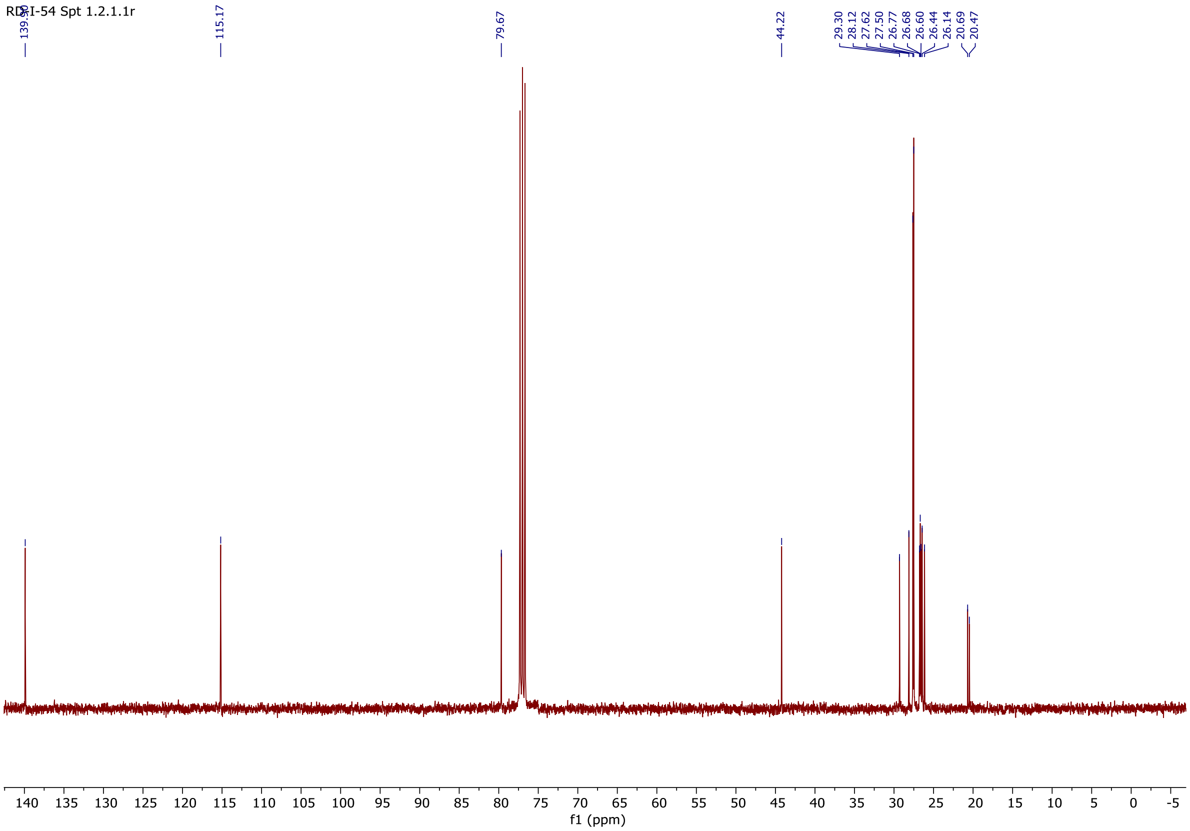

Compound 53 isomer (^{1}H NMR: 400 MHz, ^{13}C NMR: 100 MHz)

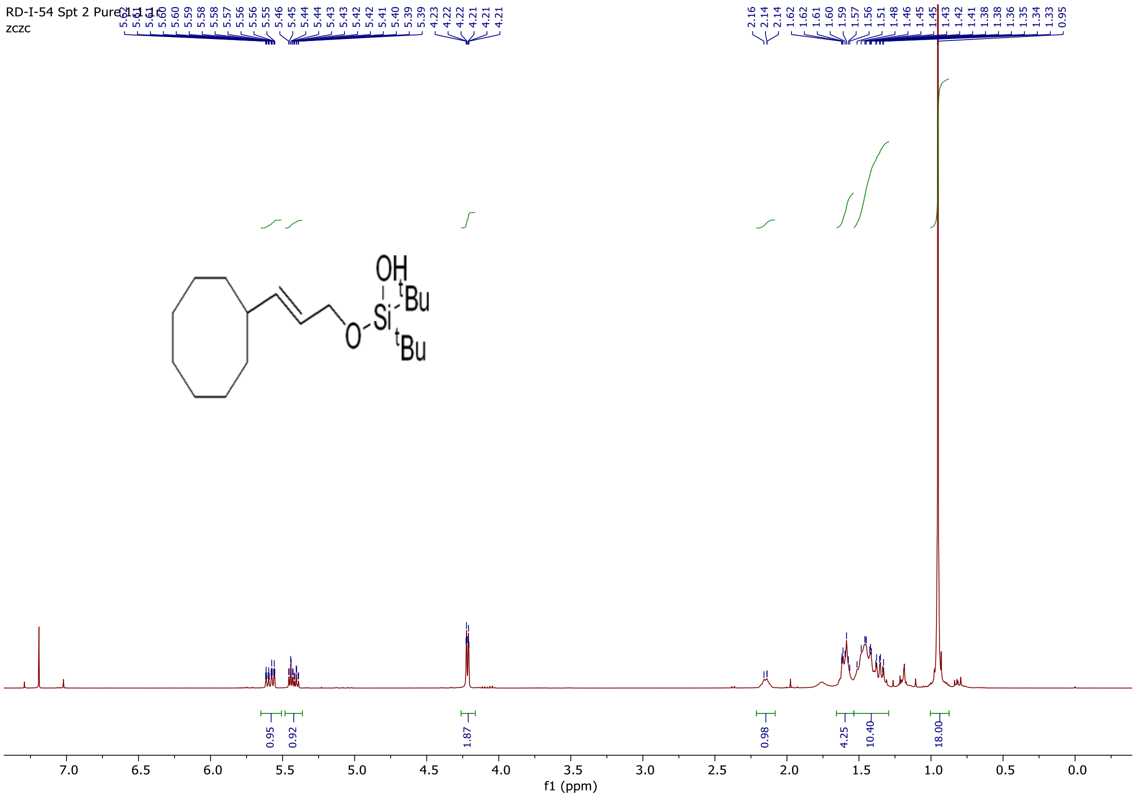

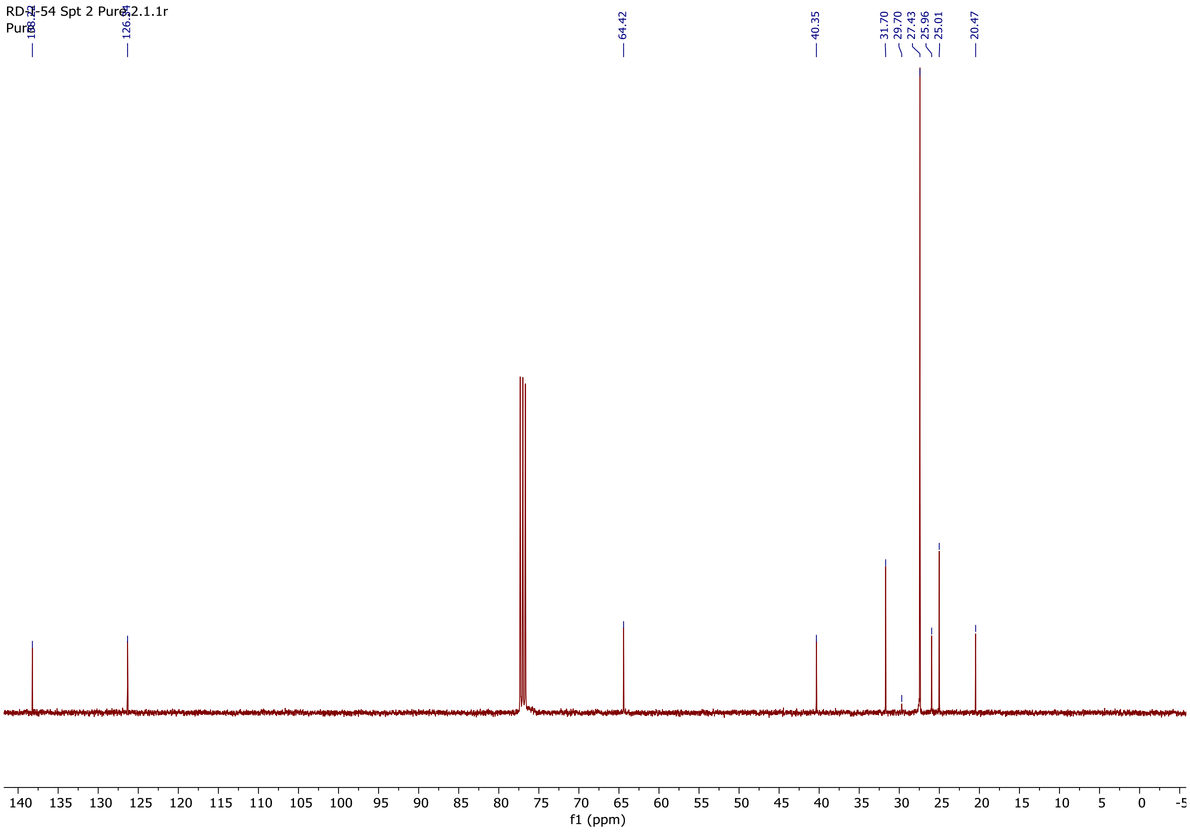

Compound 54 (^{1}H NMR: 400 MHz, ^{13}C NMR: 100 MHz)

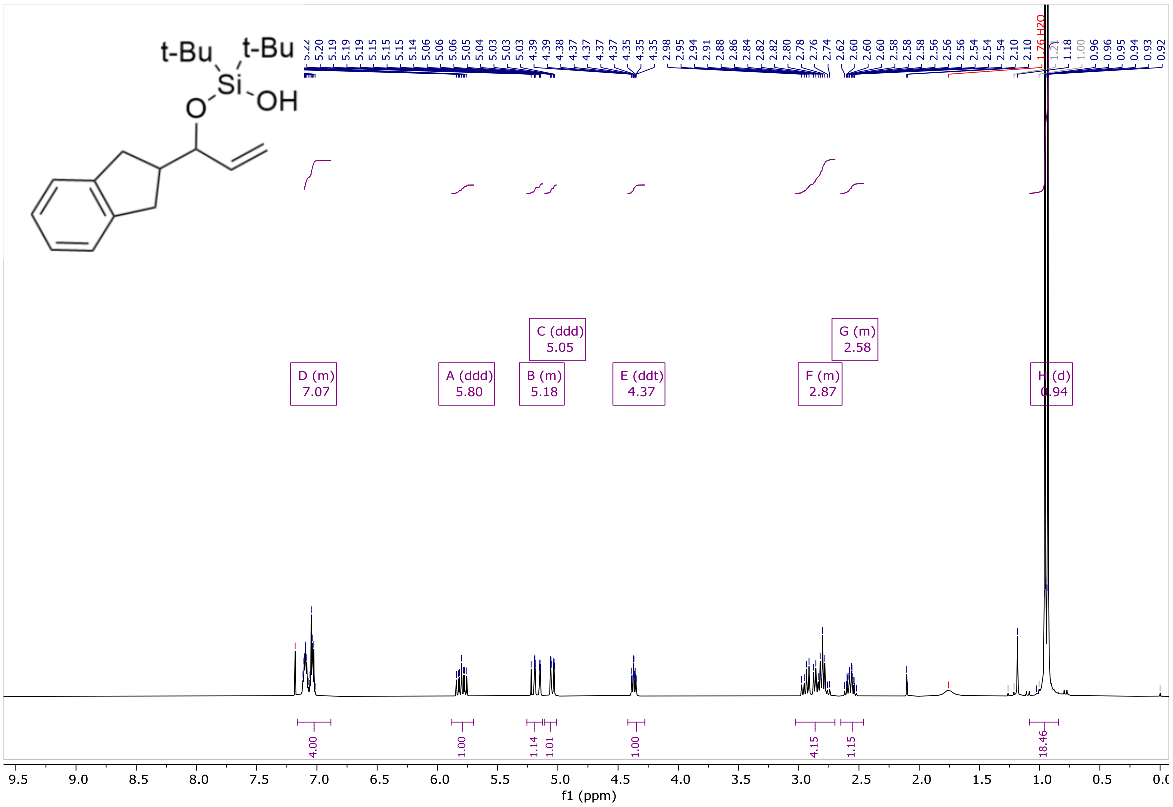

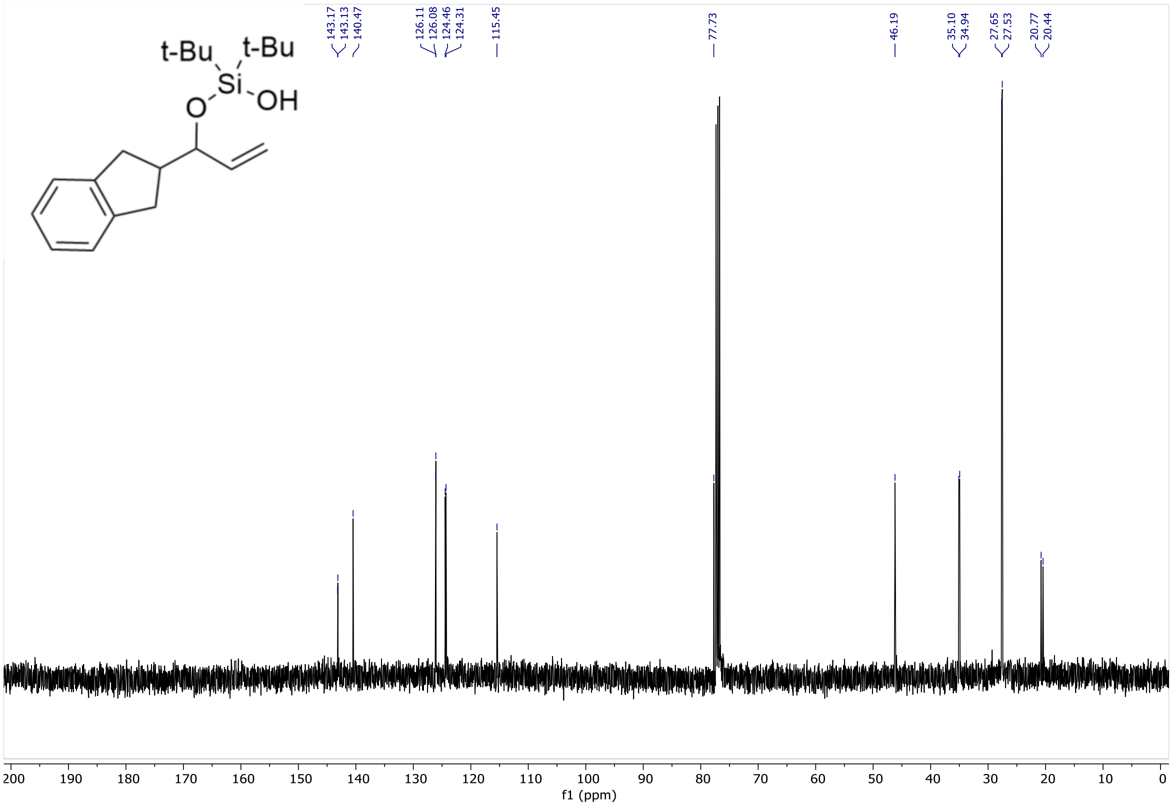

Compound 55 (^{1}H NMR: 400 MHz, ^{13}C NMR: 100 MHz)

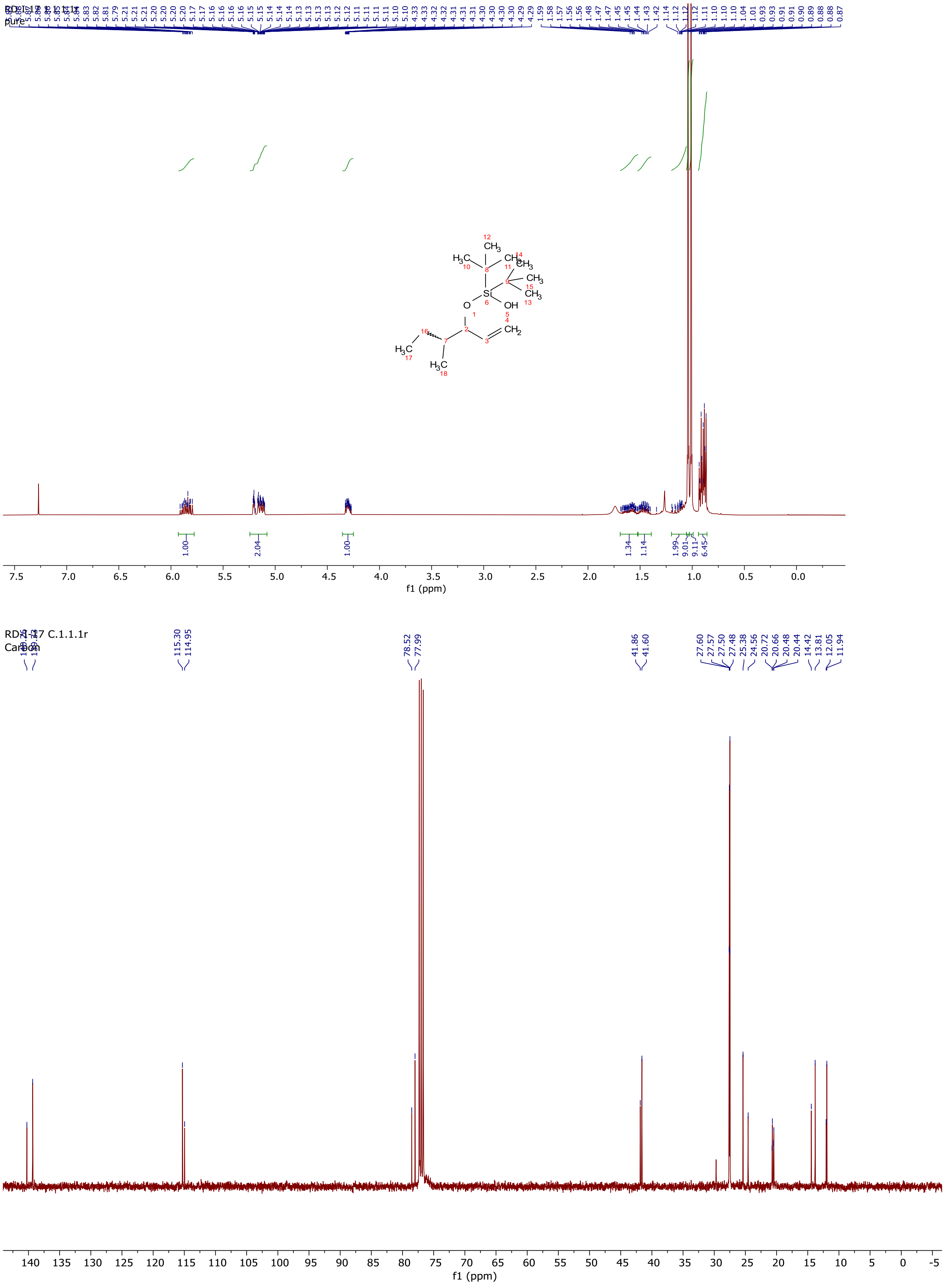

Compound 55 isomer (^{1}H NMR: 400 MHz, ^{13}C NMR: 100 MHz)

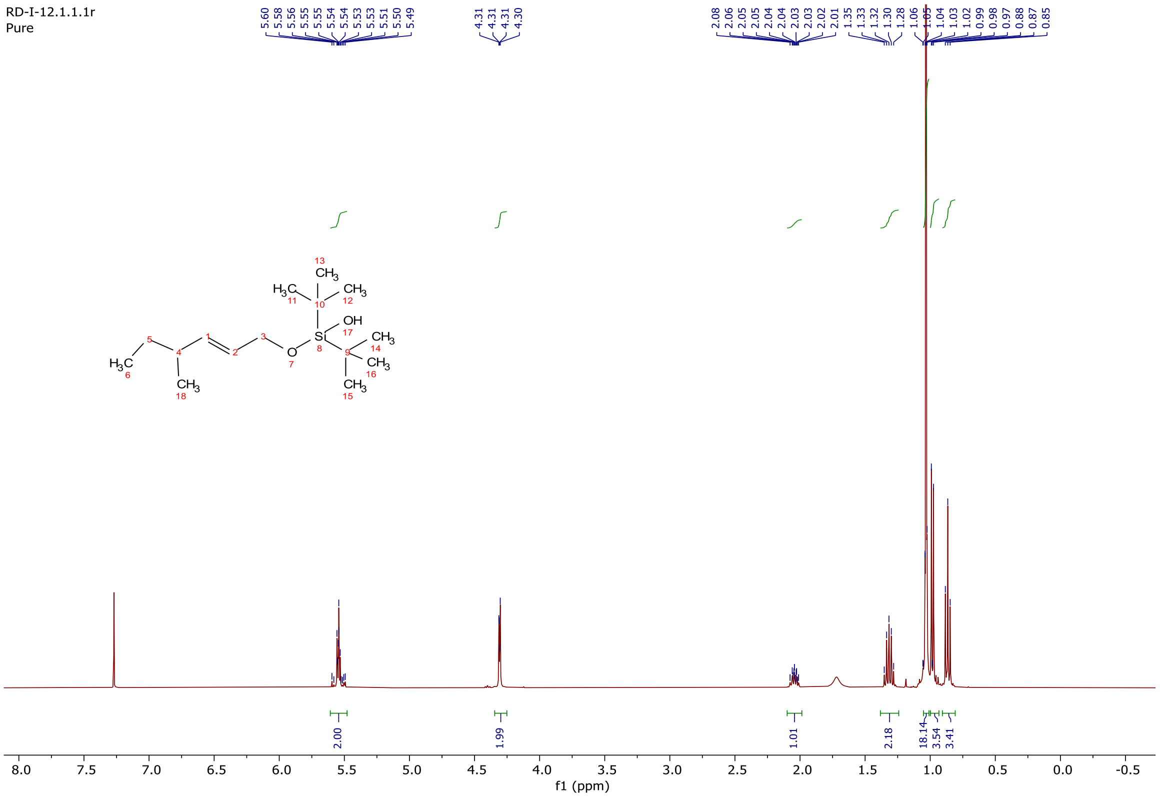

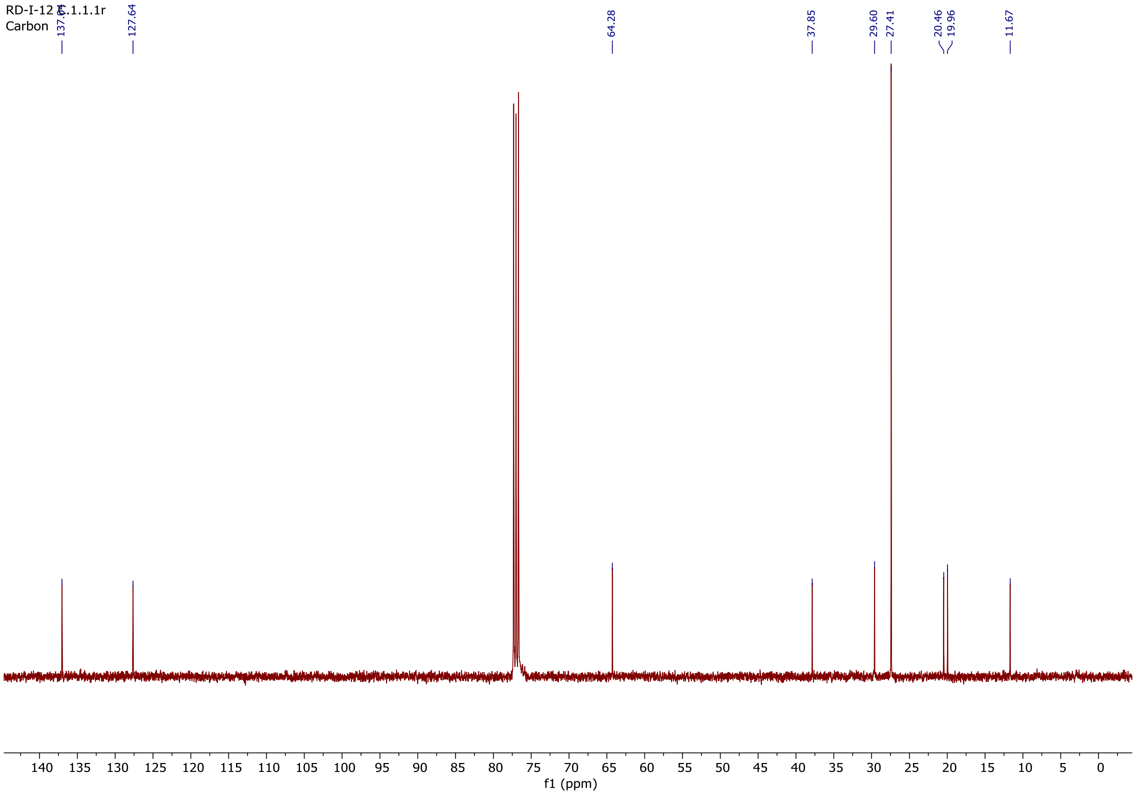

Compound 56 (^{1}H NMR: 400 MHz, ^{13}C NMR: 100 MHz)

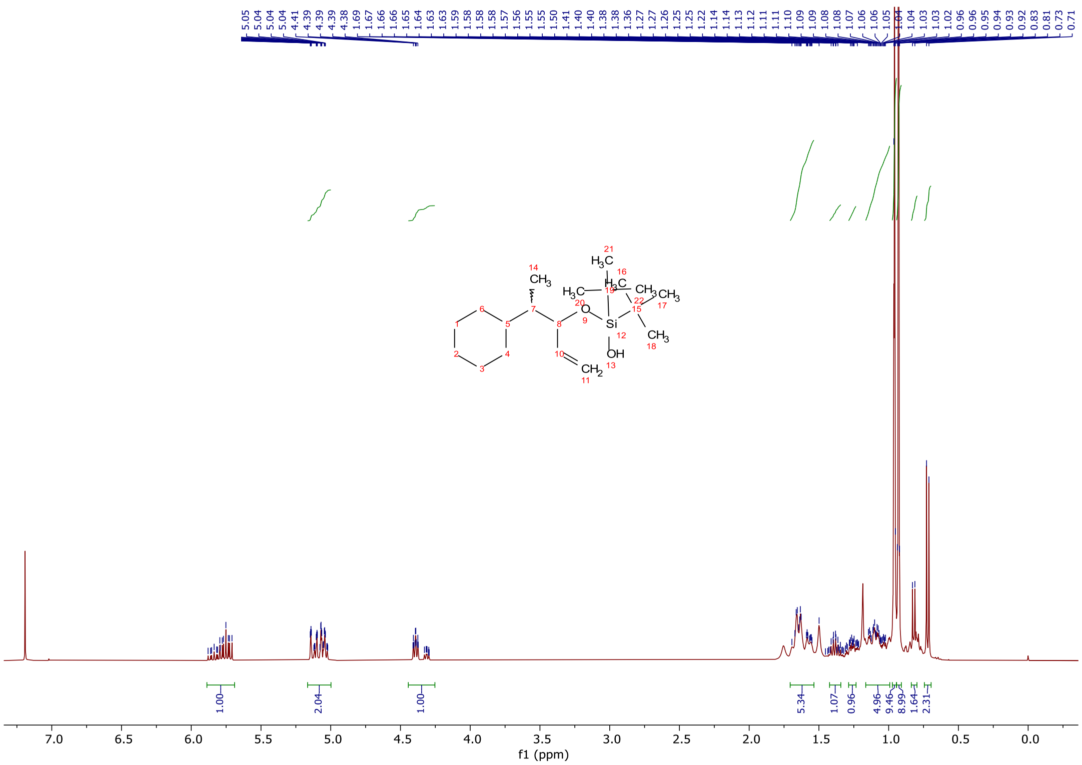

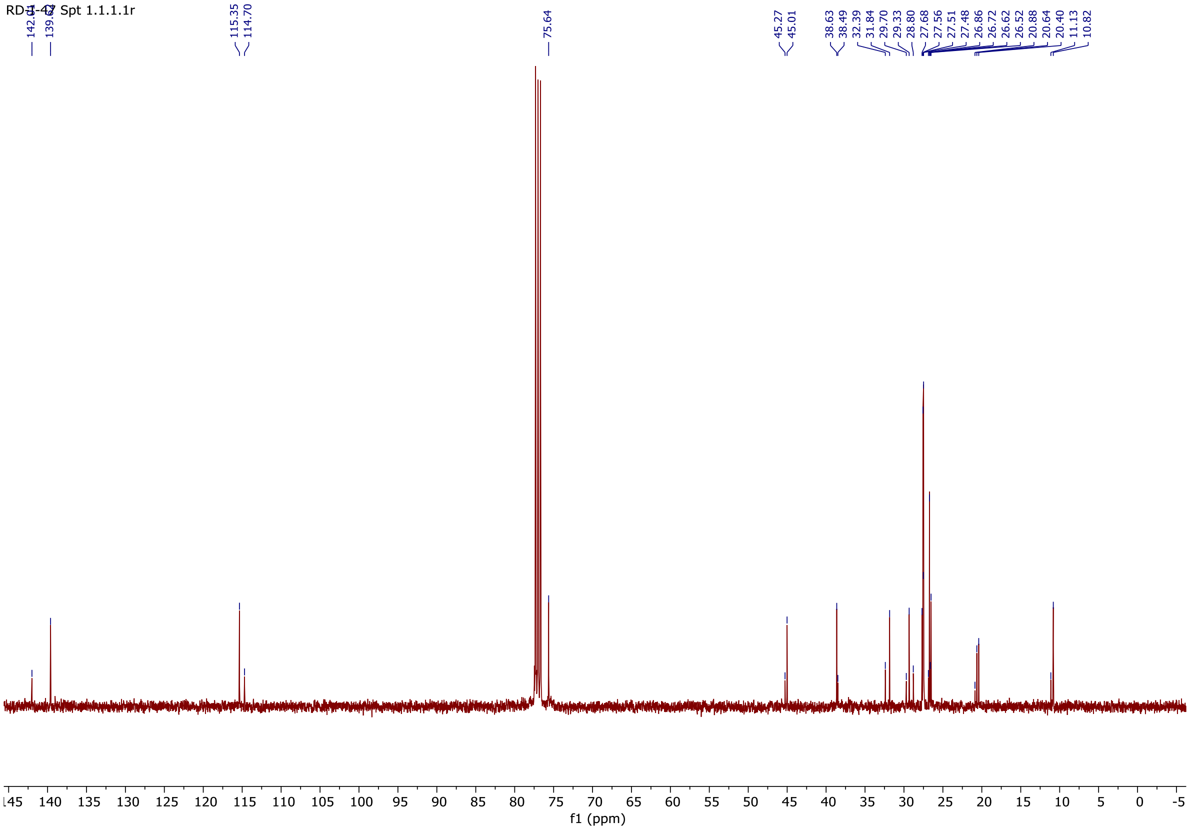

Compound 56 isomer (^{1}H NMR: 400 MHz, ^{13}C NMR: 100 MHz)

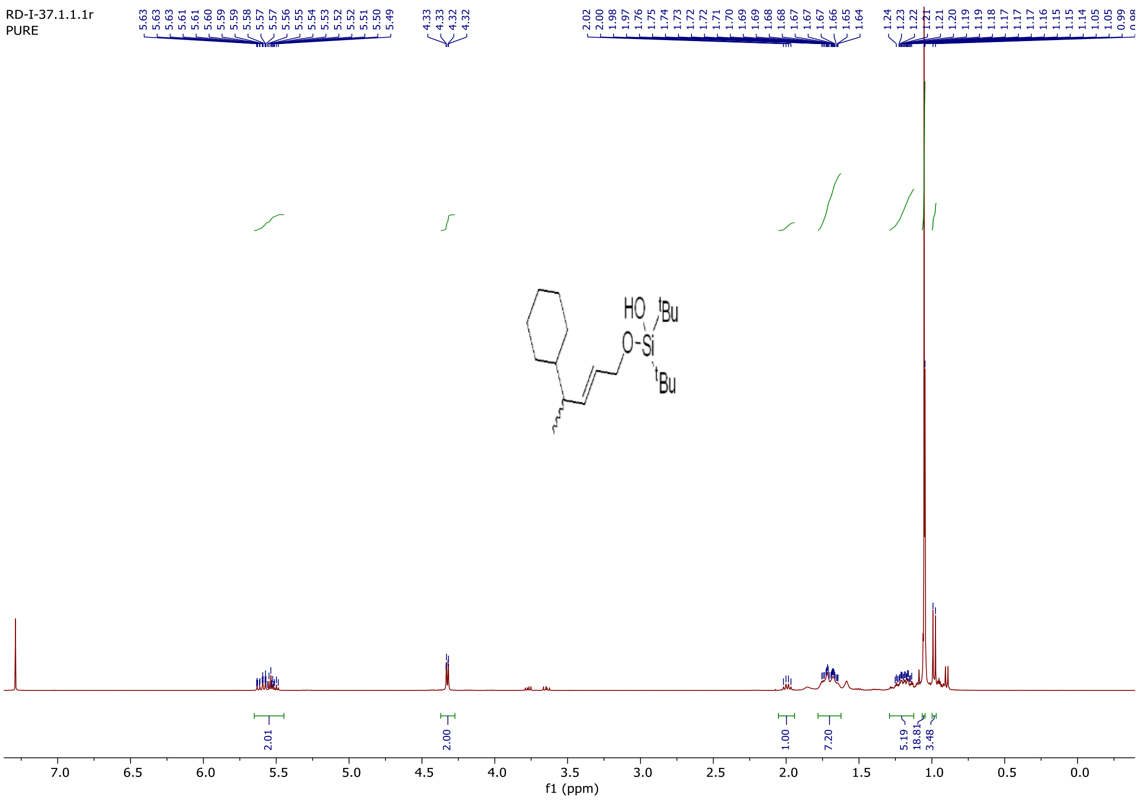

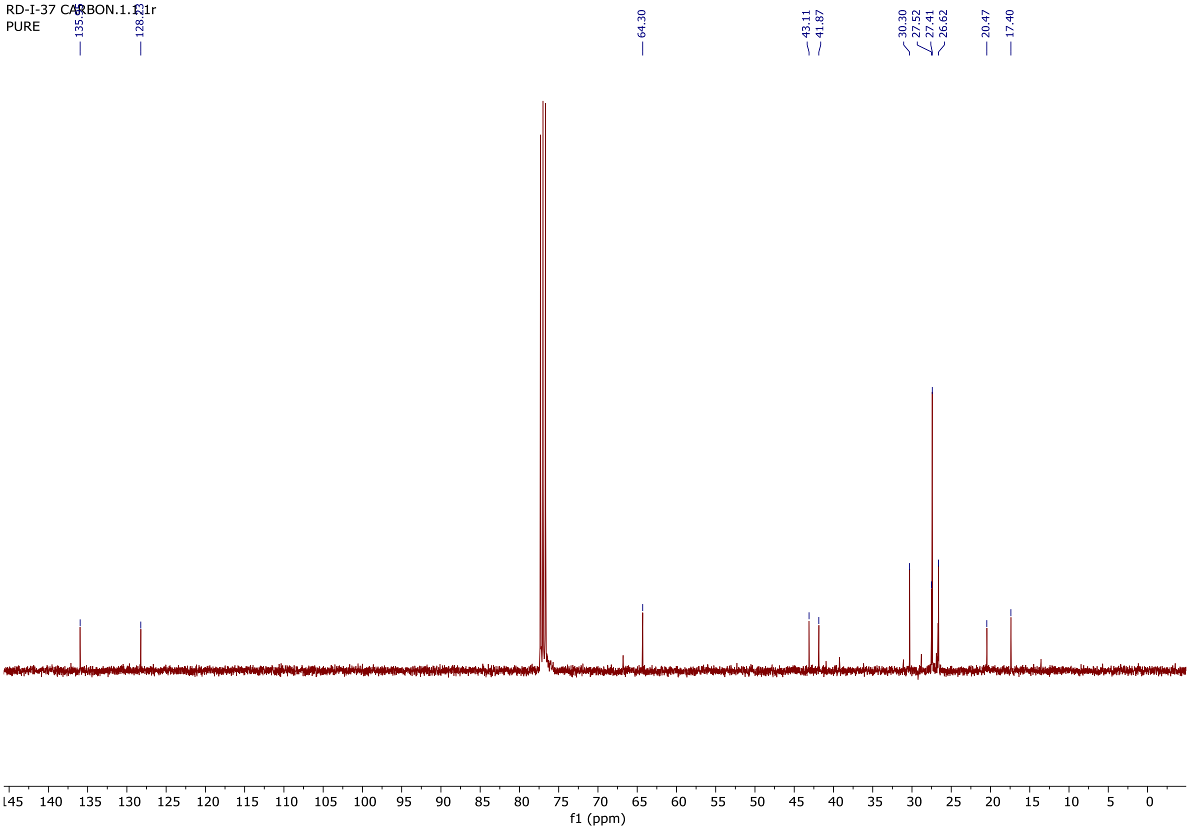

Compound 57 (^{1}H NMR: 400 MHz, ^{13}C NMR: 100 MHz)

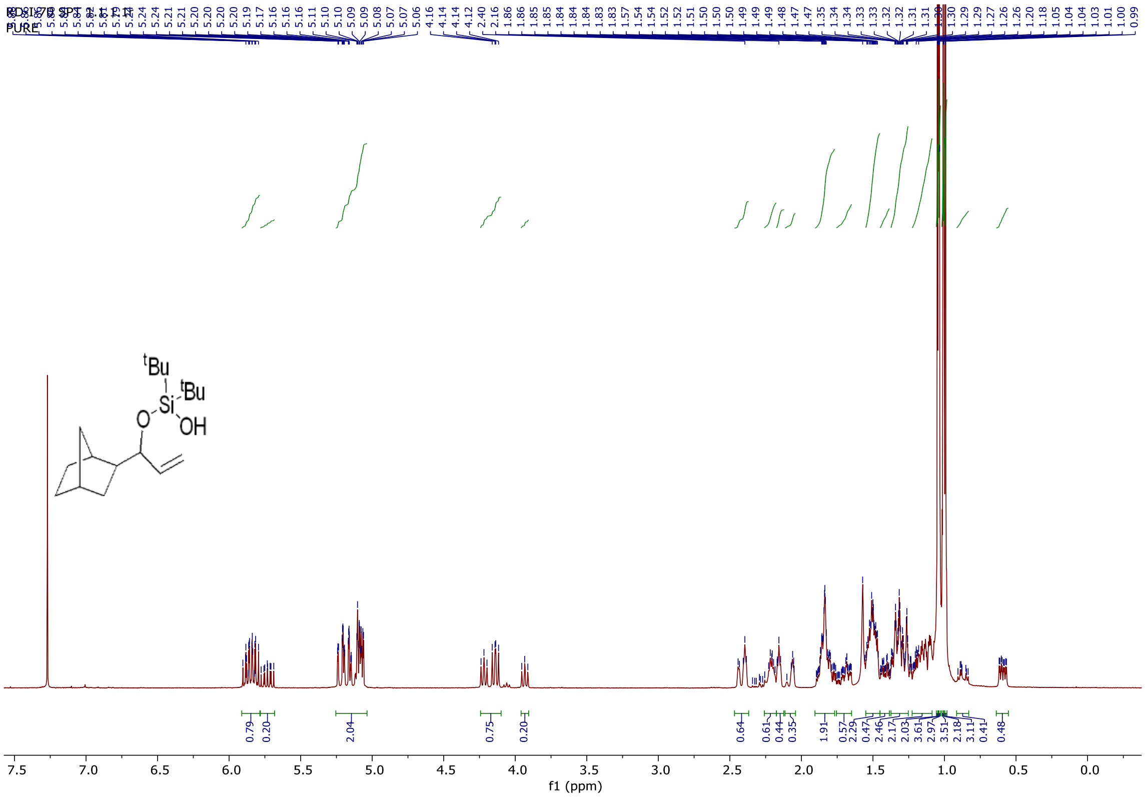

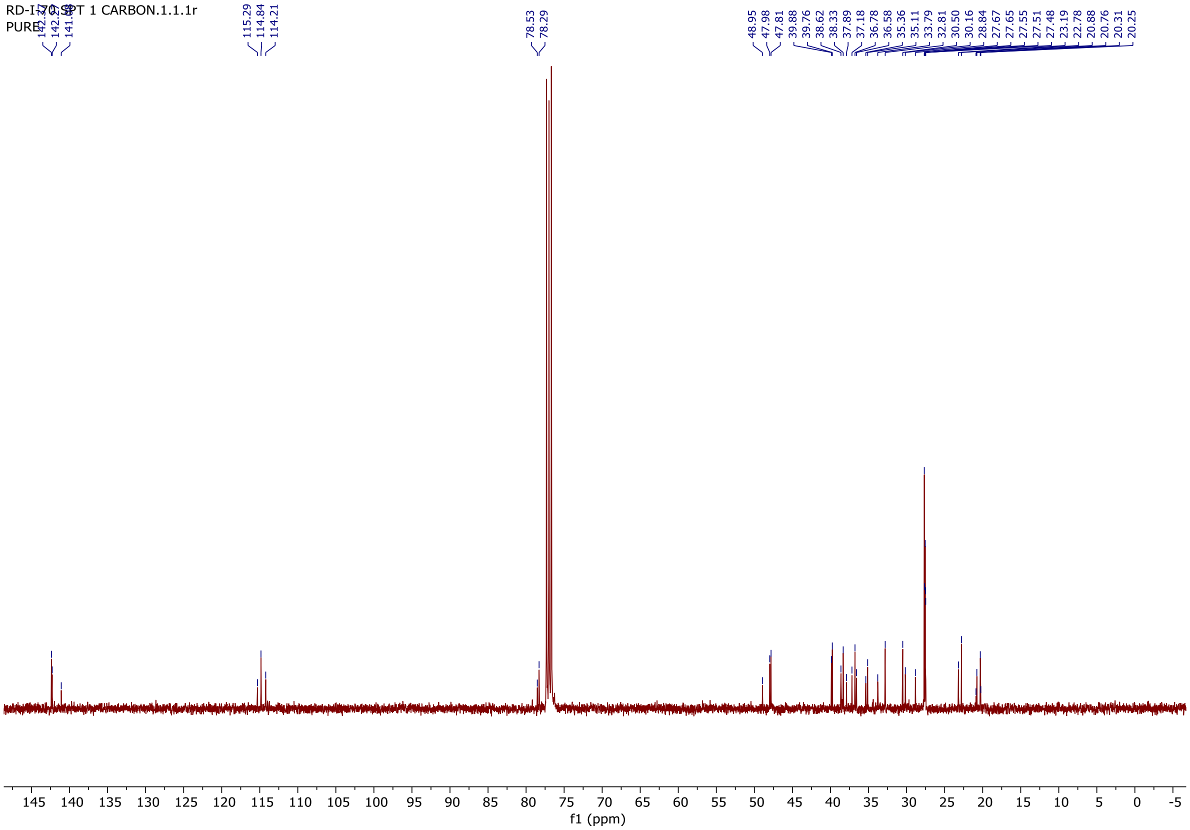

Compound 57 isomer (^{1}H NMR: 400 MHz, ^{13}C NMR: 100 MHz)

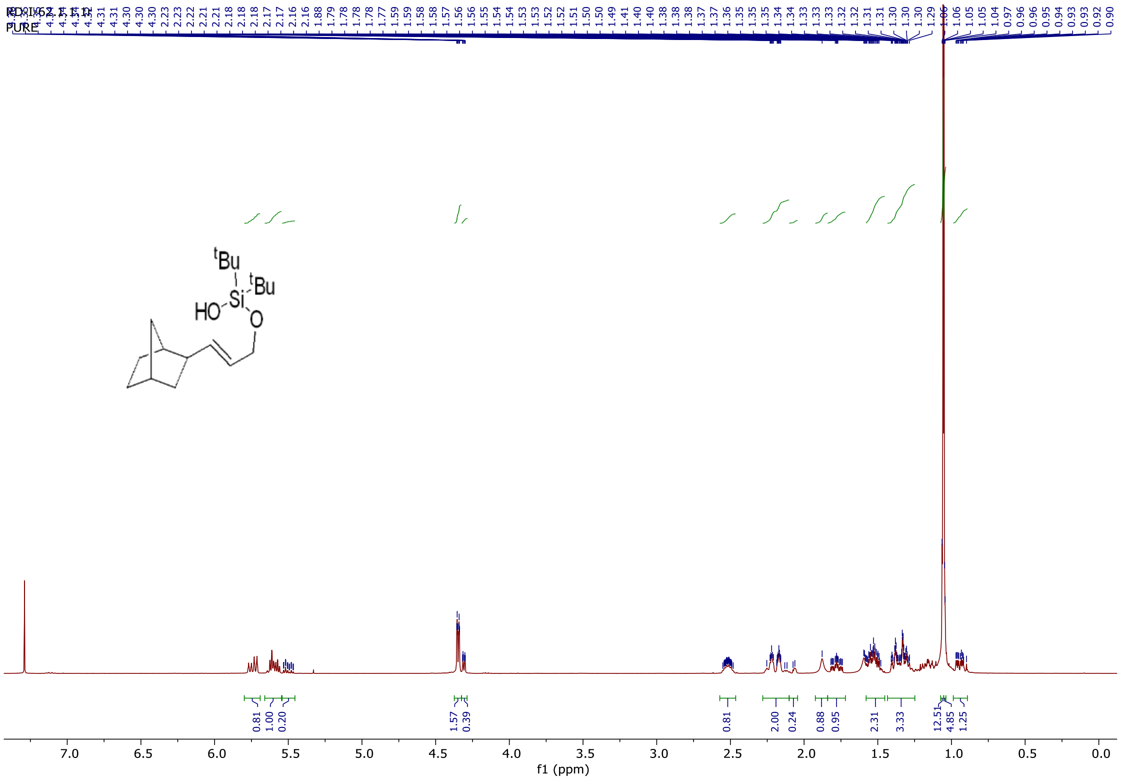

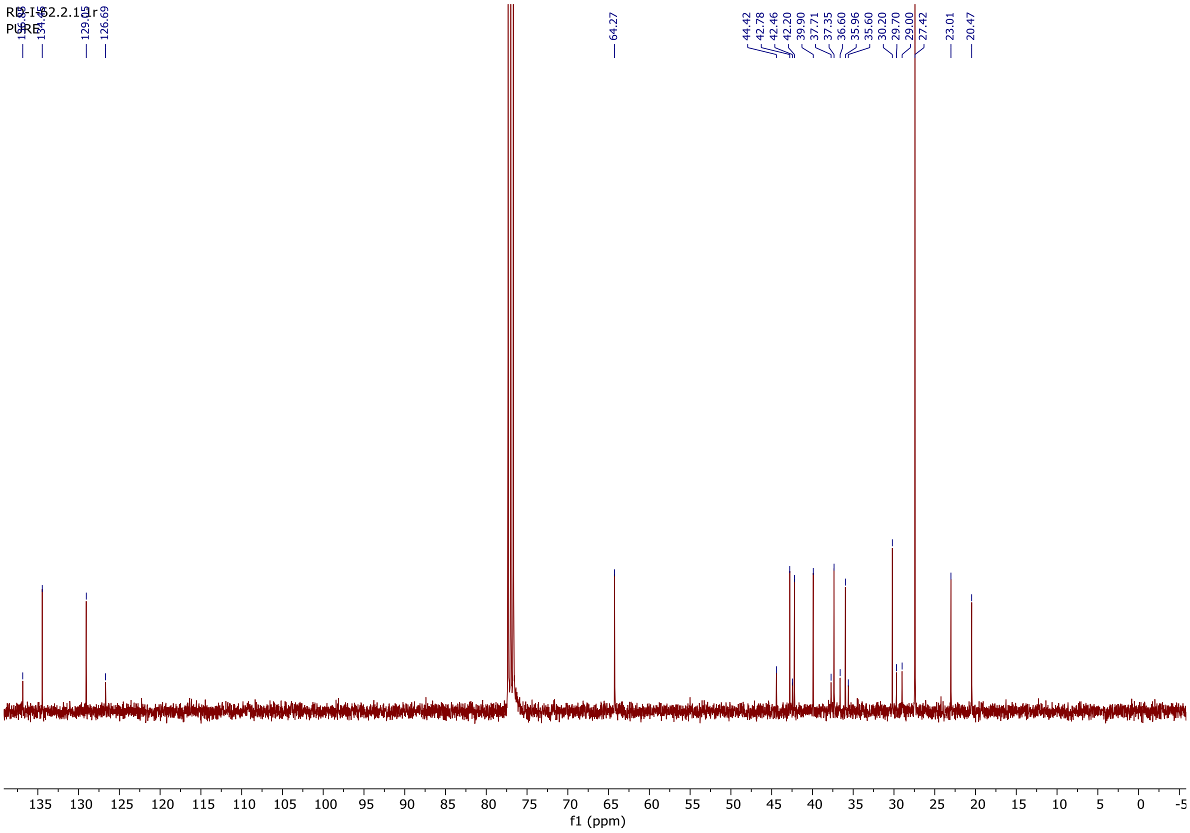

Compound 58 (^{1}H NMR: 400 MHz, ^{13}C NMR: 100 MHz)

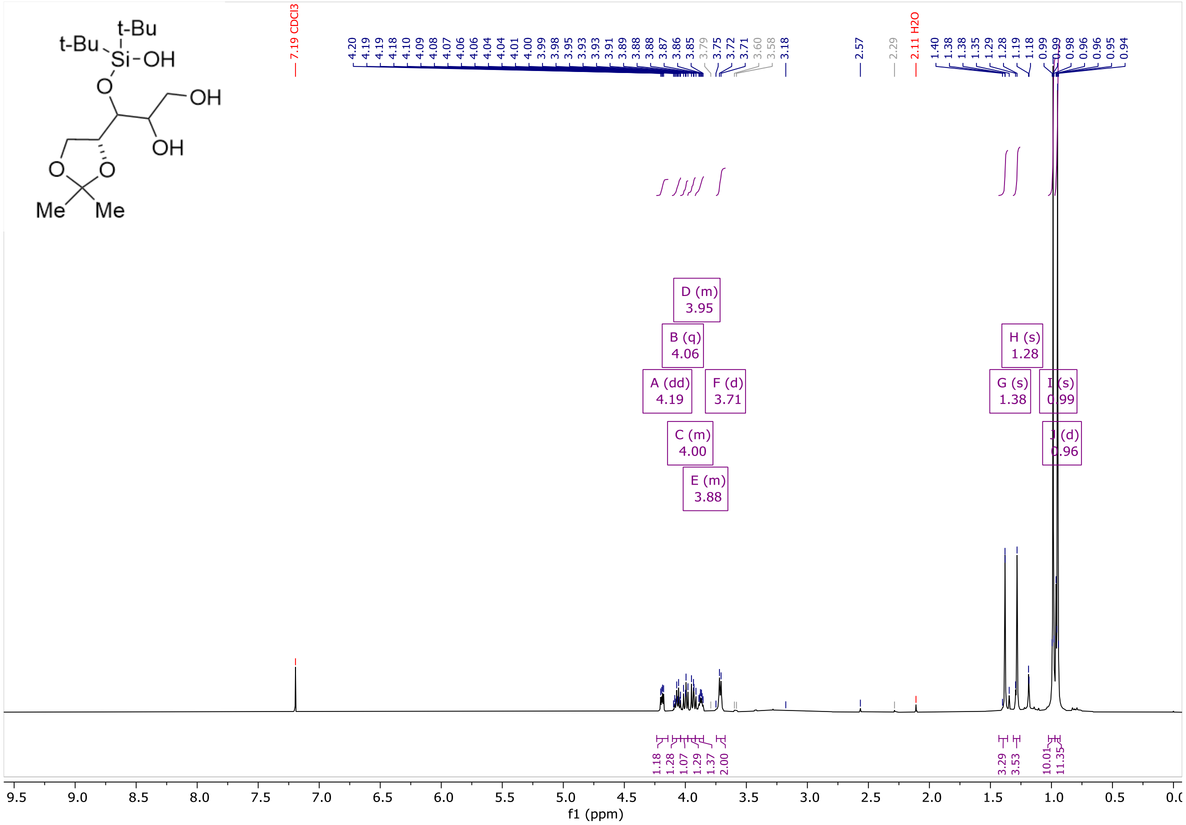

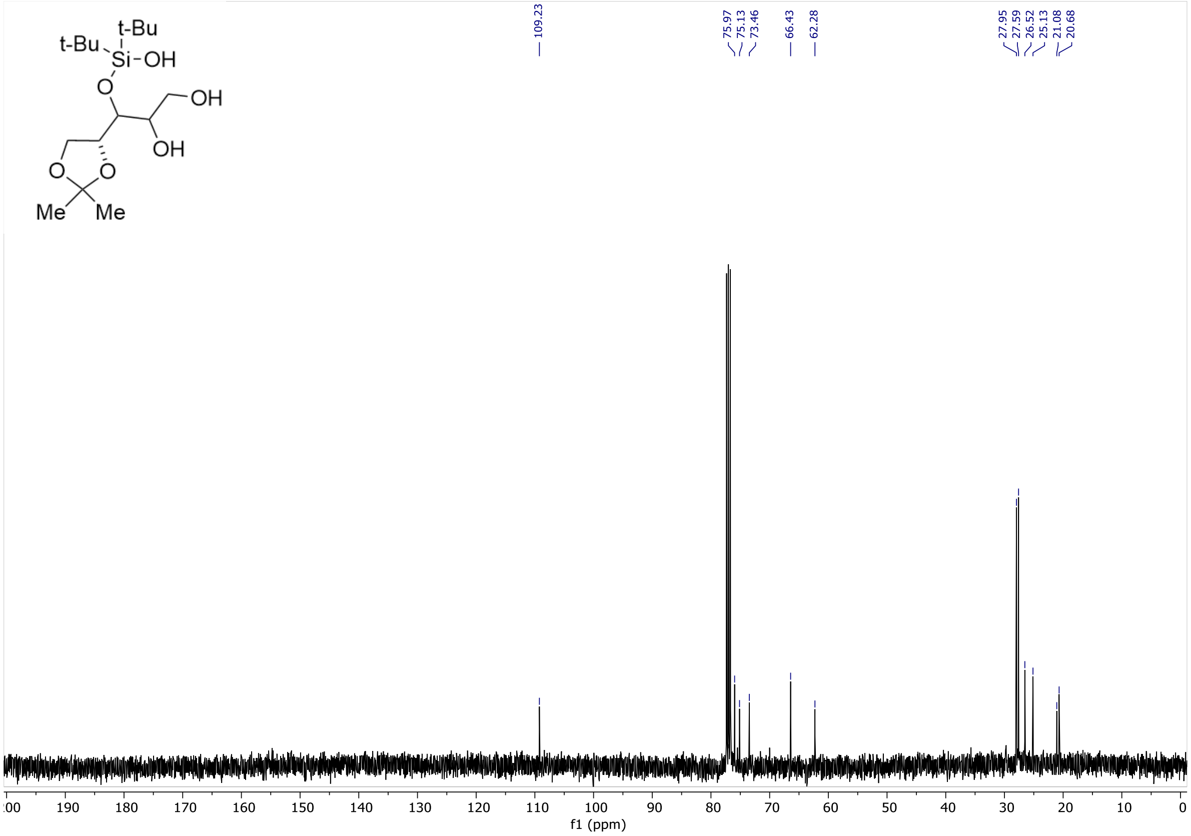

Supplement: Supplementary file 1 [file molecules-26-03829-s001.zip › molecules-1272754-supplementary/SI/Supporting Information-fs-1-17.pdf]
